# Towards a general diastereoselective route to oxabicyclo[3.2.1]octanes via a gold-catalysed cascade reaction

**DOI:** 10.1038/ncomms9617

**Published:** 2015-10-16

**Authors:** Junkai Fu, Yueqing Gu, Hao Yuan, Tuoping Luo, Song Liu, Yu Lan, Jianxian Gong, Zhen Yang

**Affiliations:** 1 Laboratory of Chemical Genomics, School of Chemical Biology and Biotechnology, Peking University Shenzhen Graduate School, Shenzhen 518055, China; 2 Key Laboratory of Bioorganic Chemistry and Molecular Engineering of Ministry of Education, Beijing National Laboratory for Molecular Science (BNLMS) and Peking-Tsinghua Center for Life Sciences, Peking University, Beijing 100871, China; 3 School of Chemistry and Chemical Engineering, Chongqing University, Chongqing 400030, China; 4 Key Laboratory of Marine Drugs, Chinese Ministry of Education, School of Medicine and Pharmacy, Ocean University of China, 5 Yushan Road, Qingdao 266003, China

## Abstract

The development of an efficient diastereoselective synthesis of the oxabicyclo[3.2.1]octane ring system bearing two oxygenated quaternary chiral centres represents a significant challenge. This motif can be found in a wide range of natural products with significant biological activities. Here we report the synthesis of such kind of scaffold using a cyclohexane-*trans*-1,4-diol with an alkyne side chain in the presence of Au(I) catalyst. This is a domino process in which two C–H, two C–O and one C–C bond is assembled through a sequence of cyclization/semi-pinacol rearrangements. This strategy has been successfully applied to the asymmetric formal total synthesis of (+)-cortistatins.

## Abstract (web summary)

Oxygenated bicyclic cores are common to many natural and bioactive compounds, but their efficient synthesis can be difficult. Here, the authors report a gold-catalysed diastereoselective process for the synthesis of oxabicyclo[3.2.1]octanes via a domino process.

## Graphical abstract

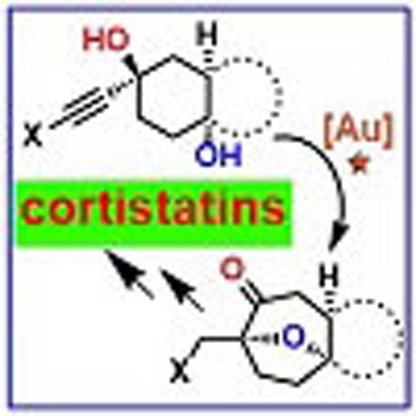

A wide variety of intriguing natural products with 8-oxabicyclo[3.2.1]octane motifs (**1**–**8**, Fig. 1) have been shown to exhibit significant biological activities.

The cortistatins (**1**–**4**) are a family of 11 steroidal alkaloids, with unique structures and prominent biological activities^1^,^2^. They were isolated from the marine sponge *Corticium simplex* by the Kobayashi group in 2006 and 2007. Among them, cortistatin A (**1**) has proved to be the strongest inhibitor of the migration and proliferation of human umbilical vein endothelial cells at concentrations as low as 100 pM, and with a therapeutic index of over 3,300. Englerin A (**5**) is isolated from the stem bark of *Phyllanthus engleri* in Tanzania. It is a guaiane sesquiterpene and has been reported to selectively inhibit the growth of renal cancer cell lines at the nanomolar level^3^. Platensimycin (**6**) comes from the fermentation broth of *Streptomyces platensis*. It is a broad-spectrum antibiotic against Gram-positive bacteria and exerts its antibacterial effect by selectively inhibiting the β-ketoacyl-(acyl-carrier-protein)synthase (FabF), one of the key enzymes in bacterial fatty acid biosynthesis^4^,^5^. Hedyosumin C (**7**), isolated from *Hedyosmum orientale*, represents a novel type of sesquiterpenoid with cytotoxic activity against human lung adenocarcinoma and leukaemia tumour cell lines^6^. Linearol (**8**), isolated from dried *Dictyota Indica*, has a significant inhibitory effect on herbivores^7^.

The biological properties, together with their complex structures have elevated them to be prominent targets for total synthesis^8^,^9^,^10^,^11^,^12^,^13^. However, the challenges associated with the diastereoselective synthesis of the 8-oxabicyclo[3.2.1]octane core reside in construction of the two oxygenic quaternary chiral centres. A general diastereoselective route for the development of divergent synthetic processes to produce assortments of skeletally diverse and densely functionalized oxabicyclo[3.2.1]octane scaffolds is always highly desirable, despite the excellent progress made thus far^14^,^15^,^16^,^17^,^18^,^19^,^20^,^21^,^22^,^23^,^24^. Herein we report a general strategy using a semi-pinacol rearrangement cascade reaction of substituted 1-ethynylcyclohexane-*trans*-1,4-diols. This goes via a highly strained oxonium ion generated *in situ* and is derived from nucleophilic addition of a hydroxyl group onto a gold-activated alkyne. This chemistry has been successfully applied to the asymmetric formal total synthesis of (+)-cortistatins.

The cascade reaction^25^ allows several bond-forming and/or -cleaving events to occur in a single synthetic operation, thus minimizing the costs and waste. Over the past 10 years, gold catalysts have risen to the forefront of cycloisomerization due to their high activities and the mild reaction conditions, and a wide variety of organic scaffolds are available by these methods^26^,^27^,^28^,^29^. As a soft Lewis-acid catalyst, gold complex could selectively activate alkynes and promote the addition of nucleophiles^30^. In this context, the intramolecular nucleophilic addition of a hydroxyl group to a Au-activated carbon–carbon triple bond, followed by a compatible synthetic transformation has proven to be a powerful method for the synthesis of structurally diverse scaffolds (Fig. 2a)^31^,^32^,^33^.

On the other hand, 1,2-alkyl migration has been considered as a key element in numbers of novel gold-catalysed cascade reactions^34^, and it could be induced through oxonium ions^35^,^36^ or gold carbenoids^37^,^38^,^39^,^40^. Recently, the electrophile-induced intermolecular addition of enol ether, followed by semi-pinacol-type 1,2-migration of the resulting oxonium ions has been well documented to be a powerful method to synthesize several complex natural products by Tu and colleagues^41^ (Fig. 2b). In accordance with our goal, an oxygenated quaternary chiral center was generated from the corresponding hydroxyl vinyl ether.

On the basis of previous work in our laboratory using gold-catalysed tandem reactions of alkynes with internal nucleophiles^42^,^43^, we wondered if oxabicyclo[3.2.1]octane scaffolds **D** could be constructed from 1-ethynyl-cyclohexane-*trans*-1,4-diol **A** via a gold-catalysed cascade reaction (Fig. 2c). Formation of the tricyclic oxabicyclo[3.2.1]octane scaffold **D** was envisioned to come about from an intramolecular 6-exo-dig nucleophilic addition of the OH group in substrate **A** onto the Au-activated triple bond, and the resultant exo-cyclic enol ether **B** was expected to undergo an isomerization to the highly strained oxonium ion **C**, followed by a semi-pinacol-type 1,2-alkyl migration (path a) to give the oxabicyclo[3.2.1]octane scaffold **D**, or via a β-hydrogen elimination (path b) to the bicyclic product **E**. Whilst this proposed cascade reaction is unknown, there have been reports of Au-catalysed nucleophilic addition of hydroxyl groups to alkynes^44^, Au-catalysed isomerization of an exo-cyclic enol ether^45^,^46^ and the oxonium-induced semi-pinacol rearrangement^34^,^41^.

## Results

### Optimization of reaction conditions

**Figure 2:**
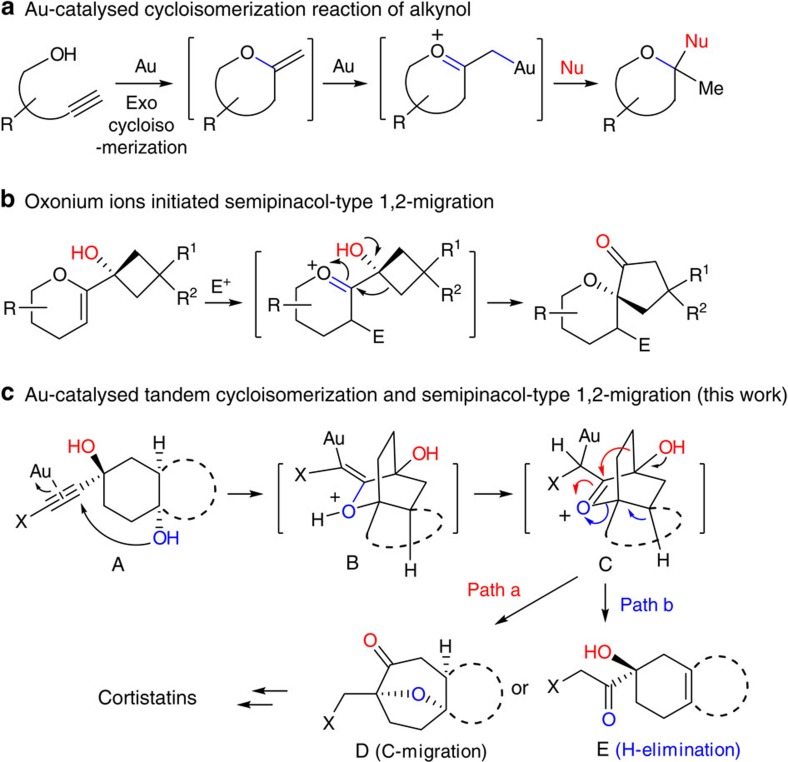
Gold-catalysed sequential reactions. (**a**) Au-catalysed cycloisomerization of alkynol to generate structurally diverse oxacycles; (**b**) semi-pinacol-type 1,2-migration of oxonium ions to construct oxaspirocycles; (**c**) our strategy to access oxabicyclo[3.2.1]octane ring systems via Au-catalysed annulations and the application to cortistatins synthesis.

Model substrates were first used to test the feasibility of the proposed strategy outlined in Fig. 2 (Table 1). To this end, 1-ethynyldecahydro-naphthalene-1,4-diol **9** was treated with AuCl and AuCl_3_ in 1,2-dichloroethane at room temperature. This gave the desired 8-oxabicyclo[3.2.1]octane **10** in 69% and 48%, respectively (entries 1 and 2; Table 1). Tests revealed Ph_3_PAuNTf_2_ (5 mol%) to be the optimum gold catalyst when used at room temperature for 2 h and gave **10** in 72% yield (entries 3–6; Table 1). In terms of solvent, dichloromethane (DCM) gave better results than dichloroethane and toluene (entries 7 and 8). Decreasing the loading of Ph_3_PAuNTf_2_ had little effect on the yield (entry 9). Platinum catalysts can also be used for alkyne-based hydroelement addition reactions. However, in this case only starting material was recovered when K_2_PtCl_6_ was used and for PtCl_2_ a yield of only 28% was observed (entries 10 and 11). Alternative Lewis acid or Brønsted acid, such as *N*-iodosuccinimide or *p*-toluenesulfonamide had no effect on the reaction, however substrate decomposition was observed when the reaction time was extended or the temperature increased (entries 12 and 13). A control experiment in the absence of gold catalyst gave no desired product, indicating that Ph_3_PAuNTf_2_ is essential (entry 14). In light of these results, the optimized conditions for this reaction are Ph_3_PAuNTf_2_ (2.5 mol%) in DCM at room temperature. The experimental conditions were particularly practical because neither flame-dried glassware, an inert atmosphere nor carefully dried solvent were required.

### Substrate scope

A number of substituted 1-ethynyldecahydronaphthalene-1,4-diols (Table 2) were used to assess the generality of this reaction. These substrates bearing electron-withdrawing groups, such as bromide, iodide and ester on the terminal alkyne were synthesized (**11a**–**11c**). The corresponding products **12a**–**12c** were obtained in good yields, allowing the further functional group inter-conversion to facilitate the synthetic transformation (entries 1–3). In addition, when the terminal alkynes had phenyl, allyl or alkyl substituents (**11d**–**11f**), the expected annulated products (**12d**–**12f**) were formed albeit in slightly lower yields (entries 4–6). Notably, when the alkyne moiety had an alkyl group, hydrogen elimination was a potential competing reaction and gave **13f** in 21% yield (entry 6).

Methoxyethoxymethyl (MEM) protection of the hydroxyl group at C4 led to product **12g** in 65% yield, indicating the reaction might proceed via the intramolecular nucleophilic addition of the MEM ether to the Au-activated alkyne, followed by deprotection of the MEM group (entry 7). Furthermore, the [6,5]-fused bicyclic substrates also underwent the sequential reaction to give the desired products in excellent yields (entries 8–11). The relative stereochemistry of **12k** was confirmed by X-ray crystallography. The bulky alkyl group and electronic effect of the ester in substrates **11c**, **11f** and **11k** meant that the reaction had to be carried out at higher temperature (70 °C) to accelerate the addition of the tertiary hydroxyl group to the triple bond.

**Figure 3:**
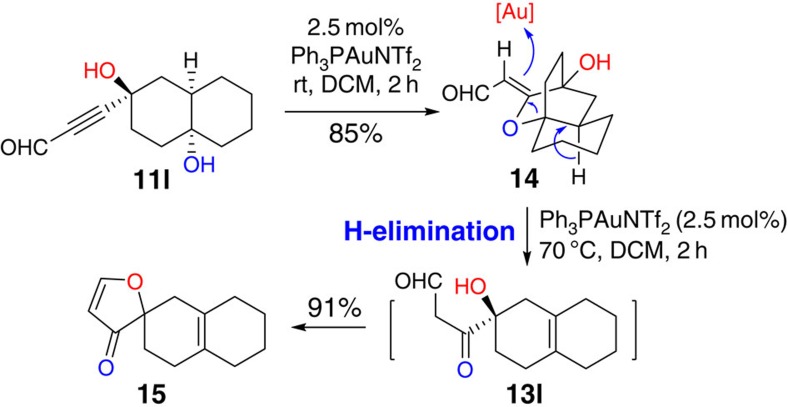
H-elimination pathway for the propargyl aldehyde substrate. A cyclization intermediate **14** could be isolated from the Au-catalysed reaction of propargyl aldehyde substrate **11l**. Further treatment with gold catalyst in higher temperature, **14** could undergo H-elimination, followed by a condensation to afford spiro-compound **15**.

It is worthwhile to mention that when substrate **11l** was used, **14** was obtained in 85% yield. This supports our proposed formation of intermediate **B** (Fig. 2c). Interestingly, further treatment of **14** with gold catalyst at high temperature (70 °C) afforded **15** in 91% yield, presumably through intermediate **13l** (Fig. 3). Alternative acids, such as TsOH, HCl, AcOH, AlCl_3_ and AlMe_3_, all failed to promote this transformation.

The Au-catalysed sequential reaction of monocyclic diol substrates were also tested (Table 3) for the synthesis of 8-oxabicyclo[3.2.1]octane derivatives equipped with several functional groups. These are useful in the synthesis of natural products containing this basic skeleton, such as (−)-englerin A (**5**), platensimycin (**6**), hedyosumin C (**7**) and linearol (**8**), and also for the preparation of a variety of valuable functionalized cyclic compounds.

The reaction of substrates **16a**–**16h** generated desired products **17a**–**17h** in good yields (Table 3). Substrates bearing a terminal alkyne and those substituted with halogen atoms or an ester tolerated the reaction conditions. These groups allow further functional group manipulation (entries 1–4; Table 3). R group has proven to be compatible with a variety of functional groups, such as alkyl group, allyl group or even just H atom (entries 5–8). In the case where R=H (entry 8), a higher temperature was required. This may be because the distance between the secondary hydroxyl group and the alkyne is greater than that in the case of tertiary alcohol-based substrates.

### Mechanistic investigation

**Figure 4:**
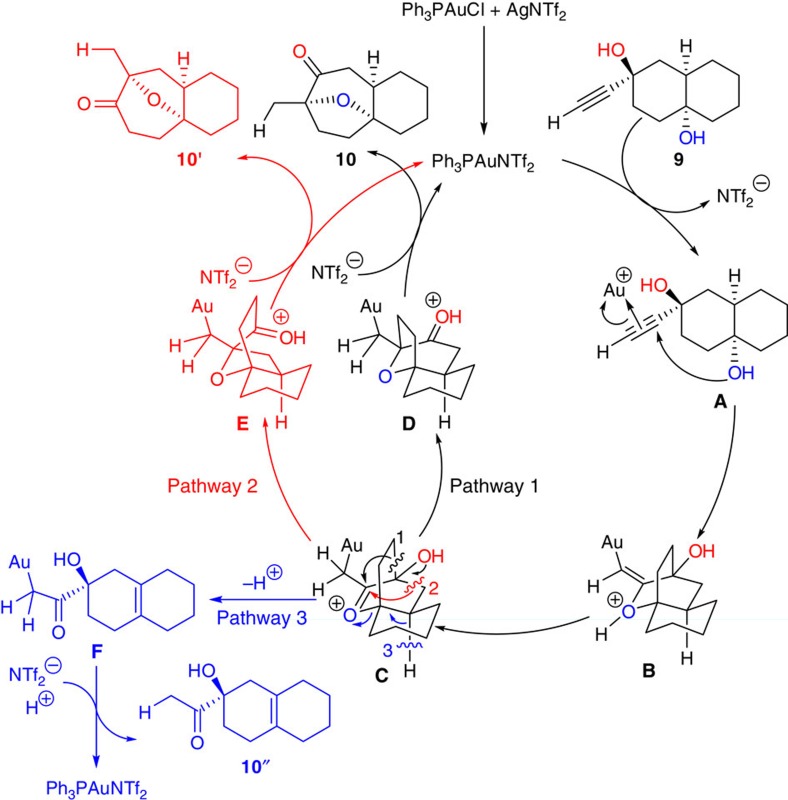
Proposed mechanism for the Au-catalysed annulation reaction. The proposed mechanism involves the sequential intramolecular nucleophilic addition/ 1,3-proton shift/ oxonium ion-initiated semi-pinacol rearrangement (pathways 1 and 2) or hydrogen elimination (pathway 3).

In our proposed mechanism for this Au-catalysed cascade reaction (Fig. 4), the alkyne moiety of the substrate is first activated by the active catalyst Ph_3_PAuNTf_2_, which is generated from the reaction between Ph_3_PAuCl and AgNTf_2_ (ref. 47), to form *π*-coordinated intermediate **A**. The subsequent intramolecular nucleophilic attack by the hydroxyl group takes place to form a [2.2.2] bicyclic intermediate **B**, which would isomerize to a highly strained oxonium intermediate **C** through a proton shift^48^. Oxonium ion-initiated semi-pinacol rearrangement could afford intermediate **D** or **E** through pathway 1 or 2 and release ring strain. The following proton shift would form product **10** or side product **10′**, respectively, and regenerate the active catalyst Ph_3_PAuNTf_2_ (ref. 49).

Alternatively, H-elimination followed by C–O bond cleavage would form intermediate **F**, which could lead to the generation of another side product **10′′** (pathway 3)^50^. Although path 3 was observed in some cases (for substrate **11f** and **11l**), path 2 was never detected. To gain further insights into the mechanism and the origins of the excellent regioselectivity of the semi-pinacol type of 1,2-alkyl migration, we completed detailed density functional theory (DFT) calculations.

### Computational study

**Figure 5:**
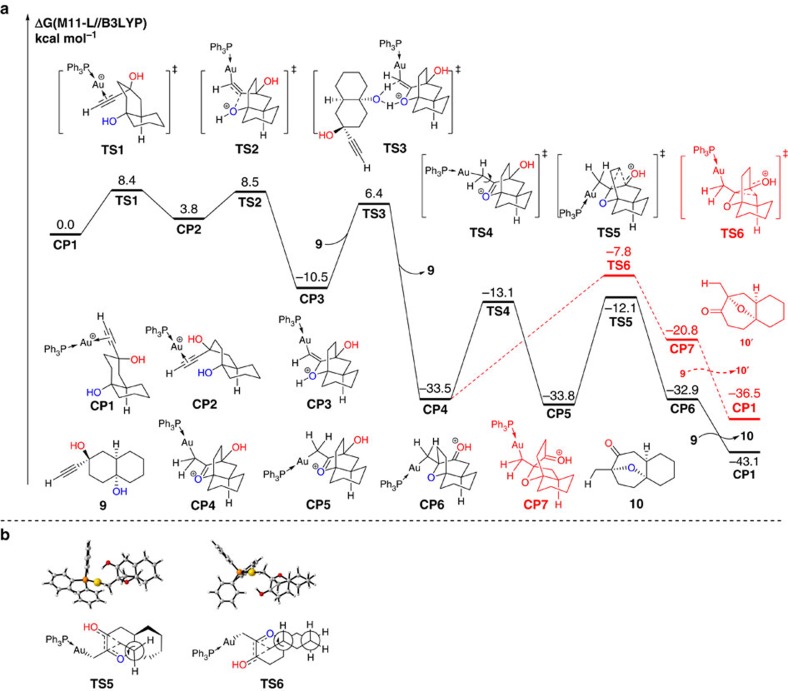
Free-energy profiles for the two competing mechanisms. (**a**) SMD-M11-L/6-311+G(d)//B3LYP/6-31+G(d) (SDD for Au) calculated free-energy profile for the sequence of intramolecular nucleophilic addition/semi-pinacol rearrangement of 1-ethynylcyclohexane-*trans*-1,4-diols established in this study for the reaction of key intermediate **CP1**. This indicates that the semi-pinacol rearrangement is the rate-determining step of this reaction and formation of complex **10** via transition state **TS5** is both thermodynamically and kinetically favourable (see Supplementary Tables 1 and 2). (**b**) The Newman projections of transition states TS5 and TS6.

The M11-L/6-311+G(d)//B3LYP/6-31+G(d) (Stuttgart/Dresden effective core potentials (SDD) basis set for Au atom) calculated free-energy profiles for the Au-catalysed annulation reaction are shown in Fig. 5a to clarify the mechanisms proposed in Fig. 4. All of the DFT calculations conducted in this study were carried out using the GAUSSIAN 09 series of programs^51^. DFT method B3LYP (refs 52, 53) with a standard 6-31+G(d) basis set (SDD basis set for Au) was used for the geometry optimizations. The M11-L functional, proposed by Peverati and Truhlar^54^, was used with a 6-311+G(d) basis set (SDD basis set for Au) to calculate the single point energies. The solvent effects were taken into consideration using single point calculations based on the gas-phase stationary points with a SMD continuum solvation model^55^,^56^,^57^. The energies presented in this paper are the M11-L calculated Gibbs free energies in a DCM solvent with B3LYP calculated thermodynamic corrections.

Initially, the alkynediol-coordinated Au-complex **CP1** was set to the relative zero of the relative free energy. The ring flip, which gives intermediate **CP2** with boat conformation, occurs via a twisted boat transition state **TS1** with a barrier of 8.4 kcal mol^−1^. The Au-activated alkynediol **CP2** might undergo an intramolecular nucleophilic addition of its hydroxyl group to the triple bond in an exo-selective manner to give oxinum ionic intermediate **CP3**, via transition state **TS2** with an energetic span of 4.7 kcal mol^−1^. The intermolecular hydrogen shift between intermediate **CP3** and **9** (or water^58^) irreversibly gives carbocation intermediate **CP4**, via transition state **TS3** with an energetic span of 16.9 kcal mol^−1^. Subsequently, the conformational isomerization of **CP4** gives carbocation intermediate **CP5**, via transition state **TS4** with a barrier of 20.4 kcal mol^−1^. The carbocation intermediate **CP5** undergoes a semi-pinacol rearrangement via transition state **TS5** with a barrier of 21.7 kcal mol^−1^ to give intermediate **CP6**. The following proton shift and coordination of **9** gives the major product **10** with 31.0 kcal mol^−1^ exothermic. Alternatively, side product **10′** would also be formed by the semi-pinacol rearrangement of another carbon atom via transition state **TS6** from **CP4**. The relative free energy of transition state **TS6** is 4.3 kcal mol^−1^ higher than that of transition state **TS5**. Therefore, complex **10′** cannot be observed experimentally. This result is consistent with the experimental observation.

The Newman projections of transition states **TS5** and **TS6** are shown in Fig. 5b to further demonstrate the regioselectivity of the semi-pinacol rearrangement, in which the axis in the projections is supposed to rotate accordingly along with the migration. In transition state **TS5**, when the carbon atom migrates, the axis of Newman projection, being located out of the cyclohexane ring, rotates anticlockwise without restriction. In transition state **TS6**, however, the cyclohexane ring restricts the free rotation of the axis on it in Newman projection. Hence, the strain coming from the conformational change of the cyclohexane ring in transition state **TS6** blocks the carbocation rearrangement.

### Formal synthesis of the cortistatins

**Figure 6:**
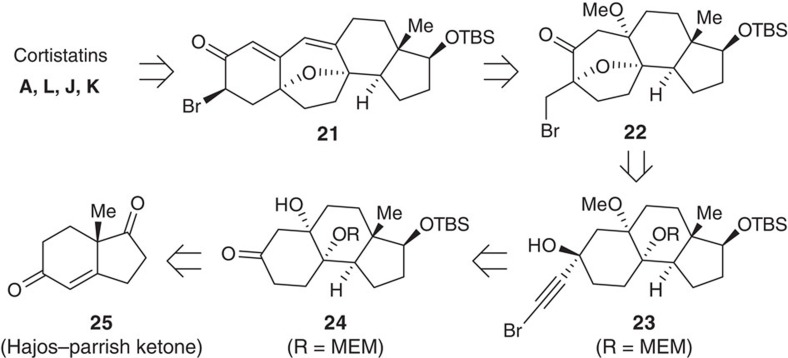
Retrosynthetic analysis of cortistatins. The current Au-catalysed annulation reaction was used as a key step to construct the oxabicyclo[3.2.1]octane ring.

The high yields and mild reaction conditions make this reaction to be a desirable method to construct oxabicyclo[3.2.1]octane scaffolds. These are present in the cortistatins and inspired us to attempt their total synthesis^59^,^60^,^61^,^62^,^63^,^64^,^65^,^66^,^67^. Our retrosynthetic analysis of cortistatins **A**, **L**, **J** and **K** is shown in Fig. 6. Inspiration came from the highly efficient synthesis of cortistatins from tetracyclic intermediate **18** reported by Myers and colleagues^65^. Ketone **19** (Br being a substituent capable of S_N_2 displacement) could be a precursor to intermediate **18**, and it might be synthesized by our gold-catalysed cascade reaction from alcohol **20**, which in turn could be made from ketone **21** via an alkynation. This ketone could be derived from Hajos–Parrish ketone **22**.

**Figure 7:**
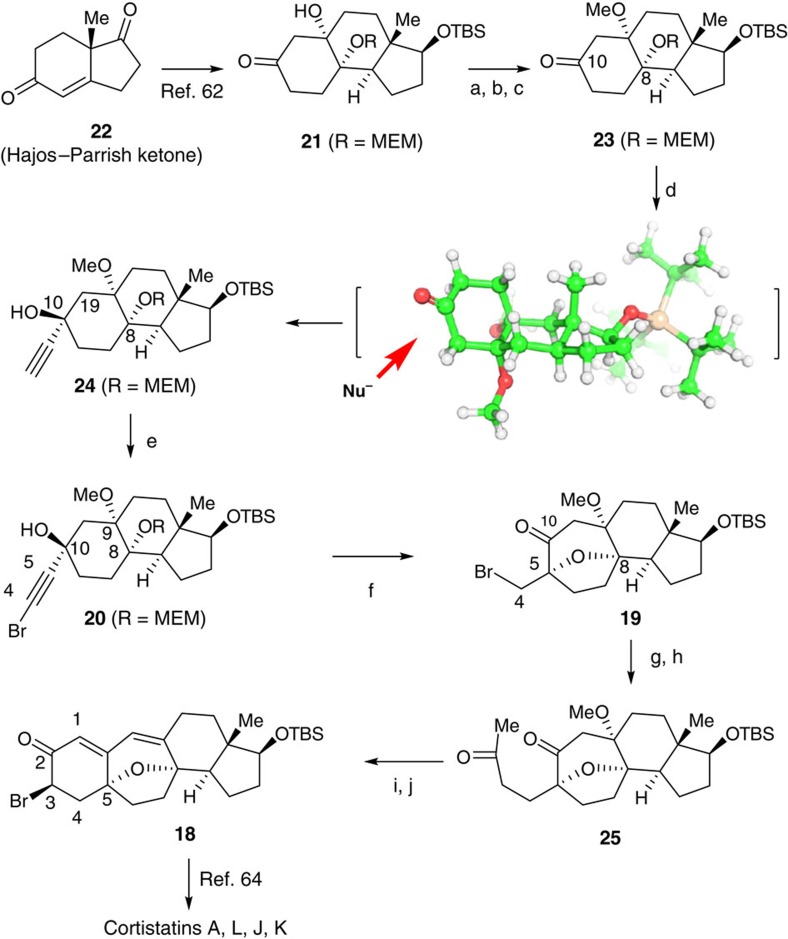
Formal total synthesis of the cortistatins. (**a**) 2,2-Dimethylpropane-1,3-diol, Pyridinium p-Toluenesulfonate (PPTS) benzene, 50 °C, 4 h, 80%; (**b**) KH, MeI, THF, 0 °C to room temperature (rt), 92%; (**c**) CAN, borate buffer (pH=8.0), 60 °C, 5 h, 55%; (**d**) ethynylmagnesium chloride, THF, 0 °C to rt, 75%+20% diastereomer; (**e**) NBS, AgNO_3_, acetone, rt, 0.5 h, 90%; (**f**) Ph_3_PAuNTf_2_ (2.5 mol%), DCM, rt, 1 h, 81%; (**g**) allyl(Bu)_3_Sn, azodiisobutyronitrile (AIBN) benzene, 80 °C, 18 h, 78%; (**h**) PdCl_2_, CuCl, O_2_, DMF-H_2_O (7:1), rt, 12 h, 90%; (**i**) NaOMe, MeOH, rt, 10 h, 70%. (**j**) TMSOTf, Et_3_N, THF, 0 °C, 0.5 h, then NBS, THF, 0 °C, 1 h, 80%.

Our synthesis commenced with known compound **21** (Fig. 7), which was prepared from Hajos–Parrish ketone **22** by a modified procedure reported by Shair and colleagues^63^ (see Supplementary Methods). Initial attempts to protect the tertiary alcohol as the methyl ether were unsuccessful. Decomposition was observed presumably because of the ketone at the β-position of the tertiary hydroxyl group. A stepwise approach was adopted, whereby **21** was first treated with Pyridinium p-Toluenesulfonate (PPTS) in the presence of 2,2-dimethylpropane-1,3-diol and the resultant ketal was then protected as its methyl ether using MeI/KH in tetrahydrofuran (THF). Removal of the ketal group^68^ gave **23** in 40% yield over the three steps.

To prepare intermediate **20**, ketone **23** was reacted with ethynylmagnesium chloride in THF at 0 °C to afford desired product **24** in 75% yield, together with its diastereisomer in 20% yield. The diastereoselective formation of product **24** could be a result of the chair-like transition state adopted by **23**, which might guide the nucleophile approaching from the bottom face of the ketone to achieve the minimum level of steric interaction (see three-dimensional structure of **23** in Fig. 7). Thus, bromination of the alkyne moiety in **24** with *N*-bromosuccinimide (NBS) in the presence of AgNO_3_ in acetone at room temperature gave **20** in 90% yield. Treatment of substrate **20** with Ph_3_PAuNTf_2_ (2.5 mol%) in DCM at room temperature for 1 h, gave desired product **19** in 81% yield.

The structure of **19** was confirmed by X-ray crystallography of its derivative (see Supplementary Fig. 69). Product **19** was subjected to a radical-type allylation by reacting it with allyltributyl stannane in the presence of azodiisobutyronitrile (AIBN) and the resultant terminal alkene was then oxidized to diketone **25** via the Wacker oxidation to give diketone **25** in 70% yield over two steps. Thus, further treatment of diketone **25** with NaOMe in MeOH to initiate a cascade aldol condensation/β-elimination^69^, followed by an α-bromination with NBS in THF at 0 °C afforded **18** in 56% yield over two steps^70^. This compound has been used by Myers and colleagues^65^ in the total synthesis of corstatins **A**, **L**, **J** and **K**.

## Discussion

In summary, a novel strategy for the diastereoselective synthesis of structurally diverse oxabicyclo[3.2.1]octane scaffolds has been achieved via a gold-catalysed cascade reaction, which is consisted of three individual reactions, featuring (1) a gold-catalysed intramolecular nucleophilic addition of the OH group onto the carbon–carbon triple bond; (2) a gold-catalysed isomerization of exo-cyclic enol ether to a highly strained oxonium ion; (3) the oxonium-induced semi-pinacol rearrangement. This chemistry has also been demonstrated in the formal total synthesis of the cortistatins. DFT calculations reveal the necessity for the selective 1,2-alkyl migration to form oxabicyclo[3.2.1]octane scaffolds. Further applications of this methodology to other biologically active natural products with the oxabicyclo[3.2.1]heptane scaffold are currently underway in our laboratory.

## Methods

### Materials

For ^1^H and ^13^C NMR spectra of the compounds in this article, see Supplementary Figs 1–67. For ORTEP diagrams, see Supplementary Figs 68 and 69.

### General

All reactions were conducted in oven-dried glassware under an inert atmosphere of dry nitrogen. All reagents were purchased and used without further purification unless otherwise specified. Solvents for flash column chromatography were technical grade and distilled before use. Analytical thin-layer chromatography was performed using silica gel plates with HSGF 254 (0.15–0.2 mm) manufactured by Shandong Huanghai Chemical Company (Qingdao, China). Visualization was performed by measuring ultraviolet absorbance (254 nm) and using the appropriate stain. Flash column chromatography was performed using Qingdao Haiyang Chemical HG/T2354-92 silica gel (45–75 mm). ^1^H and ^13^C NMR data were recorded on a Bruker 500 MHz (125 MHz for ^13^C NMR) or a Bruker 400 MHz (100 MHz for ^13^C NMR) nuclear resonance spectrometer unless otherwise specified. Chemical shifts (δ) in p.p.m. are reported relative to the residual chloroform signal (^1^H 7.26 p.p.m. and ^13^C 77.16 p.p.m.). Multiplicities are described as follows: s (singlet), bs (broad singlet), d (doublet), t (triplet), q (quartet) and m (multiplet). Coupling constants (*J*) are reported in Hertz (Hz). ^13^C NMR spectra were recorded with total proton decoupling. High-resolution mass spectrometry electrospray ionization analysis was performed by the Analytical Instrumentation Center at Peking University, and High-resolution mass spectrometry data are reported as ion mass/charge (*m/z*) ratios in atomic mass units.

### General procedure for the gold-catalysed cascade reactions

A mixture of Ph_3_PAuNTf_2_ (2.5 mg, 0.005 mmol, 0.025 equiv) and AgNTf_2_ (1.9 mg, 0.005 mmol, 0.025 equiv) in DCM (1.0 ml) was stirred for 0.5 h to generate the active gold catalyst *in situ*. The mixture was then added to a stirred solution of the substrate (0.20 mmol, 1.0 equiv) in DCM (4.0 ml) and stirred at ambient temperature for 2 h. The solvent was removed *in vacuo*, and the residue was purified by flash chromatography on silica gel to provide the desired product. For additional procedures see Supplementary Methods.

## Additional information

**Accession codes.** The X-ray crystallographic coordinates for the structures reported in this article have been deposited at the Cambridge Crystallographic Data Centre (CCDC), under deposition number CCDC 1059110 (for **S10**) and 1059111 (for **12k**). These data can be obtained free of charge from the Cambridge Crystallographic Data Centre via http://www.ccdc.cam.ac.uk/data_request/cif.

**How to cite this article:** Fu, J. *et al.* Towards a general diastereoselective route to oxabicyclo[3.2.1]octanes via a gold-catalysed cascade reaction. *Nat. Commun.* 6:8617 doi: 10.1038/ncomms9617 (2015).

## Supplementary Material

Supplementary Information Supplementary Figures 1-69, Supplementary Tables 1-2, Supplementary Methods and Supplementary References

## Figures and Tables

**Figure 1:**
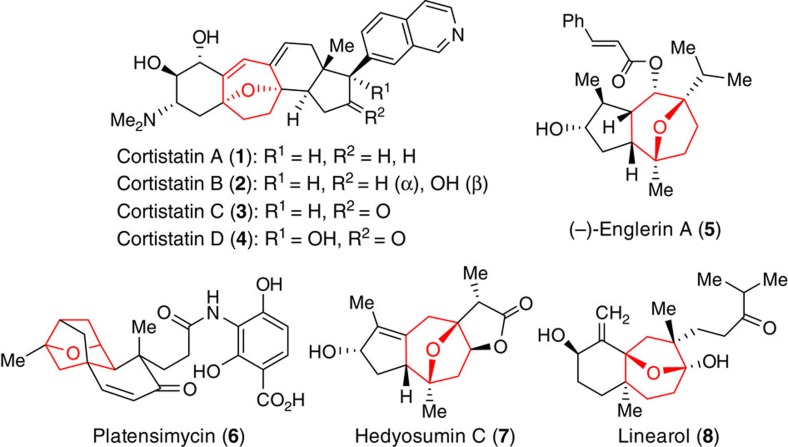
Representative natural products containing oxa-bridged seven-membered carbocycles. Selected biologically active natural products bearing functionalized 8-oxabicyclo[3.2.1]octane rings.

**Table 1:**
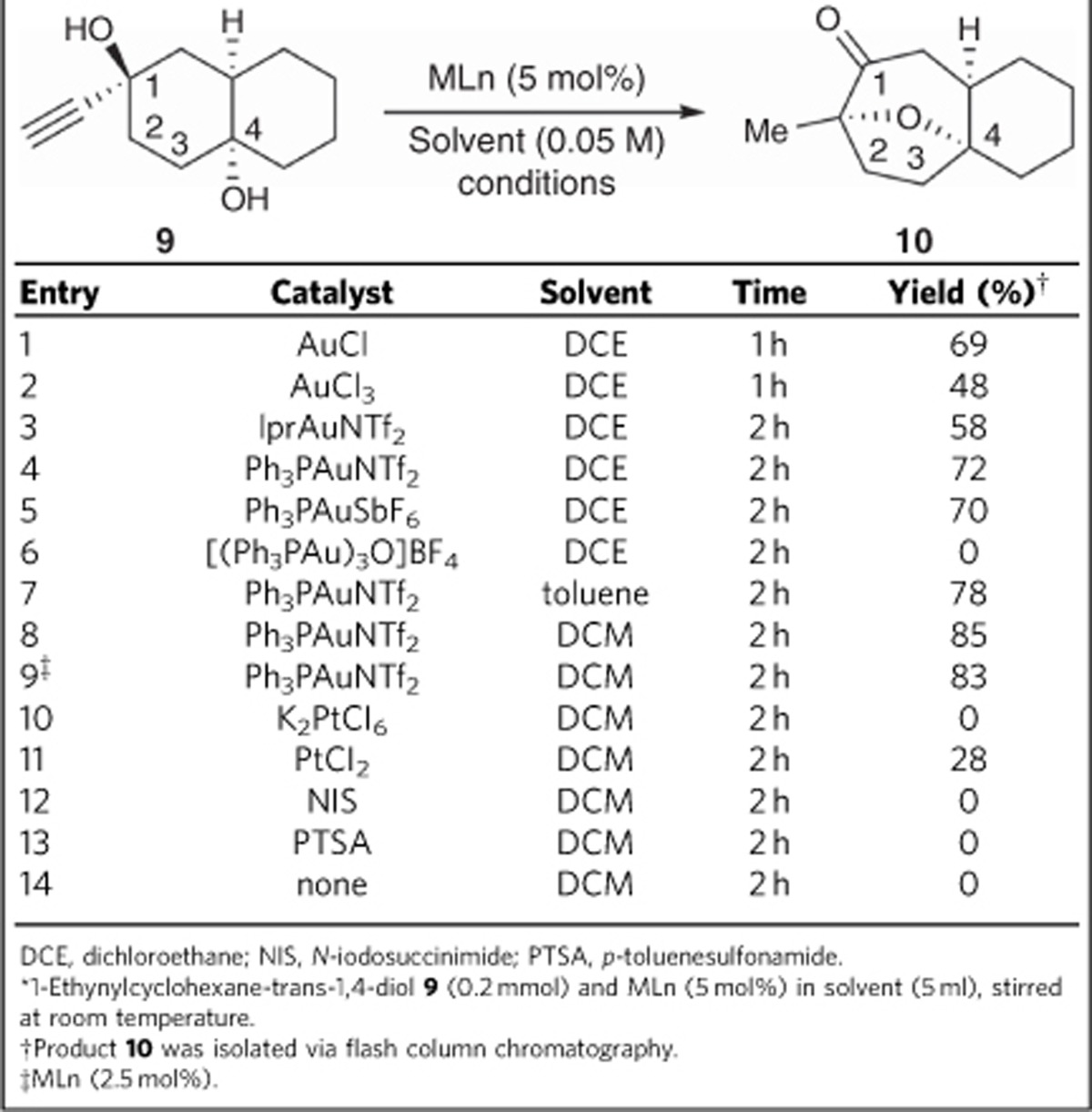
Sequential reaction of diol 9 with various catalysts*.

**Table 2:**
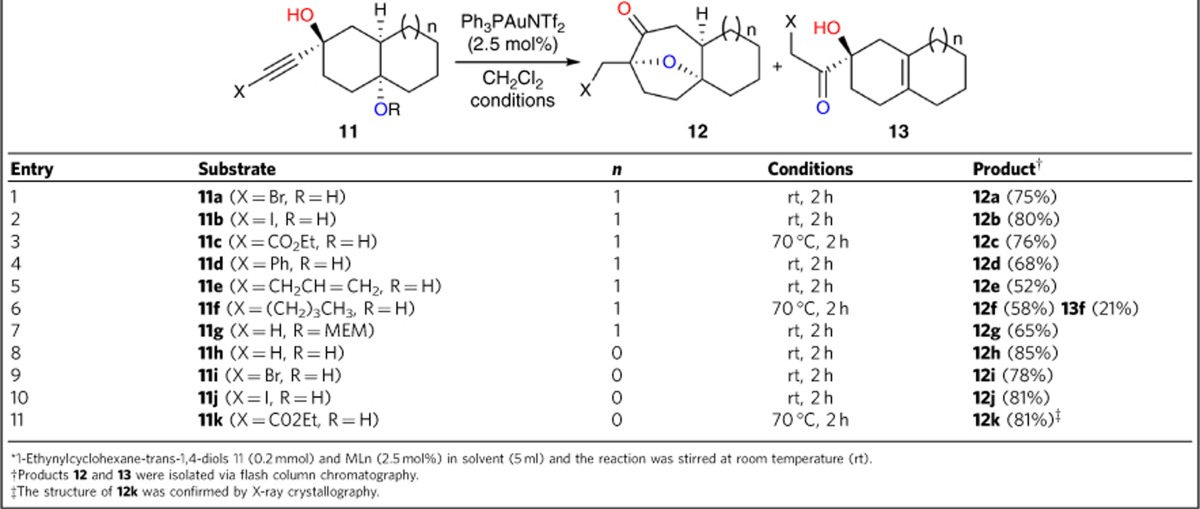
Au-catalysed sequential reactions with alkyne-substituted bicyclic diols*.

**Table 3:**
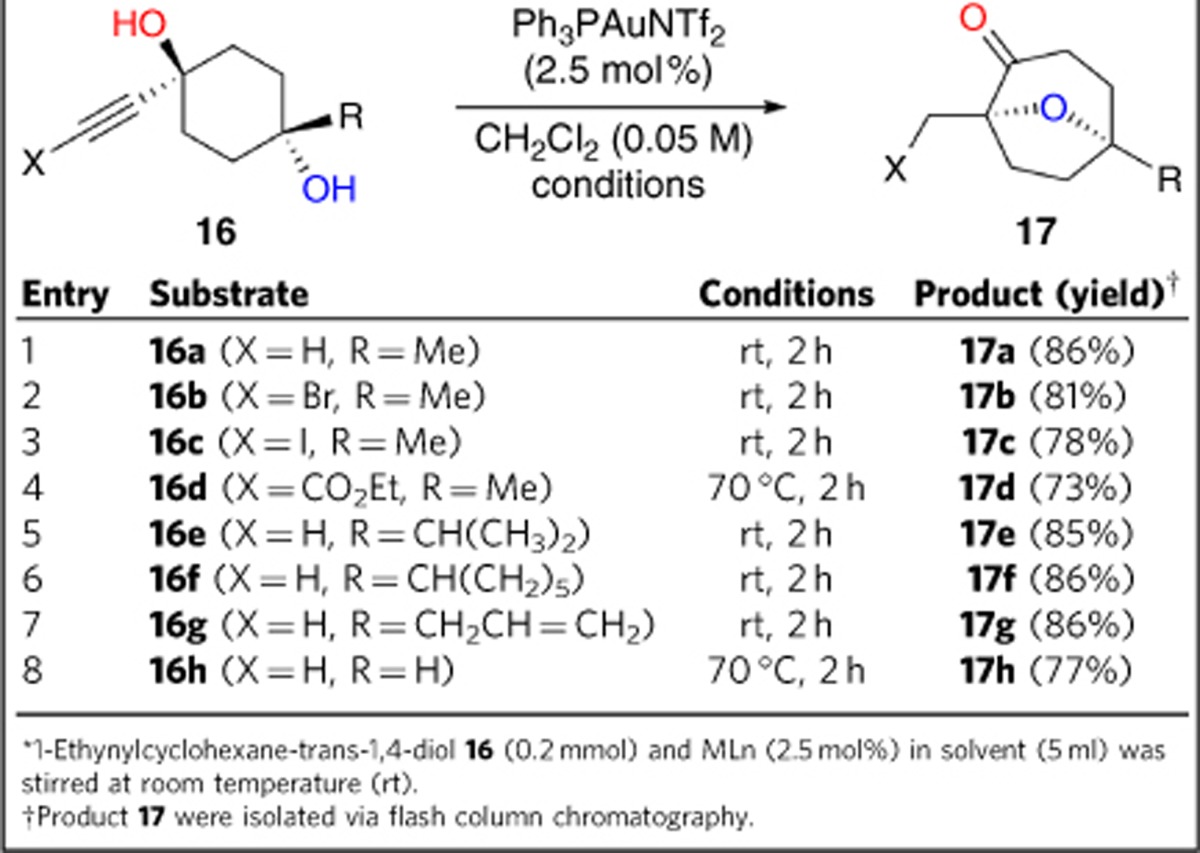
Au-catalysed sequential reactions of alkyne-substituted monocyclic diols*.

## References

[b1] Aoki S. *et al.* Cortistatins A, B, C and D, anti-angiogenic steroidal alkaloids, from the marine sponge *Corticium simplex*. J. Am. Chem. Soc. 128, 3148–3149 (2006). 16522087 10.1021/ja057404h

[b2] Aoki S. *et al.* Structure-activity relationship and biological property of cortistatins, anti-angiogenic spongean steroidal alkaloids. Bioorg. Med. Chem. 15, 6758–6762 (2007). 17765550 10.1016/j.bmc.2007.08.017

[b3] Ratnayake R., Covell D., Ransom T. T., Gustafson K. R. & Beutler J. A. Englerin A, a selective inhibitor of renal cancer cell growth, from *Phyllanthus engleri*. Org. Lett. 11, 57–60 (2009). 19061394 10.1021/ol802339w PMC2651161

[b4] Wang J. *et al.* Platensimycin is a selective FabF inhibitor with potent antibiotic properties. Nature 441, 358–361 (2006). 16710421 10.1038/nature04784

[b5] Zhang C. *et al.* Platensimycin and platencin congeners from *Streptomyces platensis*. J. Nat. Prod. 74, 329–340 (2011). 21214253 10.1021/np100635f

[b6] Su Z.-S. *et al.* Sesquiterpenoids from *Hedyosmum orientale*. J. Nat. Prod. 71, 1410–1413 (2008). 18651771 10.1021/np800240v

[b7] Pereira R. C., Pinheiro M. D., Teixeira V. L. & Gama B. A. P. Feeding preferences of the endemic gastropod Astraea latispina in relation to chemical defenses of Brazilian tropical seaweeds. Braz. J. Biol. 62, 33–40 (2002). 12185921 10.1590/s1519-69842002000100005

[b8] Ylijoki K. E. O. & Stryker J. M. [5+2] Cycloaddition reactions in organic and natural product synthesis. Chem. Rev. 113, 2244–2266 (2013). 23153111 10.1021/cr300087g

[b9] Jones D. E. & Harmata M. Application of the [4+3] Cycloaddition Reaction to the Synthesis of Natural Products, in Methods and Applications of Cycloaddition Reactions in Organic Syntheses ed. Nishiwaki N. John Wiley & Sons, Inc. (2014).

[b10] Jiménez-Núñez E., Claverie C. K., Nieto-Oberhuber C. & Echavarren A. M. Prins cyclizations in Au-catalyzed reactions of enynes. Angew. Chem. Int. Ed. 45, 5452–5455 (2006). 10.1002/anie.200601575 16850511

[b11] Jiménez-Núñez E., Molawi K. & Echavarren A. M. Stereoselective gold-catalyzed cycloaddition of functionalized ketoenynes: synthesis of (+)-orientalol F. Chem. Commun. 7327–7329 (2009). 10.1039/b920119j 20024217

[b12] Molawi K., Delpont N. & Echavarren A. M. Enantioselective synthesis of (−)-englerins A and B. Angew. Chem. Int. Ed. 49, 3517–3519 (2010). 10.1002/anie.201000890 20544903

[b13] Zhou Q., Chen X. & Ma D. Asymmetric, protecting-group-free total synthesis of (−)-englerin A. Angew. Chem. Int. Ed. 49, 3513–3516 (2010). 10.1002/anie.201000888 20544902

[b14] Singh V., Krishna U. M., Vikrant & Trivedi V. G. K. Cycloaddition of oxidopyrylium species in organic synthesis. Tetrahedron 64, 3405–3428 (2008).

[b15] Wender P. A., Lee H. Y., Wilhelm R. S. & Williams P. D. Studies on tumor promoters. 7. The synthesis of a potentially general precursor of the tiglianes, daphnanes, and ingenanes. J. Am. Chem. Soc. 111, 8954–8957 (1989).

[b16] Kitagaki S. *et al.* Enantiocontrol in tandem carbonyl ylide formation and intermolecular 1,3-dipolar cycloaddition of α-diazo ketones mediated by chiral dirhodium(II) carboxylate catalyst. J. Am. Chem. Soc. 121, 1417–1418 (1999).

[b17] Xiong H., Hsung R. P., Berry C. R. & Rameshkumar C. The first epoxidations of 1-amidoallenes. A general entry to nitrogen-substituted oxyallyl cations in highly stereoselective [4+3] cycloadditions. J. Am. Chem. Soc. 123, 7174–7175 (2001). 11459504 10.1021/ja0108638

[b18] Molander G. A., St. Jean D. J. Jr & Haas J. Toward a general route to the eunicellin diterpenes: the asymmetric total synthesis of deacetoxyalcyonin acetate. J. Am. Chem. Soc. 126, 1642–1643 (2004). 14871089 10.1021/ja0398464

[b19] Chung W. K. *et al.* Inter- and intramolecular [4+3] cycloadditions using epoxy enol silanes as functionalized oxyallyl cation precursors. J. Am. Chem. Soc. 131, 4556–4557 (2009). 19281161 10.1021/ja807566t

[b20] Ishida K., Kusama H. & Iwasawa N. Enantioselective preparation of 8-oxabicyclo[3.2.1]octane derivatives via asymmetric [3+2]-cycloaddition of platinum-containing carbonyl ylides with vinyl ethers. J. Am. Chem. Soc. 132, 8842–8843 (2010). 20540576 10.1021/ja102391t

[b21] Burns N. Z., Witten M. R. & Jacobsen E. N. Dual catalysis in enantioselective oxidopyrylium-based [5+2] cycloadditions. J. Am. Chem. Soc. 133, 14578–14581 (2011). 21848300 10.1021/ja206997e PMC3173575

[b22] Li B., Zhao Y.-J., Lai Y.-C. & Loh T.-P. Asymmetric syntheses of 8-oxabicyclo[3,2,1]octanes: a cationic cascade cyclization. Angew. Chem. Int. Ed. 51, 8041–8045 (2012). 10.1002/anie.201202699 22865563

[b23] Obradors C. & Echavarren A. M. Intermolecular gold-catalyzed cycloaddition of alkynes with oxoalkenes. Chem. Eur. J. 19, 3547–3551 (2013). 23424154 10.1002/chem.201300131

[b24] Faustino H., Alonso I., Mascareñas J. L. & López F. Gold(I)-catalyzed cascade cycloadditions between allenamides and carbonyl-tethered alkenes: an enantioselective approach to oxa-bridged medium-sized carbocycles. Angew. Chem. Int. Ed. 52, 6526–6530 (2013). 10.1002/anie.201302713 23653229

[b25] Nicolaou K. C., Edmonds D. J. & Bulger P. G. Cascade reactions in total synthesis. Angew. Chem. Int. Ed. 45, 7134–7186 (2006). 10.1002/anie.200601872 17075967

[b26] Friend C. M. & Hashmi A. S. K. Gold catalysis. Acc. Chem. Res. 47, 729–730 (2014). 24635456 10.1021/ar5000506

[b27] Hashmi A. S. K. & Rudolph M. Gold catalysis in total synthesis. Chem. Soc. Rev. 37, 1766–1775 (2008). 18762826 10.1039/b615629k

[b28] Rudolph M. & Hashmi A. S. K. Gold catalysis in total synthesis–an update. Chem. Soc. Rev. 41, 2448–2462 (2012). 22182942 10.1039/c1cs15279c

[b29] Fürstner A. From understanding to prediction: Gold- and platinum-based π–acid catalysis for target oriented synthesis. Acc. Chem. Res. 47, 925–938 (2014). 24279341 10.1021/ar4001789

[b30] Dorel R. & Echavarren A. M. Gold(I)-catalyzed activation of alkynes for the construction of molecular complexity. Chem. Rev. 115, 9028–9072 (2015). 25844920 10.1021/cr500691k PMC4580024

[b31] Hashmi A. S. K. Gold-catalyzed organic reactions. Chem. Rev. 107, 3180–3211 (2007). 17580975 10.1021/cr000436x

[b32] Hashmi A. S. K. & Hutchings G. J. Gold catalysis. Angew. Chem. Int. Ed. 45, 7896–7936 (2006). 10.1002/anie.200602454 17131371

[b33] Hashmi A. S. K. *et al.* Gold catalysis: tandem reactions of diyne–diols and external nucleophiles as an easy access to tricyclic cage-like structures. Chem. Eur. J. 16, 9846–9854 (2010). 20632335 10.1002/chem.201001322

[b34] Crone B. & Kirsch S. F. 1,2-Alkyl migration as a key element in the invention of cascade reactions catalyzed by π–acids. Chem. Eur. J. 14, 3514–3522 (2008). 18318026 10.1002/chem.200701985

[b35] Kirsch S. F., Binder J. T., Liébert C. & Menz H. Gold(III)- and platinum(II)-catalyzed domino reaction consisting of heterocyclization and 1,2-migration: efficient synthesis of highly substituted 3(2H)-furanones. Angew. Chem. Int. Ed. 45, 5878–5880 (2006). 10.1002/anie.200601836 16871607

[b36] Zhang J. & Schmalz H.-G. Gold(I)-catalyzed reaction of 1-(1-alkynyl)-cyclopropyl ketones with nucleophiles: a modular entry to highly substituted furans. Angew. Chem. Int. Ed. 45, 6704–6707 (2006). 10.1002/anie.200601252 16986180

[b37] Wang T. *et al.* Synthesis of highly substituted 3-formylfurans by a gold(I)-catalyzed oxidation/1,2-alkynyl migration/cyclization cascade. Angew. Chem. Int. Ed. 53, 3715–3719 (2014). 10.1002/anie.201310146 24616030

[b38] Wang T., Huang L., Shi S., Rudolph M. & Hashmi A. S. K. Synthesis of highly substituted *N*-(furan-3-ylmethylene)benzenesulfonamides by a gold(I)-catalyzed oxidation/1,2-alkynyl migration/cyclization cascade. Chem. Eur. J. 20, 14868–14871 (2014). 25236914 10.1002/chem.201404229

[b39] Nösel P. *et al.* 1,6-Carbene transfer: gold-catalyzed oxidative diyne cyclizations. J. Am. Chem. Soc. 135, 15662–15666 (2013). 24050384 10.1021/ja4085385

[b40] Lauterbach T. *et al.* Gold-Catalyzed carbenoid transfer reactions of diynes – pinacol rearrangement *versus* retro-Buchner reaction. Adv. Synth. Catal. 357, 775–781 (2015).

[b41] Song Z.-L., Fan C.-A. & Tu Y.-Q. Semipinacol rearrangement in natural product synthesis. Chem. Rev. 111, 7523–7556 (2011). 21851053 10.1021/cr200055g

[b42] Shi H. *et al.* Total syntheses of Drimane-type sesquiterpenoids enabled by a gold-catalyzed tandem reaction. J. Am. Chem. Soc. 133, 14944–14947 (2011). 21861520 10.1021/ja206837j

[b43] Yue G. *et al.* Collective synthesis of cladiellins based on the gold-catalyzed cascade reaction of 1,7-diynes. Angew. Chem. Int. Ed. 53, 1837–1840 (2014). 10.1002/anie.201309449 24474595

[b44] Widenhoefer R. A. & Song F. Gold-Catalyzed Addition of Oxygen Nucleophiles to C-C Multiple Bonds, in Catalyzed Carbon-Heteroatom Bond Formation ed. Yudin A. K. Wiley-VCH Verlag GmbH & Co. KGaA (2010).

[b45] Barluenga J., Diéguez A., Fernández A., Rodríguez F. & Fañanás F. J. Gold- or platinum-catalyzed tandem cycloisomerization/Prins-type cyclization reactions. Angew. Chem. Int. Ed. 45, 2091–2093 (2006). 10.1002/anie.200503874 16498691

[b46] Barluenga J., Fernández A., Diéguez A., Rodríguez F. & Fañanás F. J. Gold- or platinum-catalyzed cascade processes of alkynol derivatives involving hydroalkoxylation reactions followed by Prins-type cyclizations. Chem. Eur. J. 15, 11660–11667 (2009). 19760715 10.1002/chem.200900856

[b47] Mézailles N., Ricard L. & Gagosz F. Phosphine gold(I) bis-(trifluoromethanesulfonyl)imidate complexes as new highly efficient and air-stable catalysts for the cycloisomerization of enynes. Org. Lett. 7, 4133–4136 (2005). 16146370 10.1021/ol0515917

[b48] Krauter C. M., Hashmi A. S. K. & Pernpointner M. A new insight into gold(I)-catalyzed hydration of alkynes: proton transfer. ChemCatChem 2, 1226–1230 (2010).

[b49] Hashmi A. S. K. Homogeneous gold catalysis beyond assumptions and proposals-characterized intermediates. Angew. Chem. Int. Ed. 49, 5232–5241 (2010). 10.1002/anie.200907078 20572216

[b50] Xu M., Ren T.-T. & Li C.-Y. Gold-catalyzed oxidative rearrangement of homopropargylic ether via oxonium ylide. Org. Lett. 14, 4902–4905 (2012). 22954390 10.1021/ol302238t

[b51] Frisch M. J. *et al.* Gaussian 09 Gaussian, Inc., Wallingford, CT (2013).

[b52] Becke A. D. Density-functional thermochemistry. III. The role of exact exchange. J. Chem. Phys. 98, 5648–5652 (1993).

[b53] Lee C., Yang W. & Parr R. G. Development of the Colle-Salvetti correlation-energy formula into a functional of the electron density. Phys. Rev. B 37, 785–789 (1988). 10.1103/physrevb.37.785 9944570

[b54] Peverati R. & Truhlar D. G. Improving the accuracy of hybrid meta-GGA density functionals by range separation. J. Phys. Chem. Lett. 2, 2810–2817 (2011).

[b55] Cances E., Mennunci B. & Tomasi J. A new integral equation formalism for the polarizable continuum model: theoretical background and applications to isotropic and anisotropic dielectrics. J. Chem. Phys. 107, 3032–3041 (1997).

[b56] Cossi M., Barone V., Cammi R. & Tomasi J. Ab initio study of solvated molecules: a new implementation of the polarizable continuum model. J. Chem. Phys. Lett. 255, 327–335 (1996).

[b57] Barone V., Cossi M. & Tomasi J. Geometry optimization of molecular structures in solution by the polarizable continuum model. J. Comput. Chem. 19, 404–417 (1998).

[b58] Xia Y. *et al.* An unexpected role of a trace amount of water in catalyzing proton transfer in phosphine-catalyzed (3+2) cycloaddition of allenoates and alkenes. J. Am. Chem. Soc. 129, 3470–3471 (2007). 17319666 10.1021/ja068215h

[b59] Narayan A. R. H., Simmons E. M. & Sarpong R. Synthetic strategies directed towards the Cortistatin family of natural products. Eur. J. Org. Chem. 2010, 3553–3567 (2010).

[b60] Shenvi R. A., Guerrero C. A., Shi J., Li C.-C. & Baran P. S. Synthesis of (+)-cortistatin A. J. Am. Chem. Soc. 130, 7241–7243 (2008). 18479104 10.1021/ja8023466 PMC2652360

[b61] Shi J. *et al.* Scalable synthesis of cortistatin A and related structures. J. Am. Chem. Soc. 133, 8014–8027 (2011). 21539314 10.1021/ja202103e PMC3119343

[b62] Nicolaou K. C., Sun Y.-P., Peng X.-S., Polet D. & Chen D. Y.-K. Total synthesis of (+)-cortistatin A. Angew. Chem. Int. Ed. 47, 7310–7313 (2008). 10.1002/anie.200803550 18704899

[b63] Lee H. M., Nieto-Oberhuber C. & Shair M. D. Enantioselective synthesis of (+)-cortistatin A, a potent and selective inhibitor of endothelial cell proliferation. J. Am. Chem. Soc. 130, 16864–16866 (2008). 19053422 10.1021/ja8071918

[b64] Nicolaou K. C. *et al.* Total synthesis and biological evaluation of cortistatins A and J and analogues thereof. J. Am. Chem. Soc. 131, 10587–10597 (2009). 19722632 10.1021/ja902939t

[b65] Flyer A. N., Si C. & Myers A. G. Synthesis of cortistatins A, J, K and L. Nat. Chem. 2, 886–892 (2010). 20861906 10.1038/nchem.794 PMC2946095

[b66] Nilson M. G. & Funk R. L. Total synthesis of (±)-cortistatin J from furan. J. Am. Chem. Soc. 133, 12451–12453 (2010). 21761900 10.1021/ja206138d PMC3154988

[b67] Yamashita S., Iso K., Kitajima K., Himuro M. & Hirama M. Total synthesis of cortistatins A and J. J. Org. Chem. 76, 2408–2425 (2011). 21405132 10.1021/jo2002616

[b68] Markó I. E. *et al.* Cerium(IV)-catalyzed deprotection of acetals and ketals under mildly basic conditions. Angew. Chem. Int. Ed. 38, 3207–3209 (1999). 10.1002/(sici)1521-3773(19991102)38:21<3207::aid-anie3207>3.0.co;2-i 10556904

[b69] Tan D. S., Dudley G. B. & Danishefsky S. J. Synthesis of the functionalized tricyclic skeleton of guanacastepene A: a tandem epoxide-opening β-elimination/Knoevenagel cyclization. Angew. Chem. Int. Ed. 41, 2185–2188 (2002). 19746639

[b70] Fang L. *et al.* Formal synthesis of cortistatins. J. Org. Chem. 76, 2479–2487 (2011). 21391624 10.1021/jo102202t

