# Supplementary material for: Towards a general diastereoselective route to oxabicyclo[3.2.1]octanes via a gold-catalysed cascade reaction
Source: Nat Commun. 2015 Oct 16;0:8617. doi: 10.1038/ncomms9617 (PMC4634332; doi:10.1038/ncomms9617)
Supplement: Supplementary Information — Supplementary Figures 1-69, Supplementary Tables 1-2, Supplementary Methods and Supplementary References [file ncomms9617-s1.pdf]

Supplementary Figures

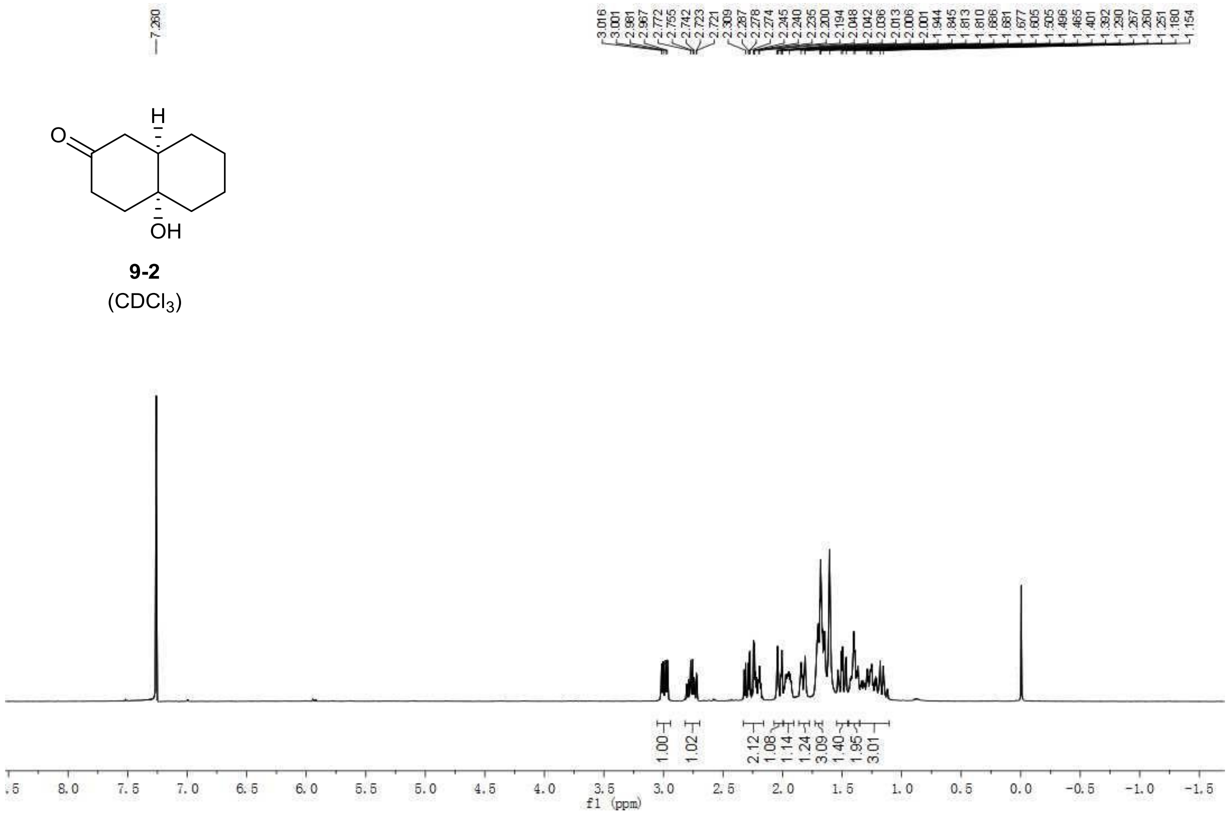

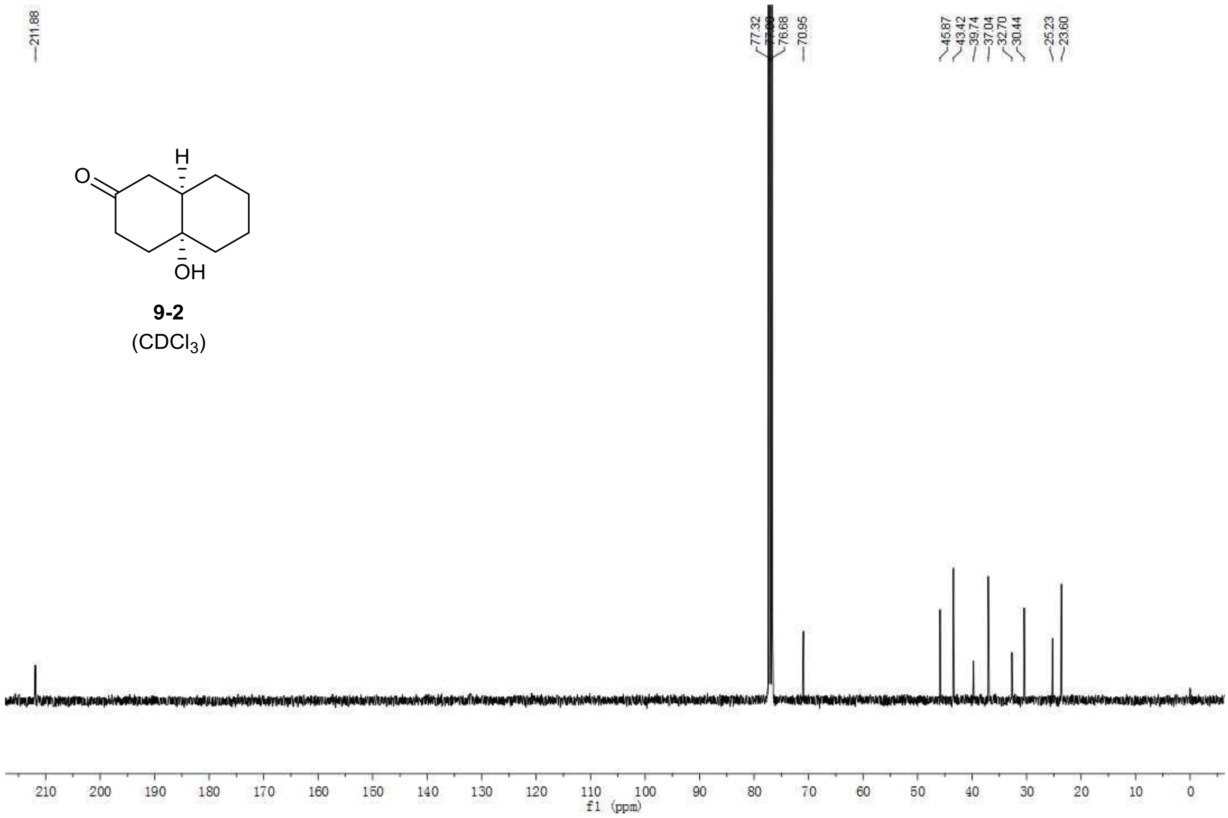

Supplementary Figure 1. ^{1}H and ^{13}C NMR spectra for 9-2.

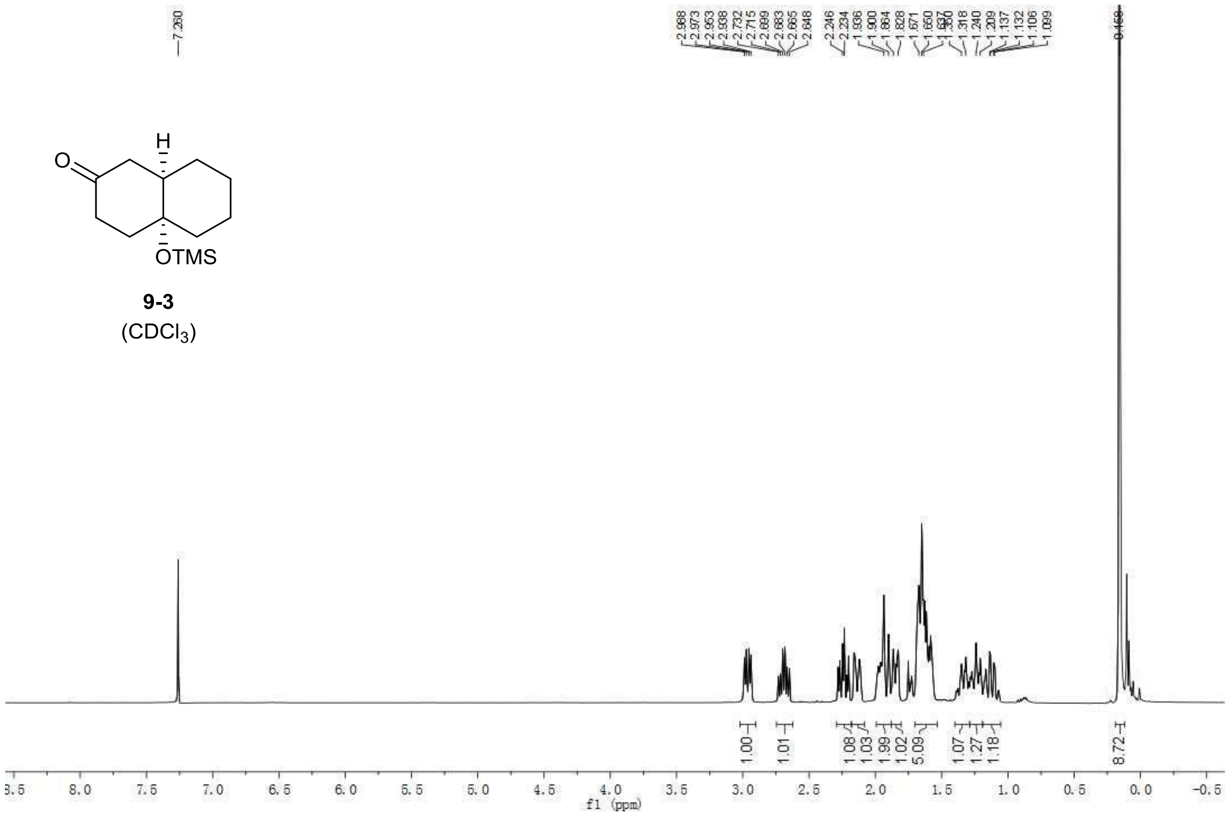

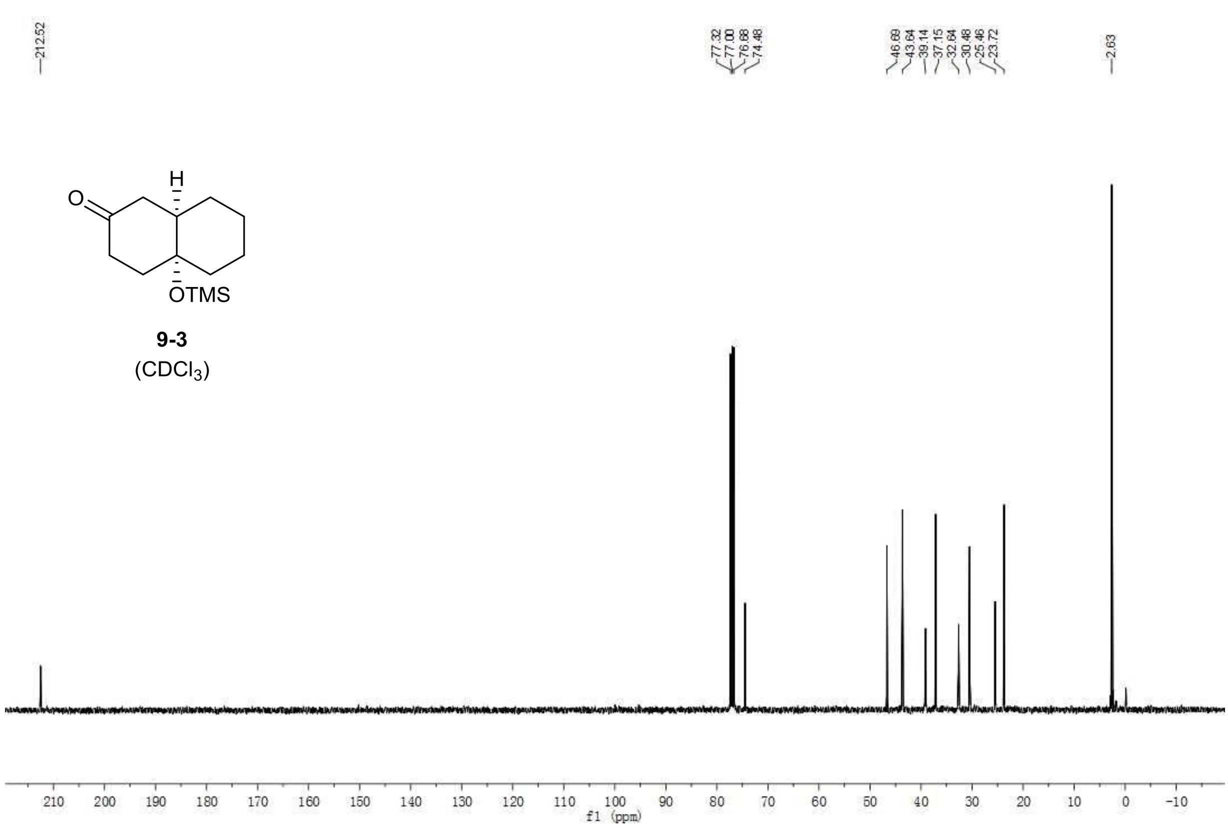

Supplementary Figure 2. ^{1}H and ^{13}C NMR spectra for 9-3.

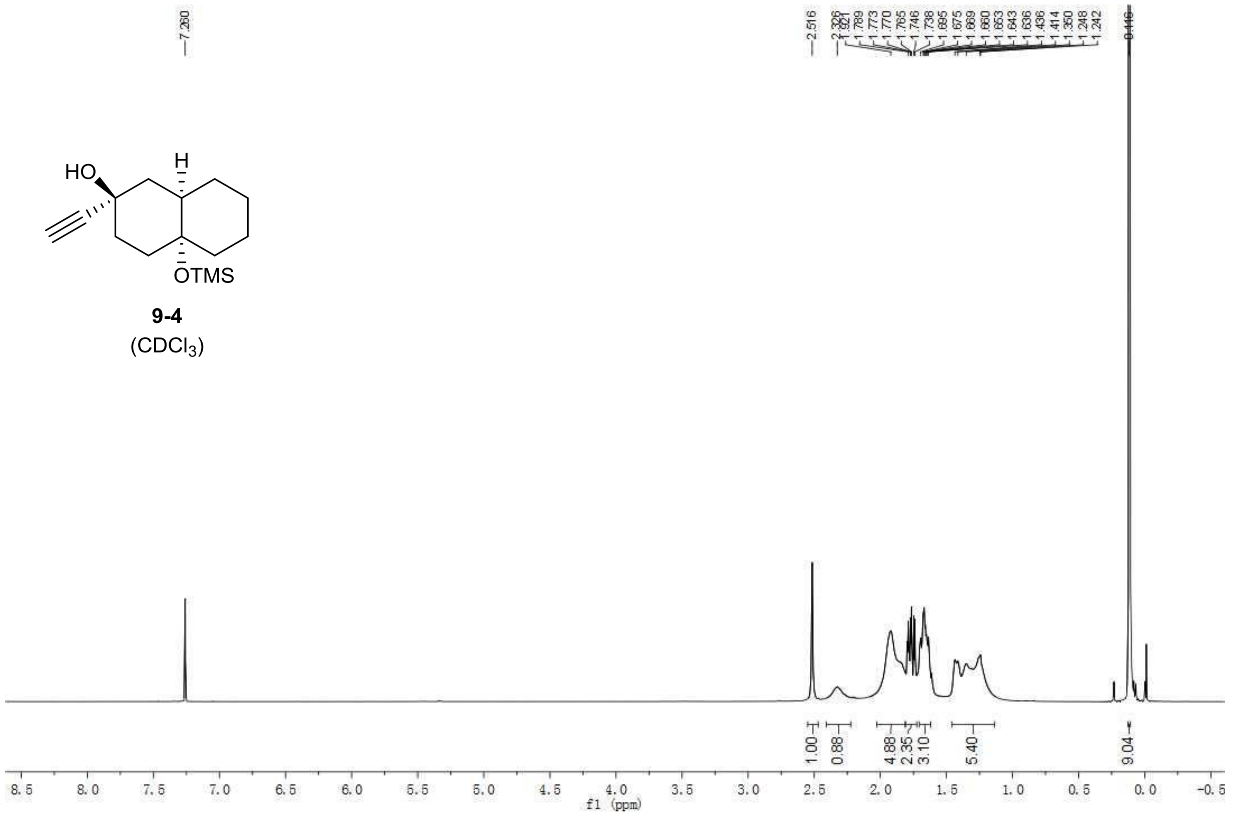

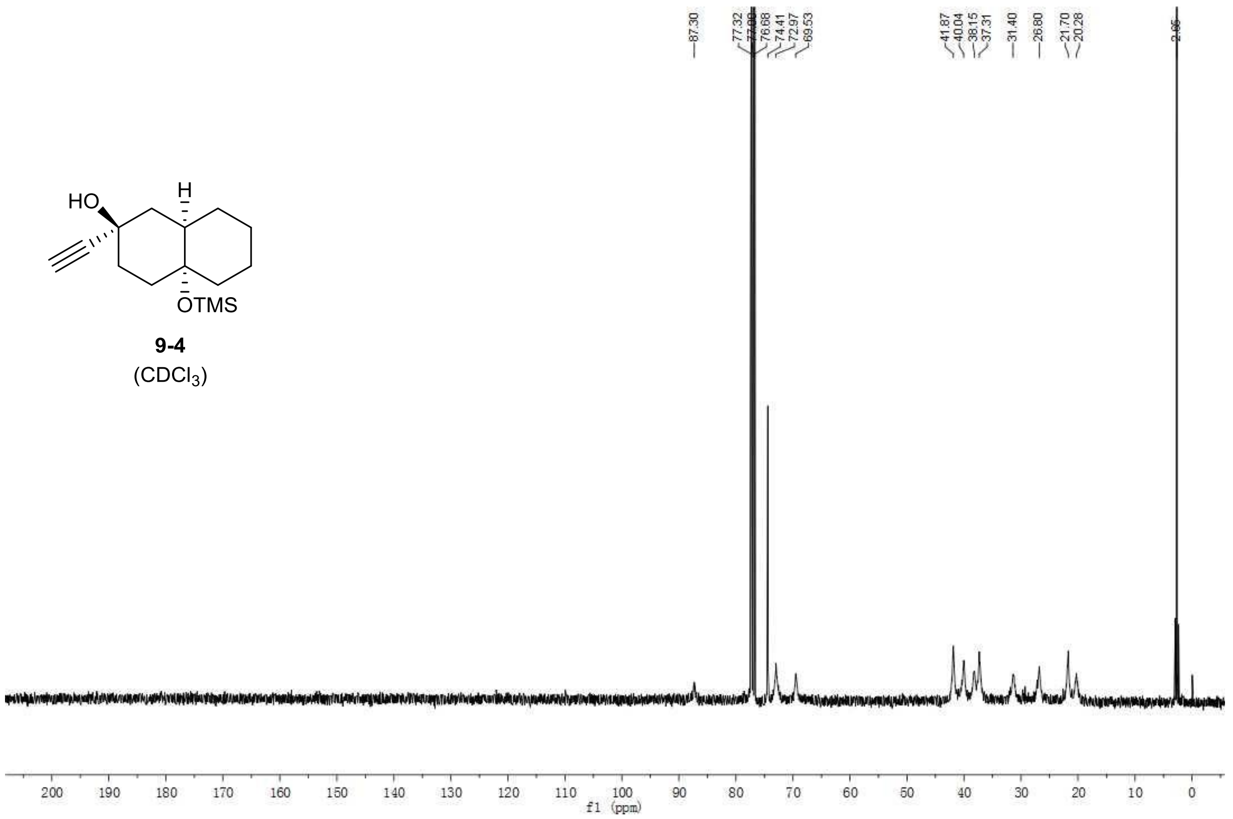

Supplementary Figure 3. ^{1}H and ^{13}C NMR spectra for 9-4.

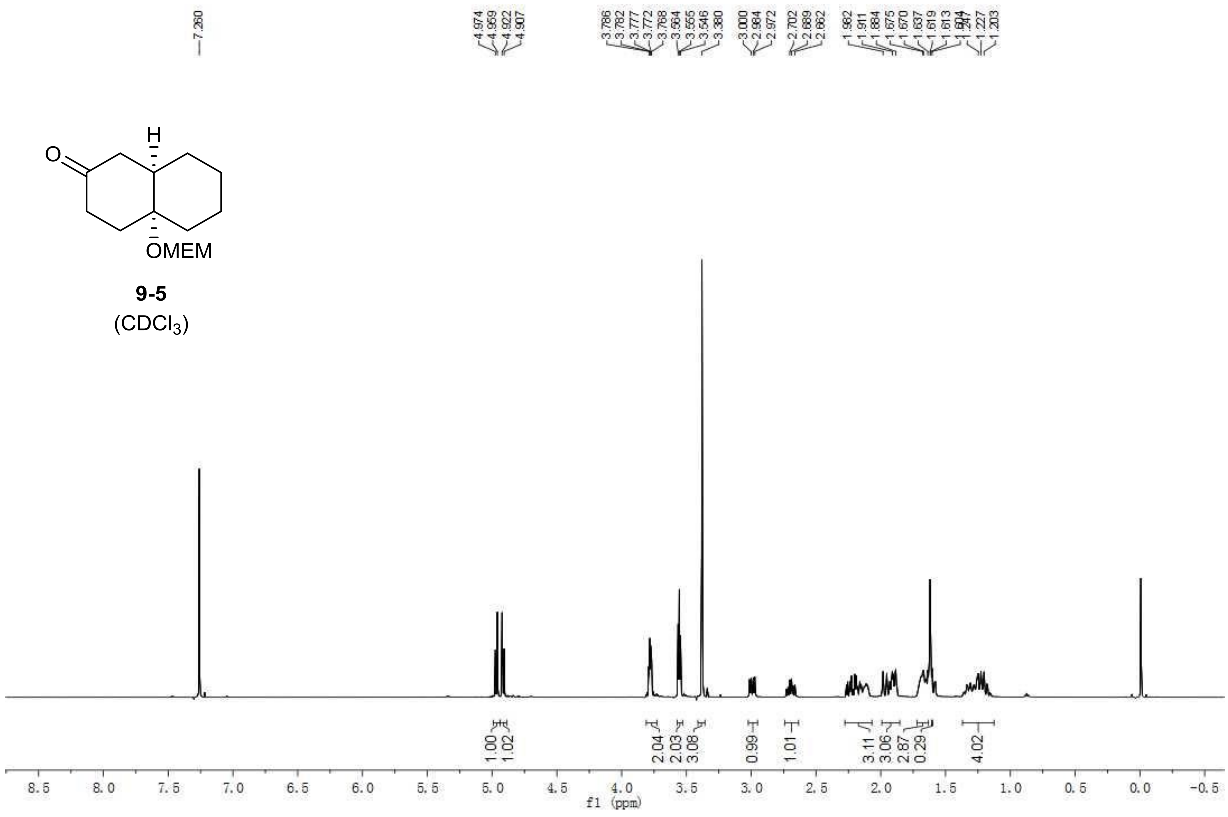

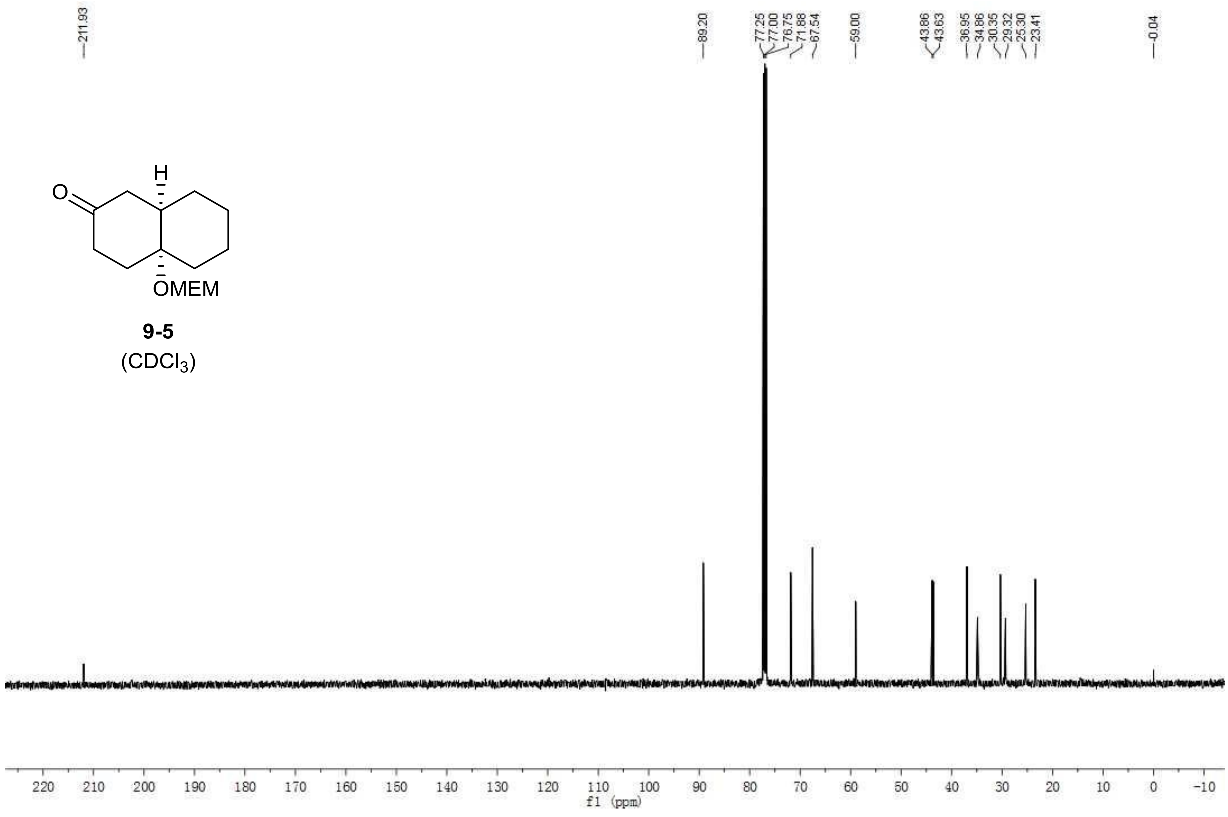

Supplementary Figure 4. ^{1}H and ^{13}C NMR spectra for 9-5.

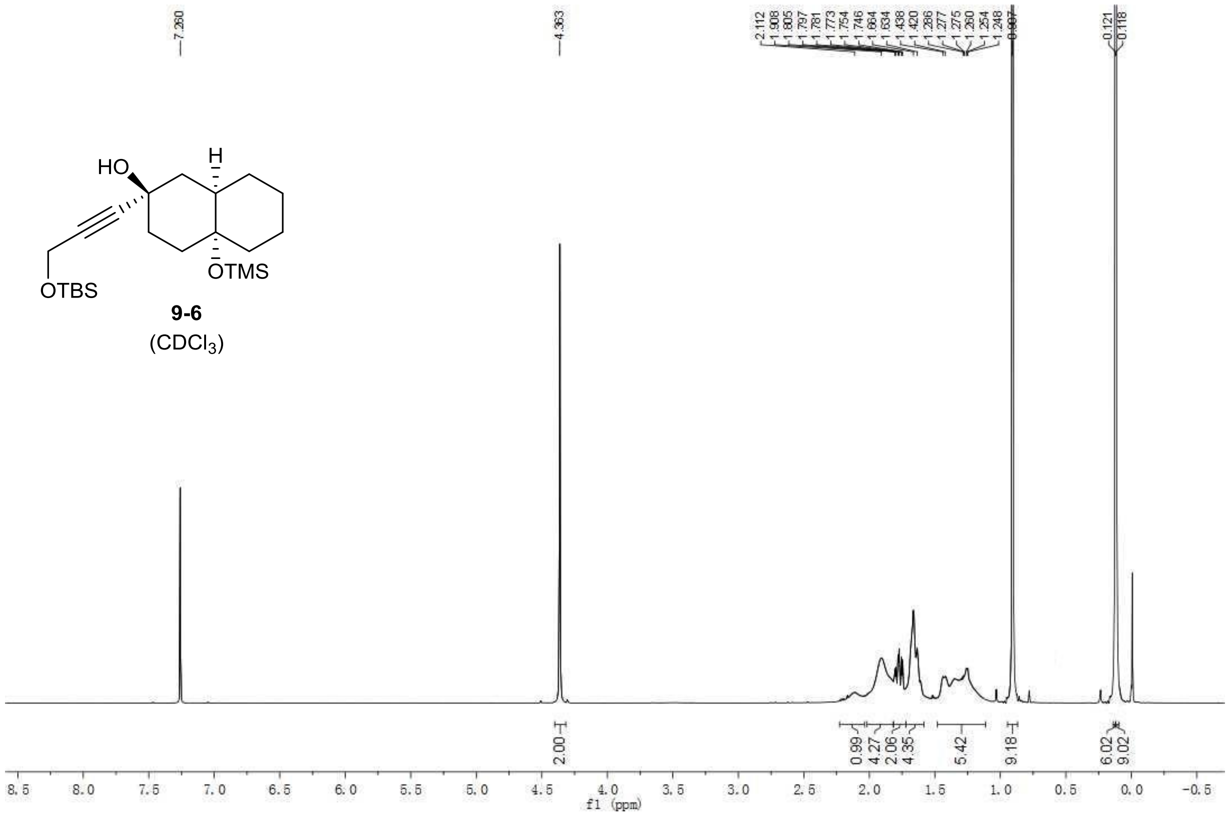

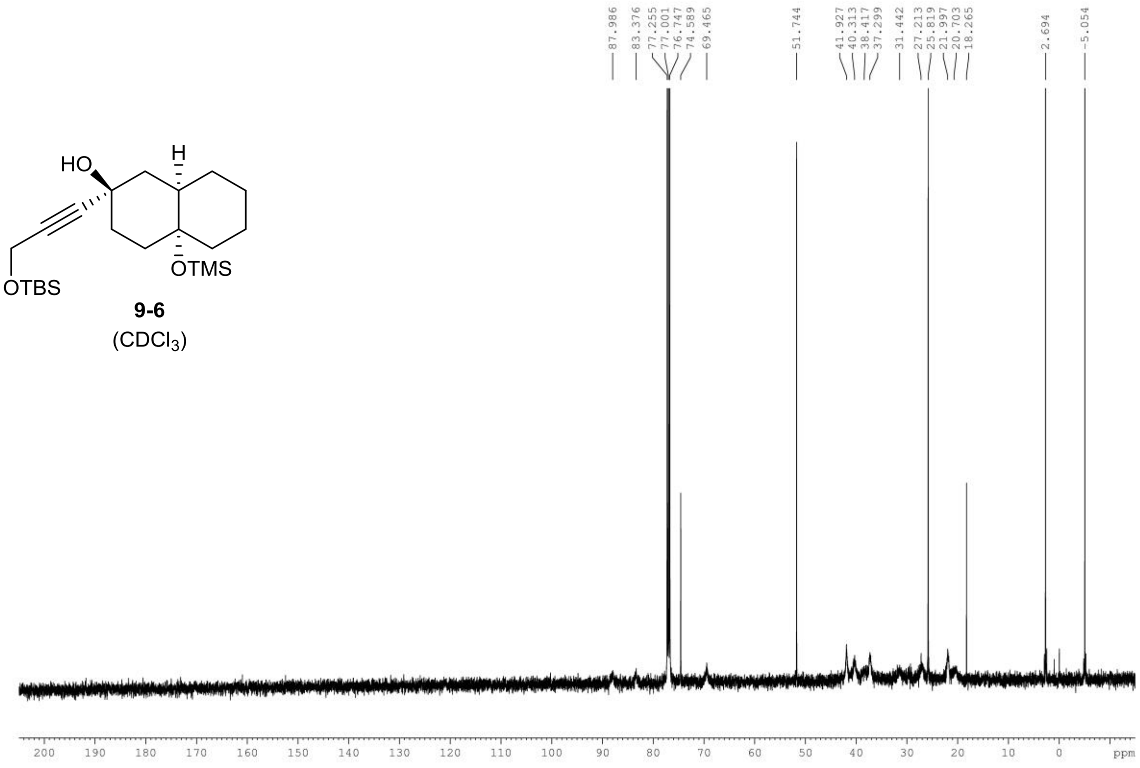

Supplementary Figure 5. ^{1}H and ^{13}C NMR spectra for 9-6.

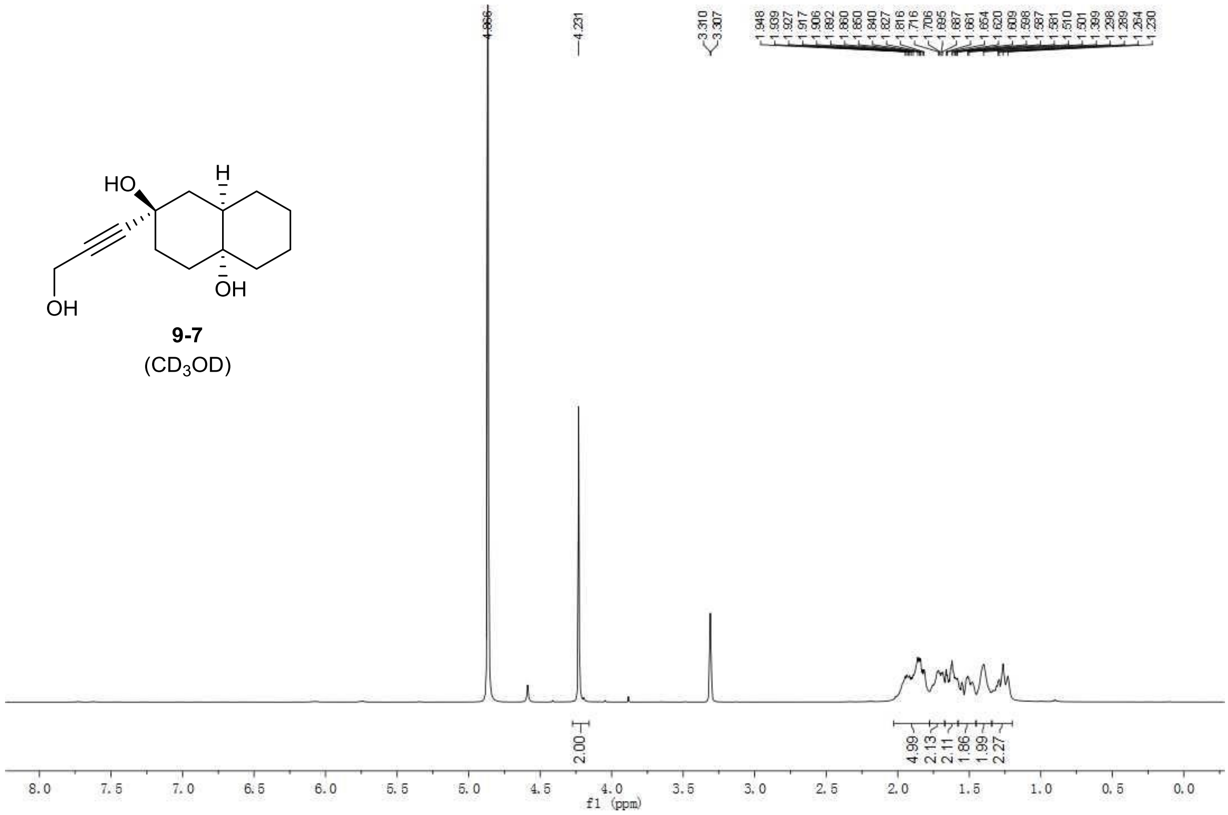

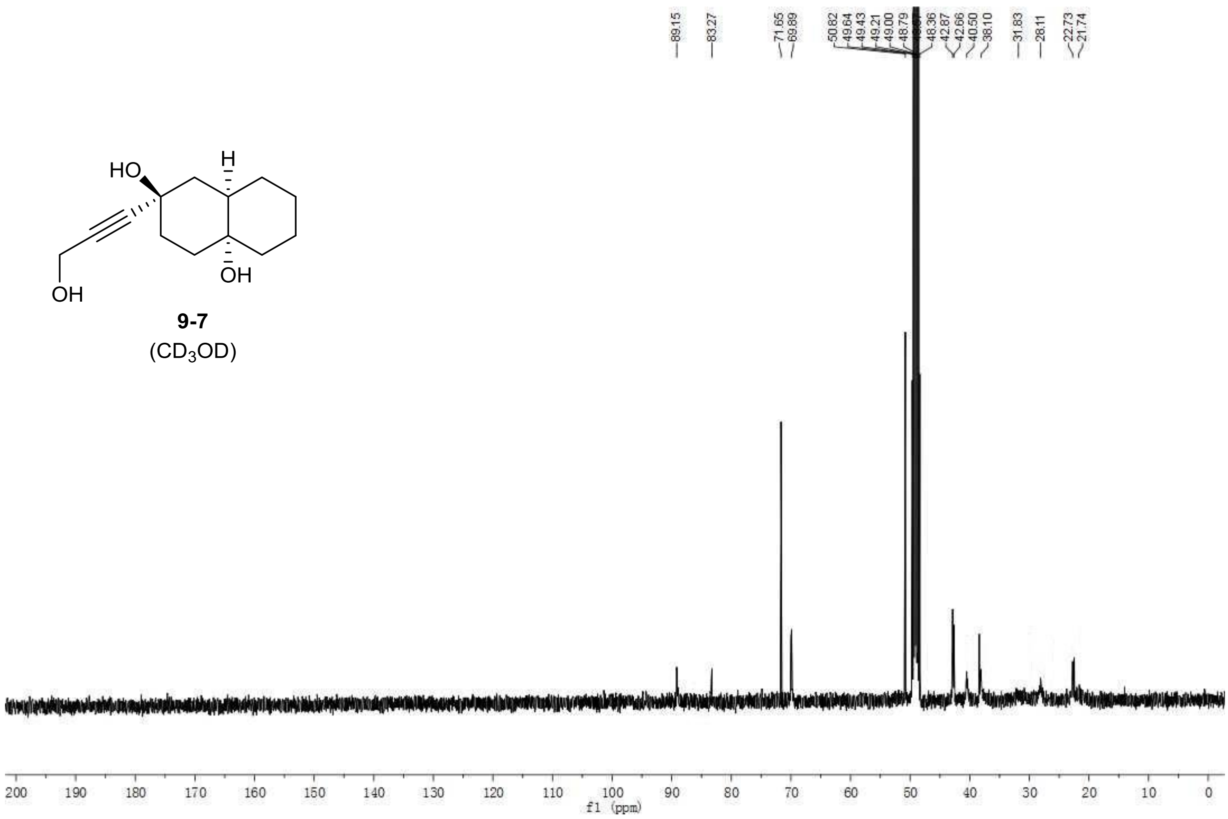

Supplementary Figure 6. ^{1}H and ^{13}C NMR spectra for 9-7.

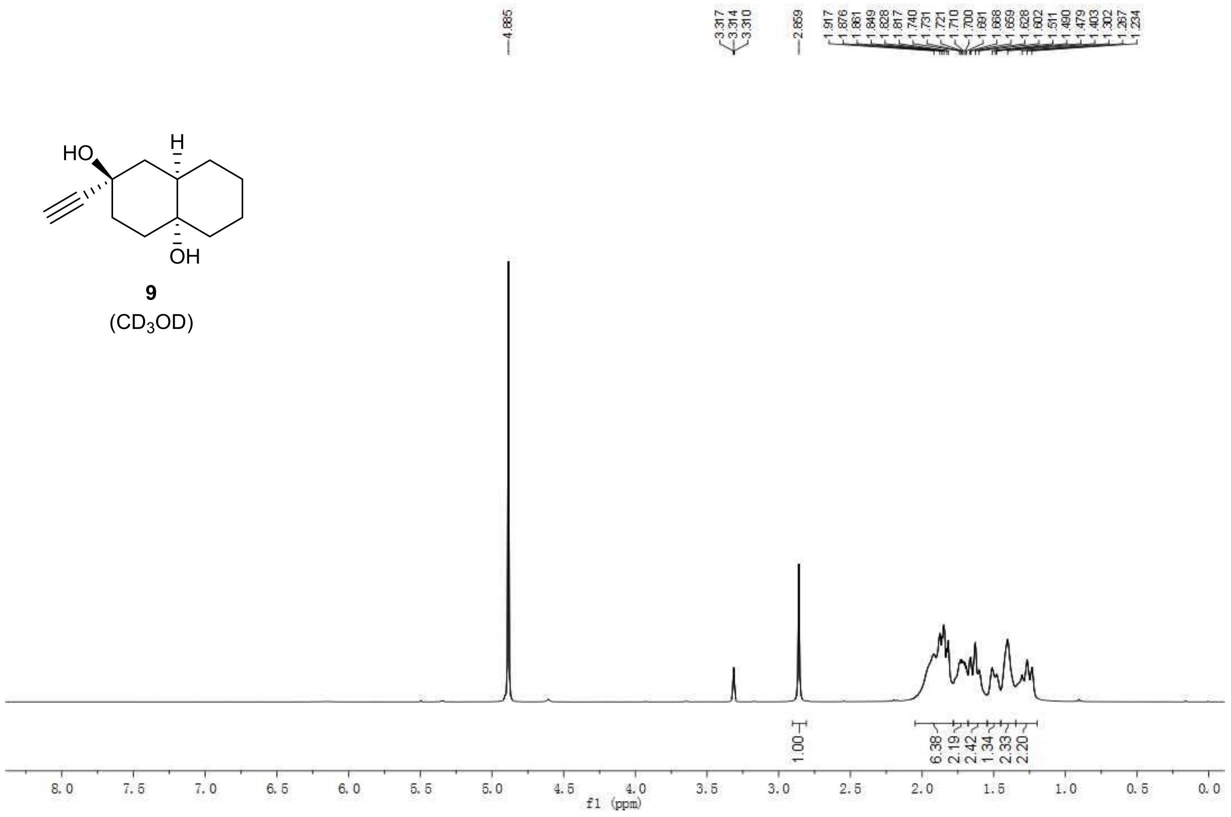

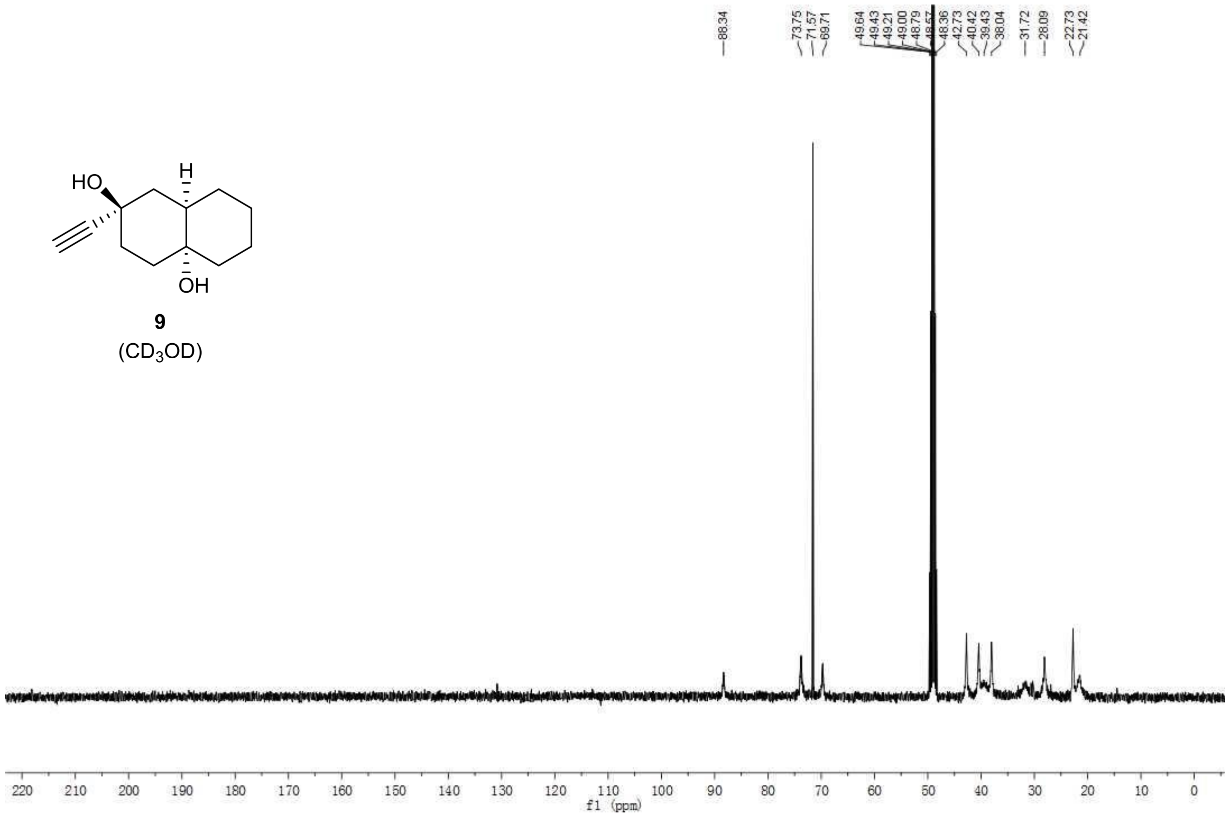

Supplementary Figure 7. ^{1}H and ^{13}C NMR spectra for 9.

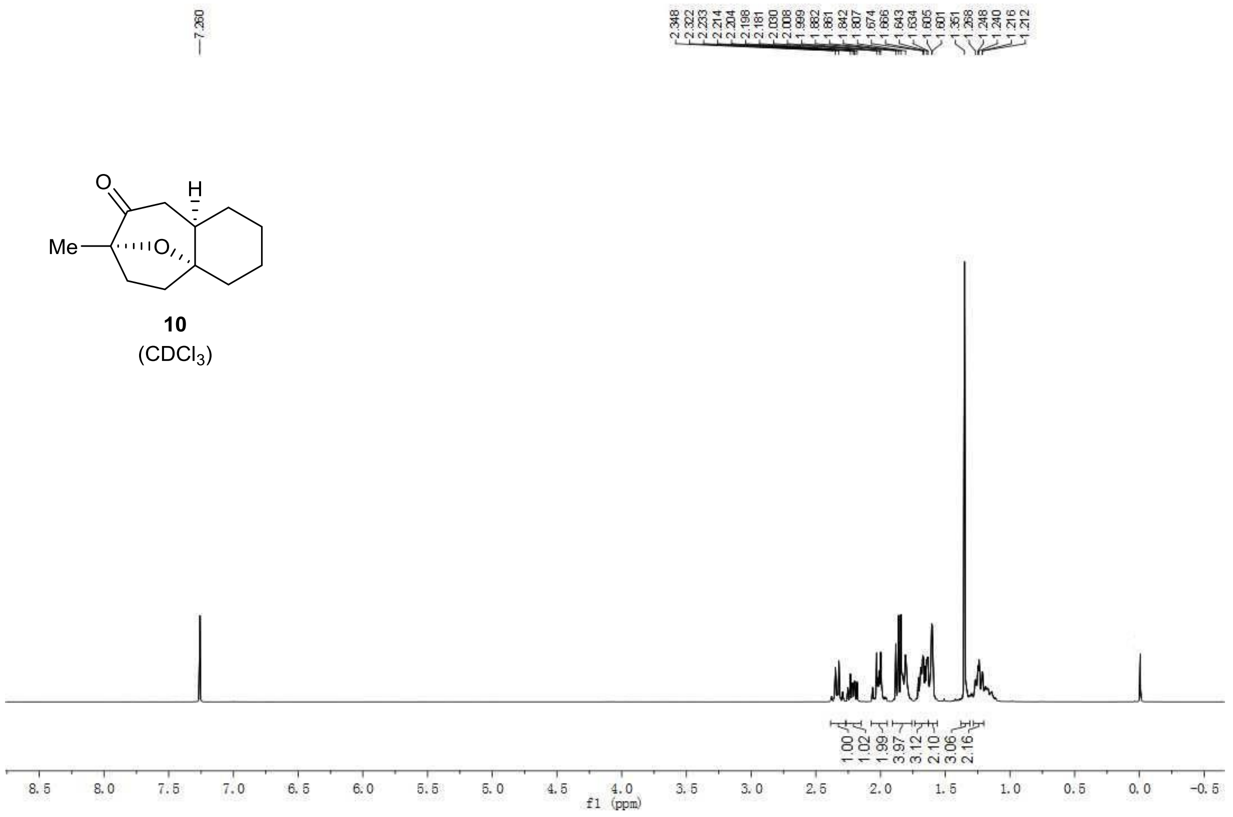

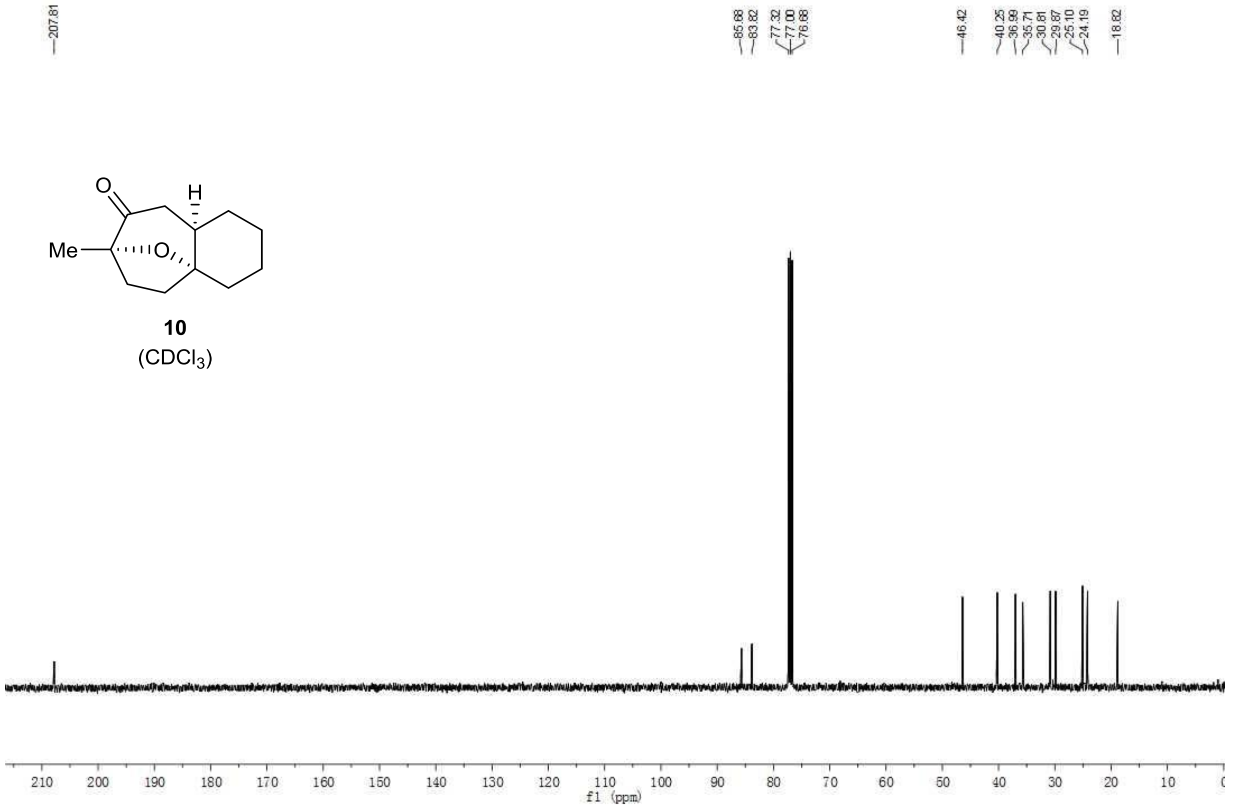

Supplementary Figure 8. ^{1}H and ^{13}C NMR spectra for 10.

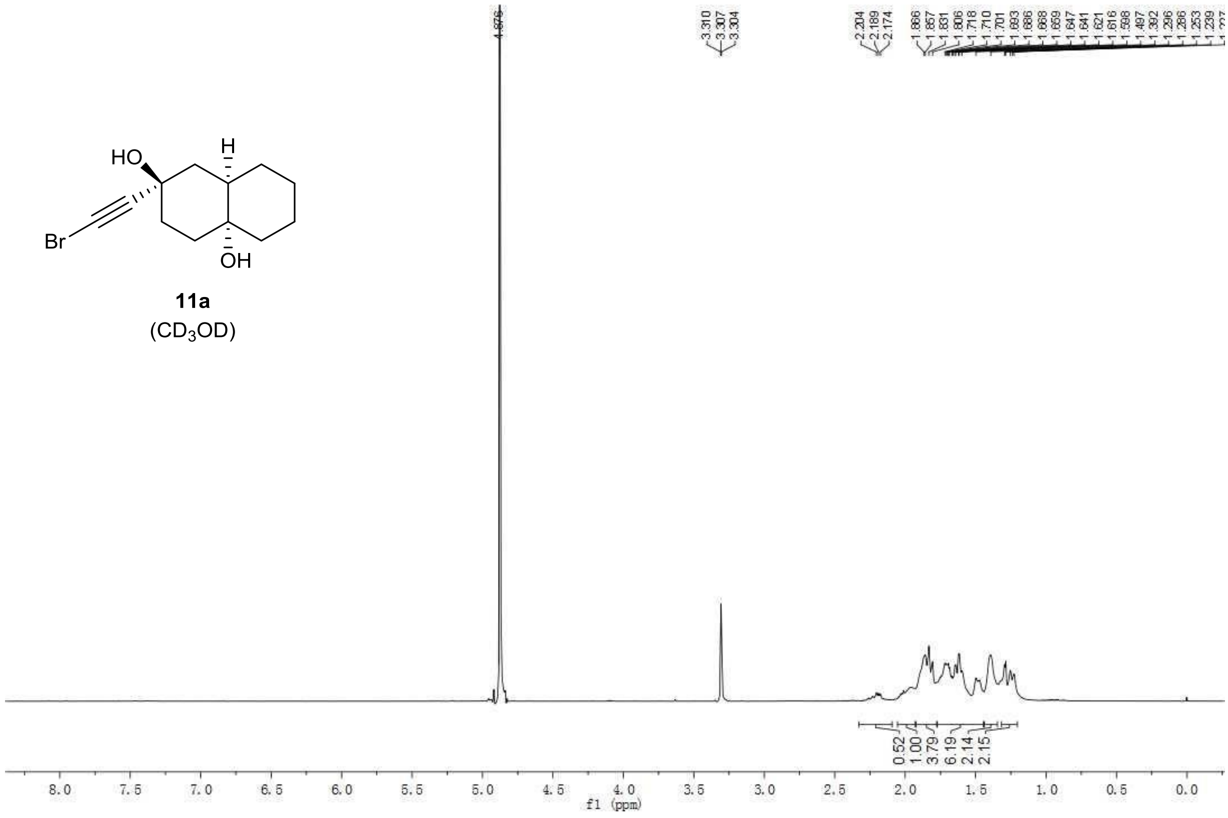

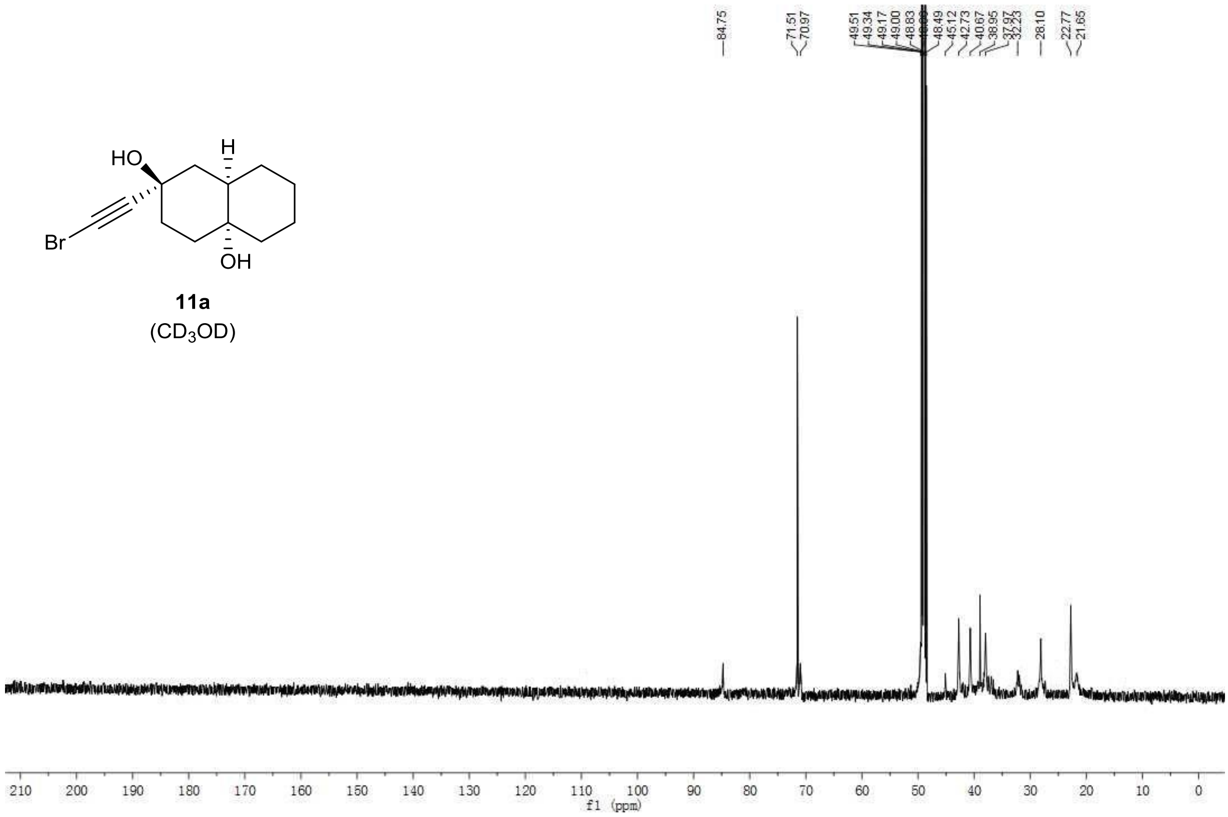

Supplementary Figure 9. ^{1}H and ^{13}C NMR spectra for 11a.

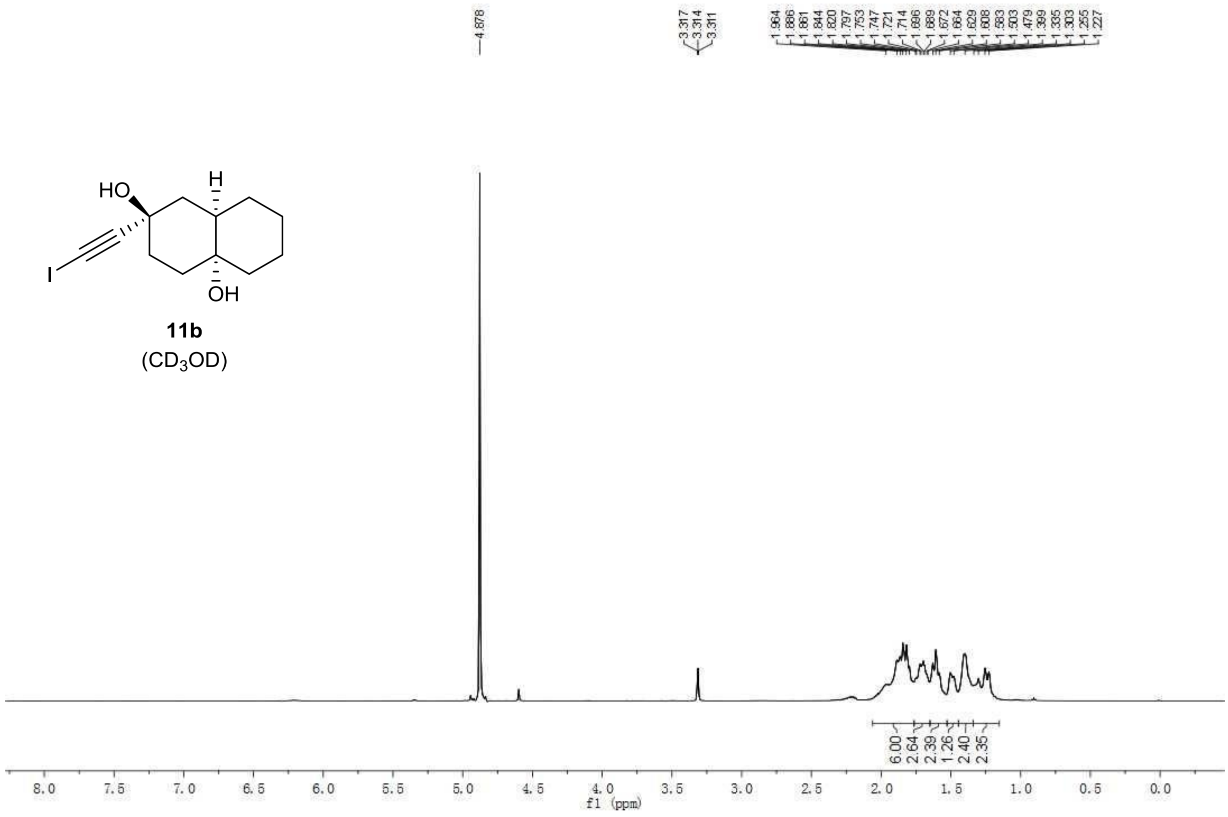

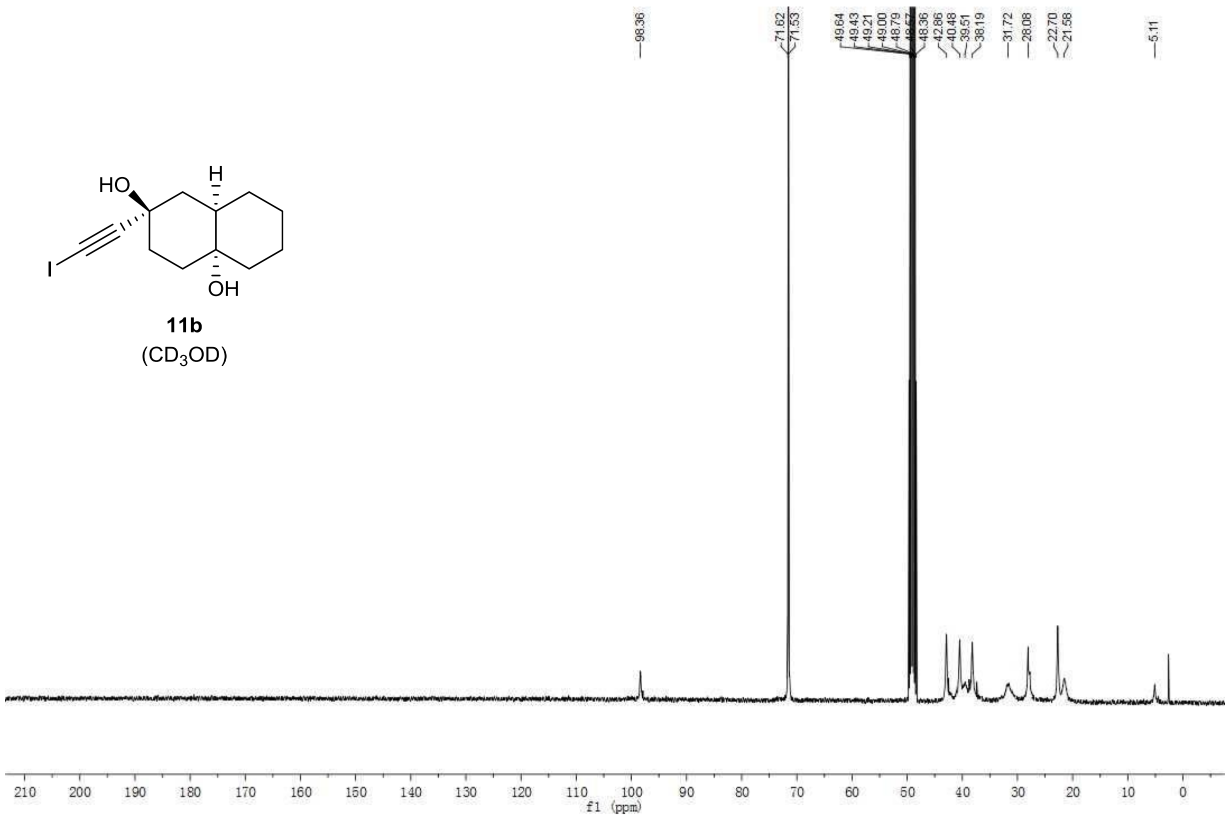

Supplementary Figure 10. ^{1}H and ^{13}C NMR spectra for 11b.

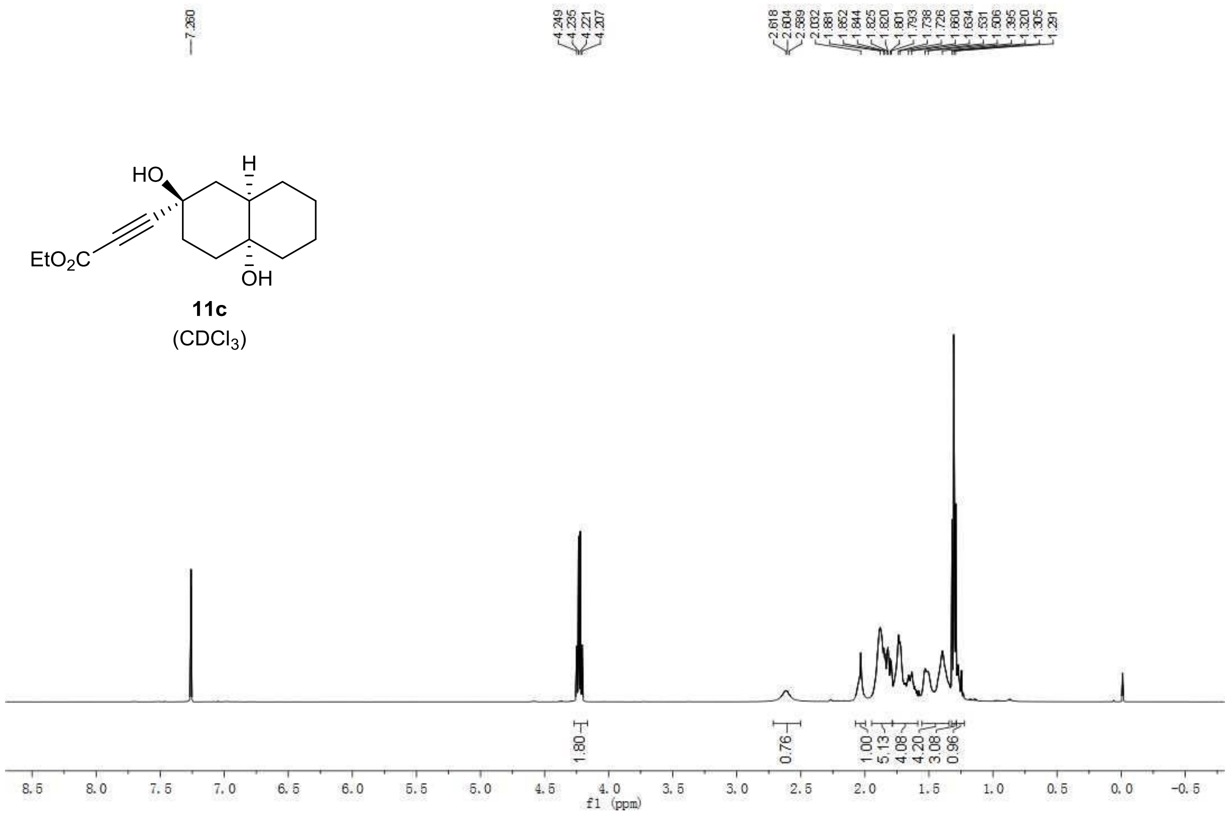

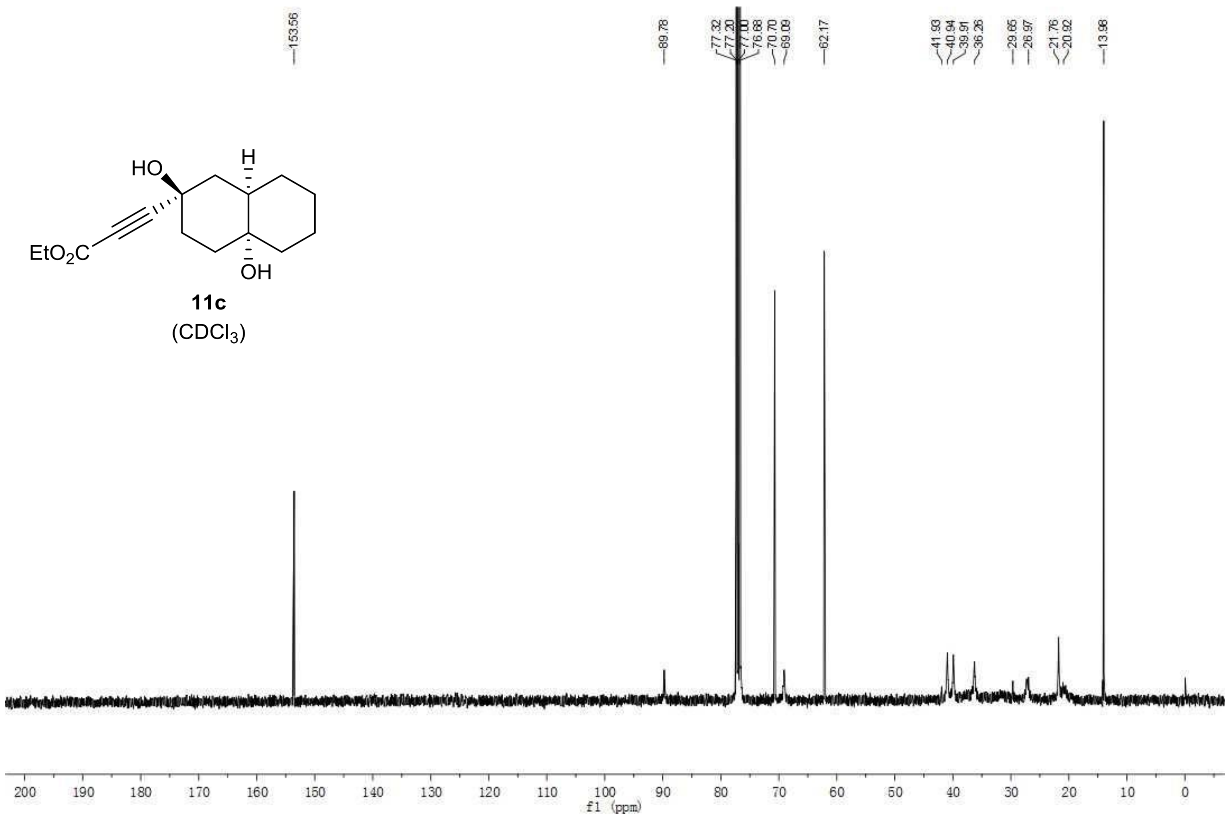

Supplementary Figure 11. ^{1}H and ^{13}C NMR spectra for 11c.

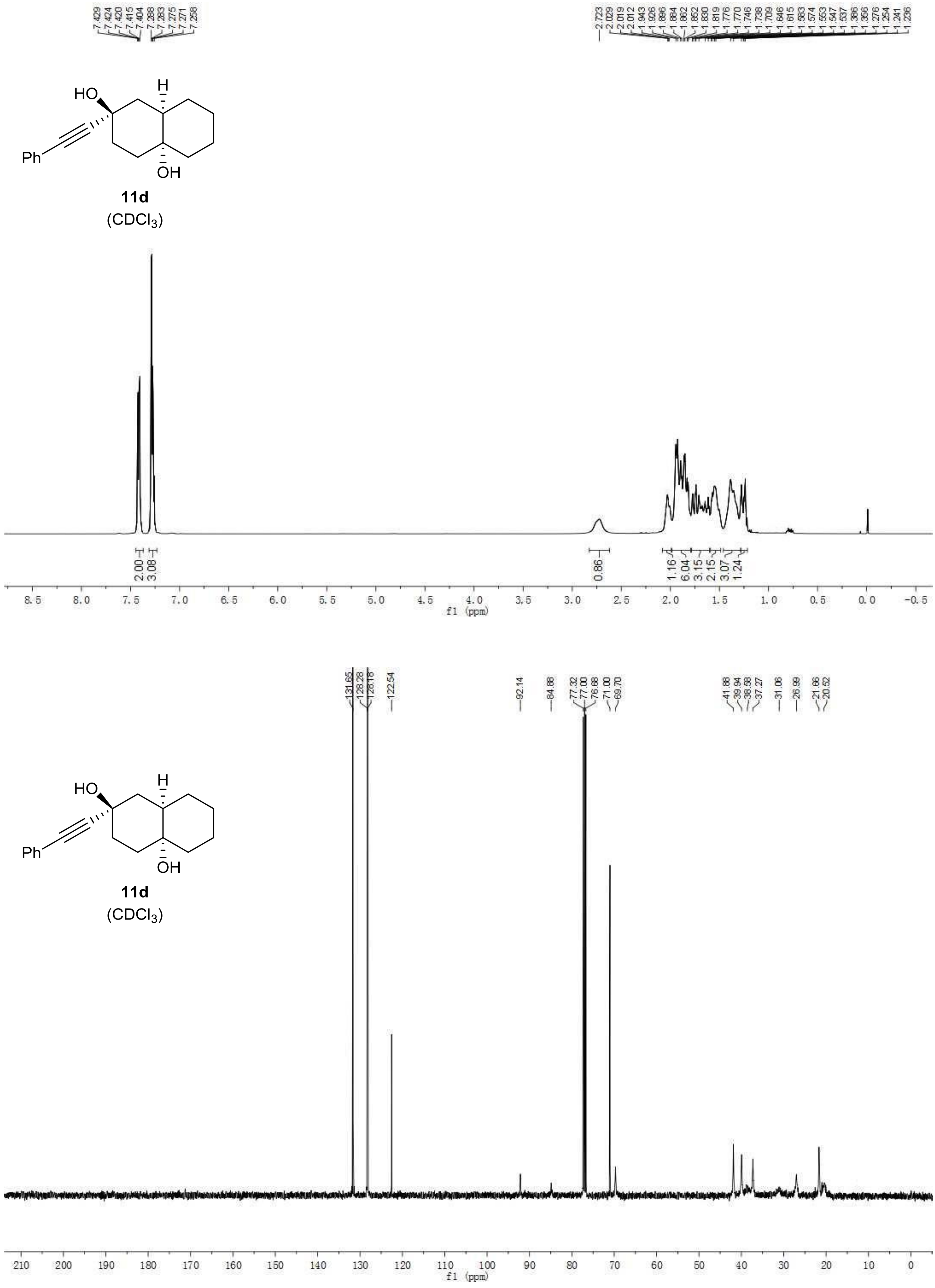

Supplementary Figure 12. ^{1}H and ^{13}C NMR spectra for 11d.

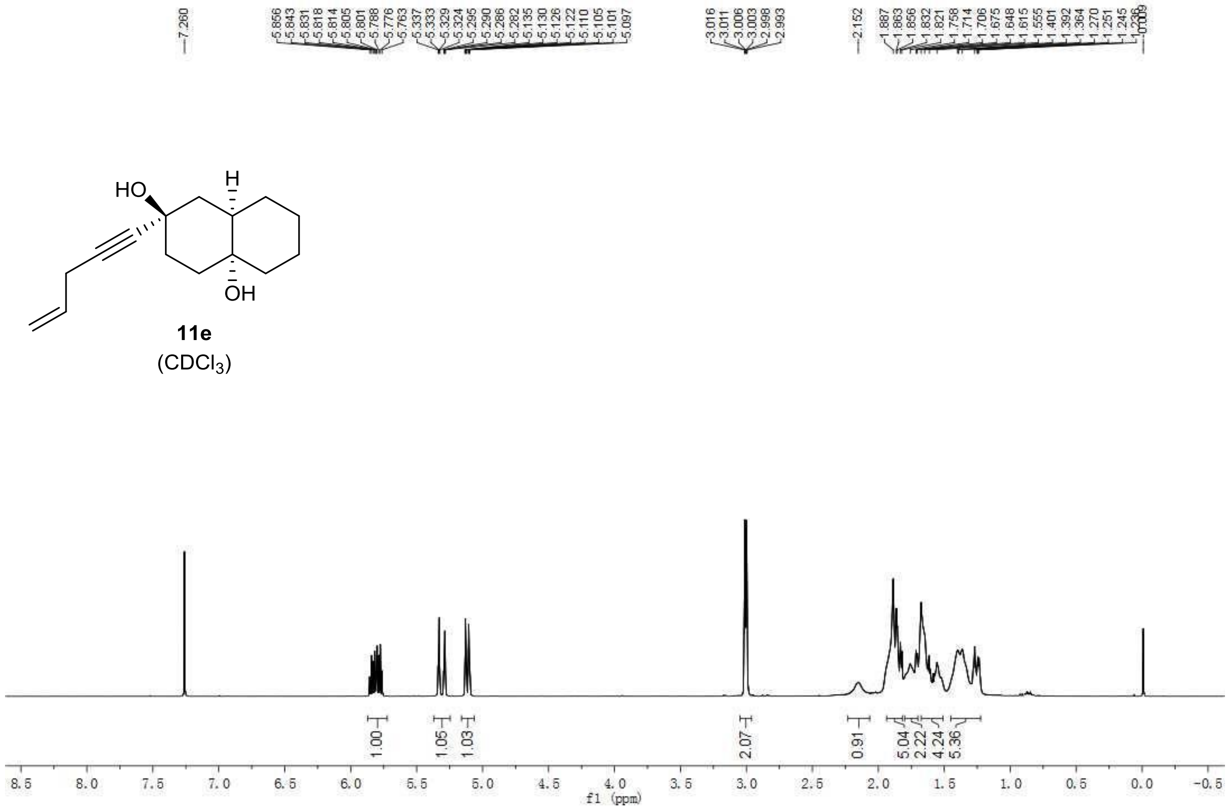

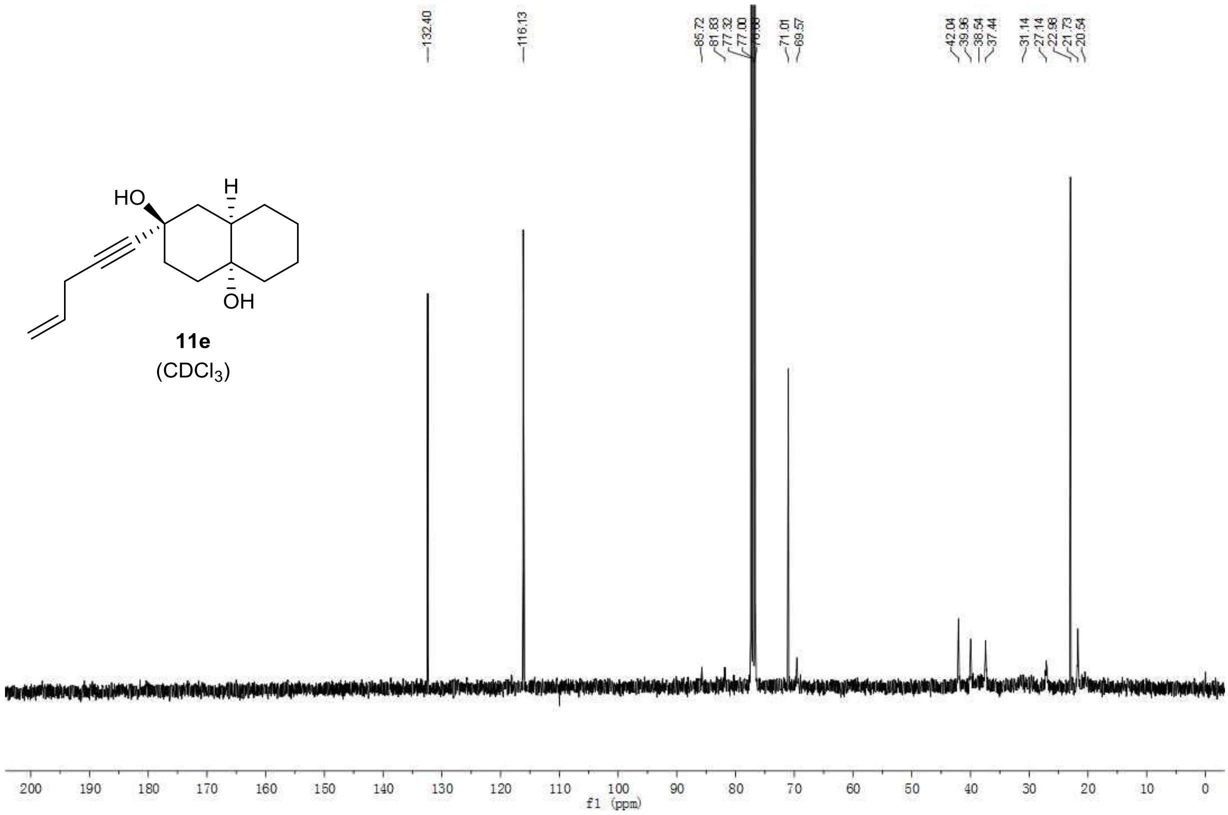

Supplementary Figure 13. ^{1}H and ^{13}C NMR spectra for 11e.

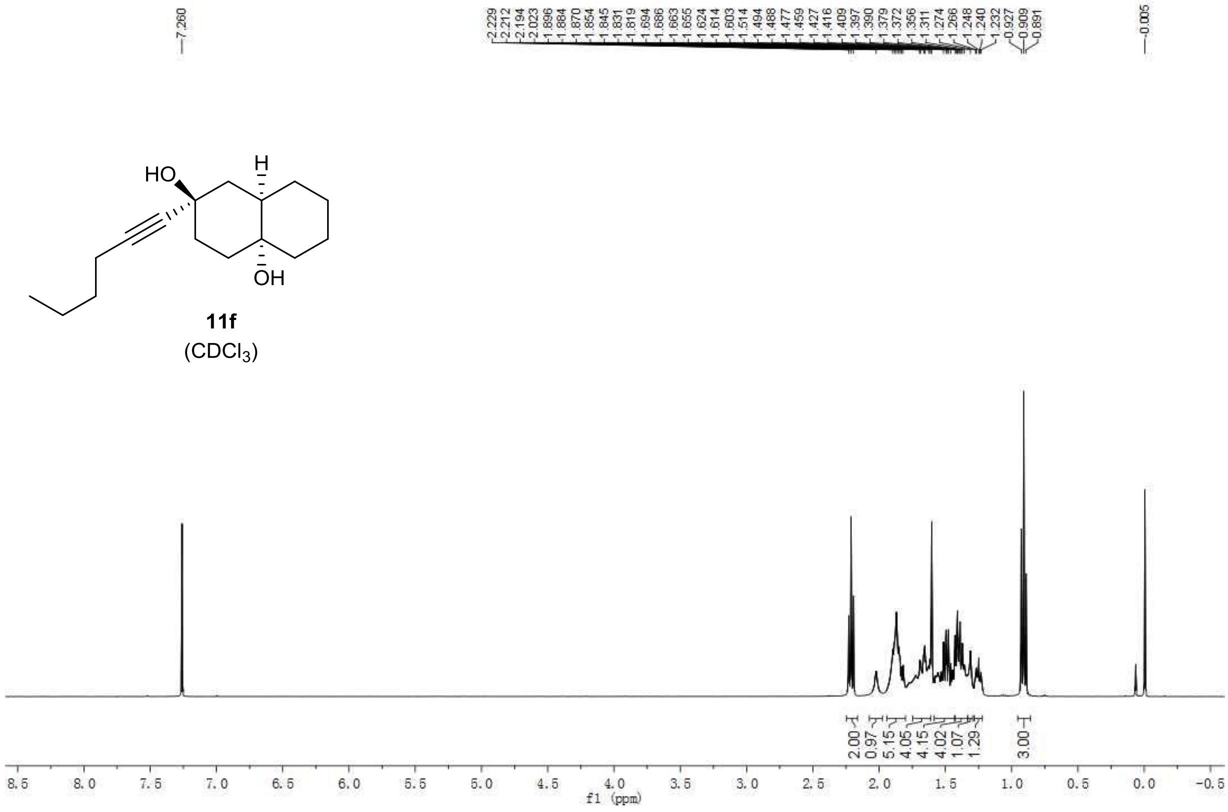

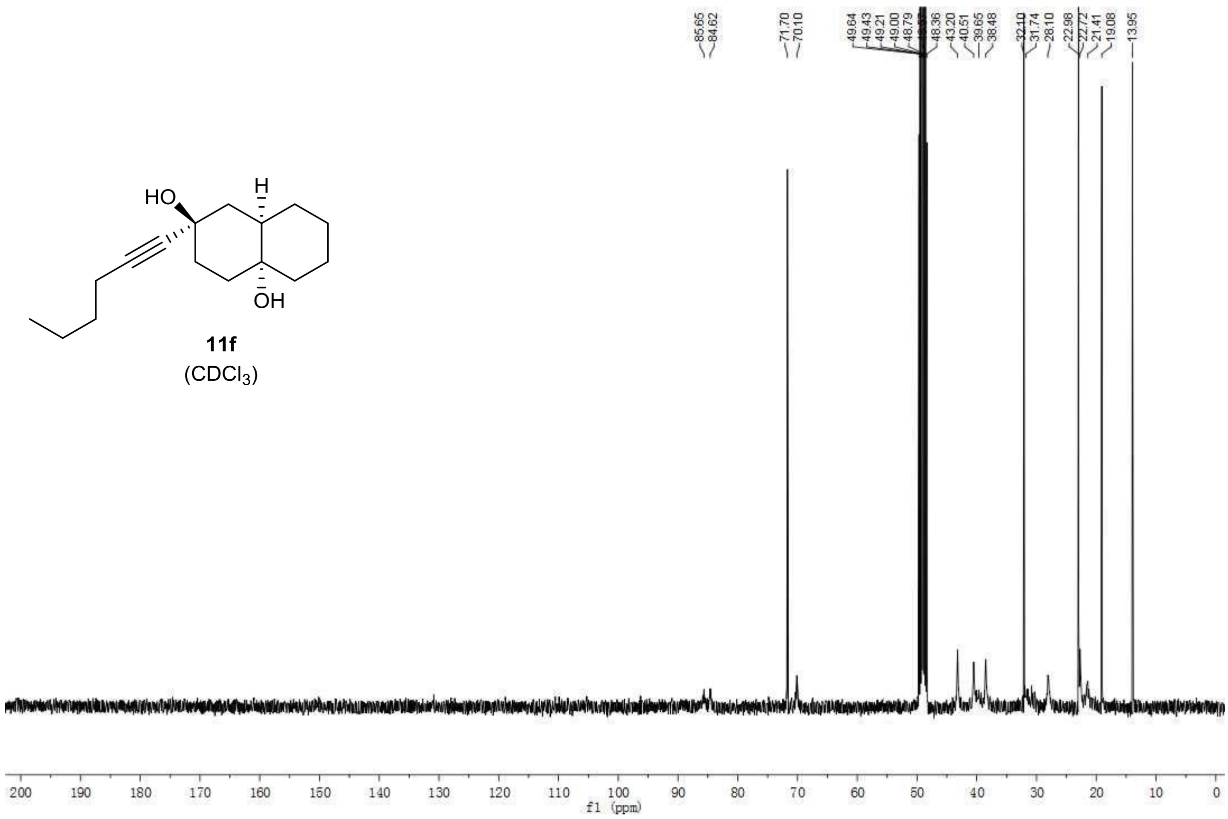

Supplementary Figure 14. ^{1}**H and** ^{13}**C NMR spectra for 11f.**

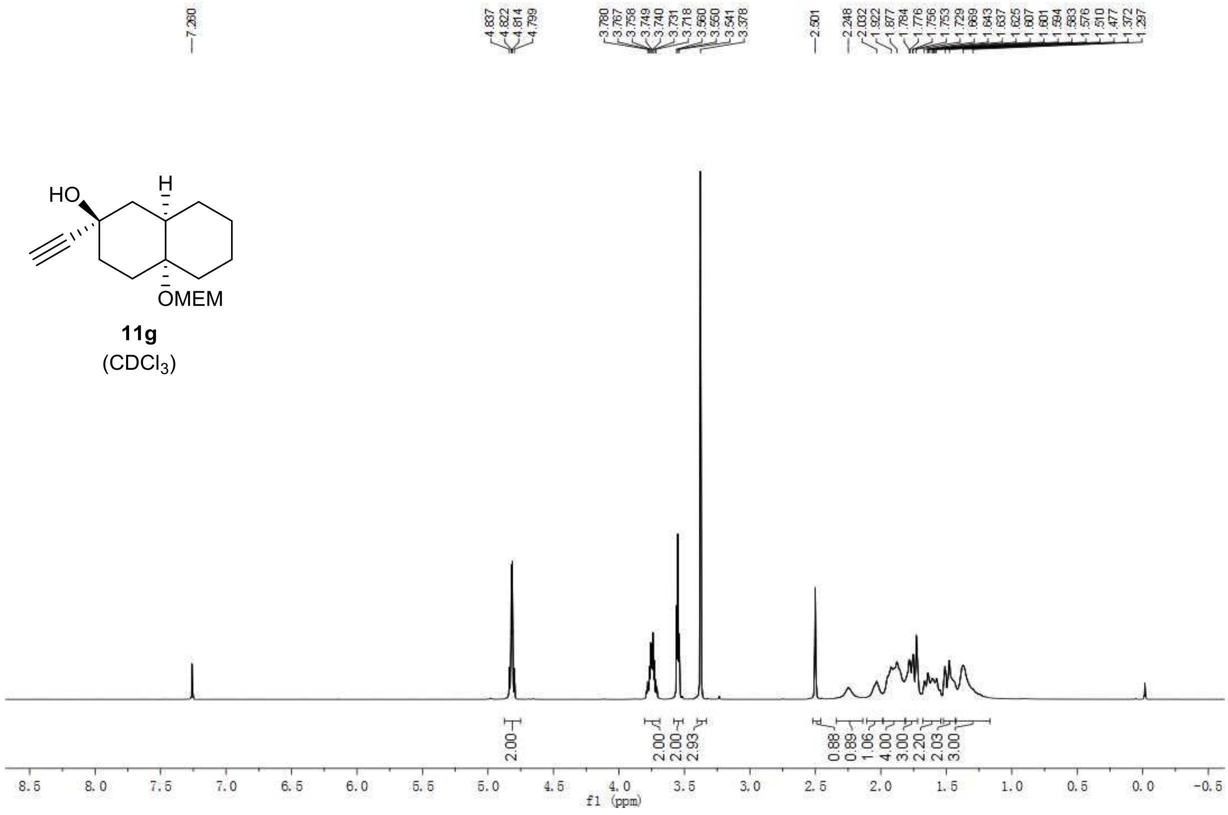

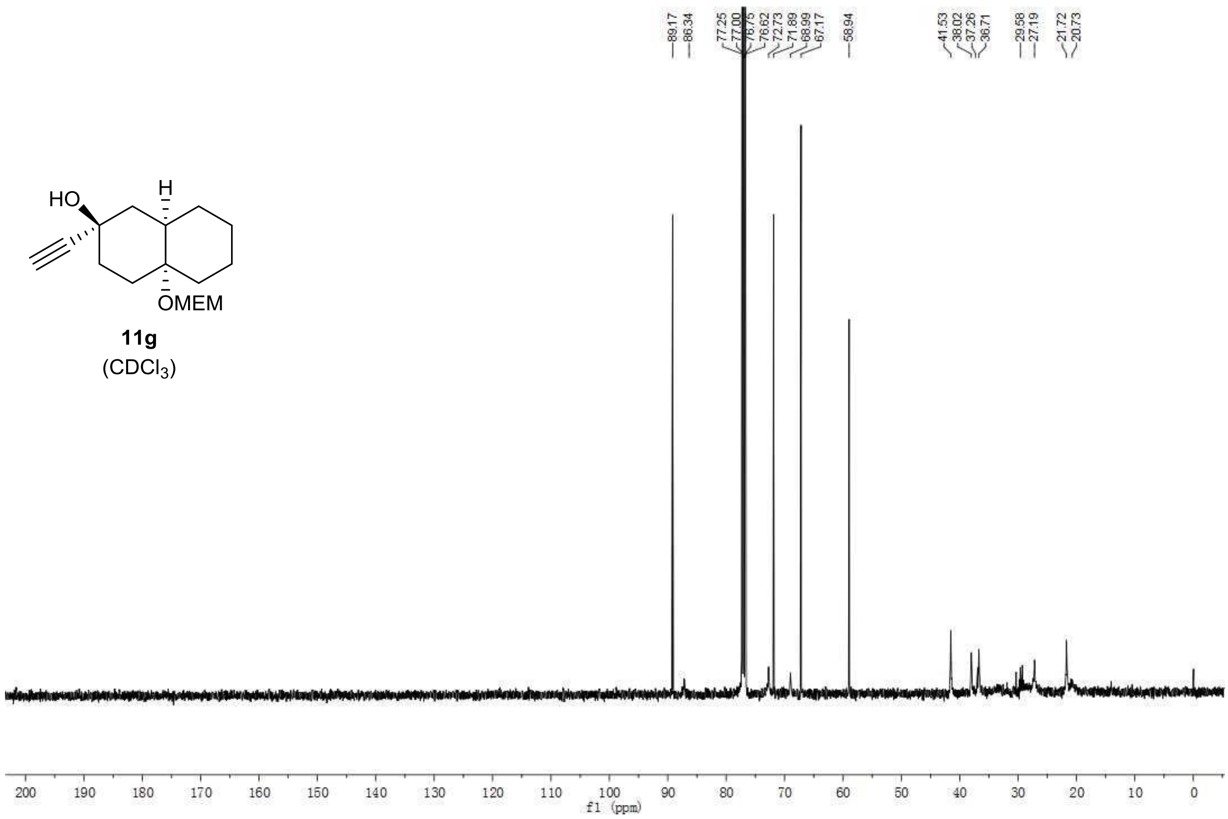

Supplementary Figure 15. ^{1}H and ^{13}C NMR spectra for 11g.

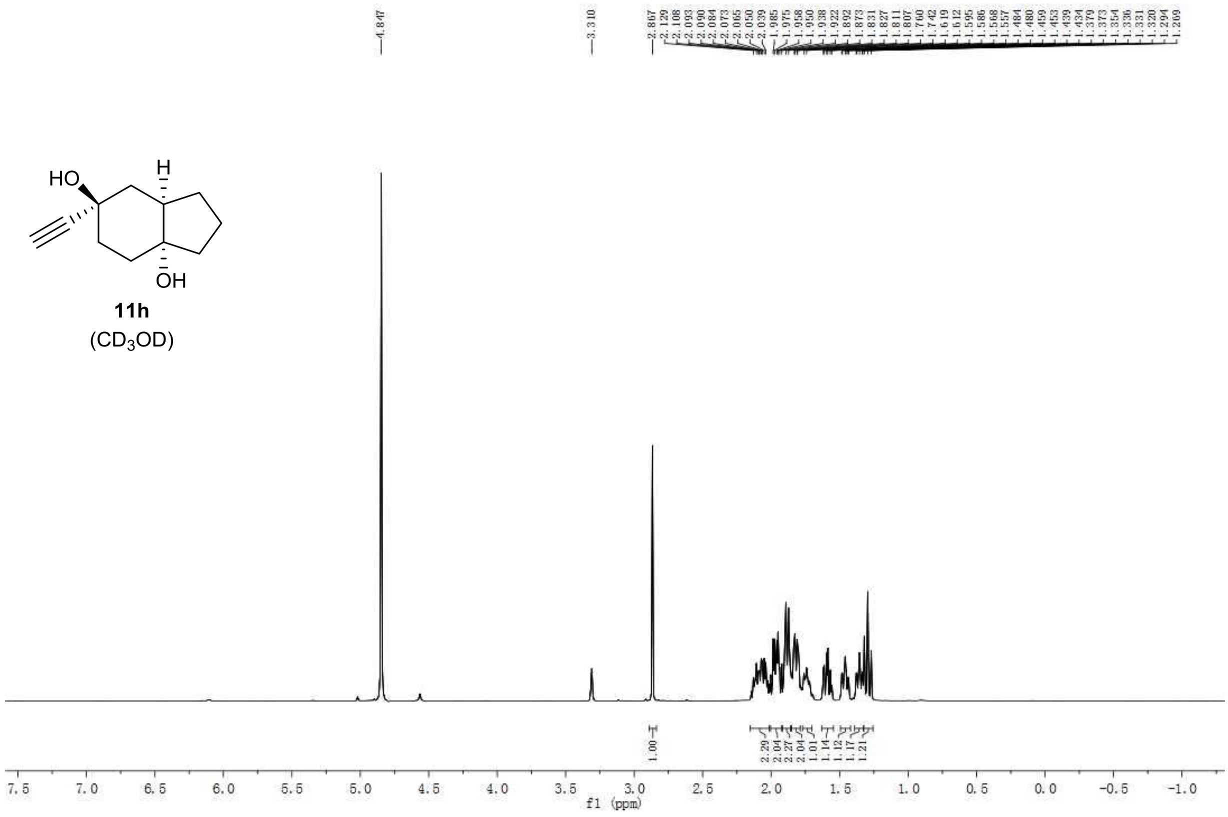

11h
(CD_3OD)

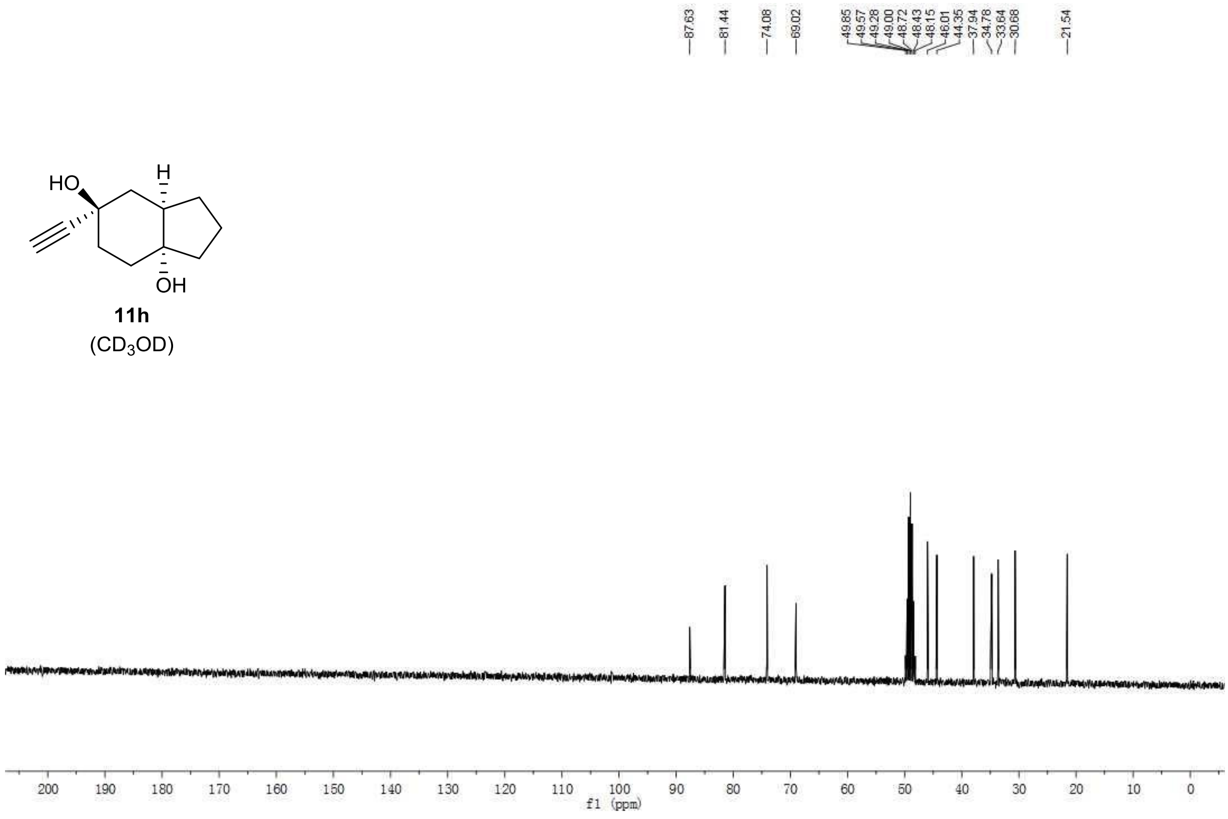

11h
(CD_3OD)

Supplementary Figure 16. ^{1}H and ^{13}C NMR spectra for 11h.

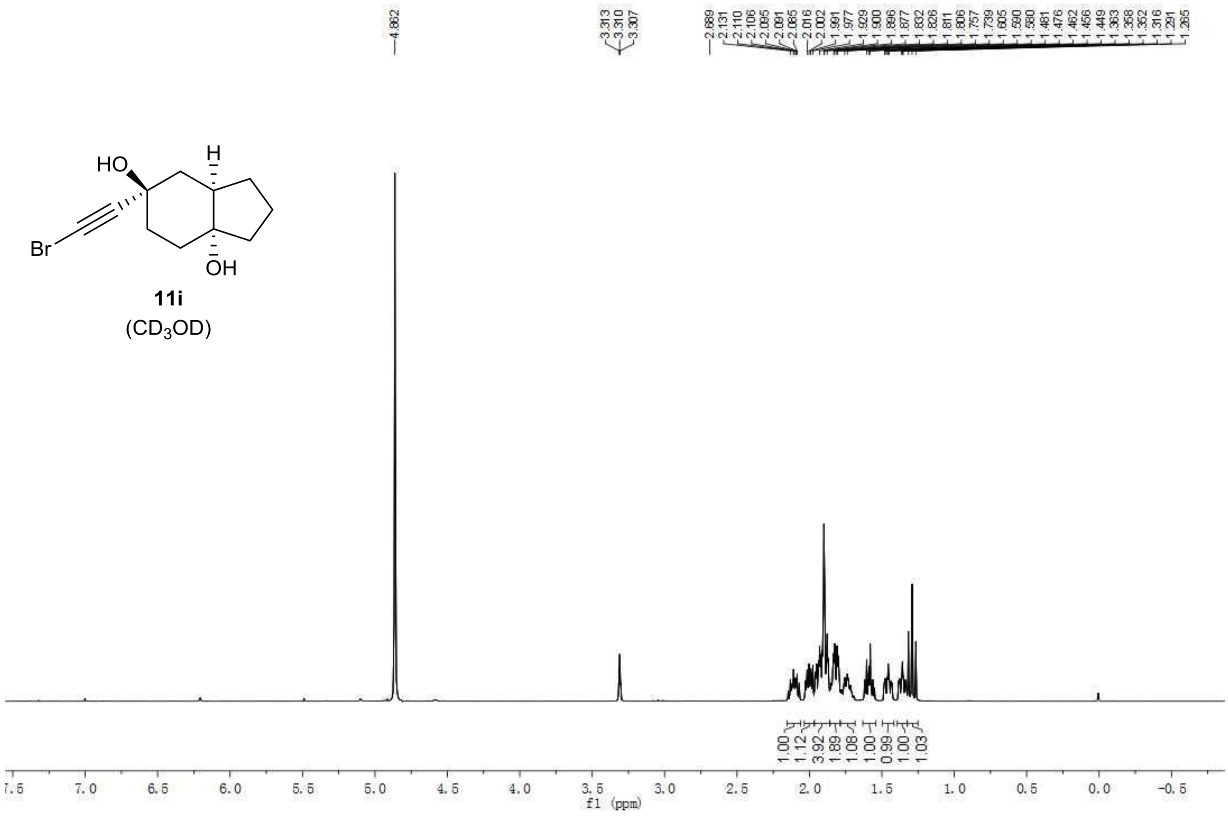

11i
(CD_3OD)

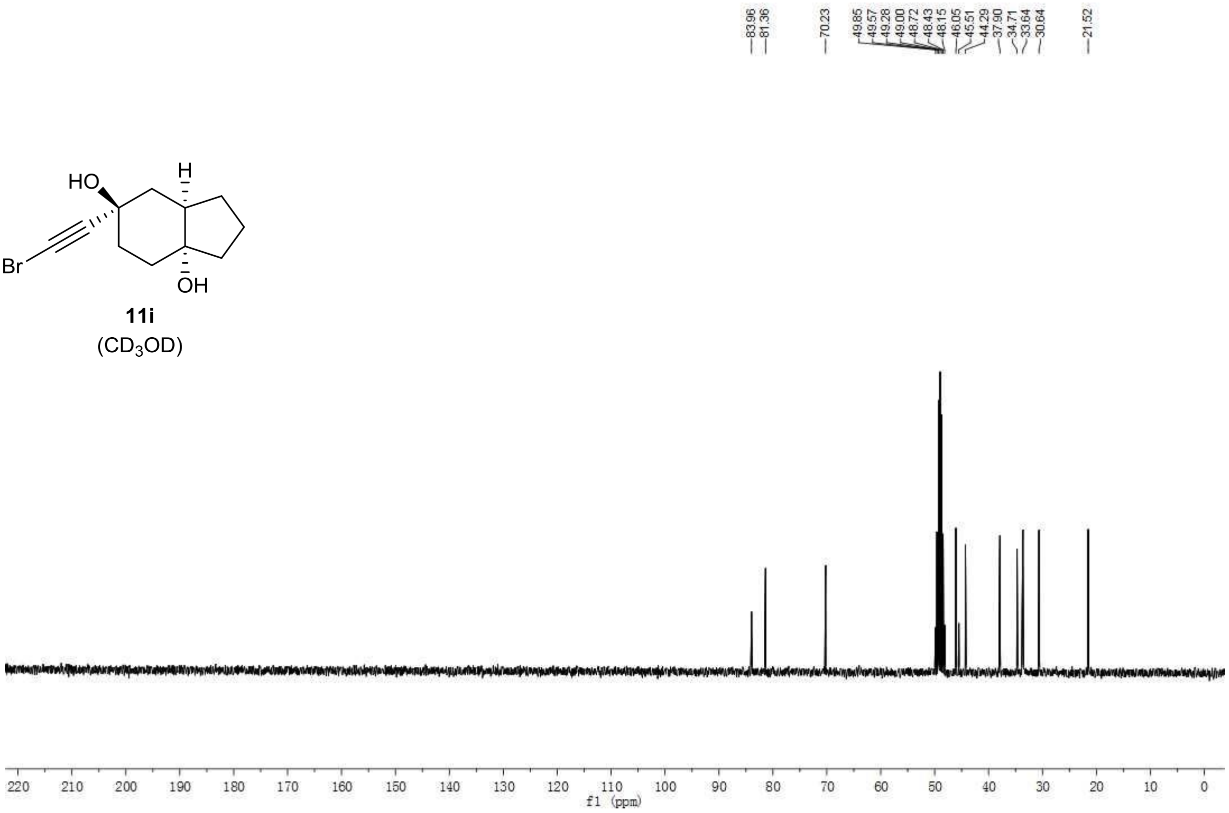

11i
(CD_3OD)

Supplementary Figure 17. ^{1}H and ^{13}C NMR spectra for 11i.

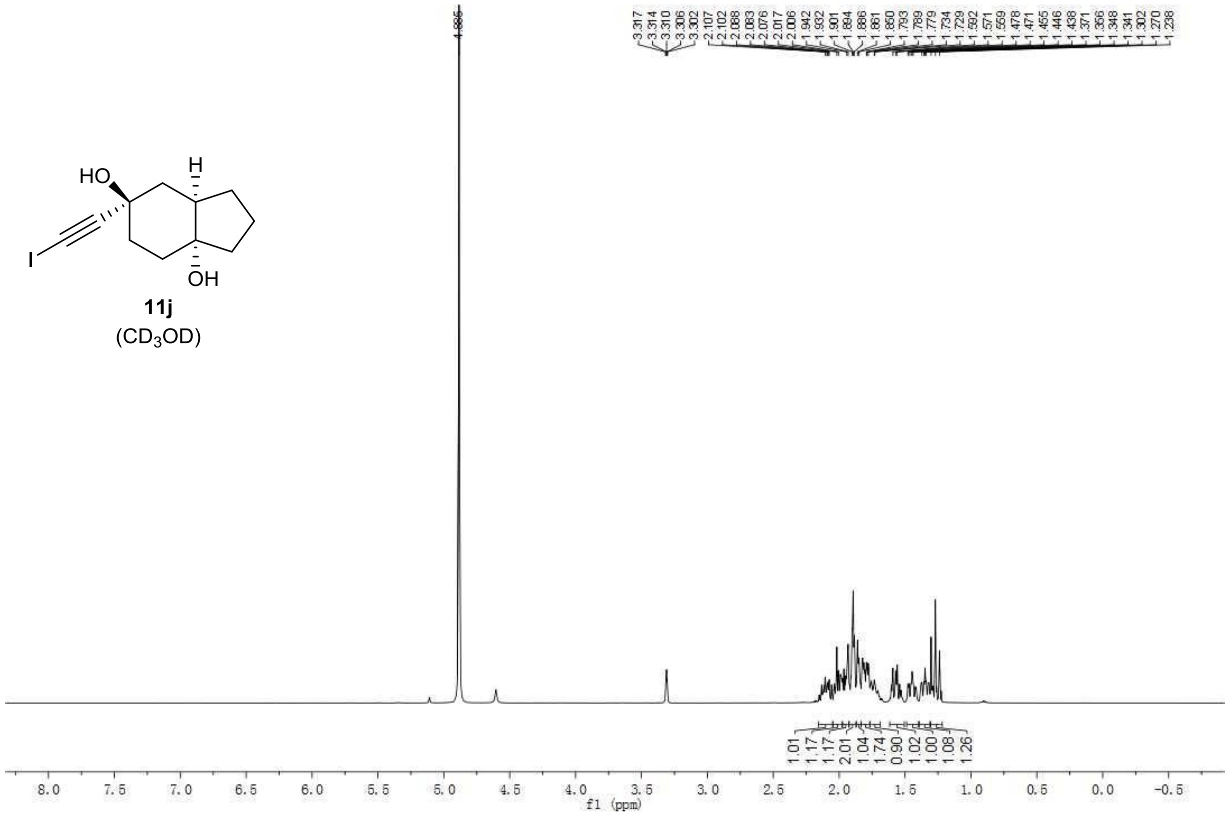

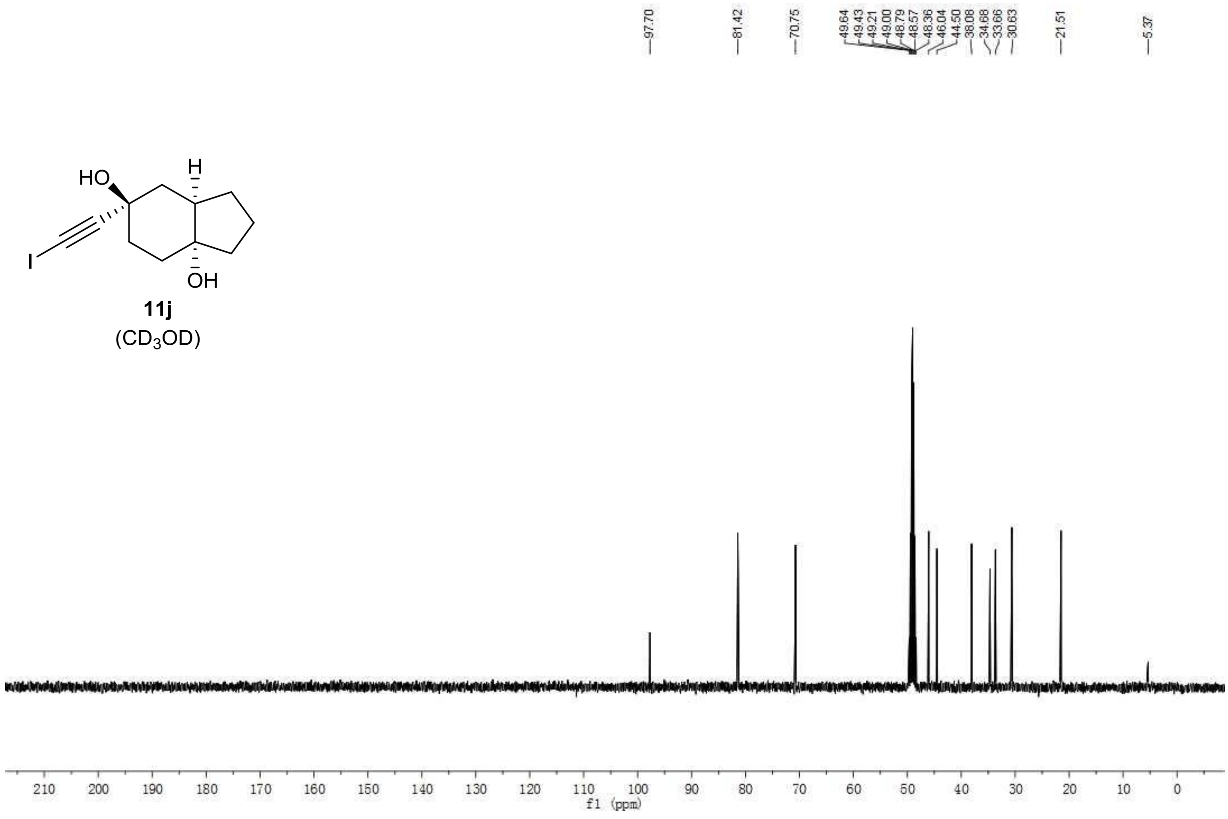

Supplementary Figure 18. **^{1}H and ^{13}C NMR spectra for 11j.**

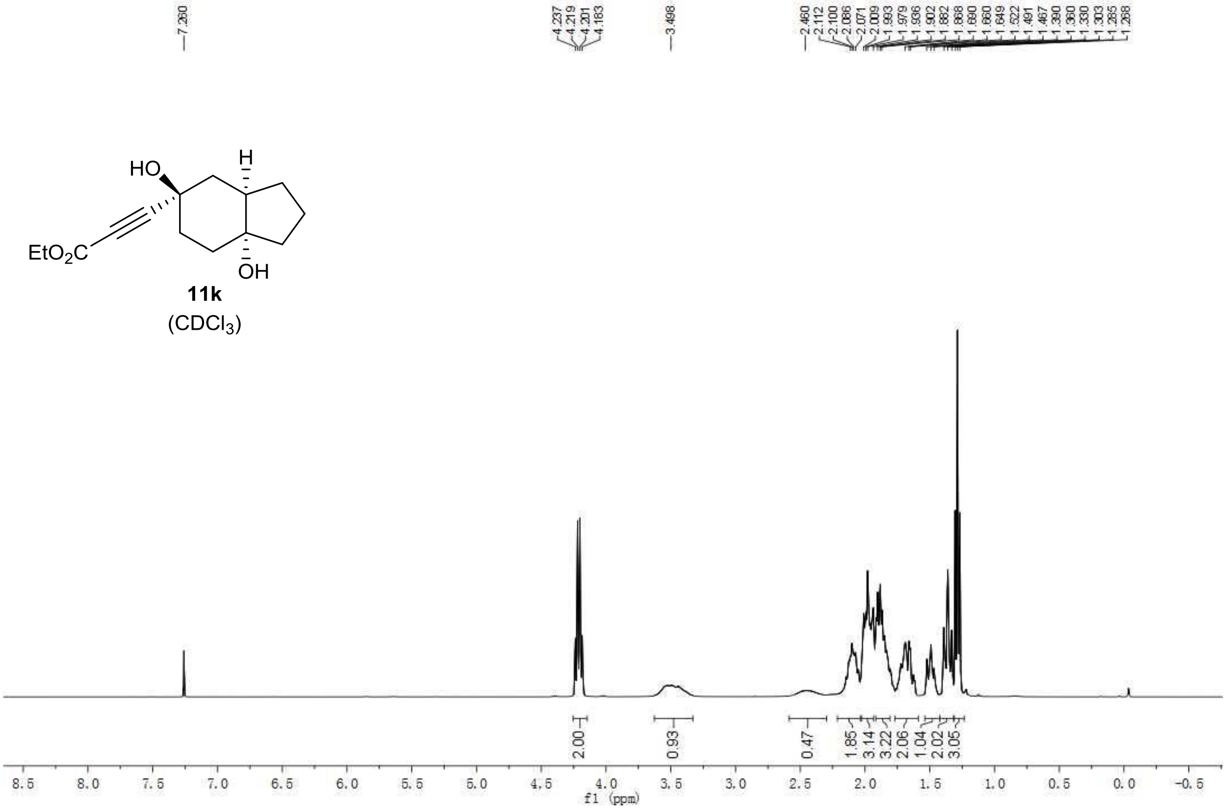

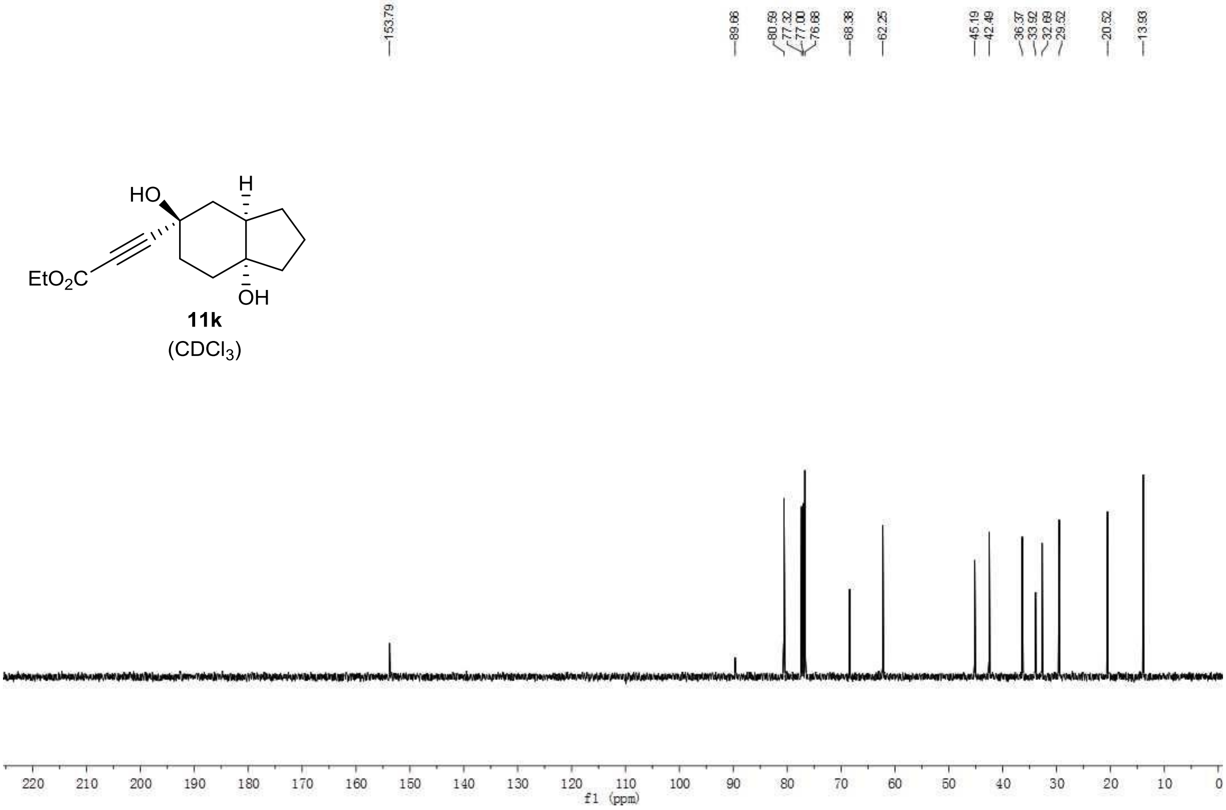

Supplementary Figure 19. ^{1}H and ^{13}C NMR spectra for 11k.

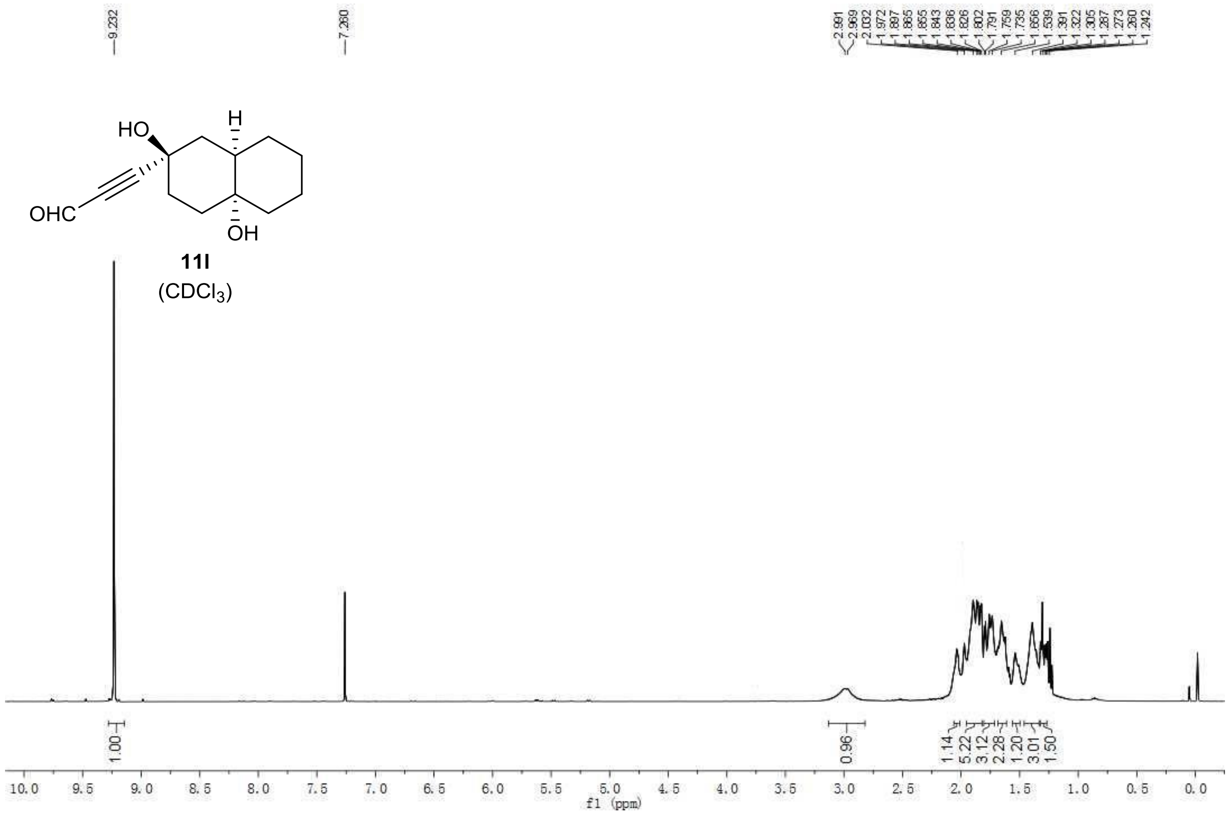

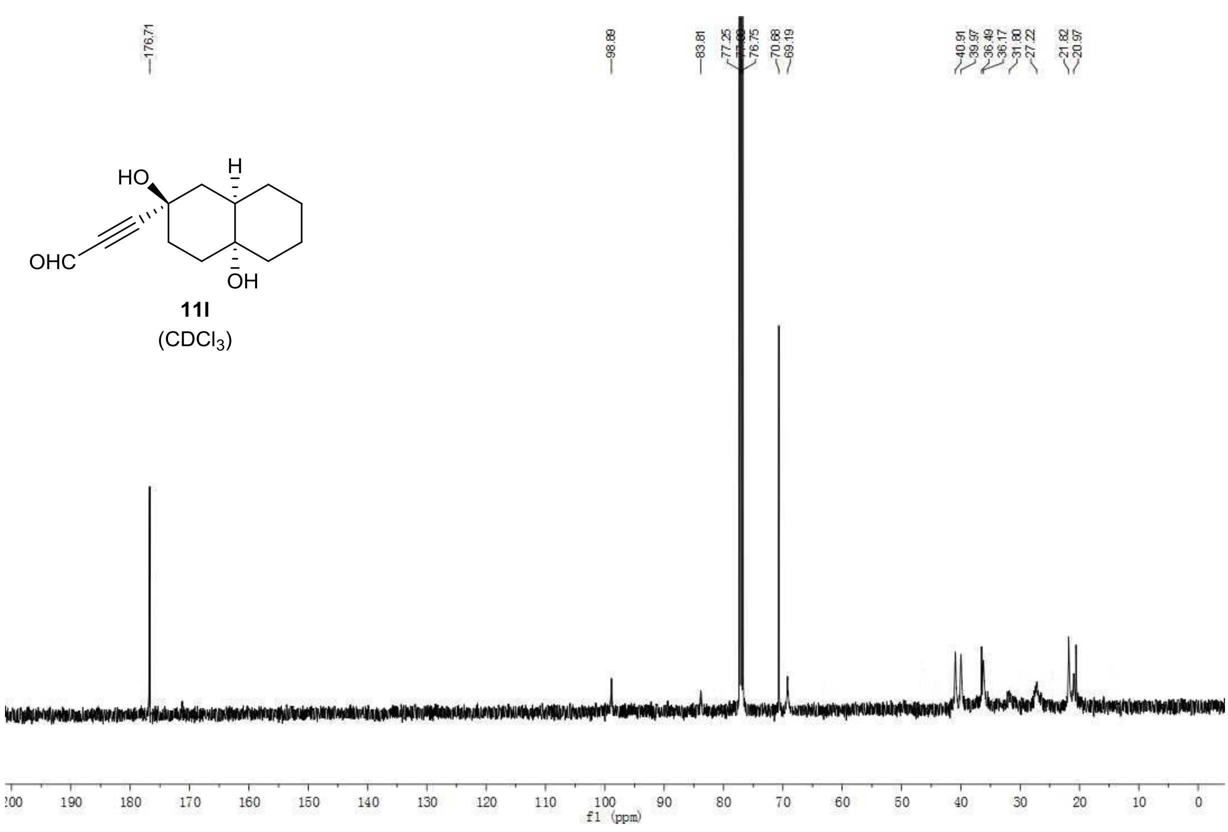

Supplementary Figure 20. ^{1}H and ^{13}C NMR spectra for 11l.

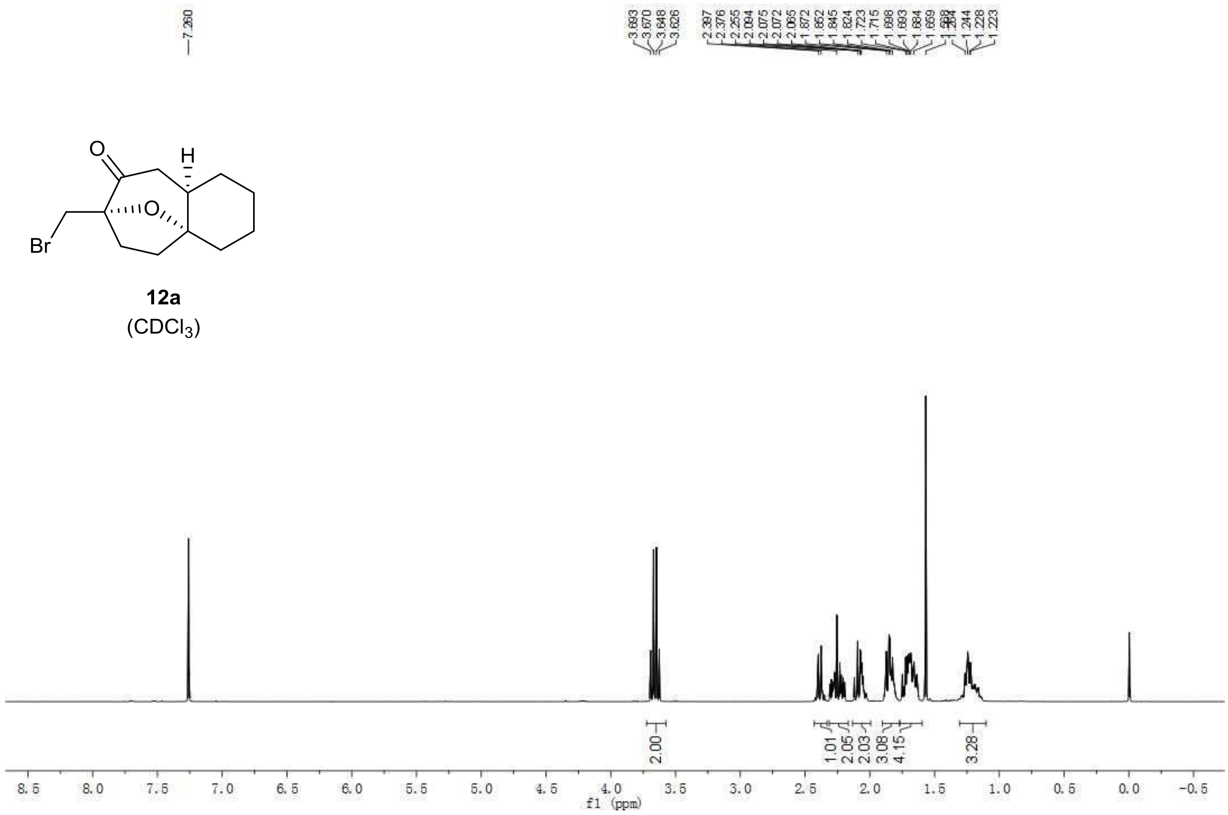

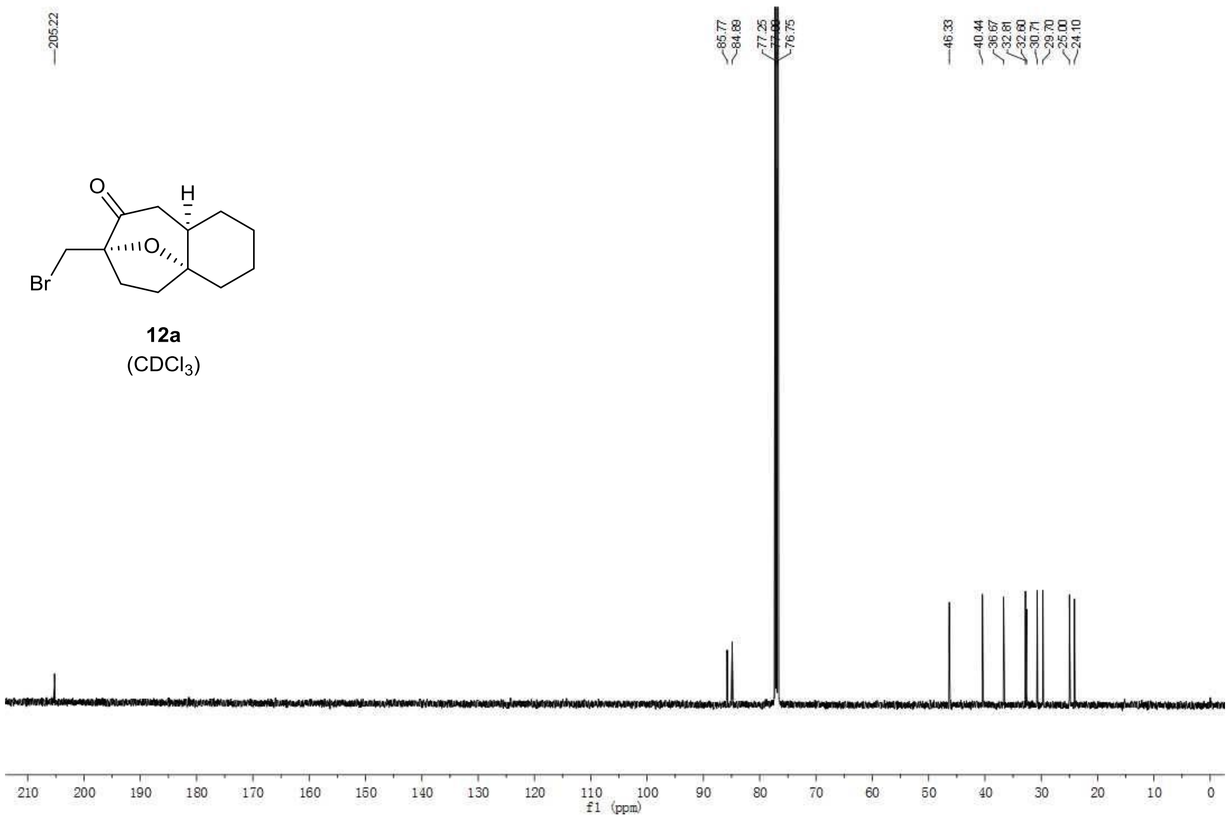

Supplementary Figure 21. ^{1}H and ^{13}C NMR spectra for 12a.

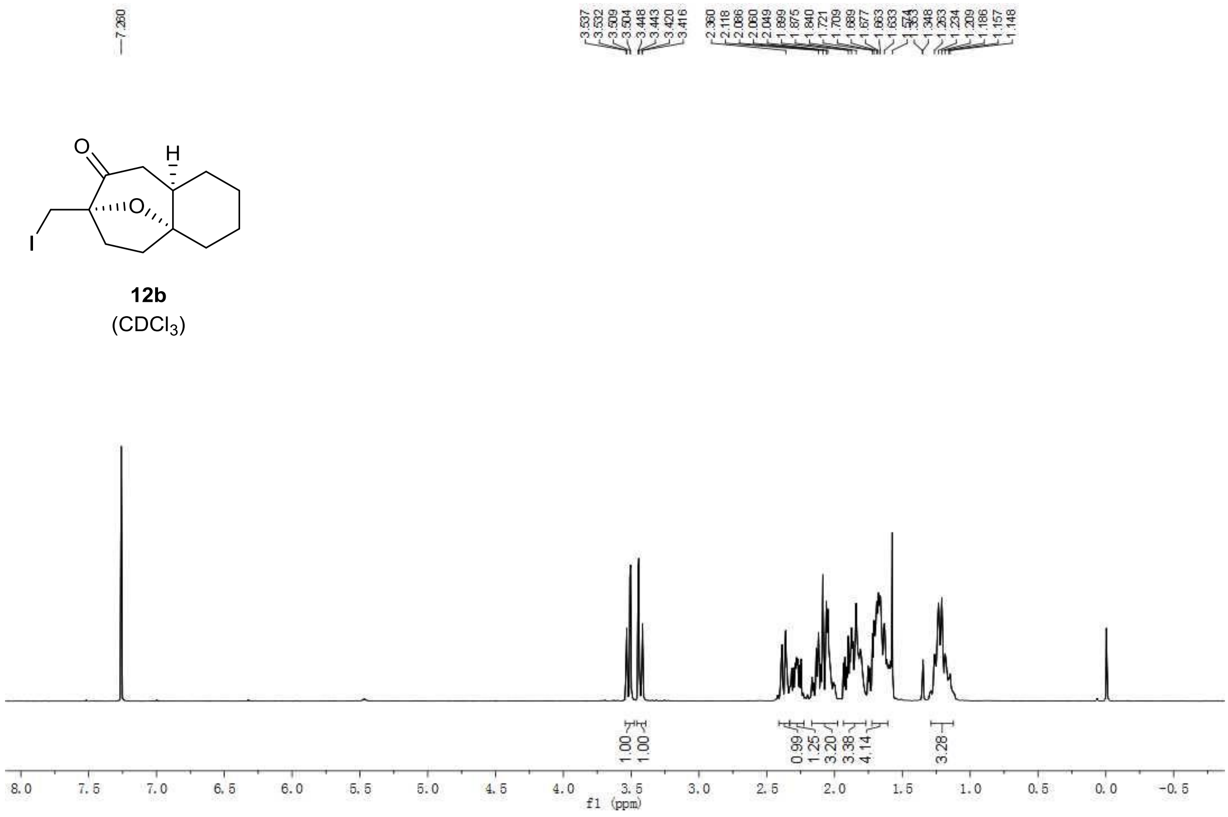

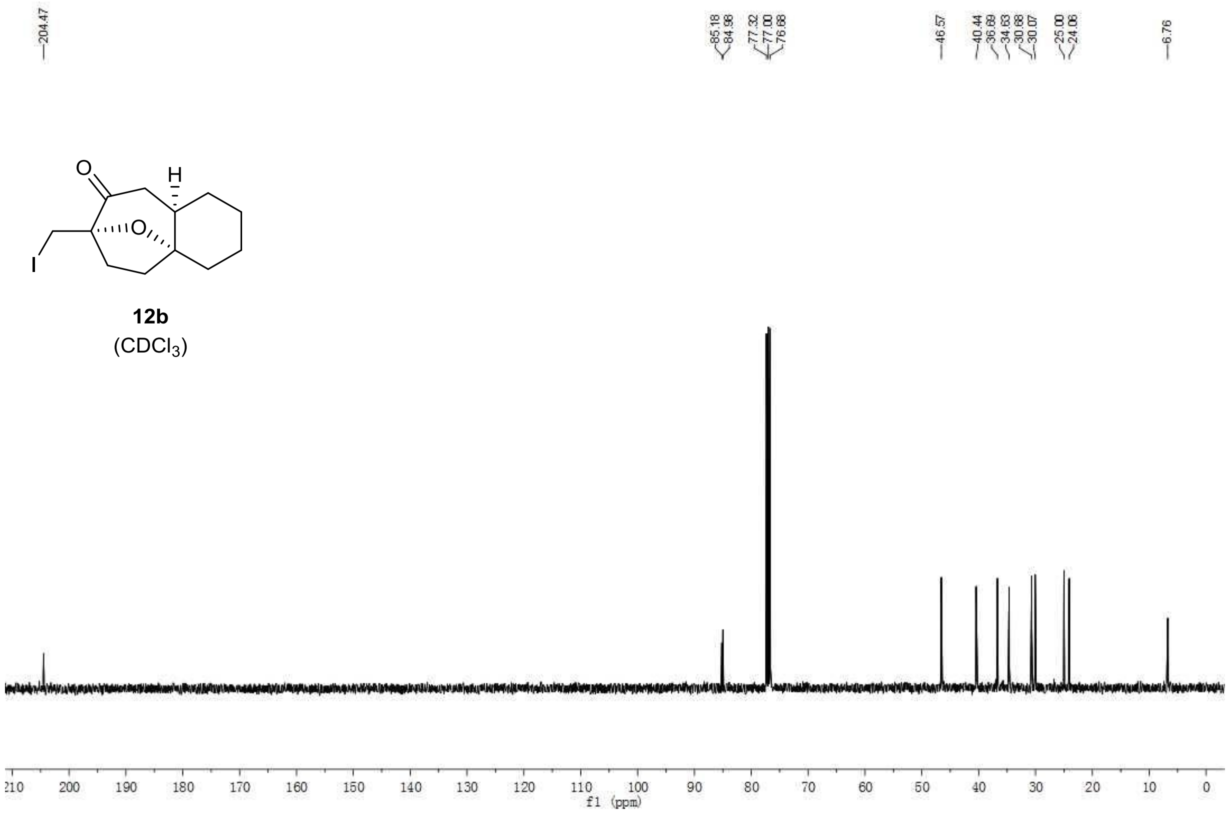

Supplementary Figure 22. ^{1}H and ^{13}C NMR spectra for 12b.

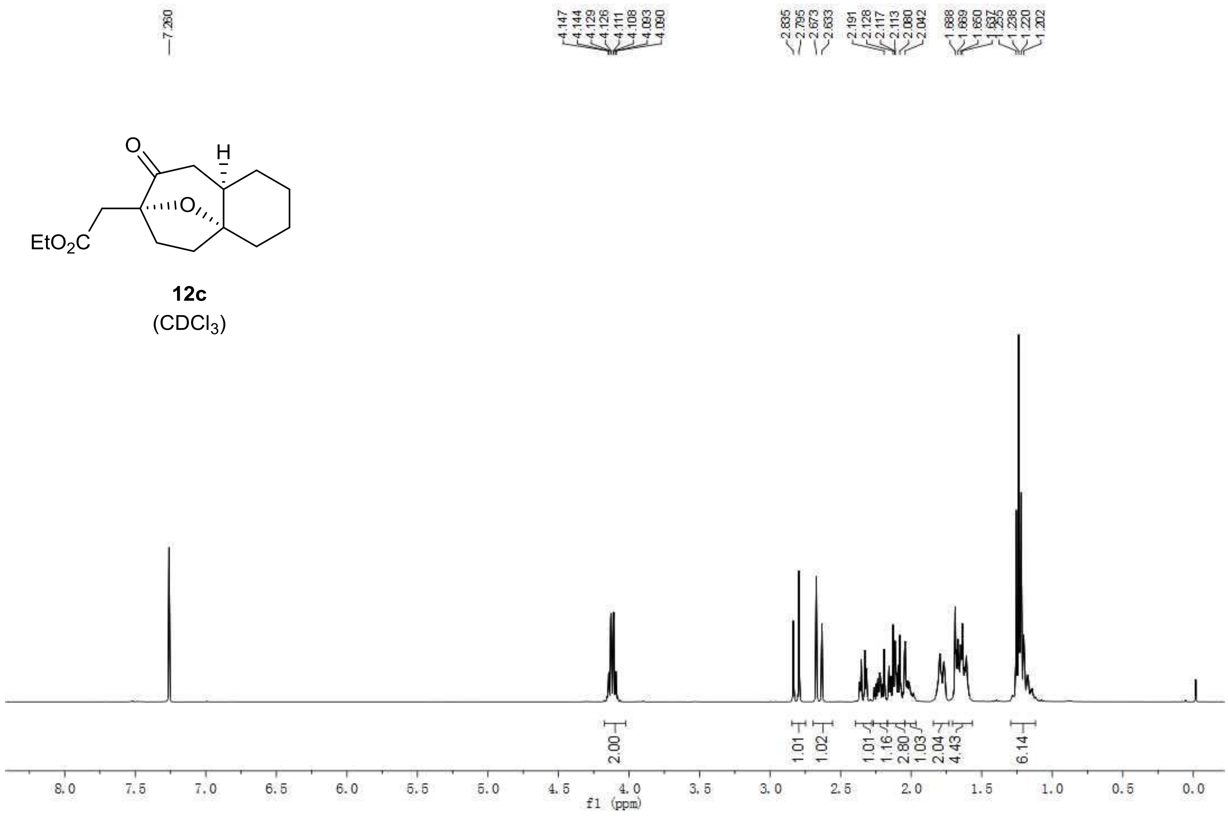

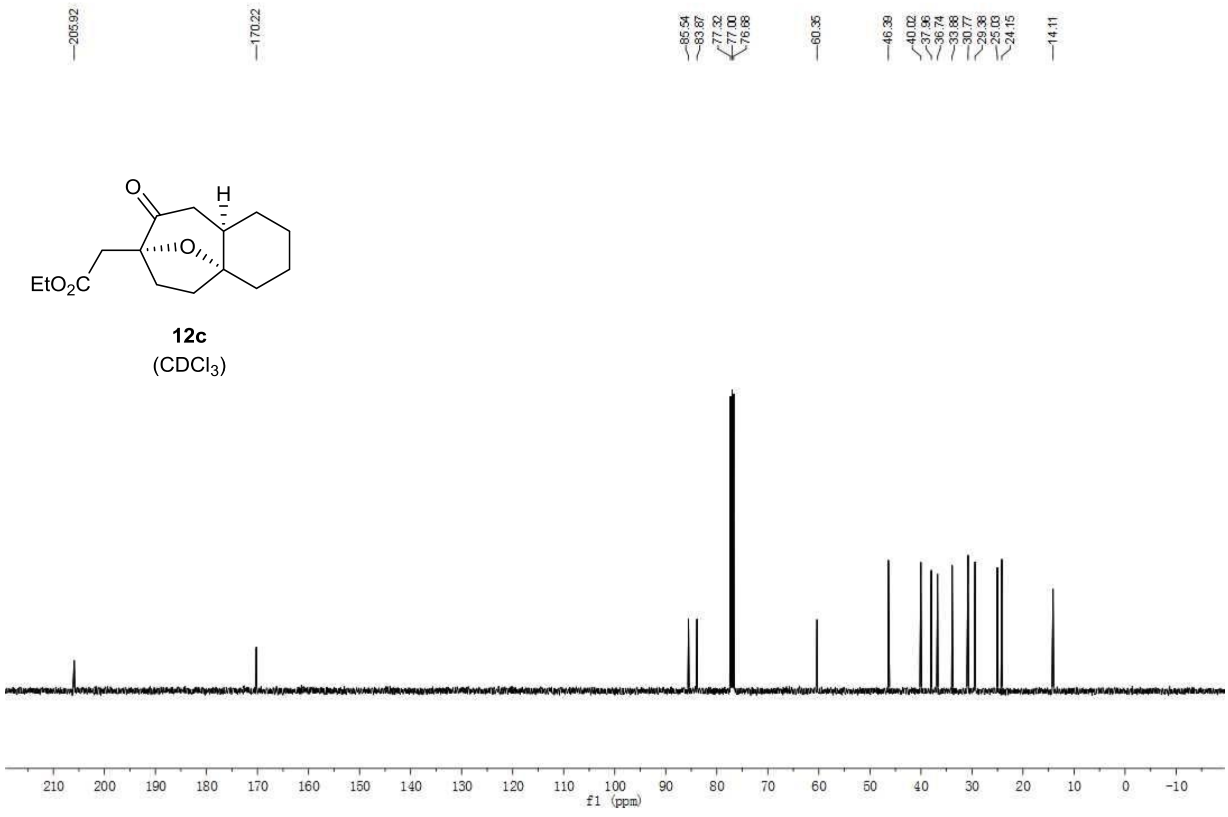

Supplementary Figure 23. ^{1}**H and** ^{13}**C NMR spectra for 12c.**

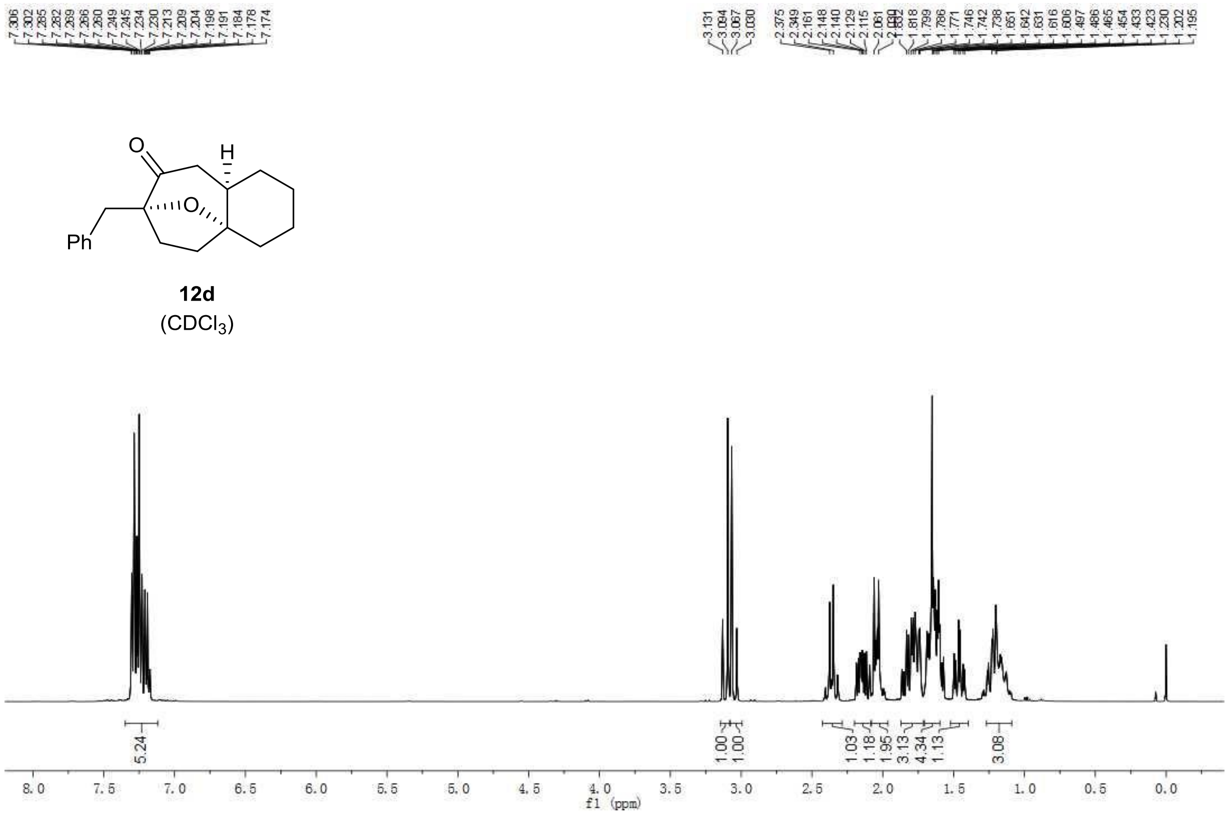

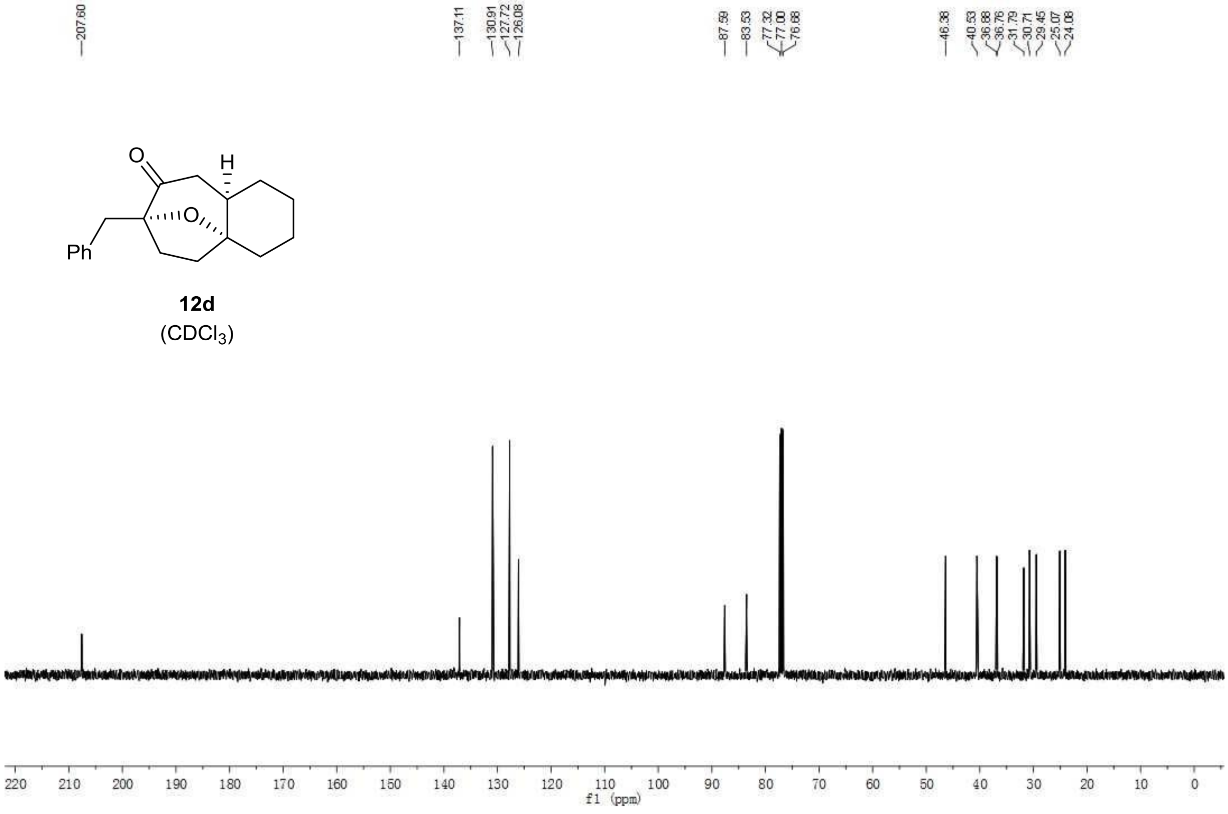

Supplementary Figure 24. ^{1}H and ^{13}C NMR spectra for 12d.

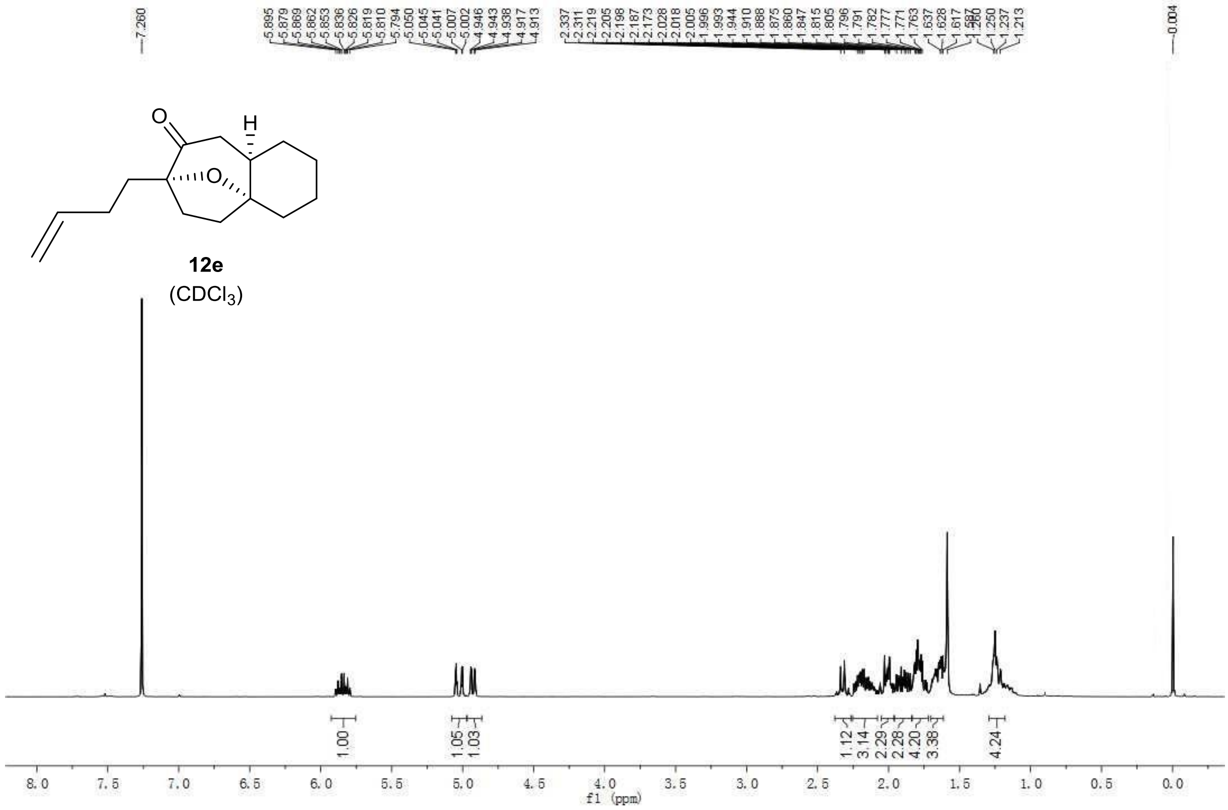

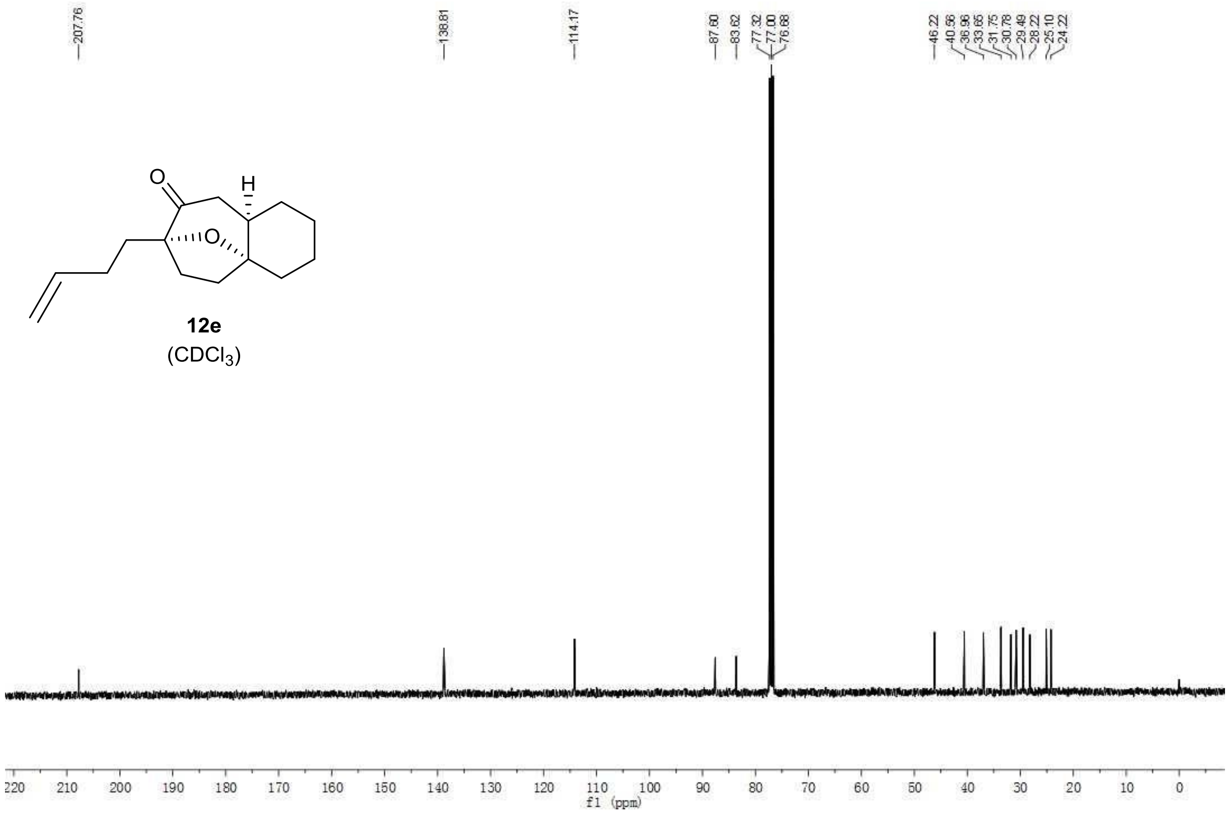

Supplementary Figure 25. ^{1}H and ^{13}C NMR spectra for 12e.

12f
($CDCl_3$)

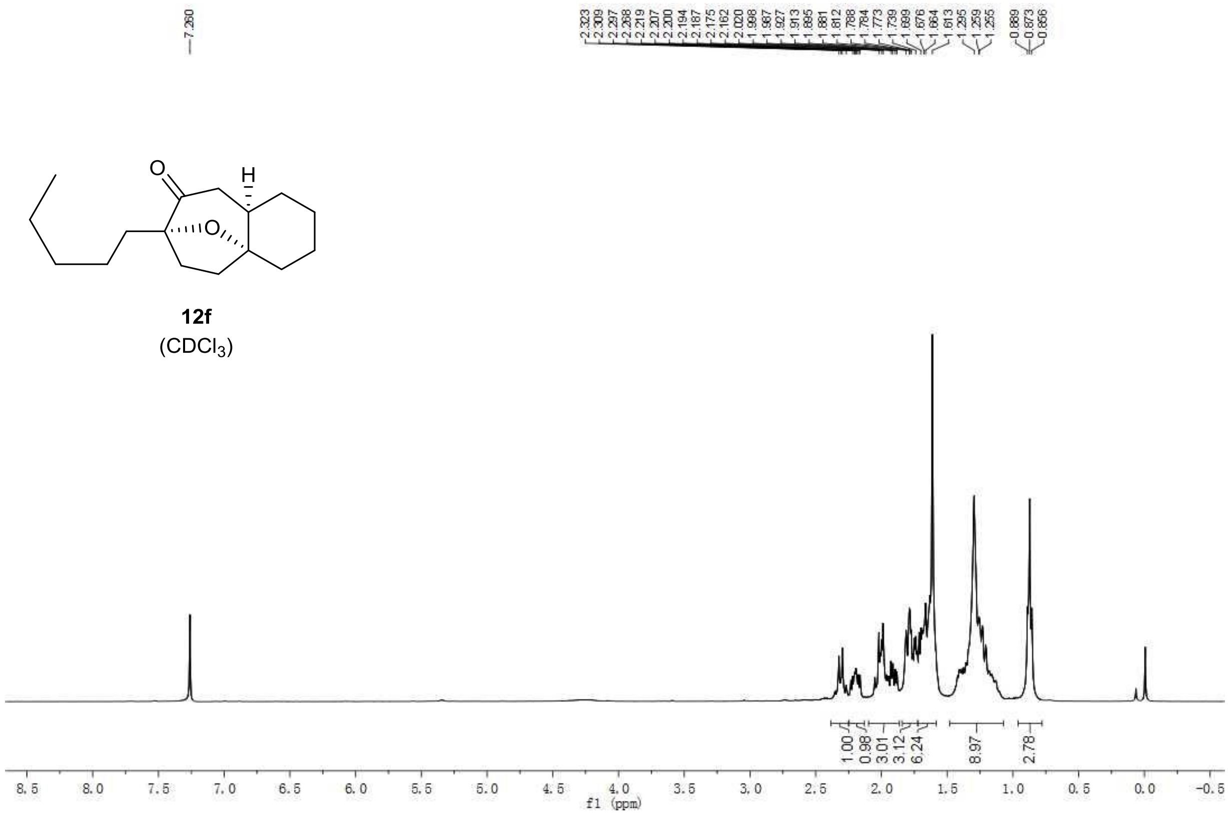

12f
($CDCl_3$)

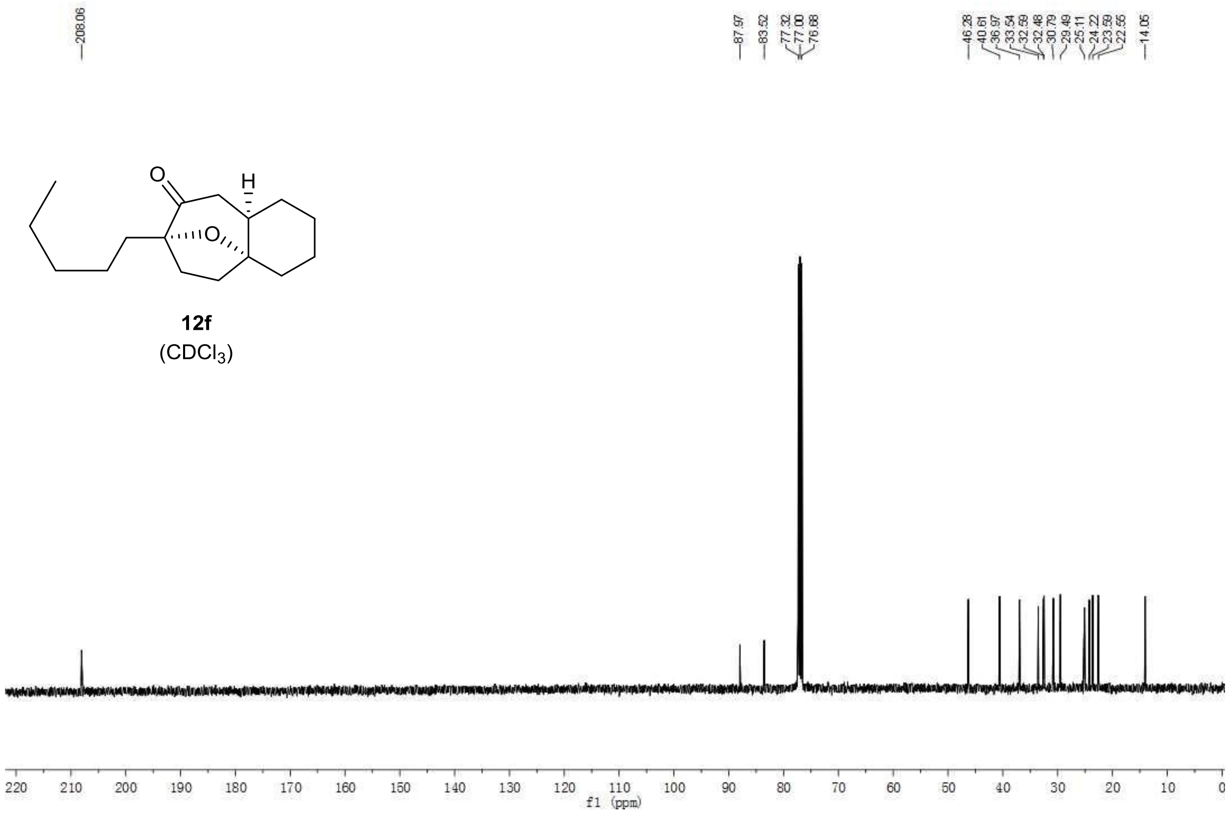

Supplementary Figure 26. ^{1}H and ^{13}C NMR spectra for 12f.

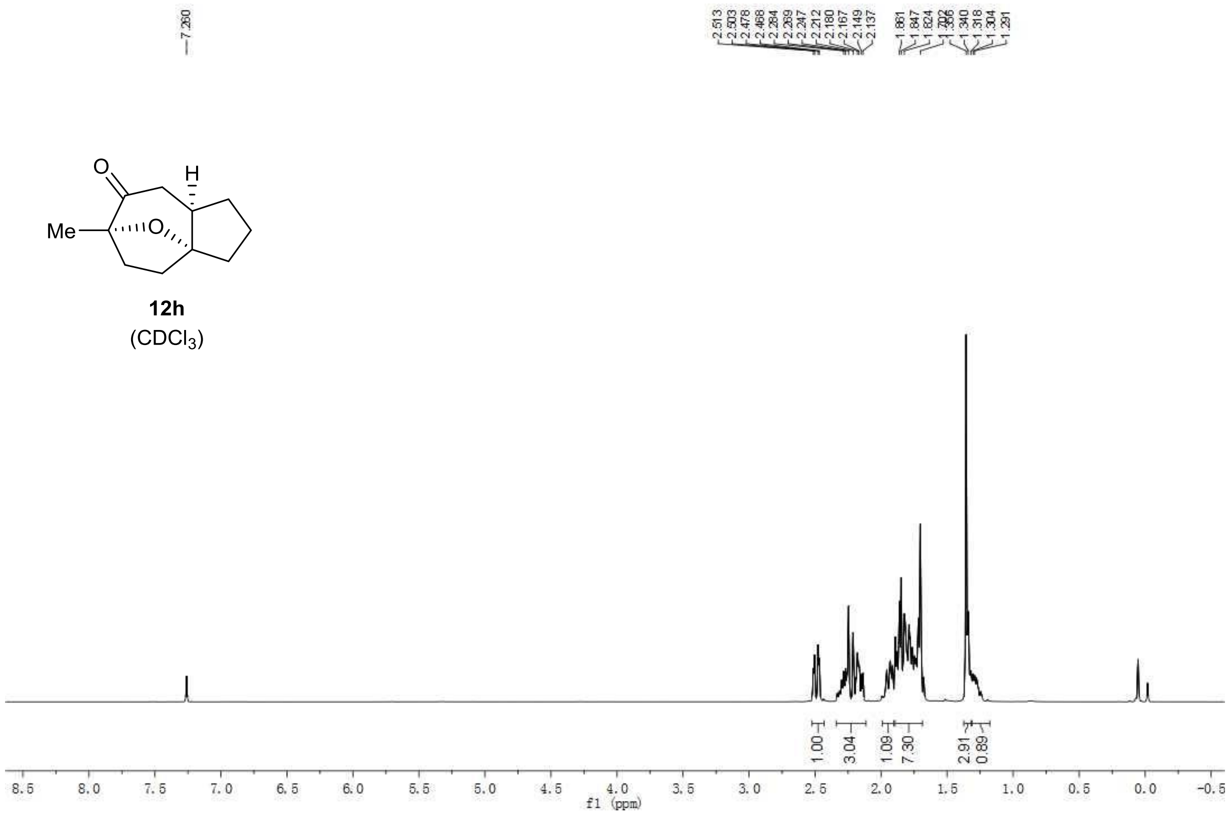

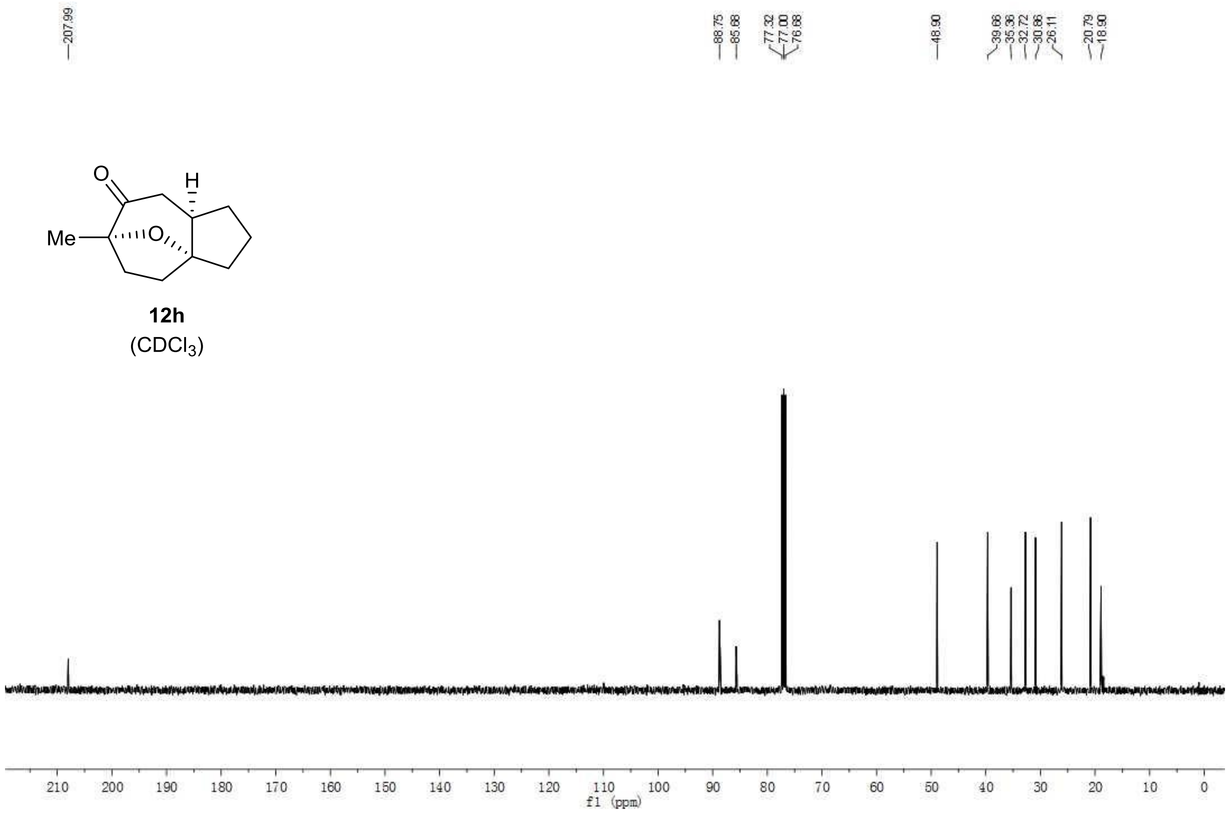

Supplementary Figure 27. ^{1}H and ^{13}C NMR spectra for 12h.

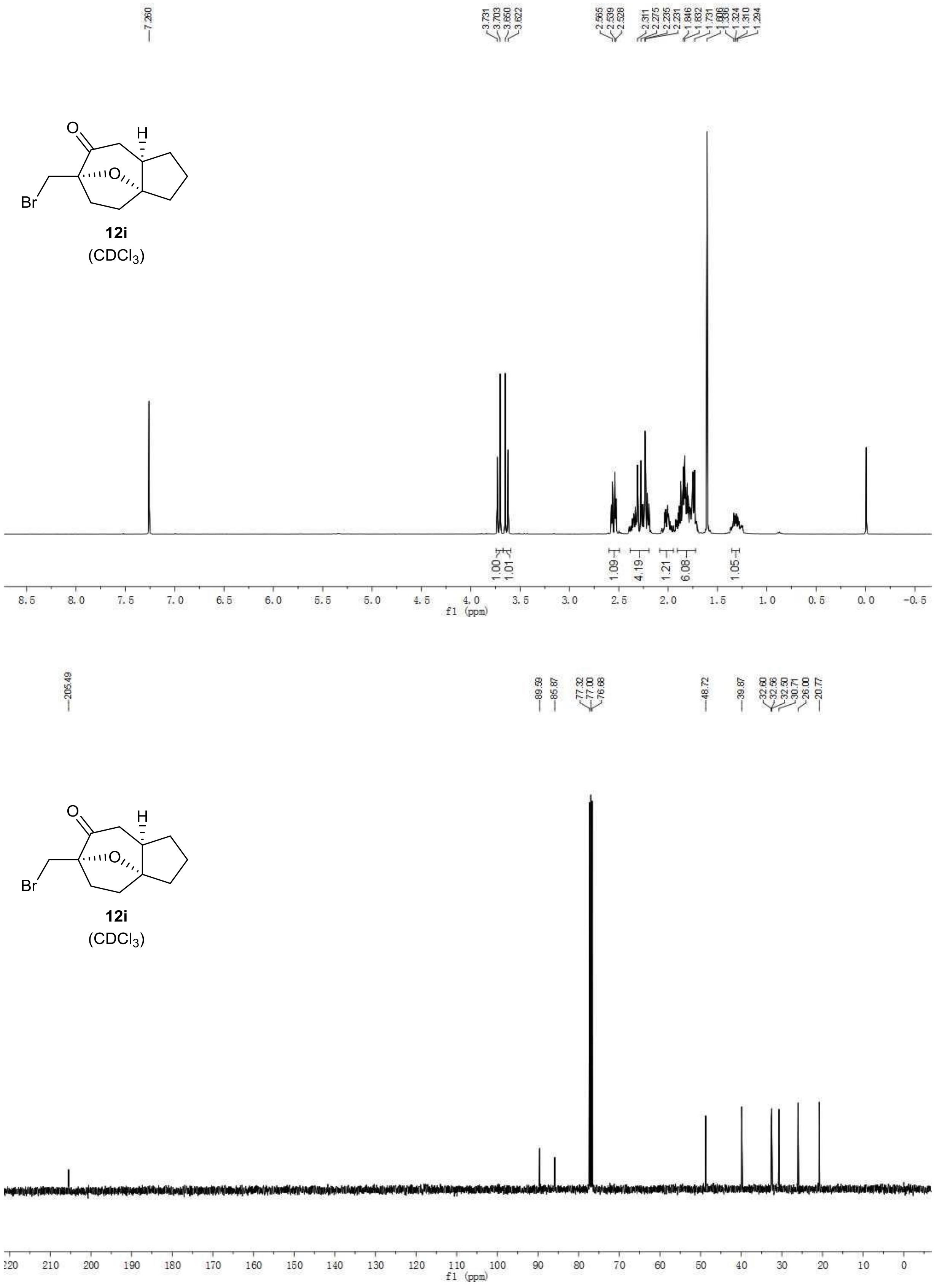

Supplementary Figure 28. ^{1}H and ^{13}C NMR spectra for 12i.

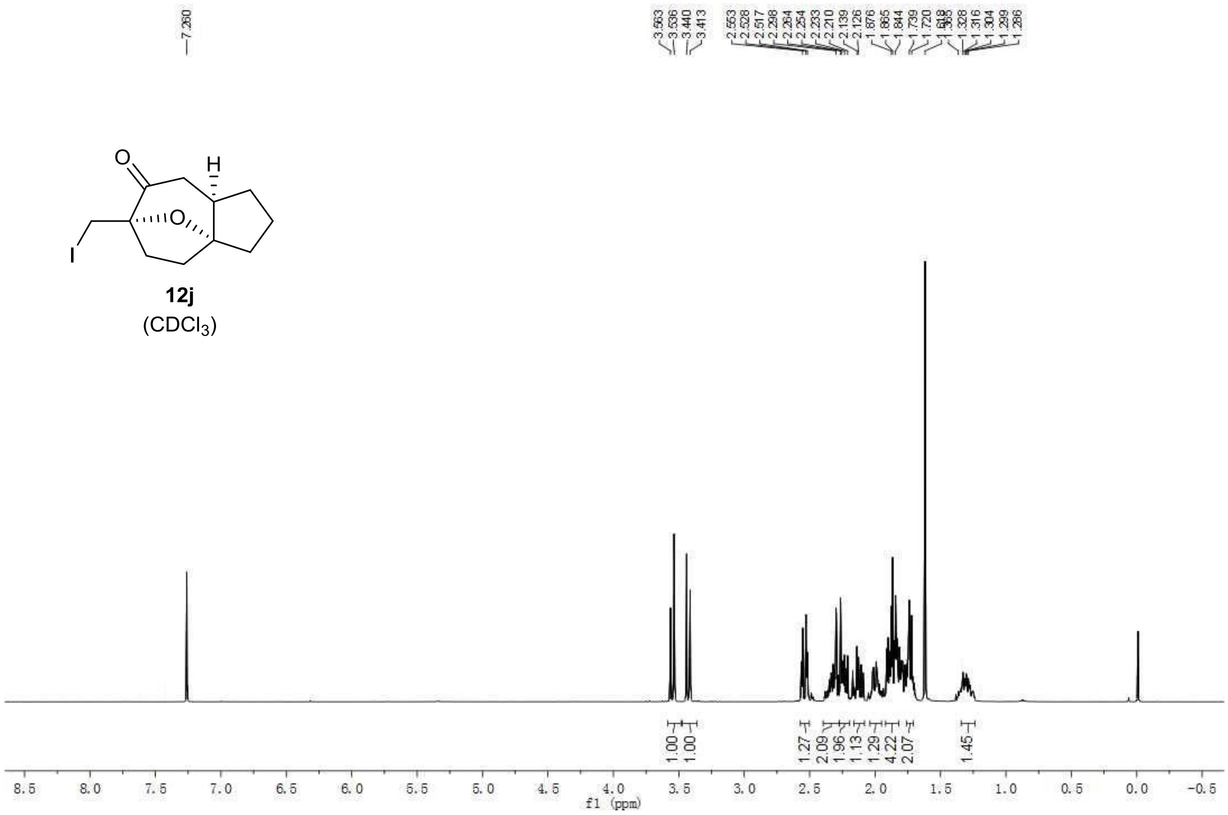

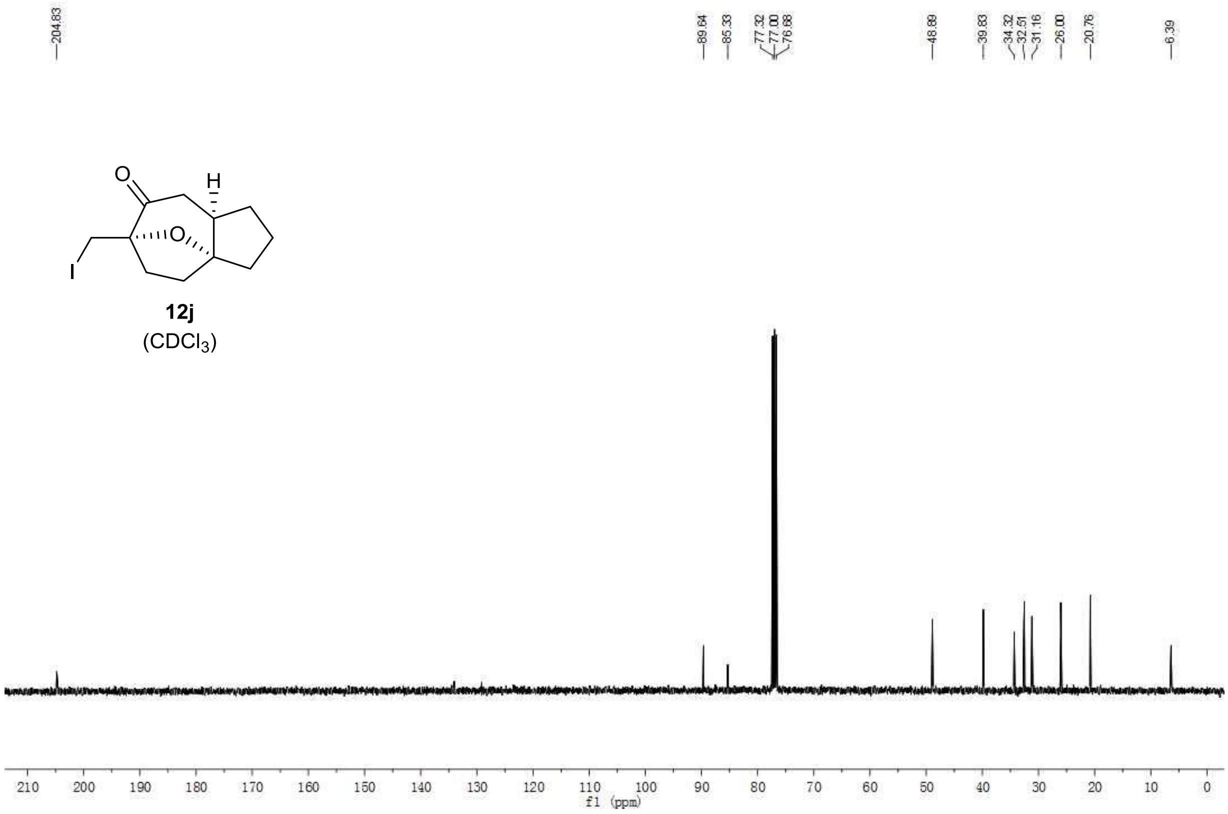

Supplementary Figure 29. ^{1}H and ^{13}C NMR spectra for 12j.

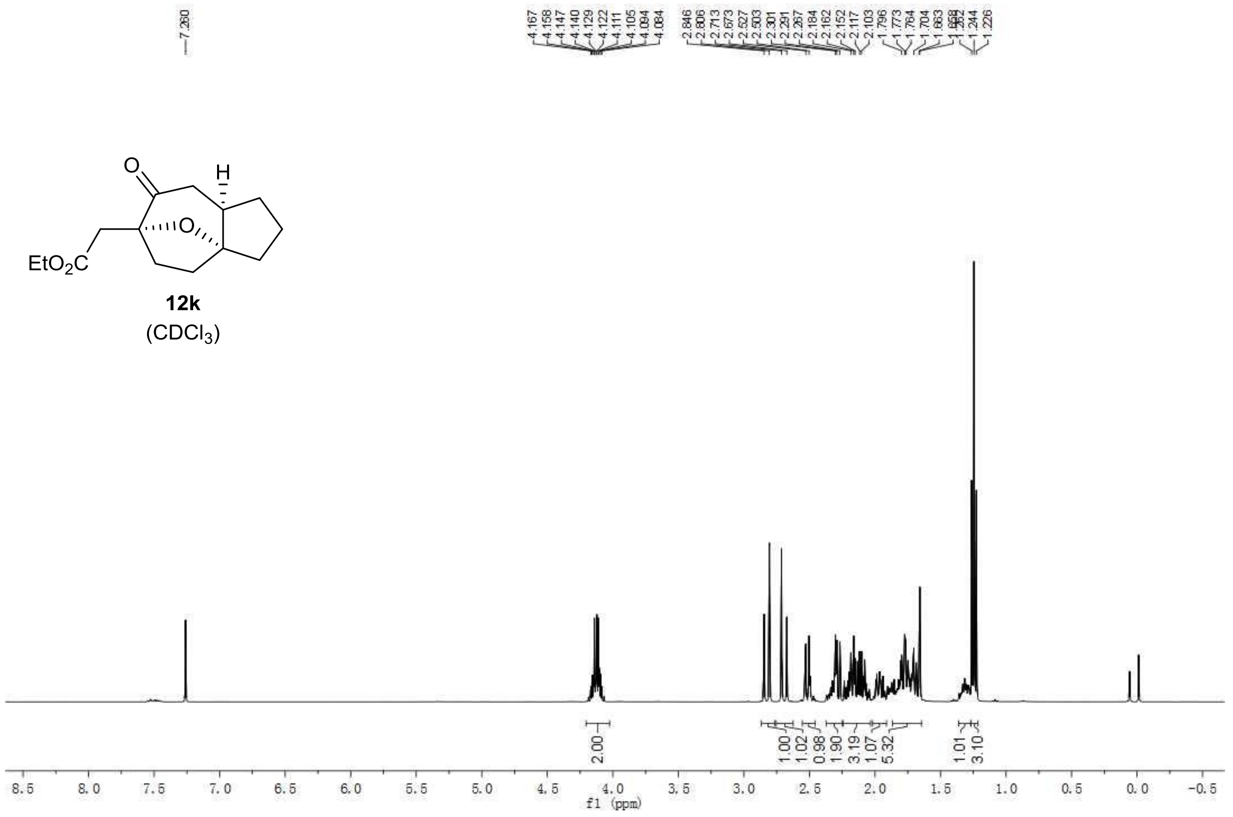

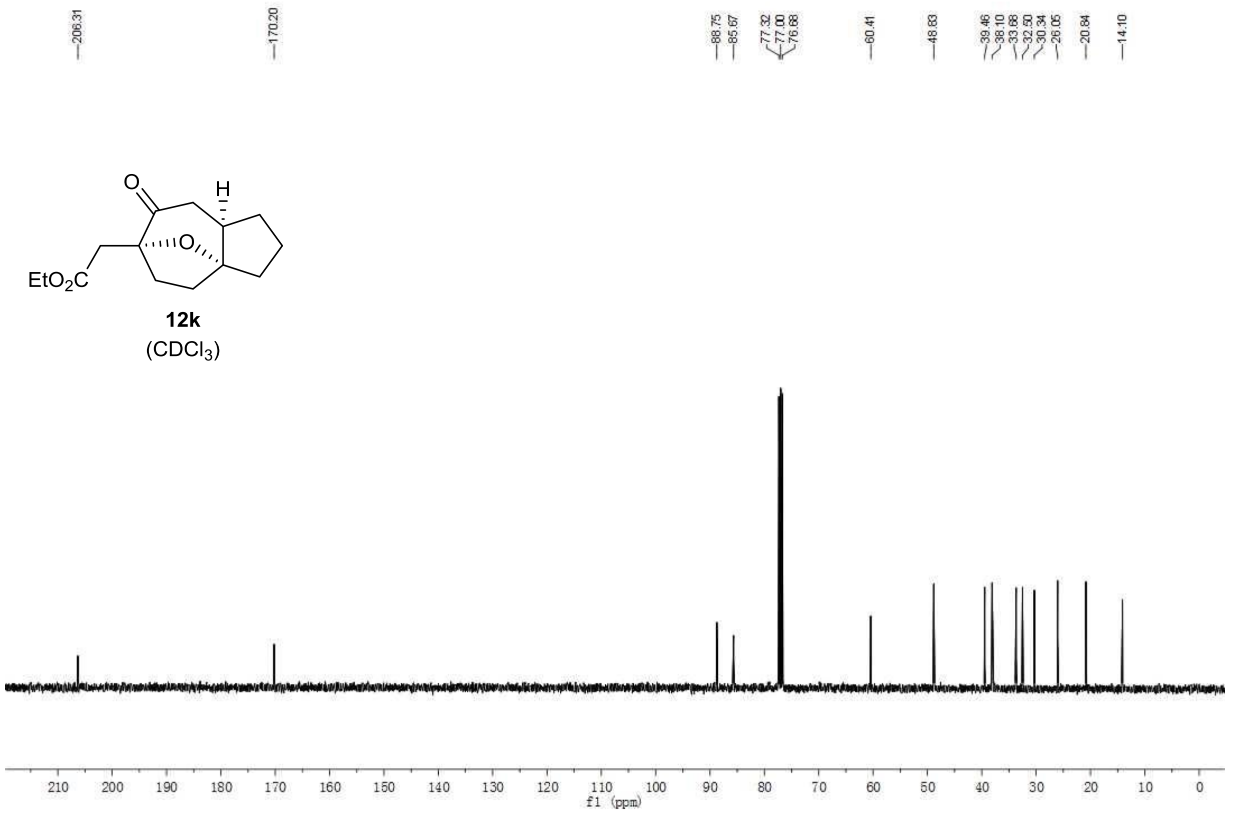

Supplementary Figure 30. ^{1}H and ^{13}C NMR spectra for 12k.

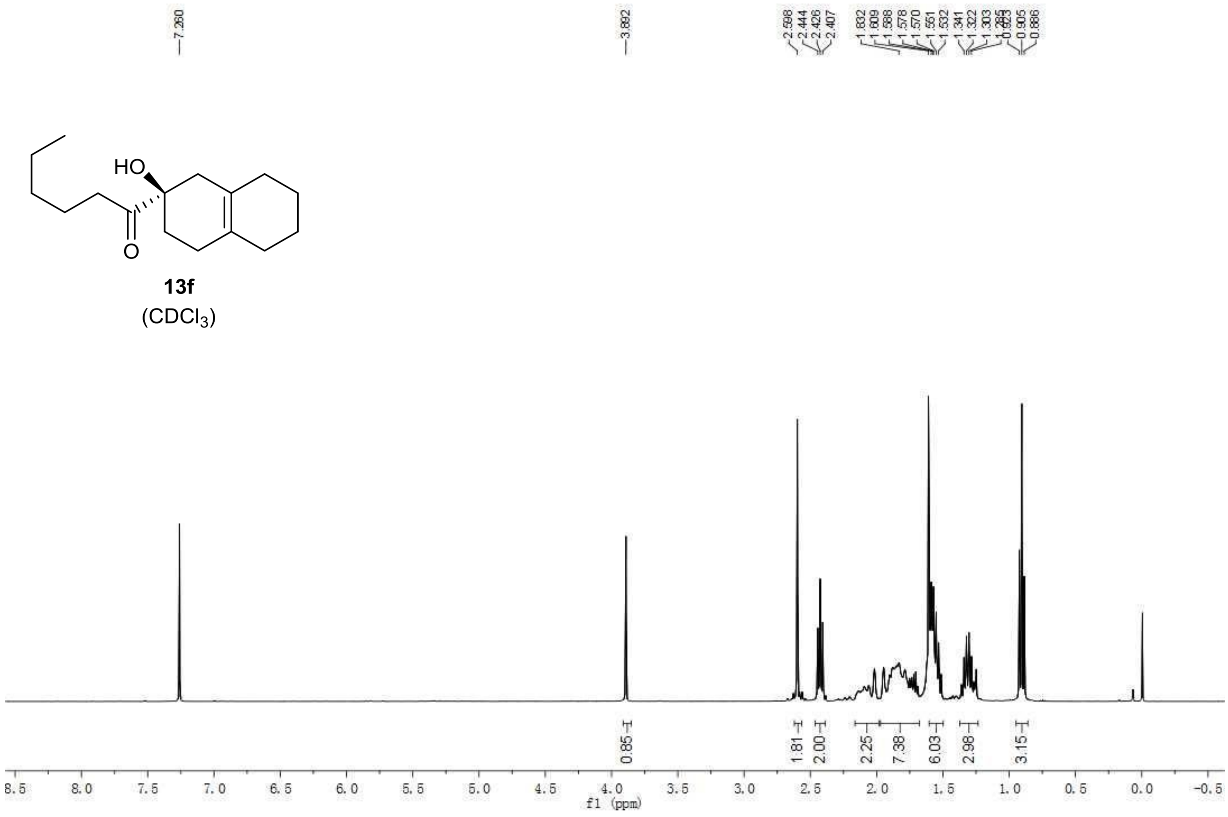

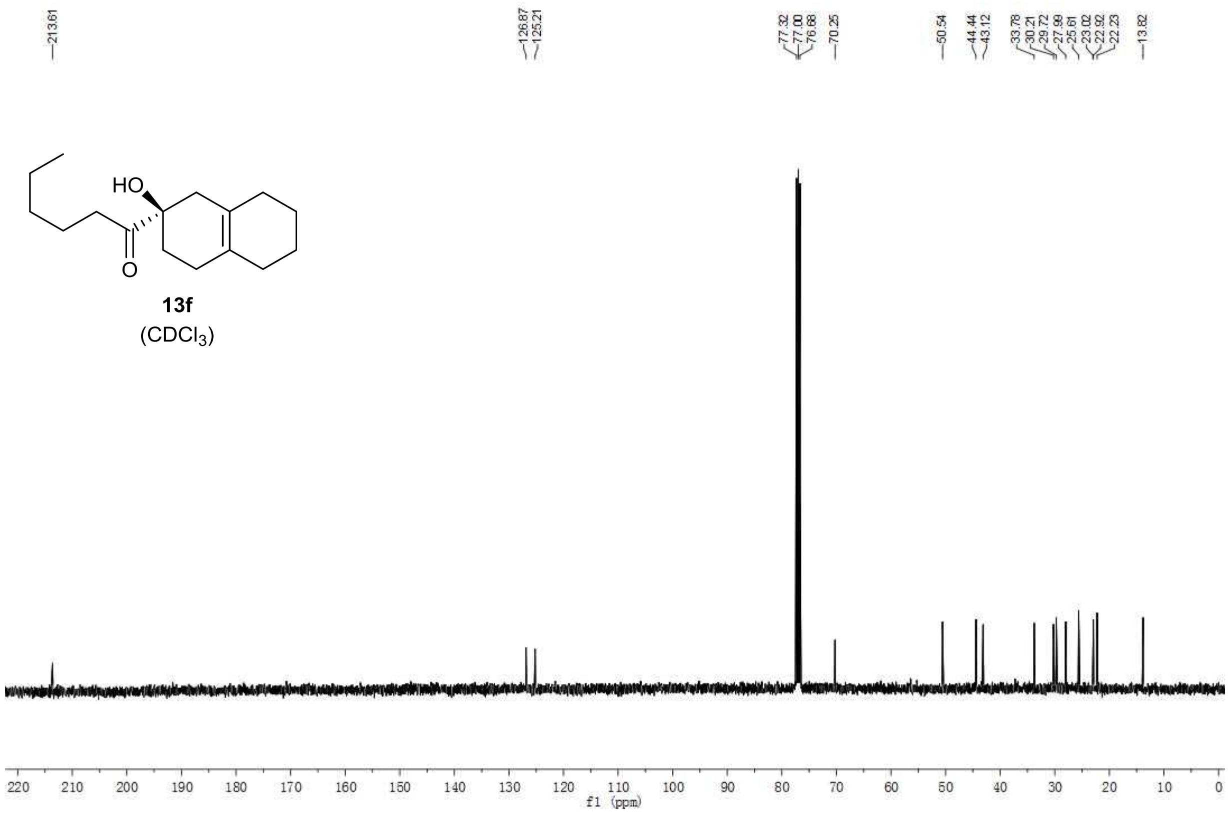

Supplementary Figure 31. ^{1}H and ^{13}C NMR spectra for 13f.

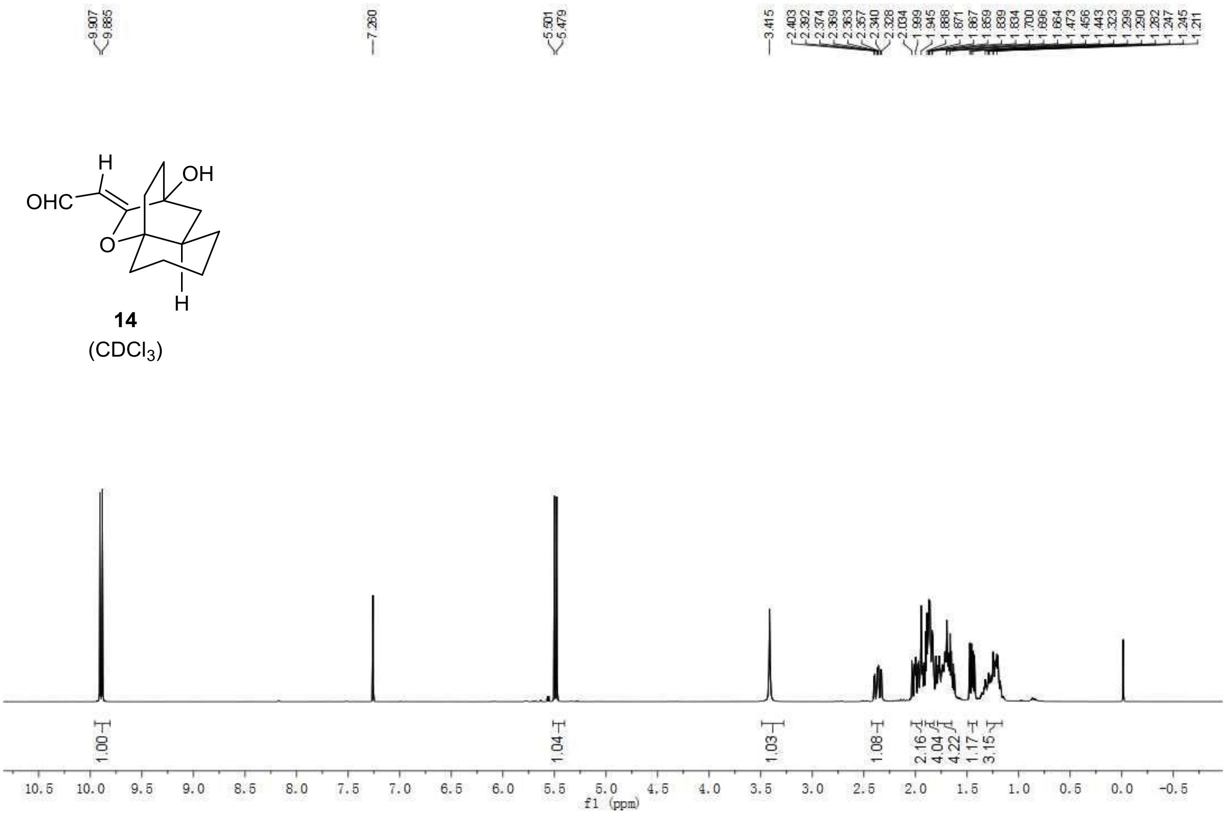

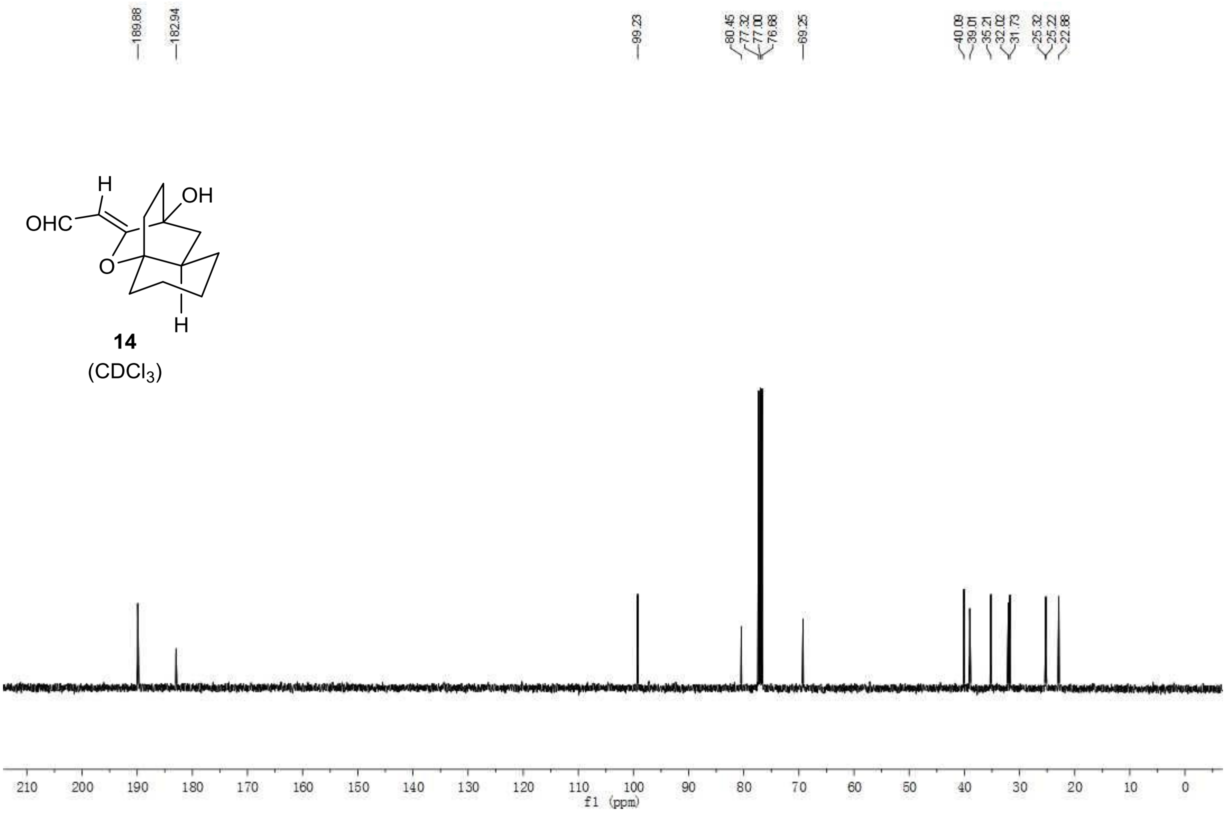

Supplementary Figure 32. ^{1}H and ^{13}C NMR spectra for 14.

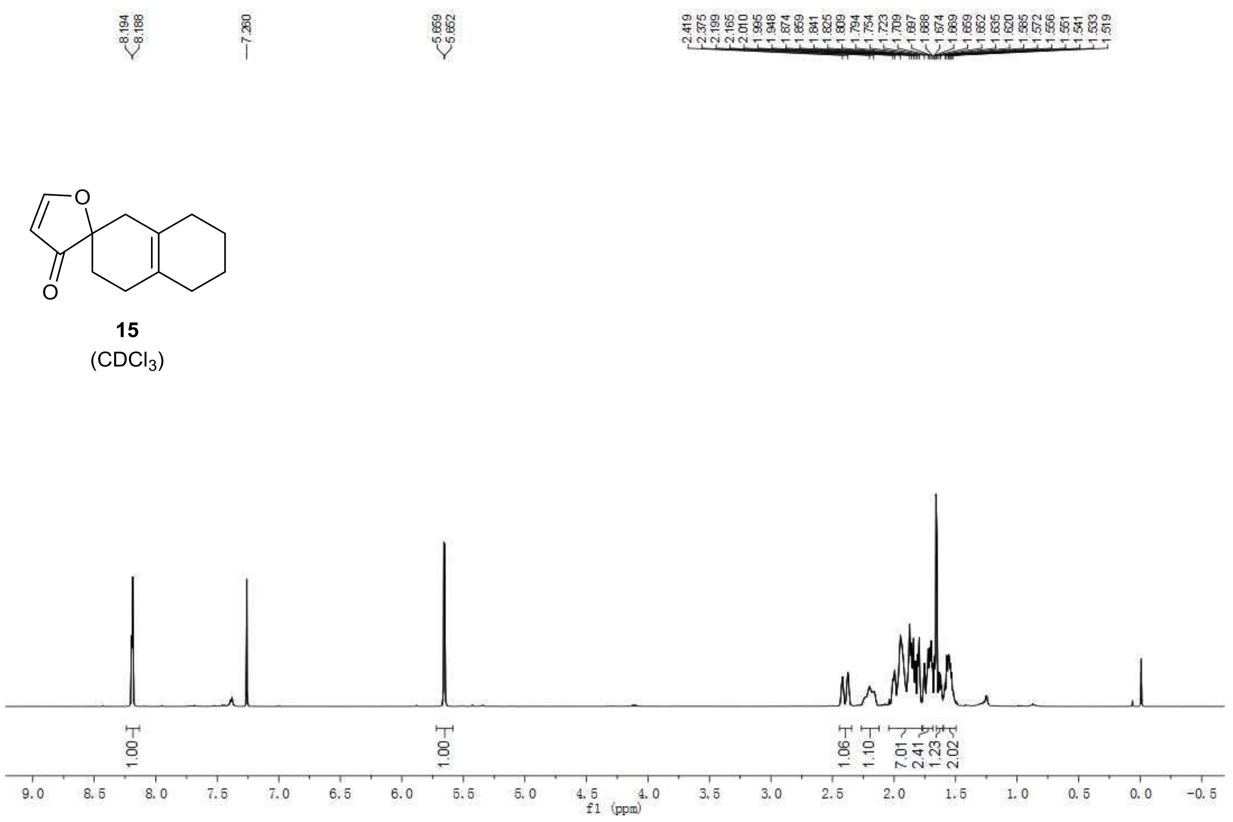

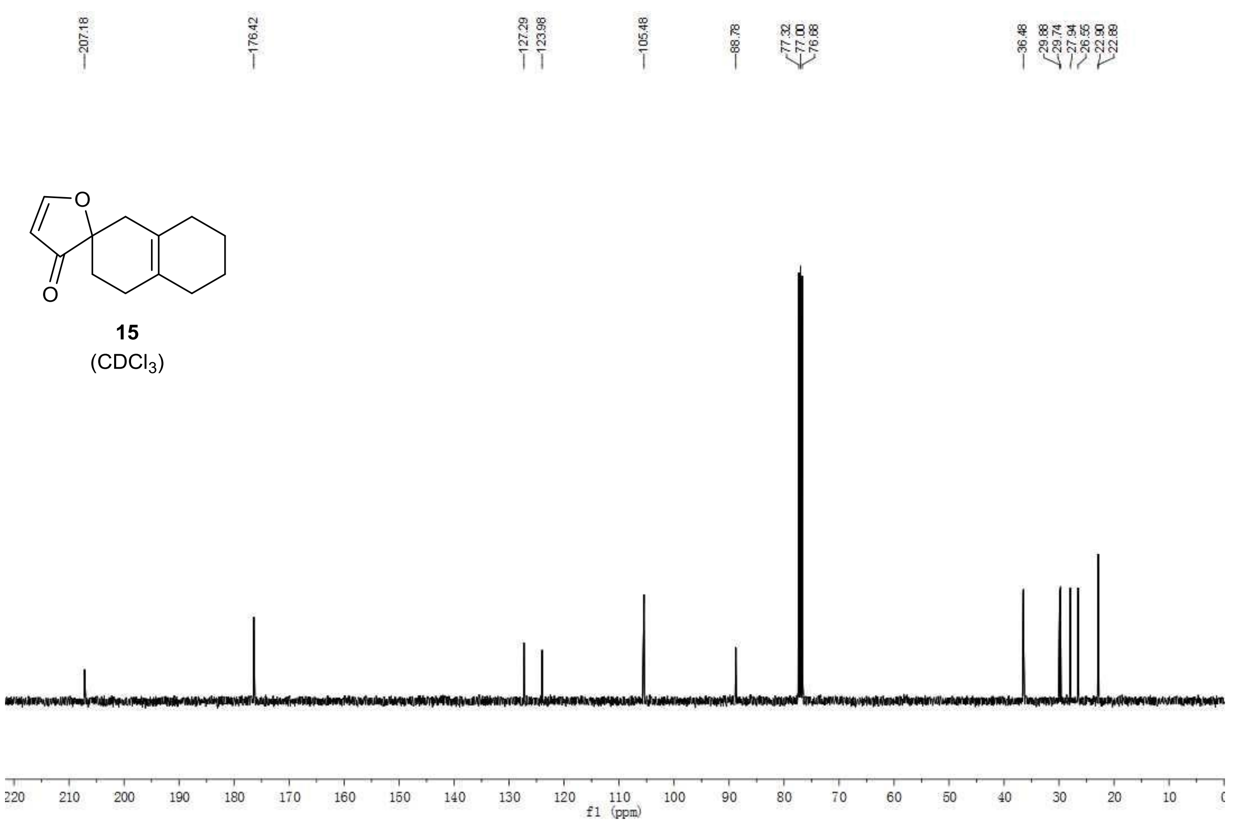

Supplementary Figure 33. ^{1}H and ^{13}C NMR spectra for 15.

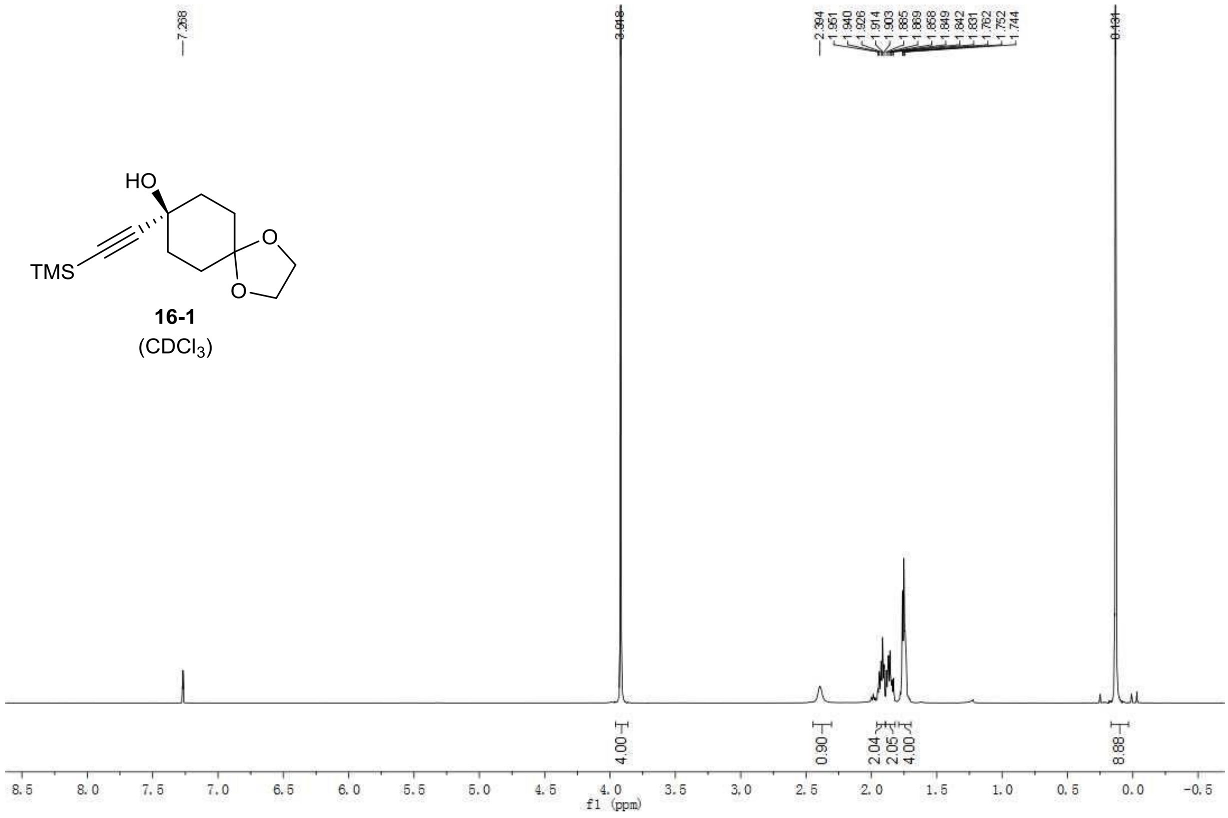

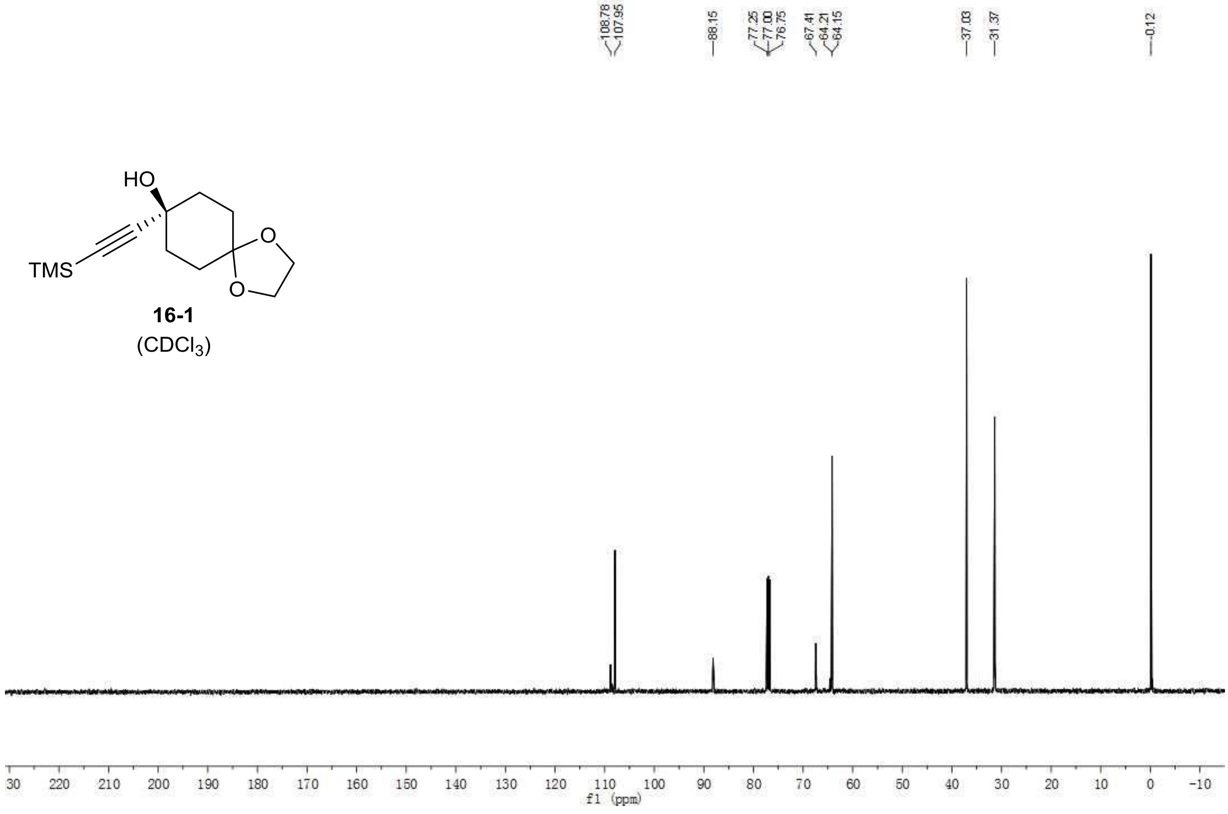

Supplementary Figure 34. ^{1}H and ^{13}C NMR spectra for 16-1.

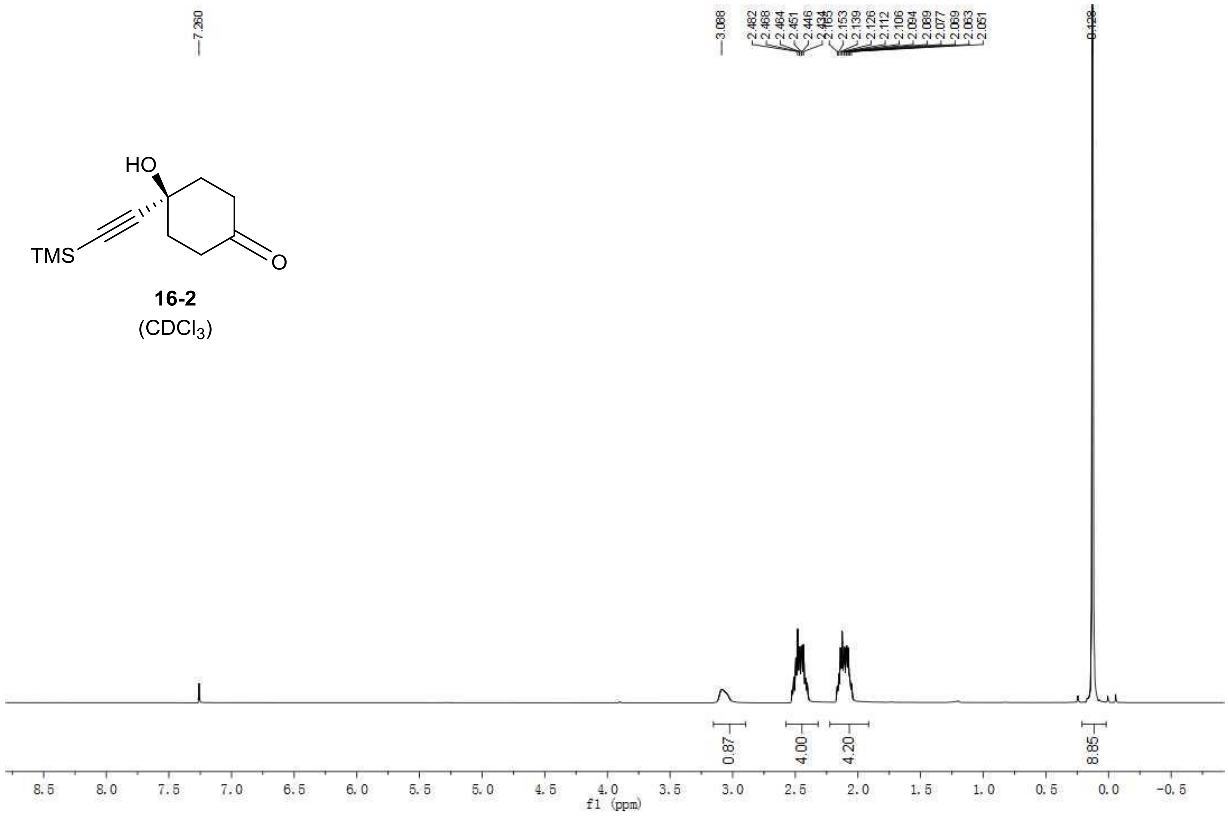

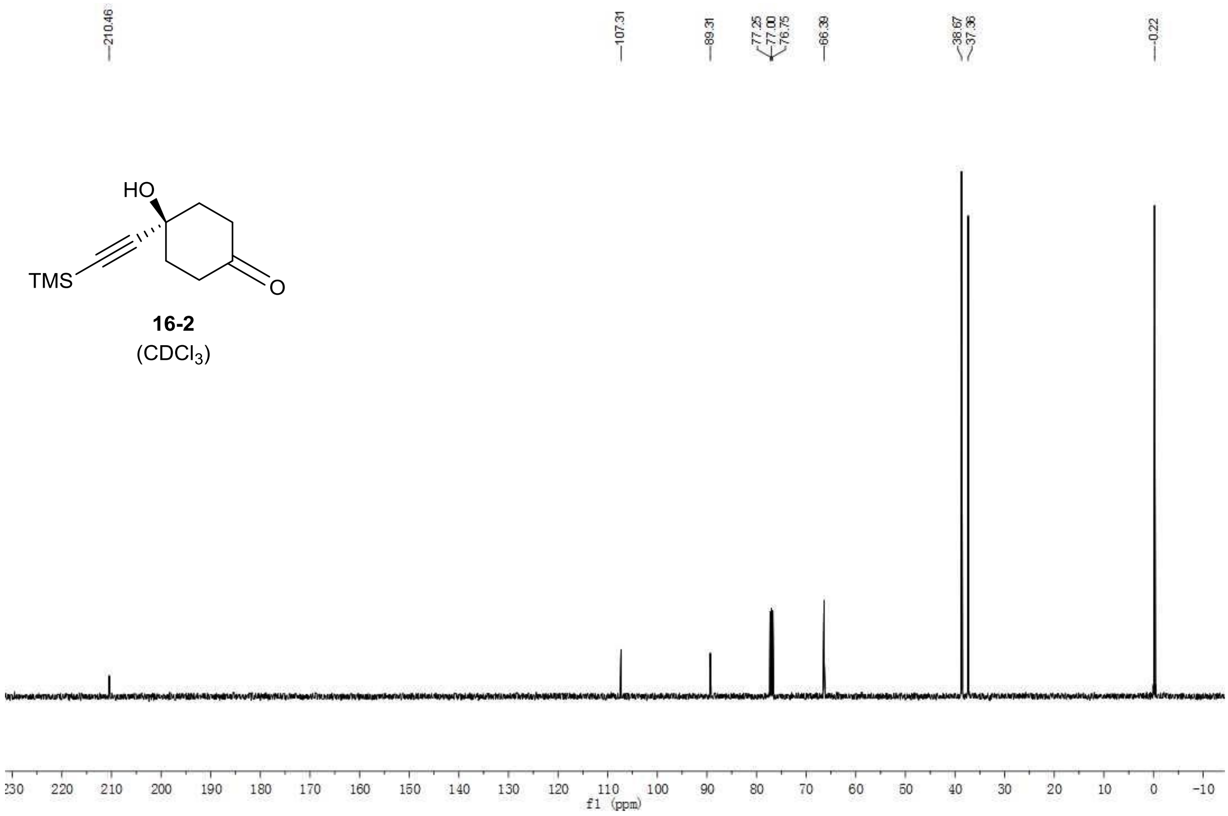

Supplementary Figure 35. ^{1}H and ^{13}C NMR spectra for 16-2.

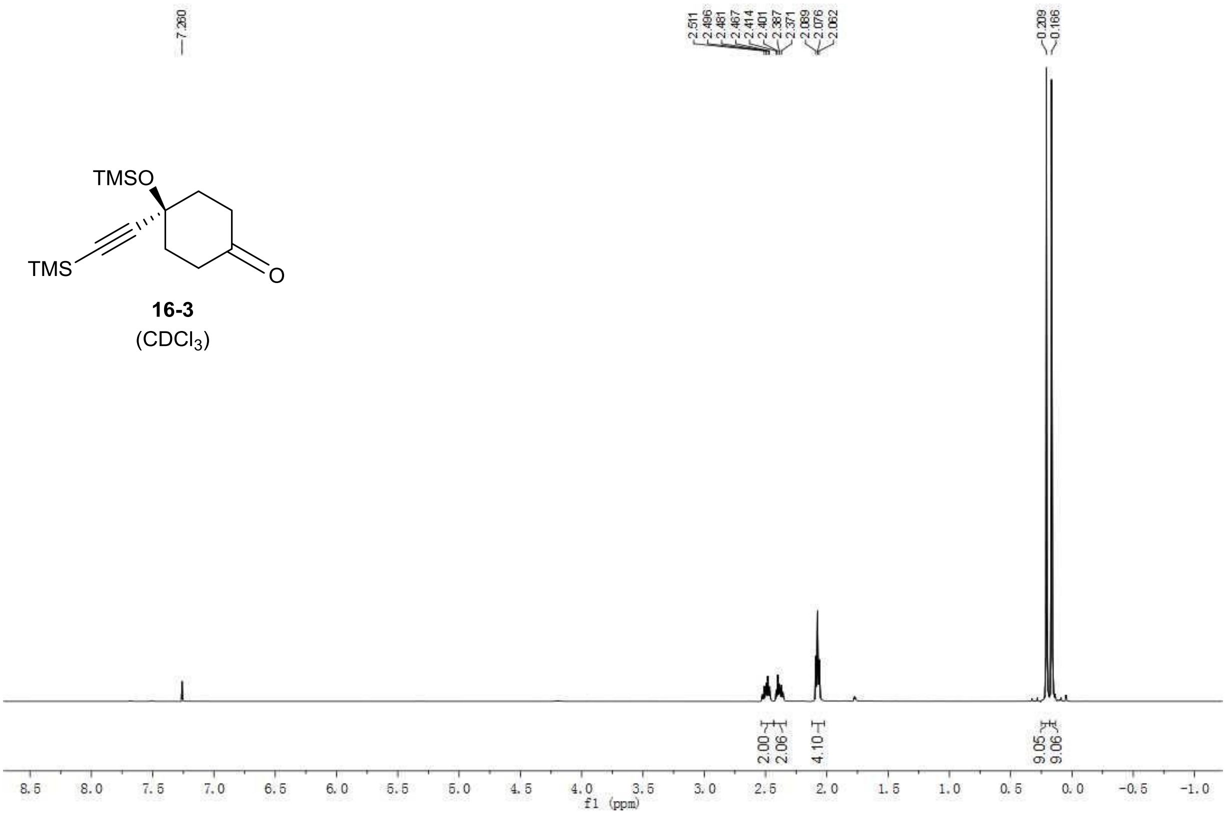

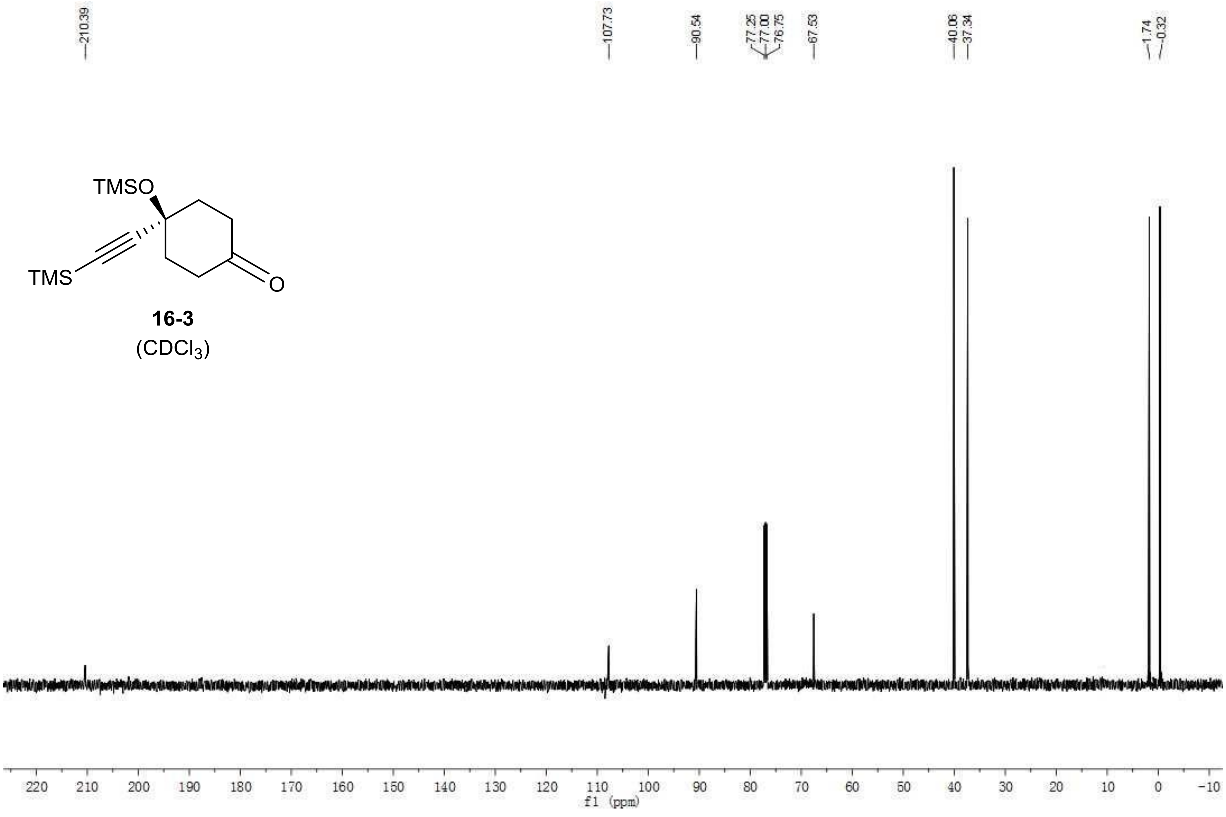

Supplementary Figure 36. ^{1}H and ^{13}C NMR spectra for 16-3.

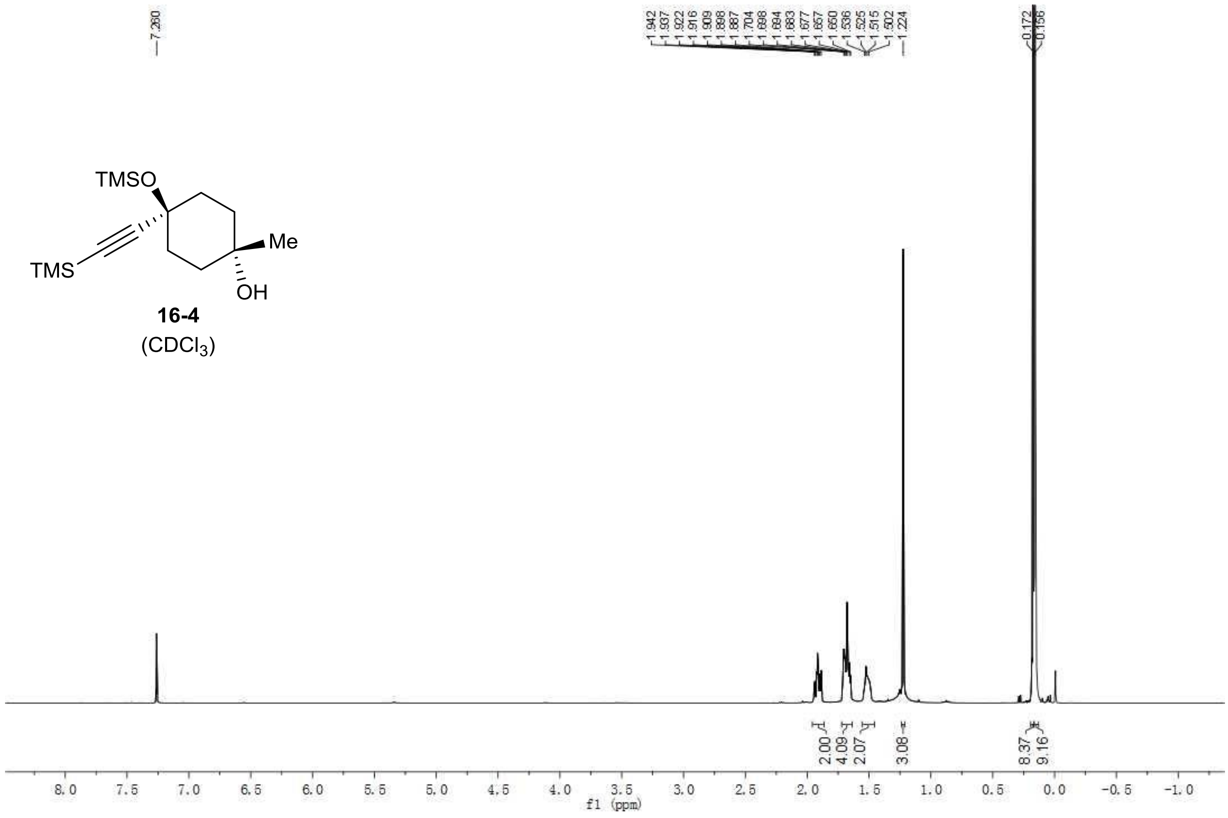

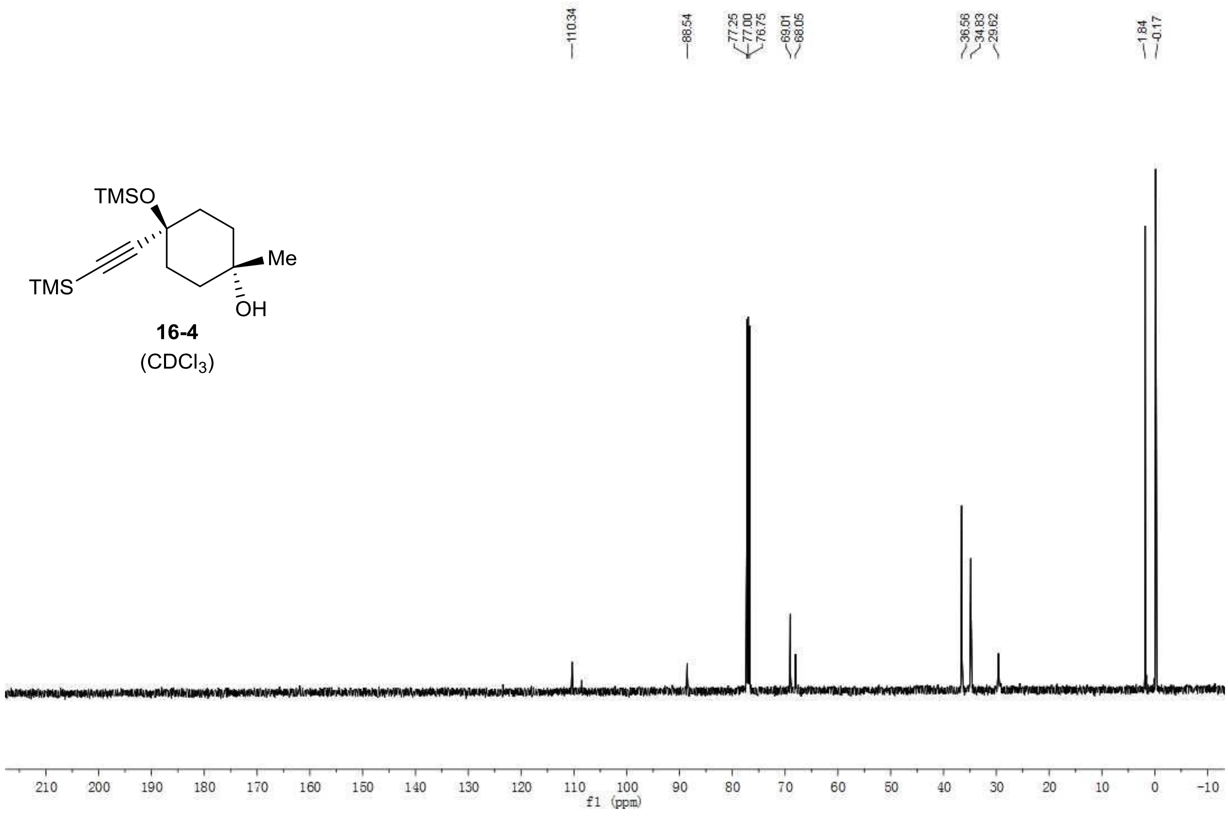

Supplementary Figure 37. ^{1}H and ^{13}C NMR spectra for 16-4.

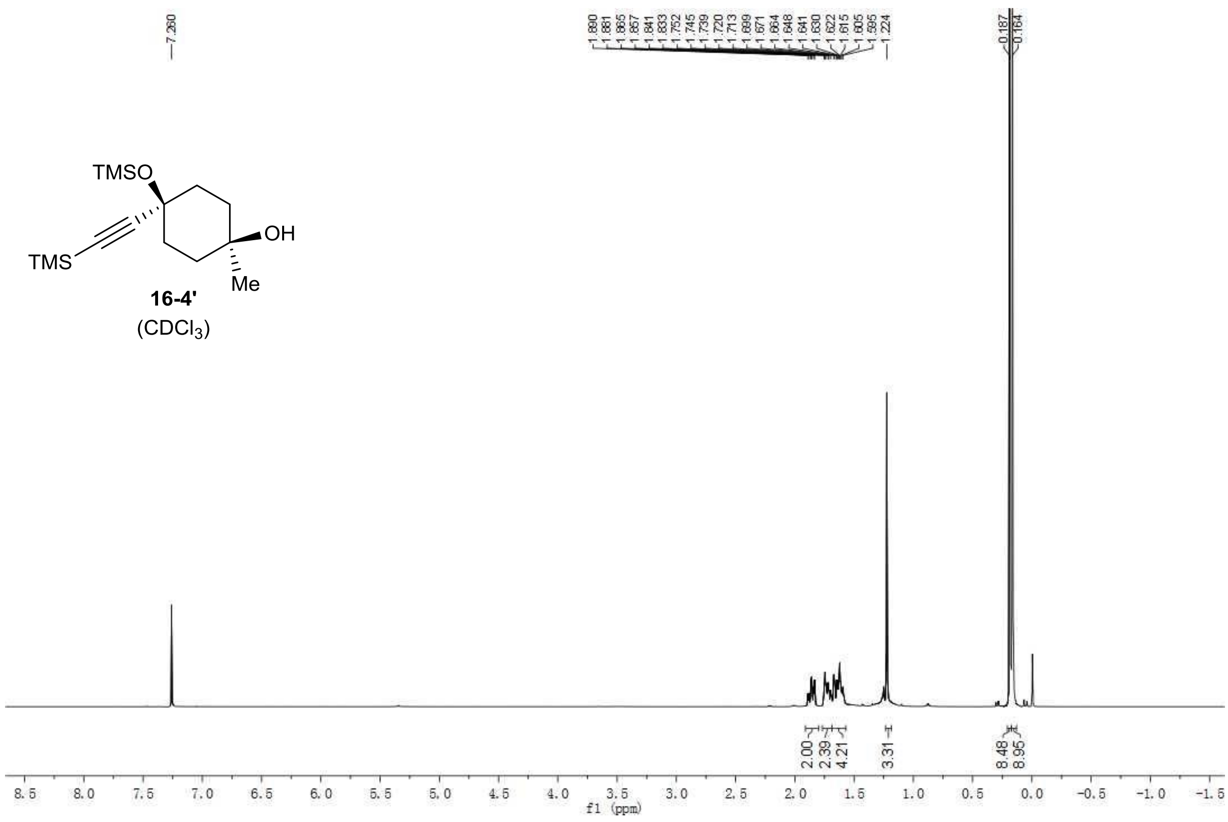

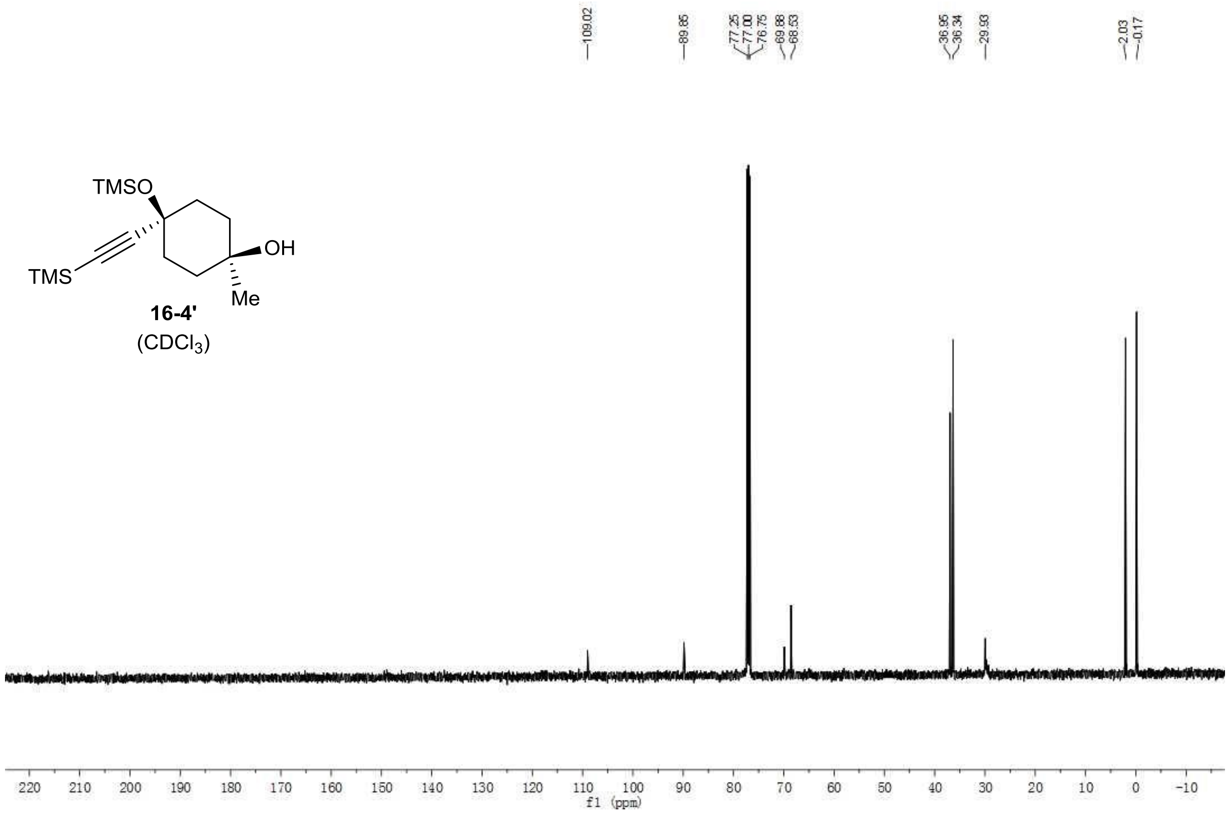

Supplementary Figure 38. ^{1}H and ^{13}C NMR spectra for 16-4’.

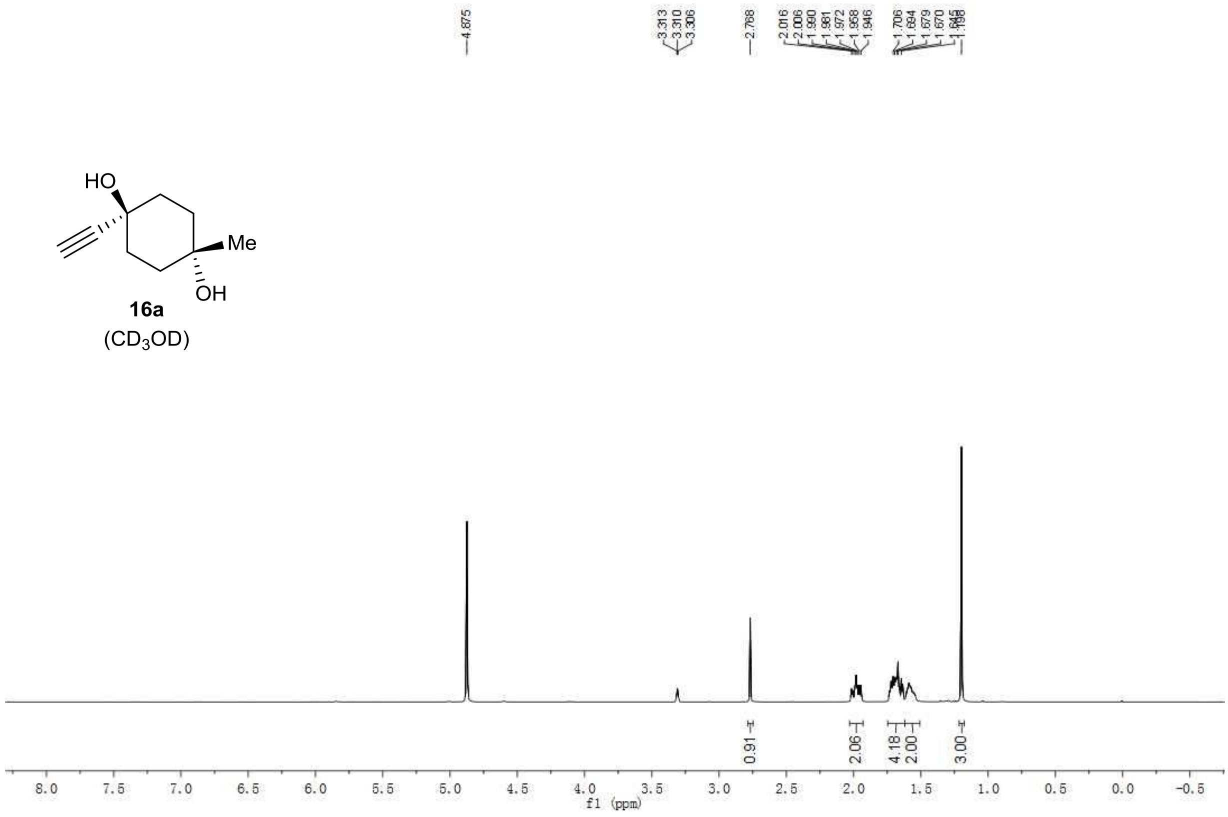

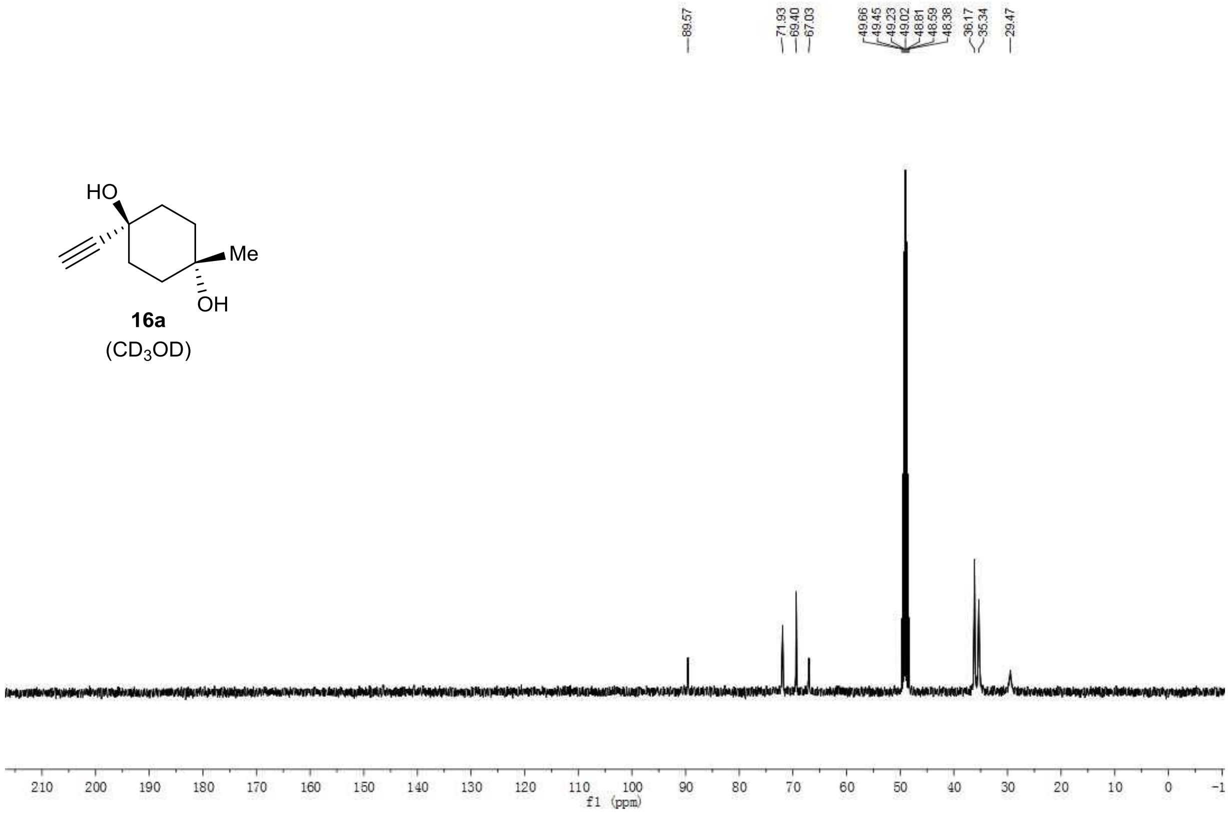

Supplementary Figure 39. ^{1}H and ^{13}C NMR spectra for 16a.

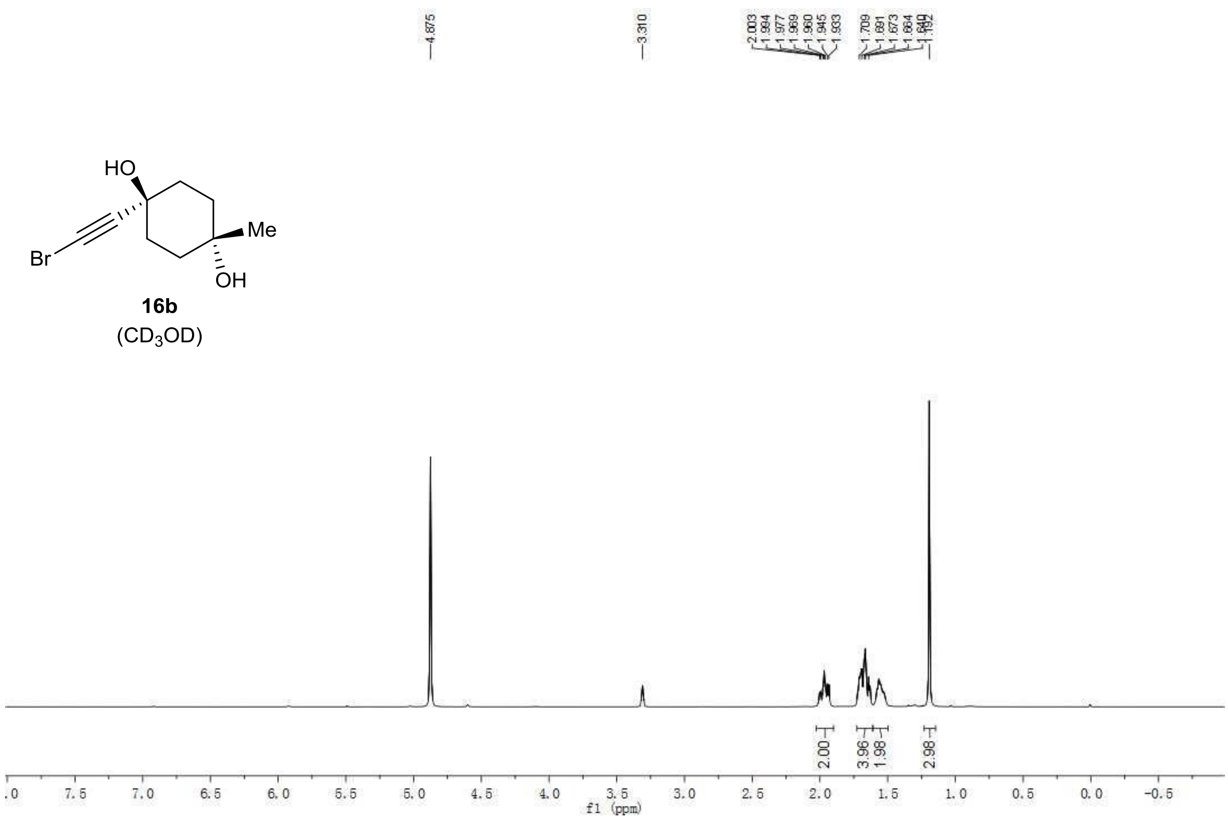

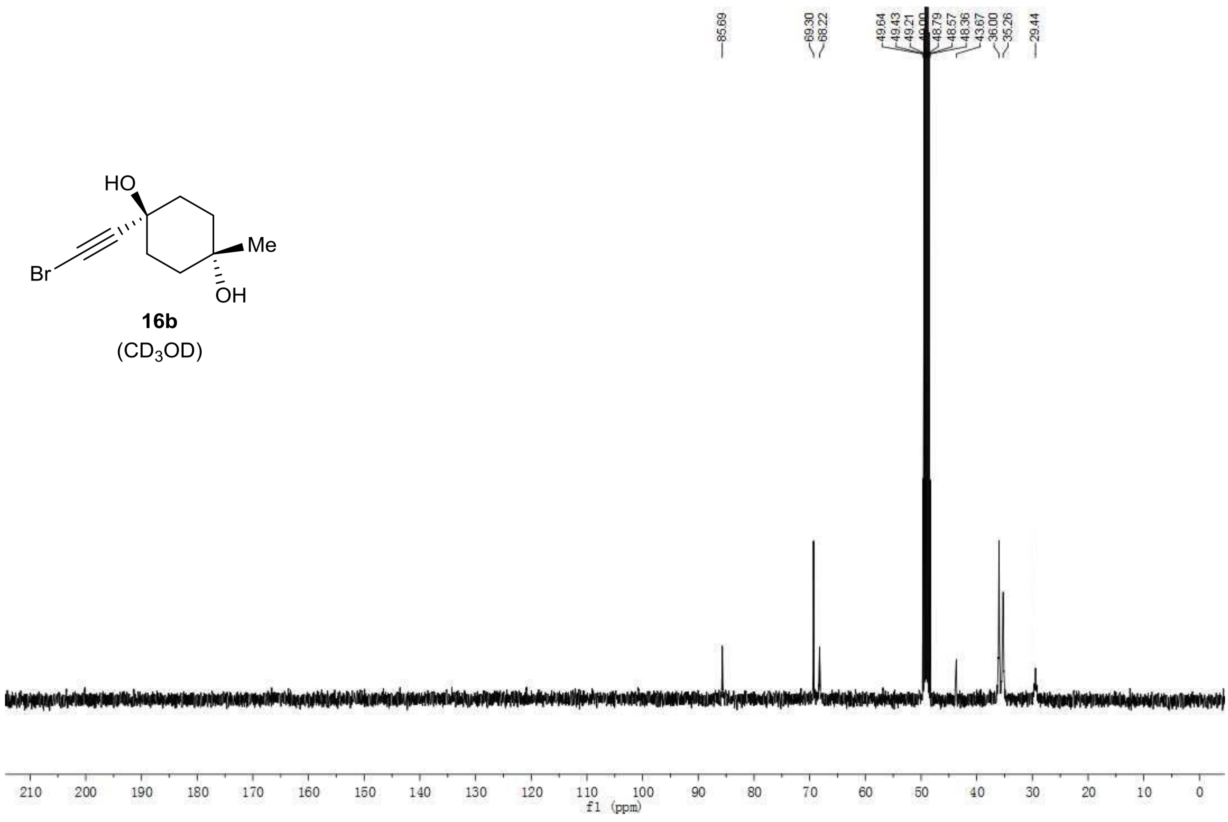

Supplementary Figure 40. ^{1}H and ^{13}C NMR spectra for 16b.

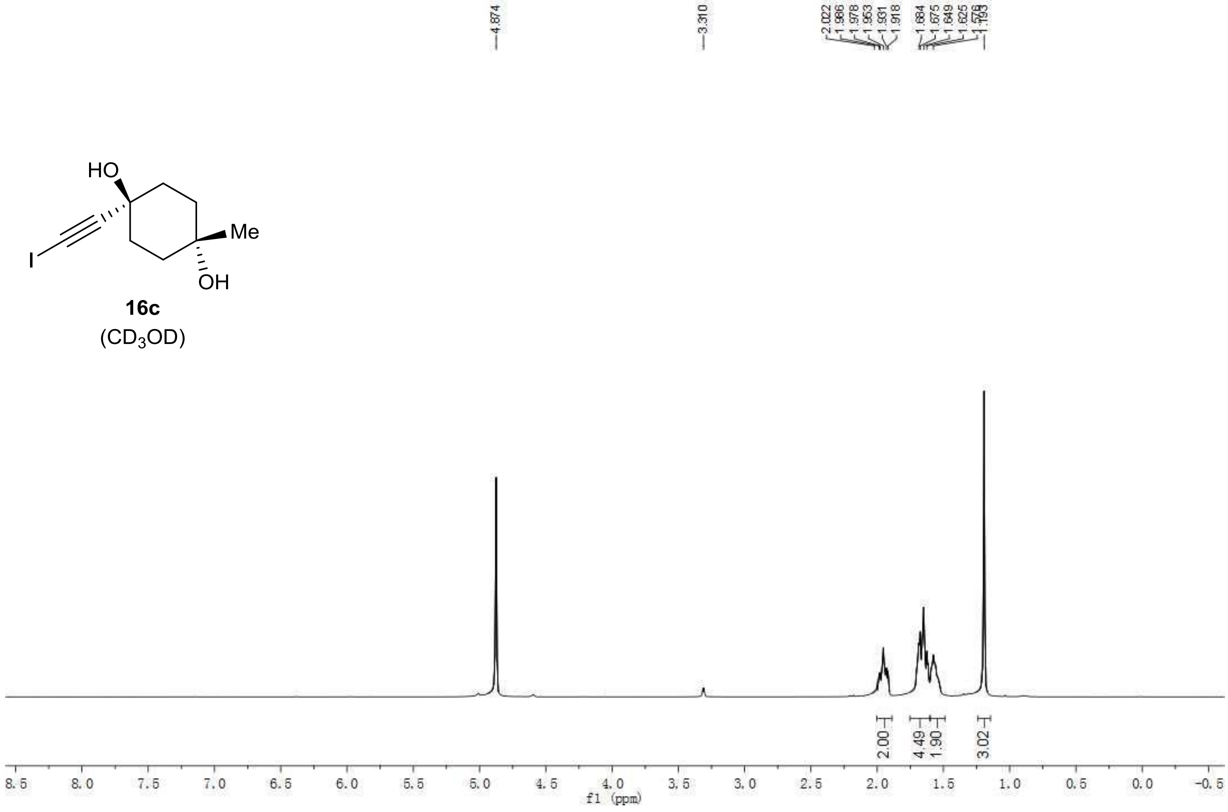

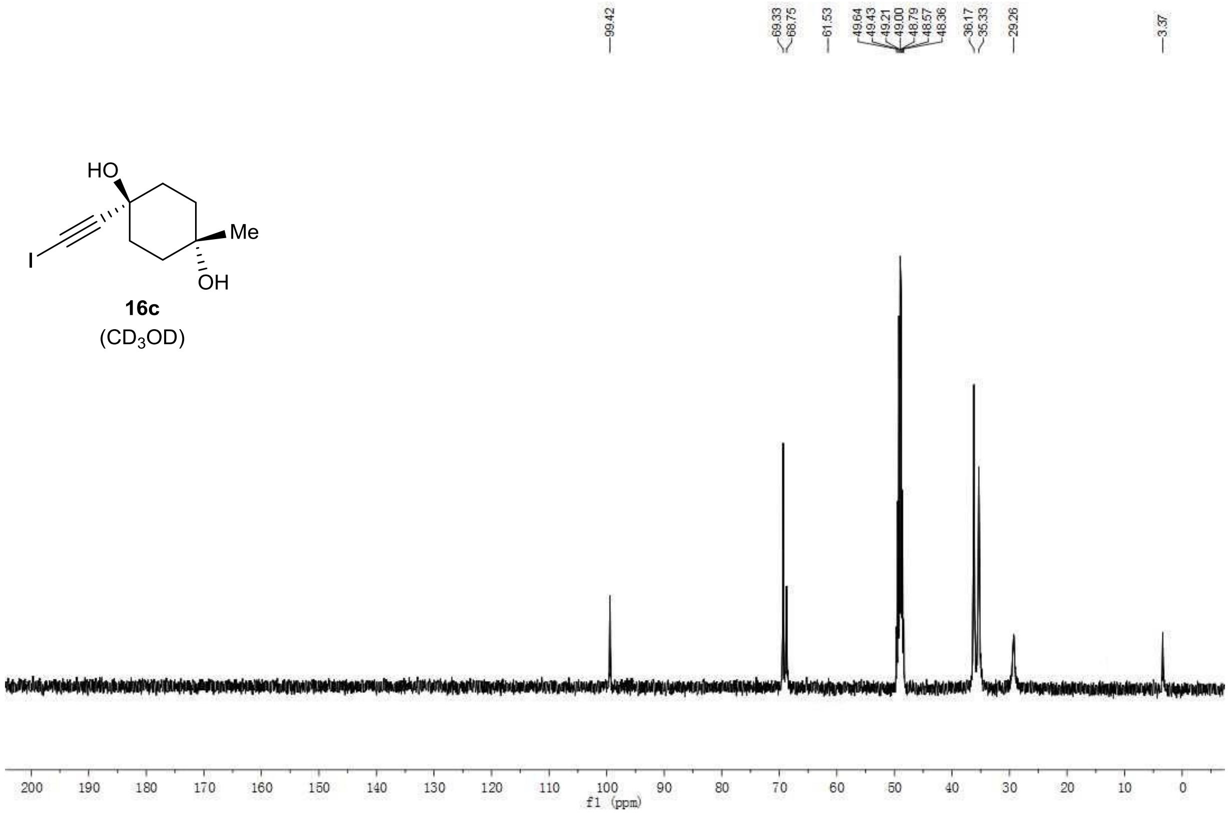

Supplementary Figure 41. ^{1}H and ^{13}C NMR spectra for 16c.

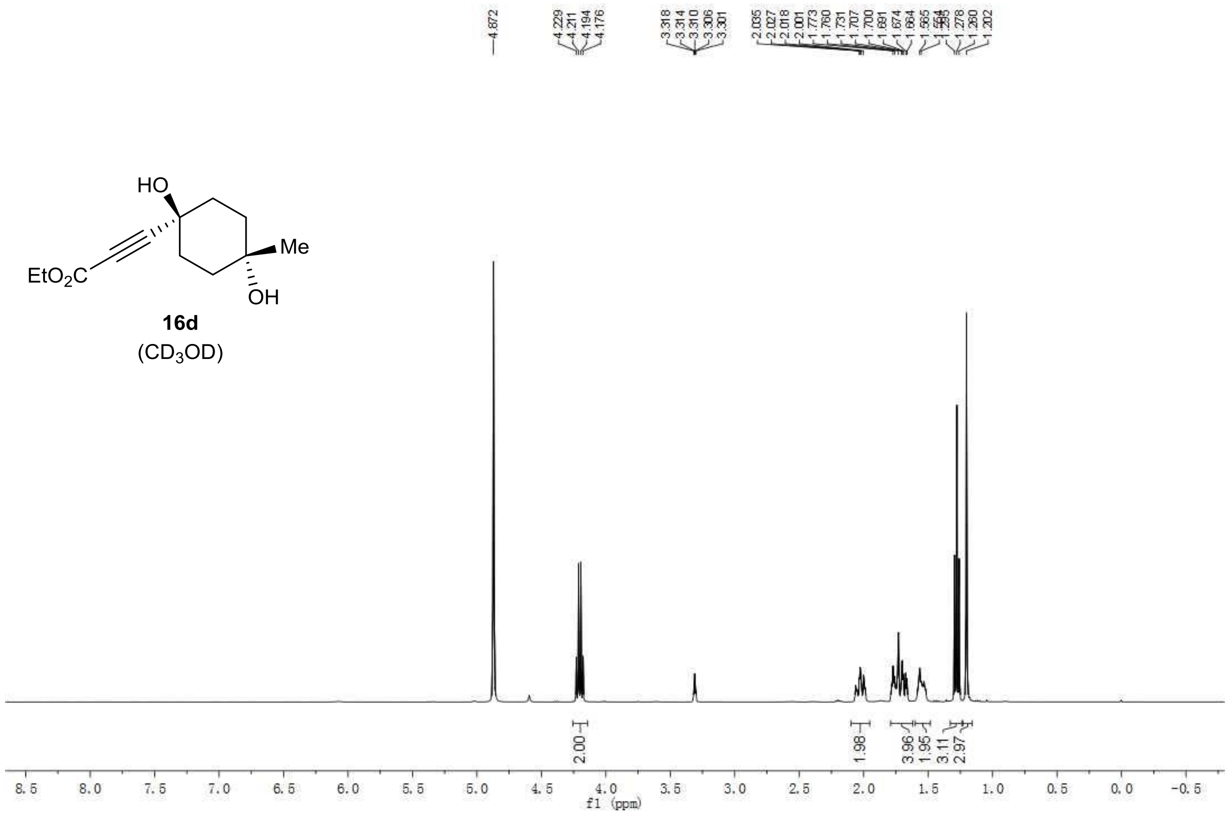

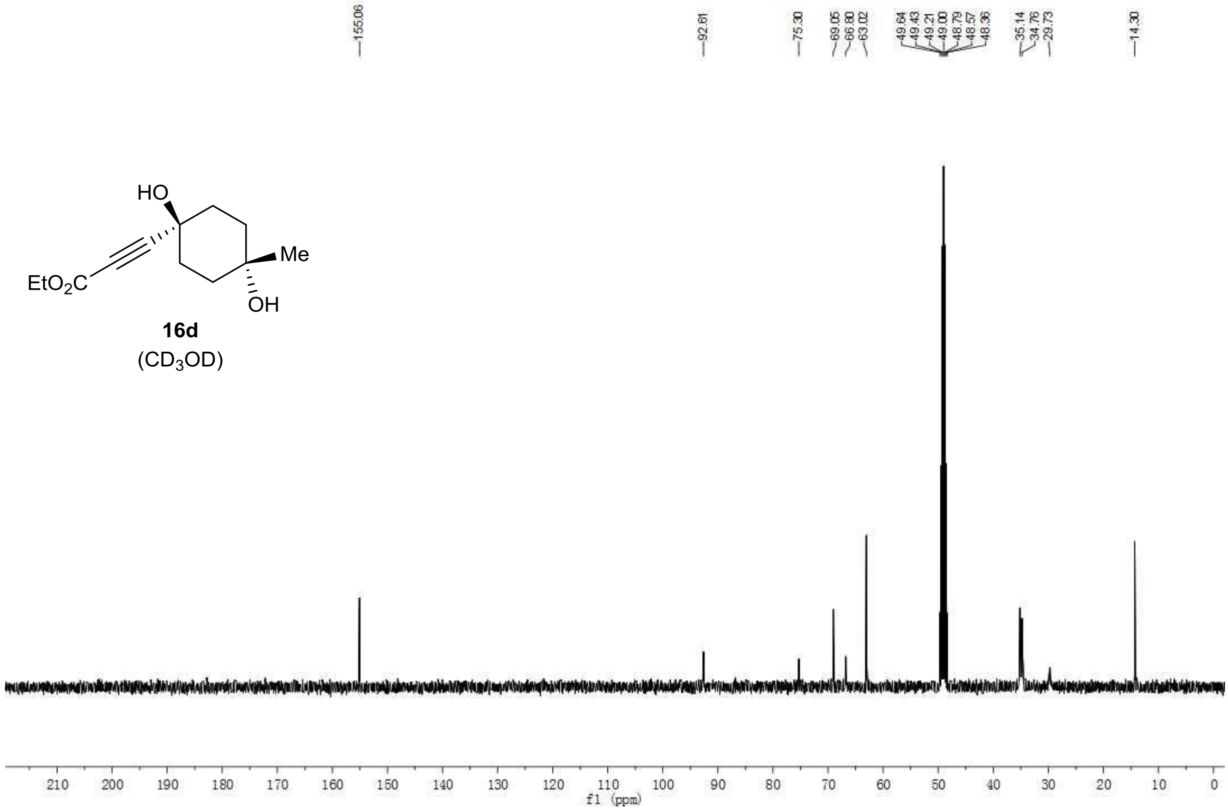

Supplementary Figure 42. ^{1}H and ^{13}C NMR spectra for 16d.

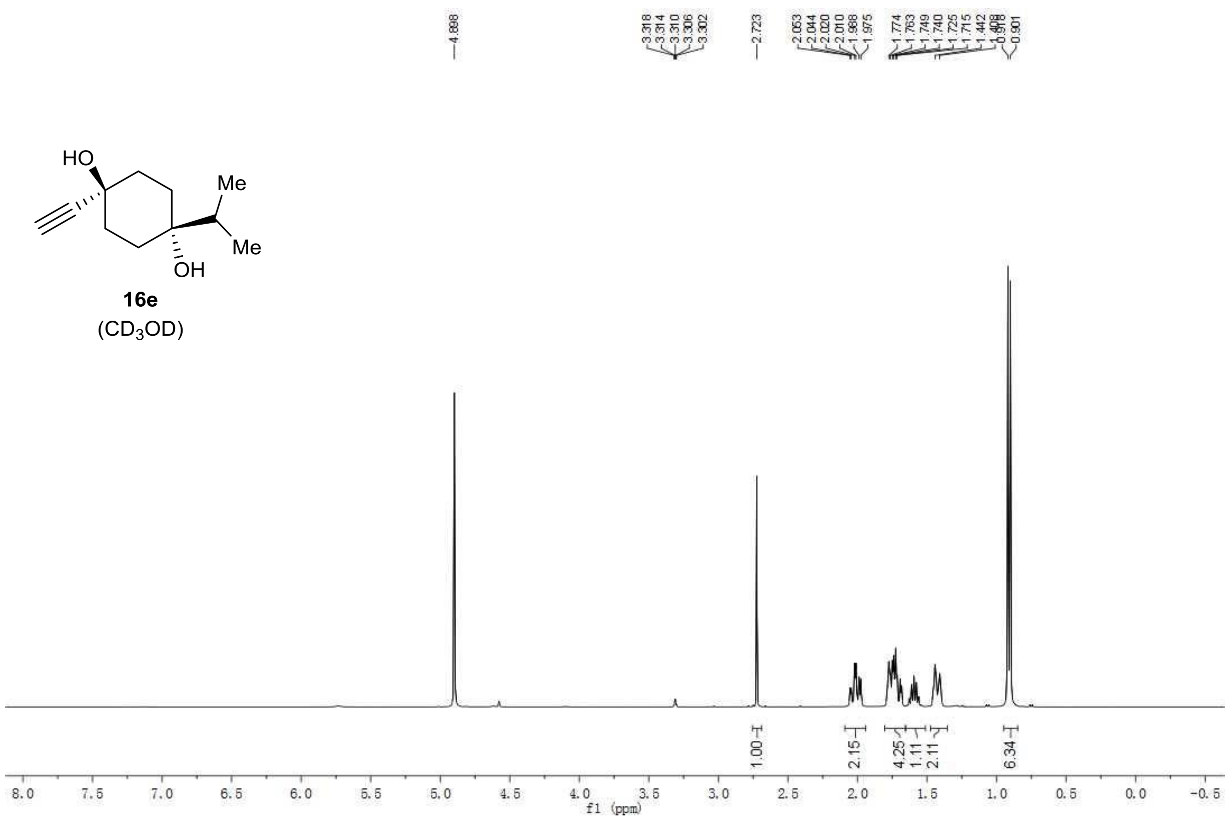

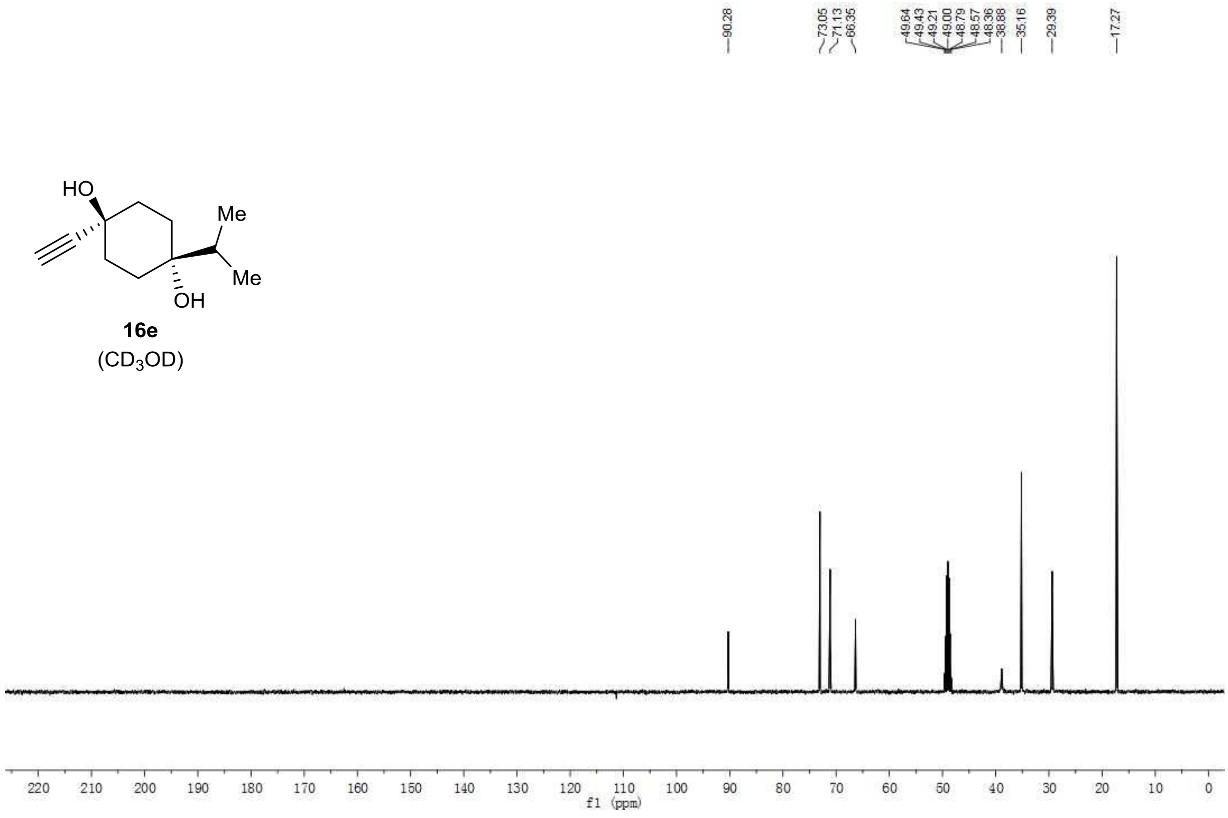

Supplementary Figure 43. ^{1}**H and** ^{13}**C NMR spectra for 16e.**

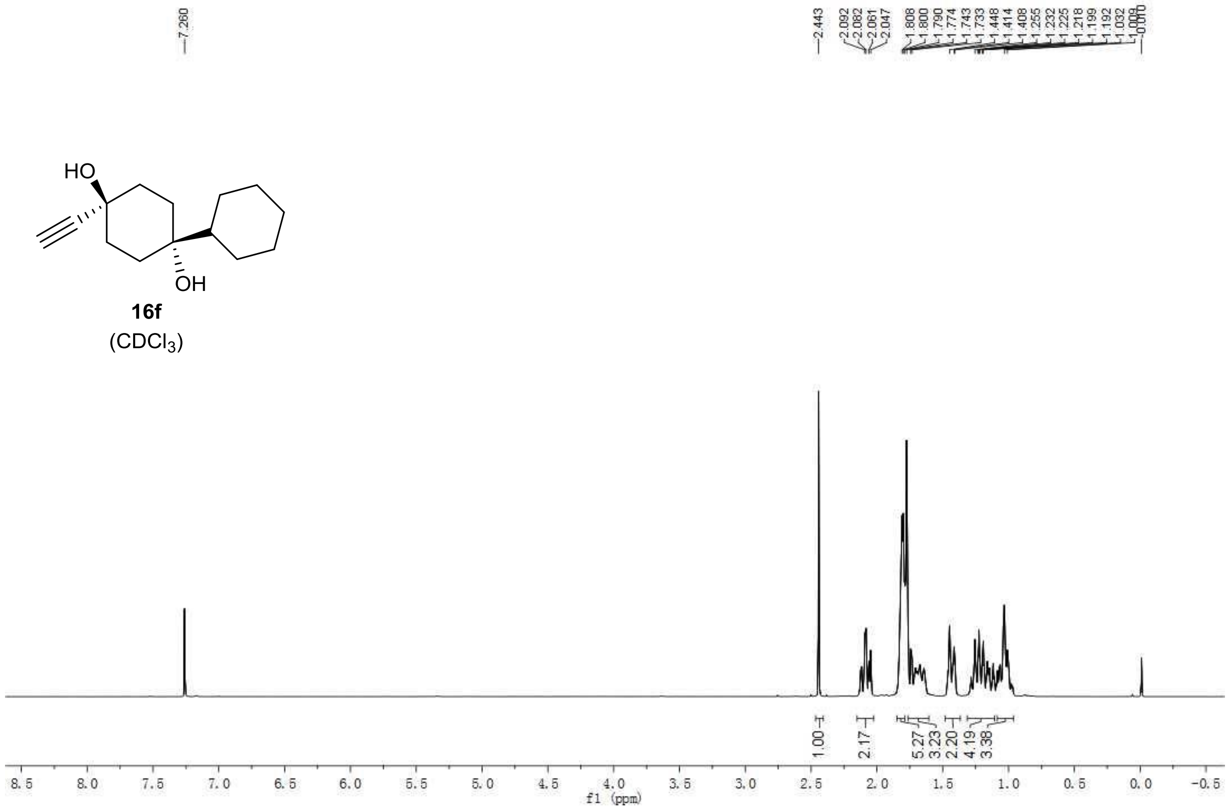

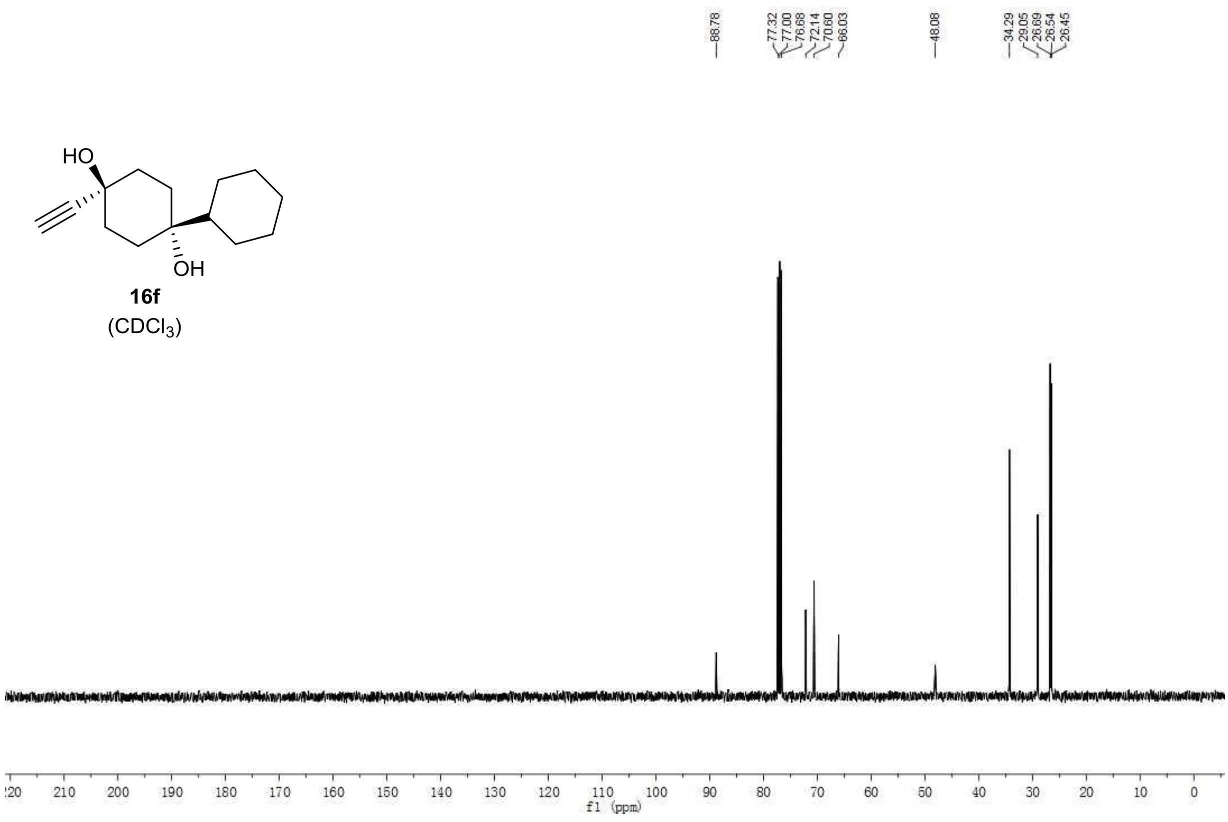

Supplementary Figure 44. ^{1}H and ^{13}C NMR spectra for 16f.

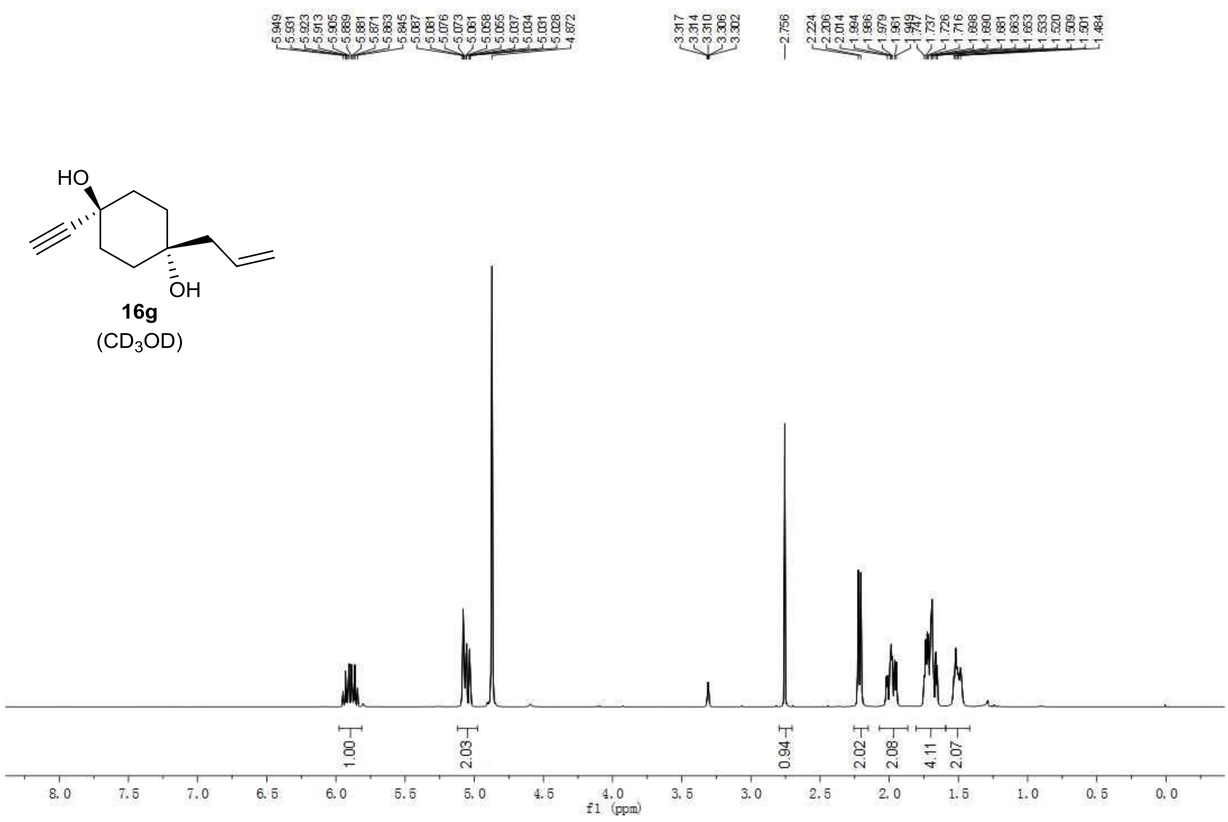

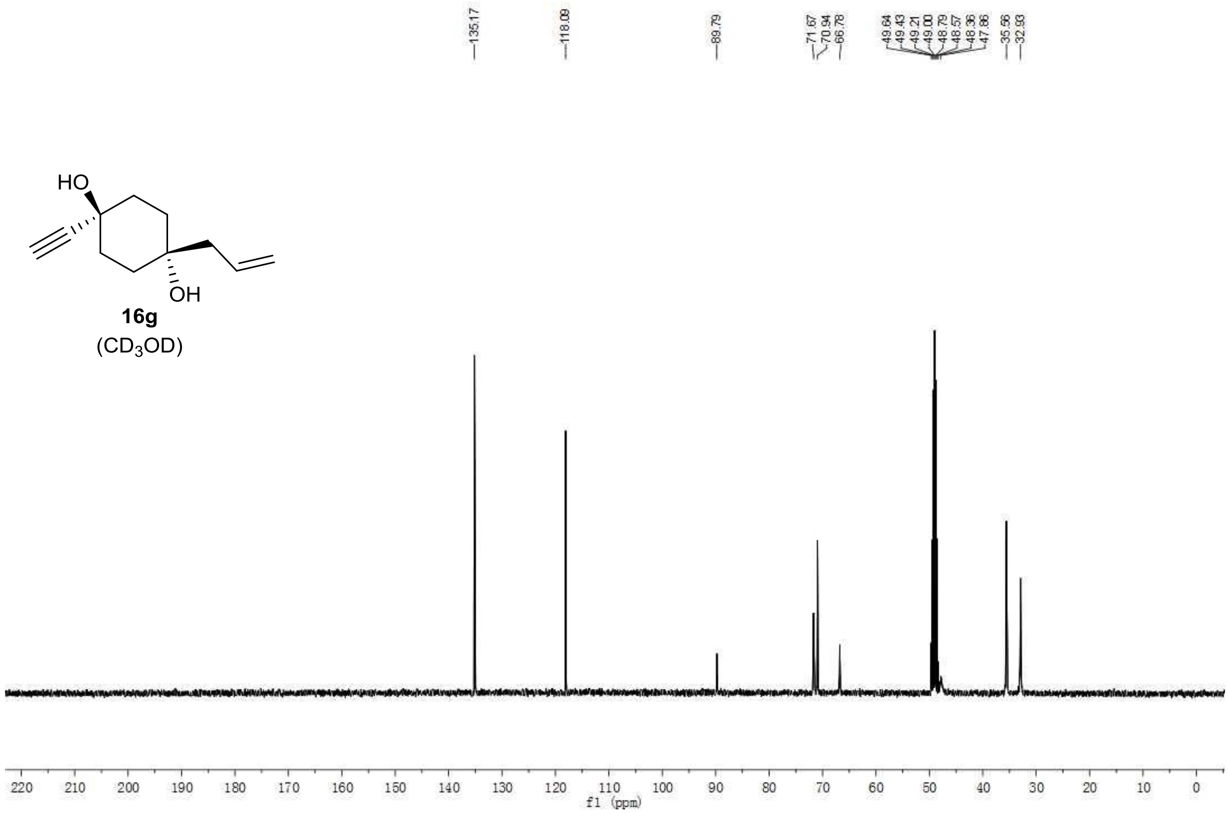

Supplementary Figure 45. ^{1}H and ^{13}C NMR spectra for 16g.

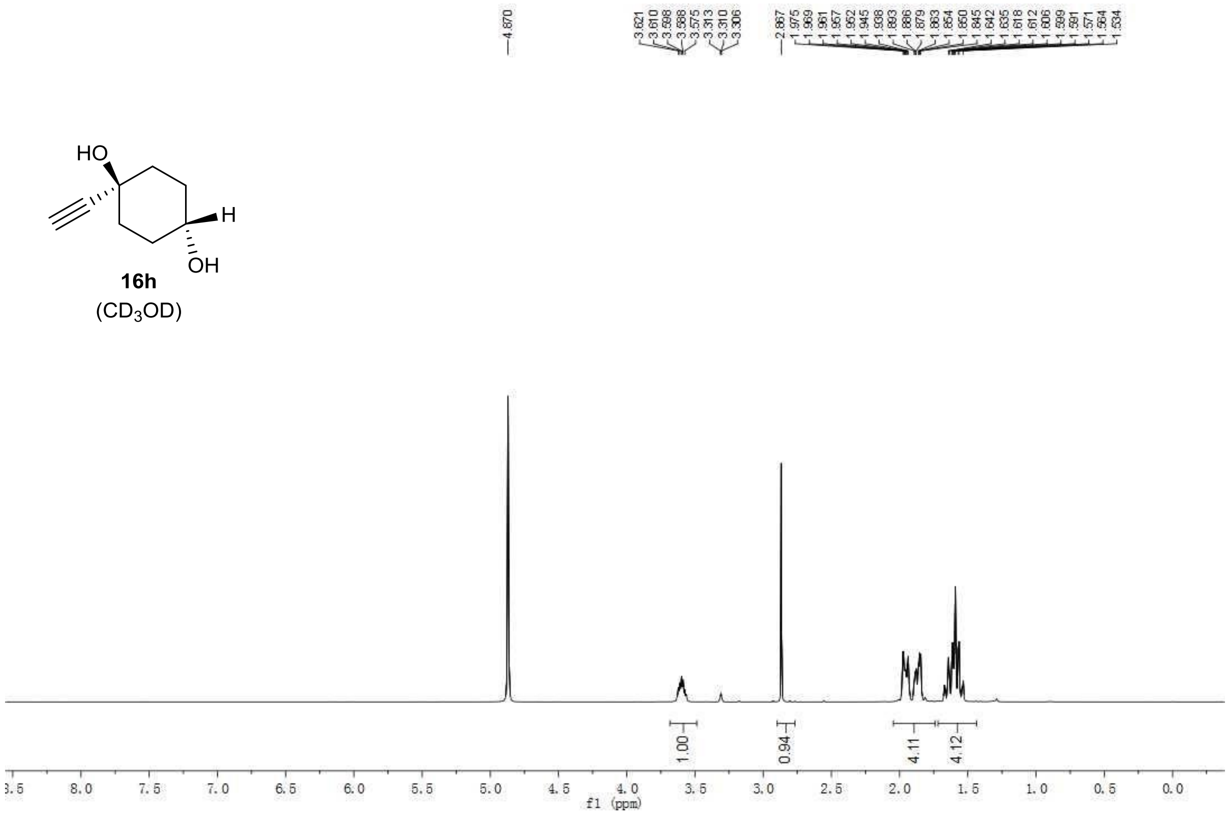

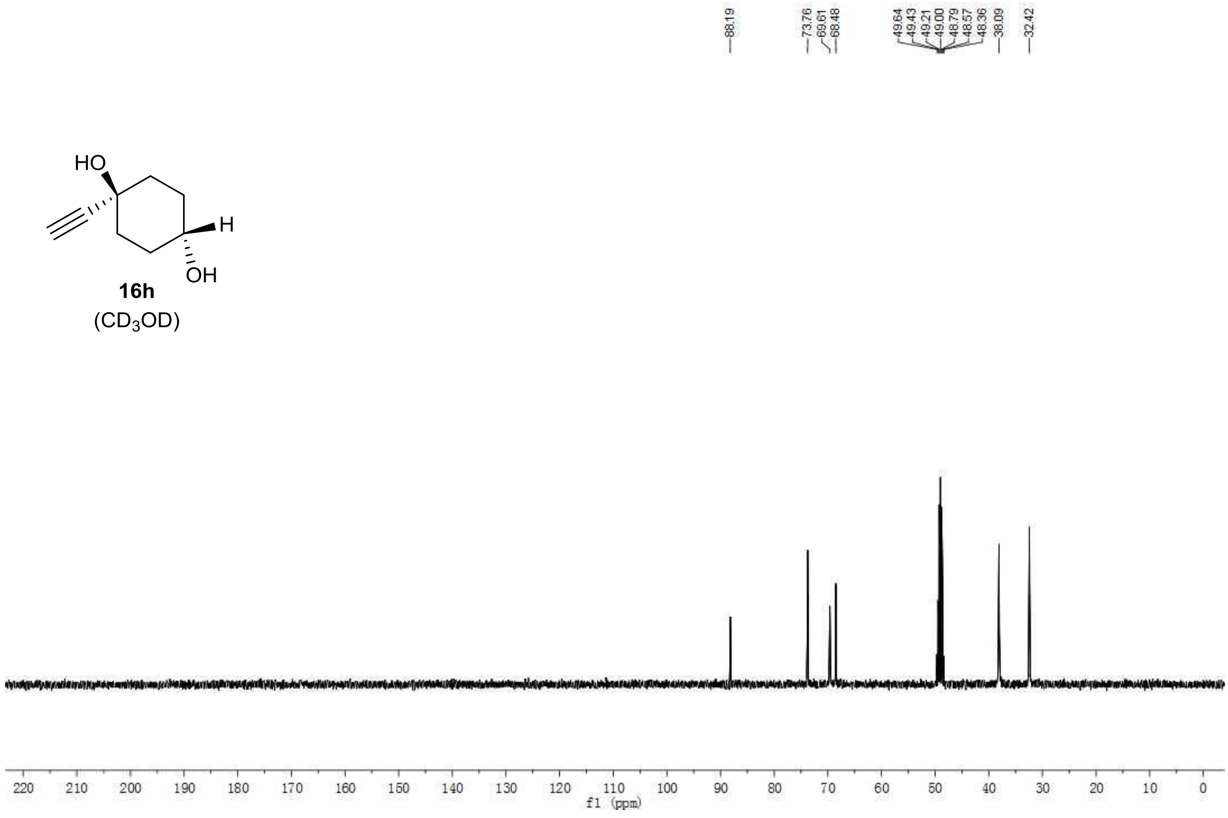

Supplementary Figure 46. ^{1}H and ^{13}C NMR spectra for 16h.

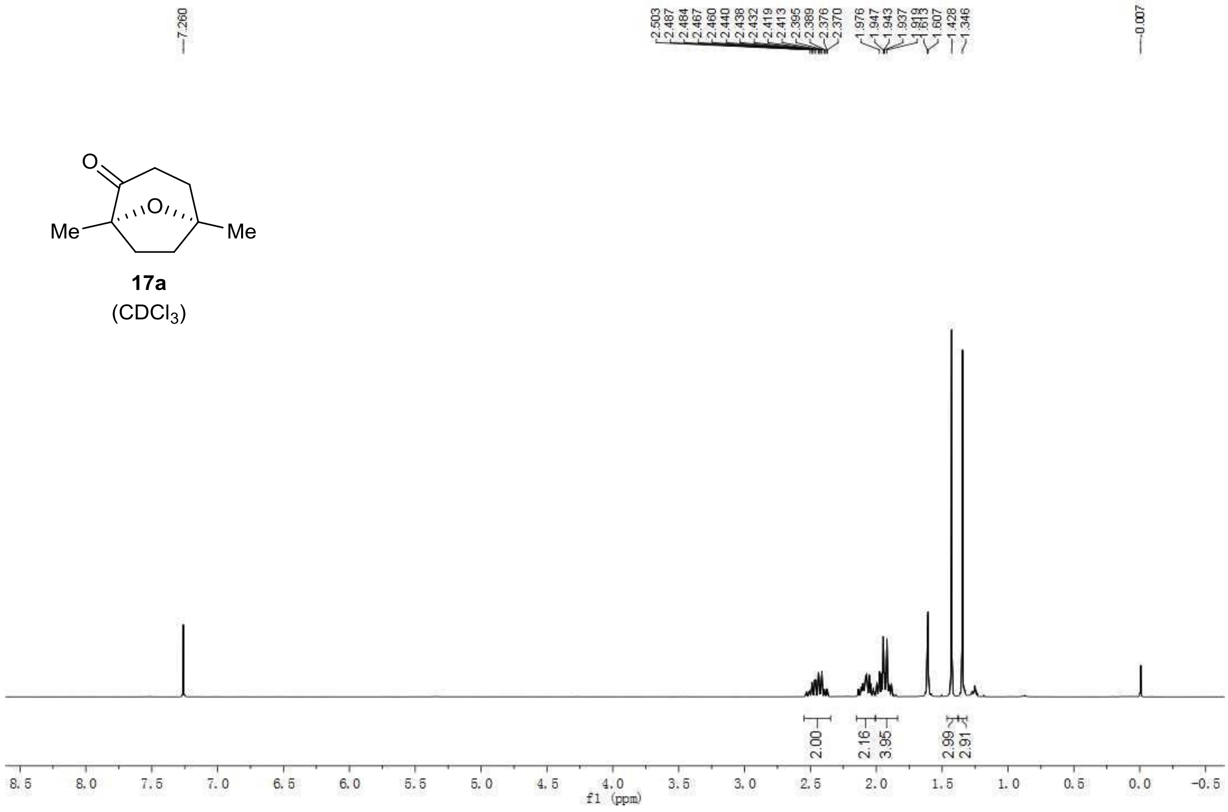

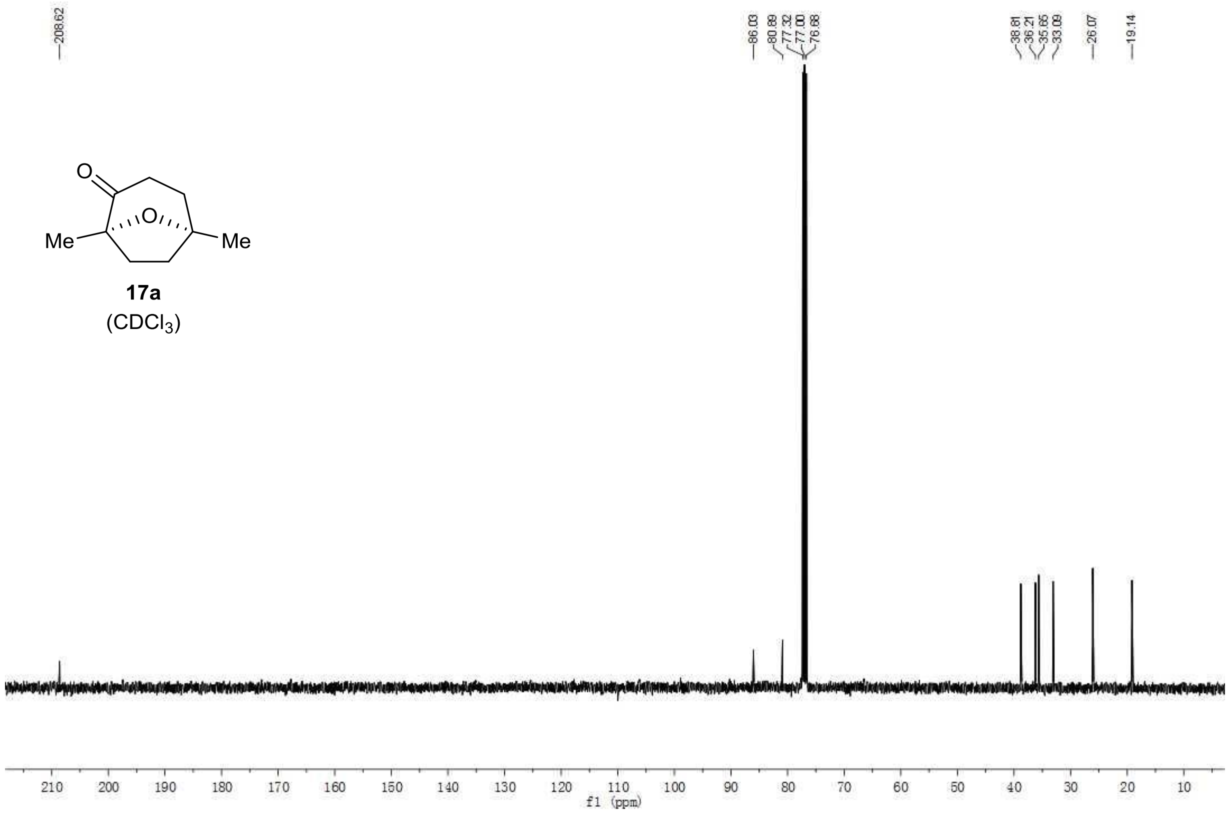

Supplementary Figure 47. ^{1}H and ^{13}C NMR spectra for 17a.

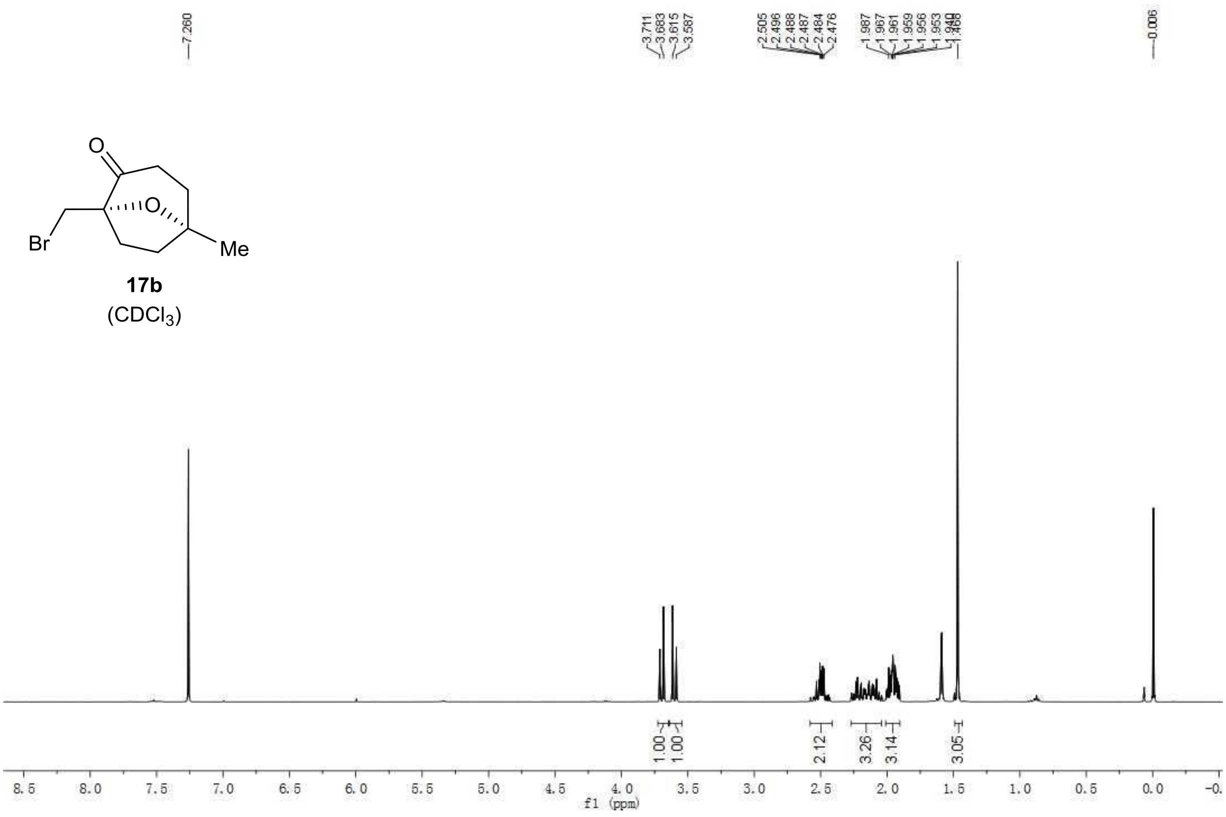

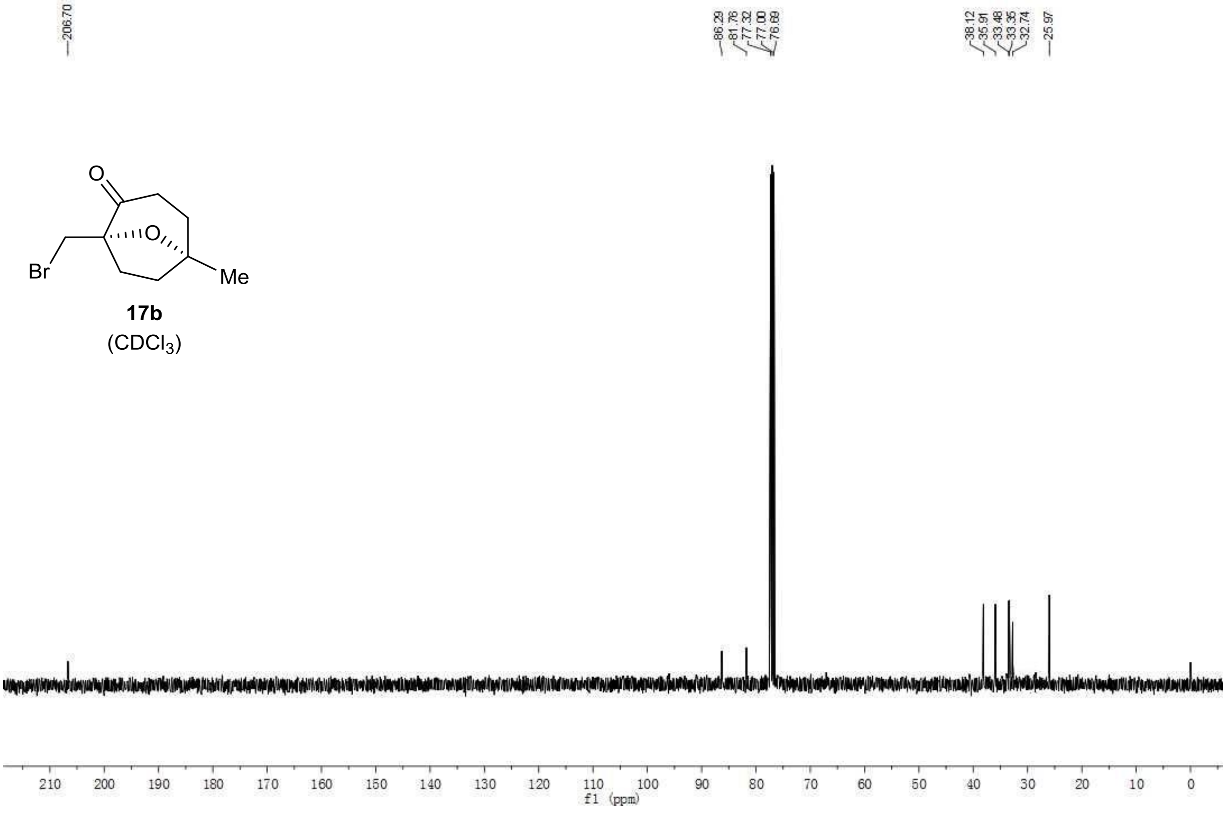

Supplementary Figure 48. ^{1}H and ^{13}C NMR spectra for 17b.

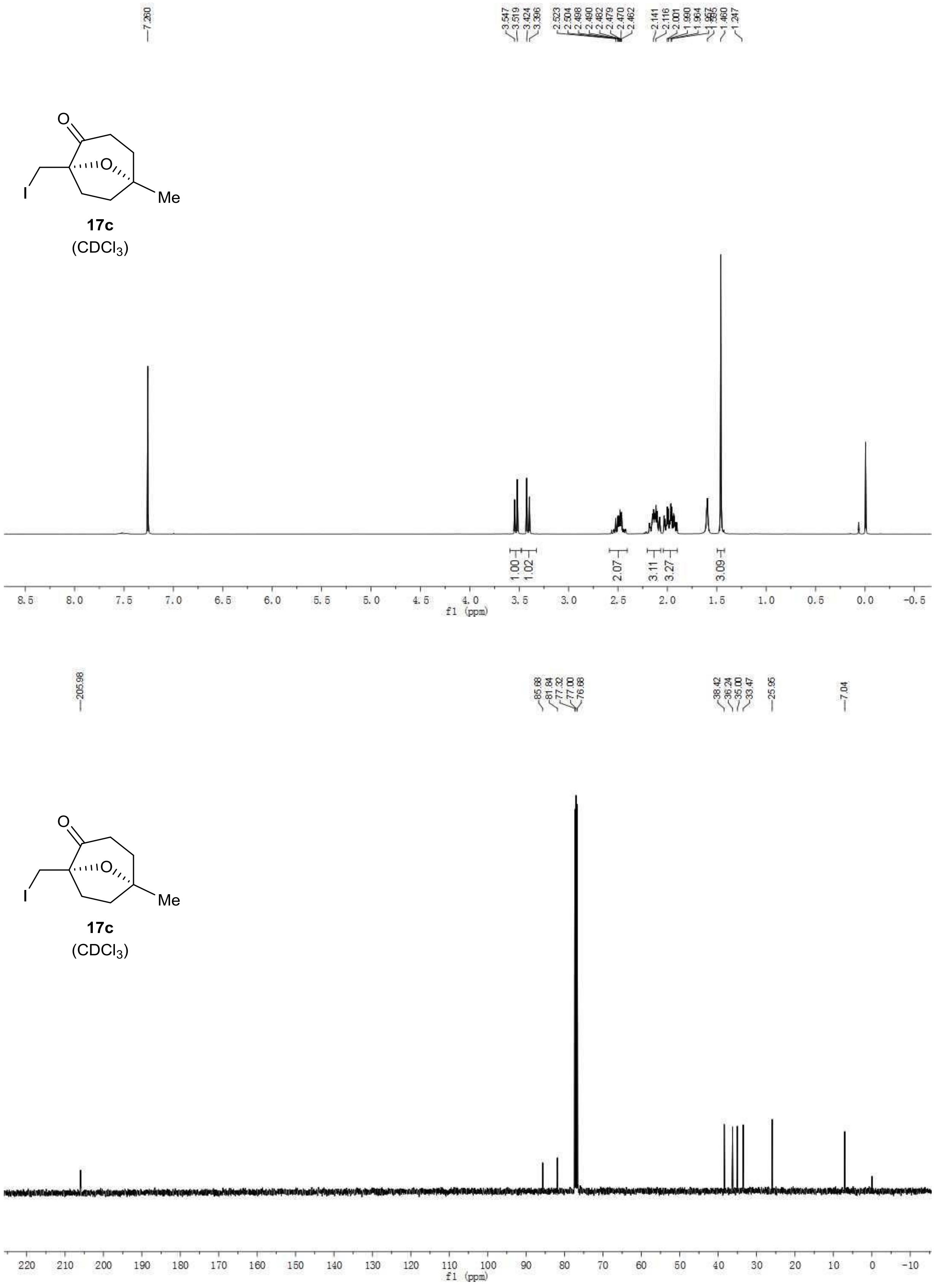

Supplementary Figure 49. ^{1}**H and** ^{13}**C NMR spectra for 17c.**

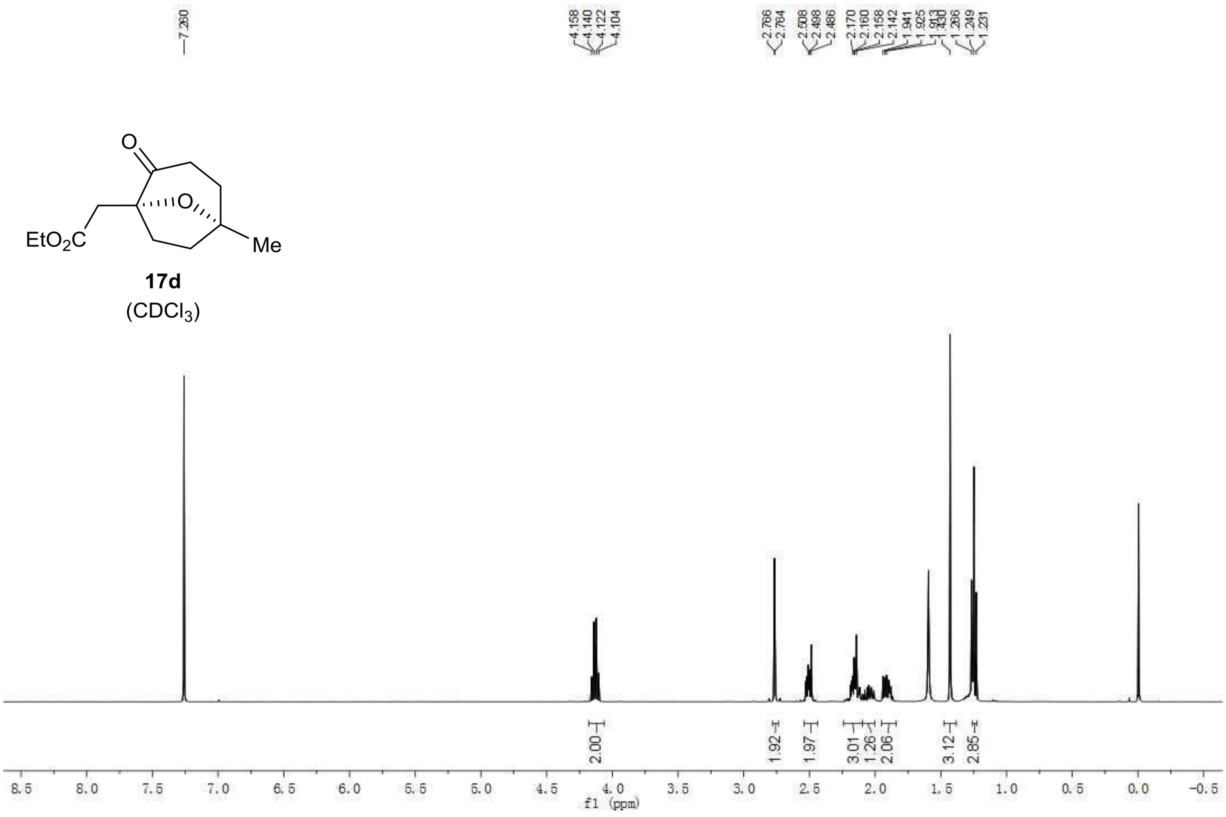

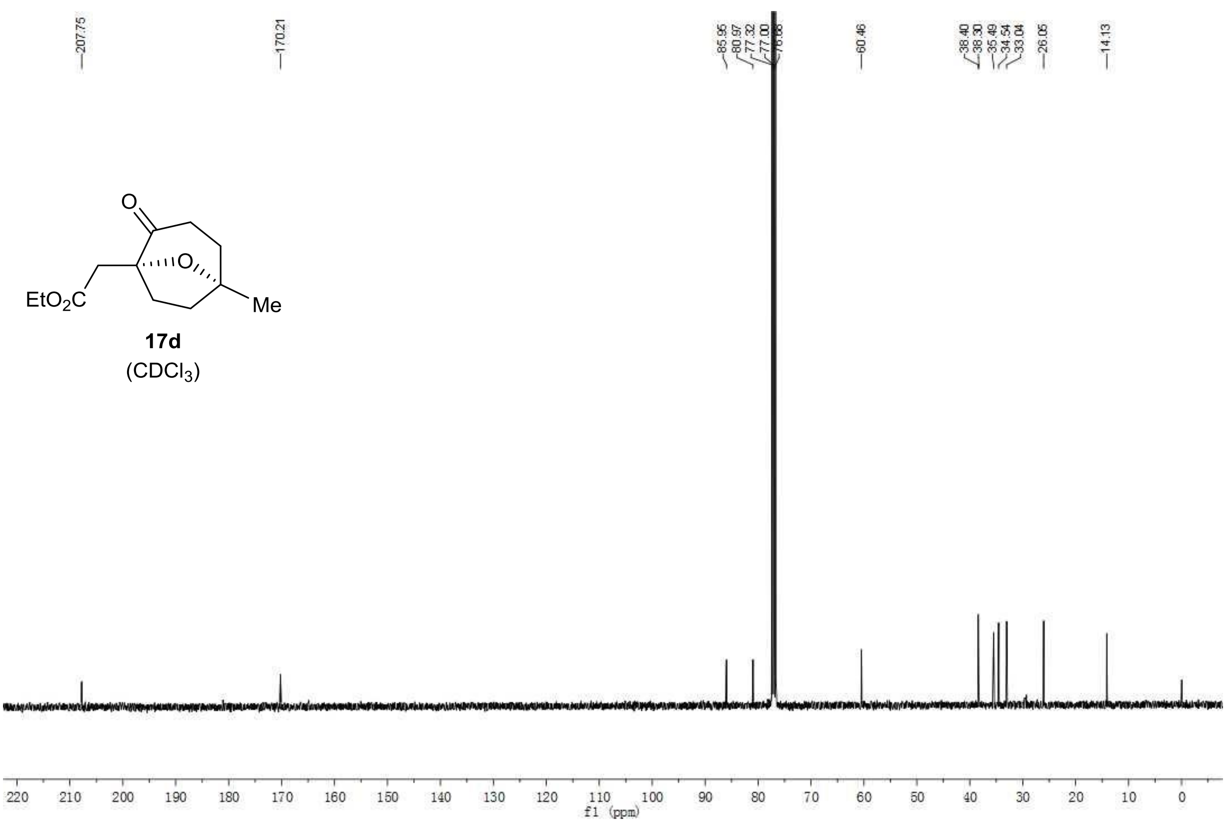

Supplementary Figure 50. ^{1}H and ^{13}C NMR spectra for 17d.

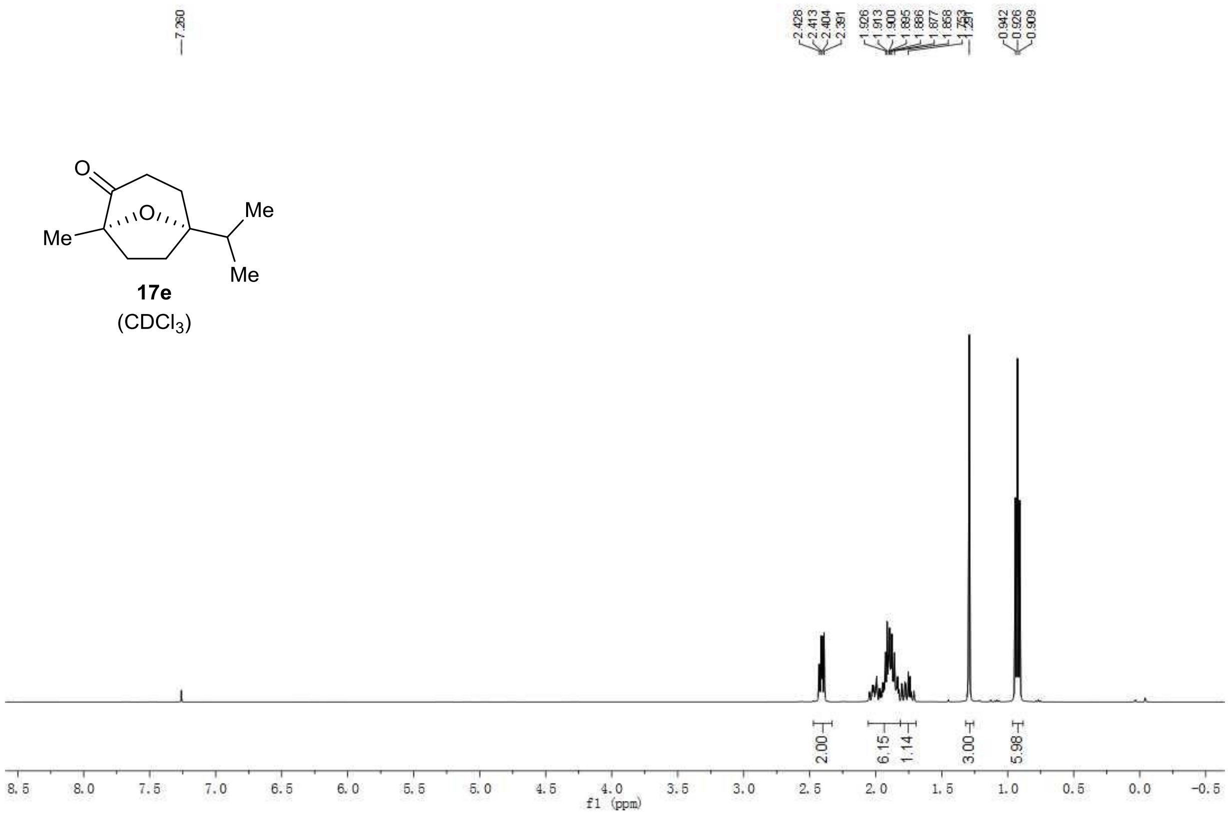

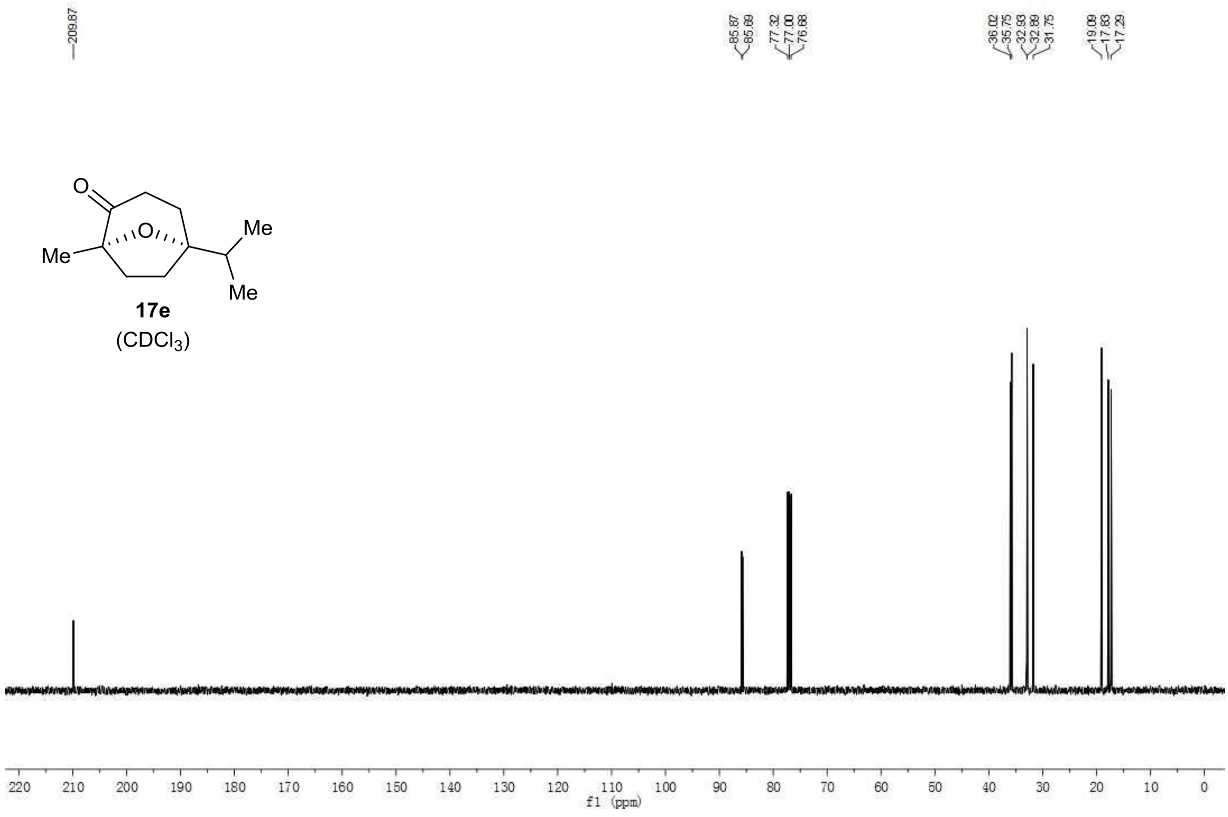

Supplementary Figure 51. ^{1}H and ^{13}C NMR spectra for 17e.

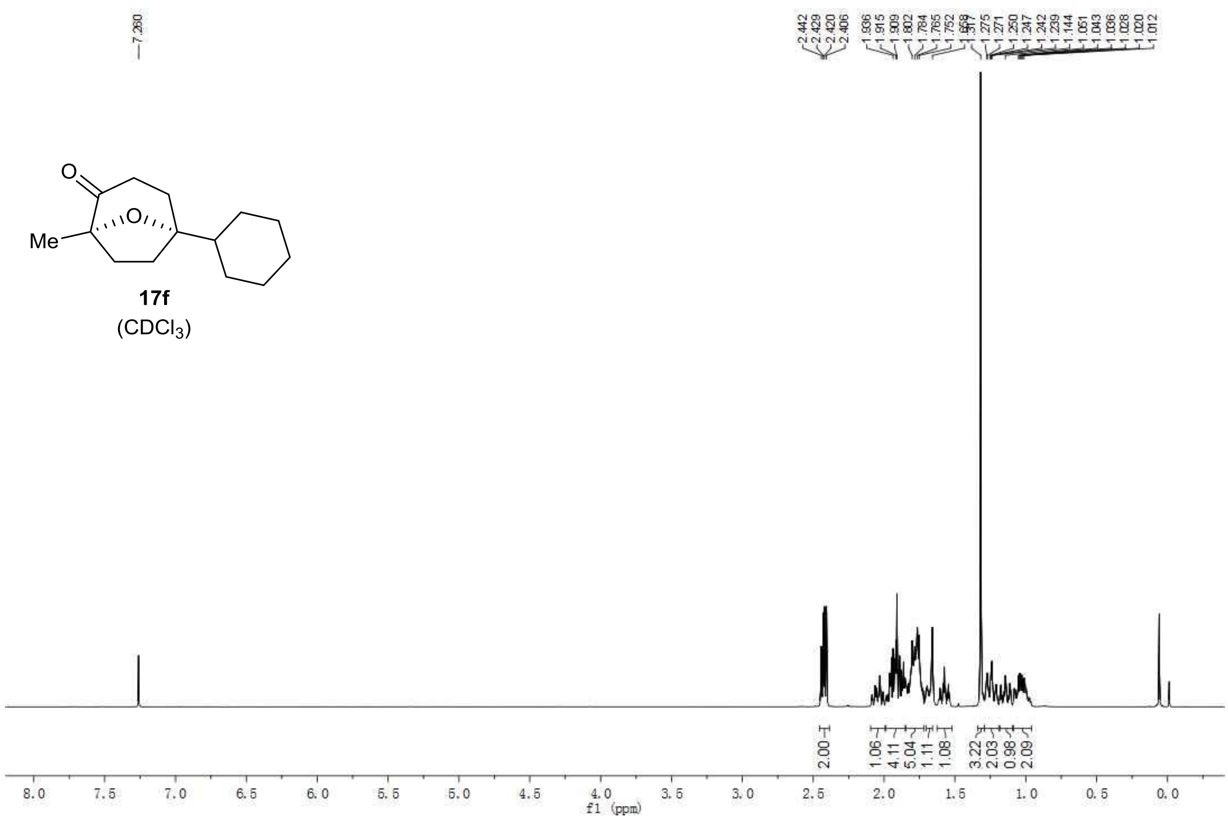

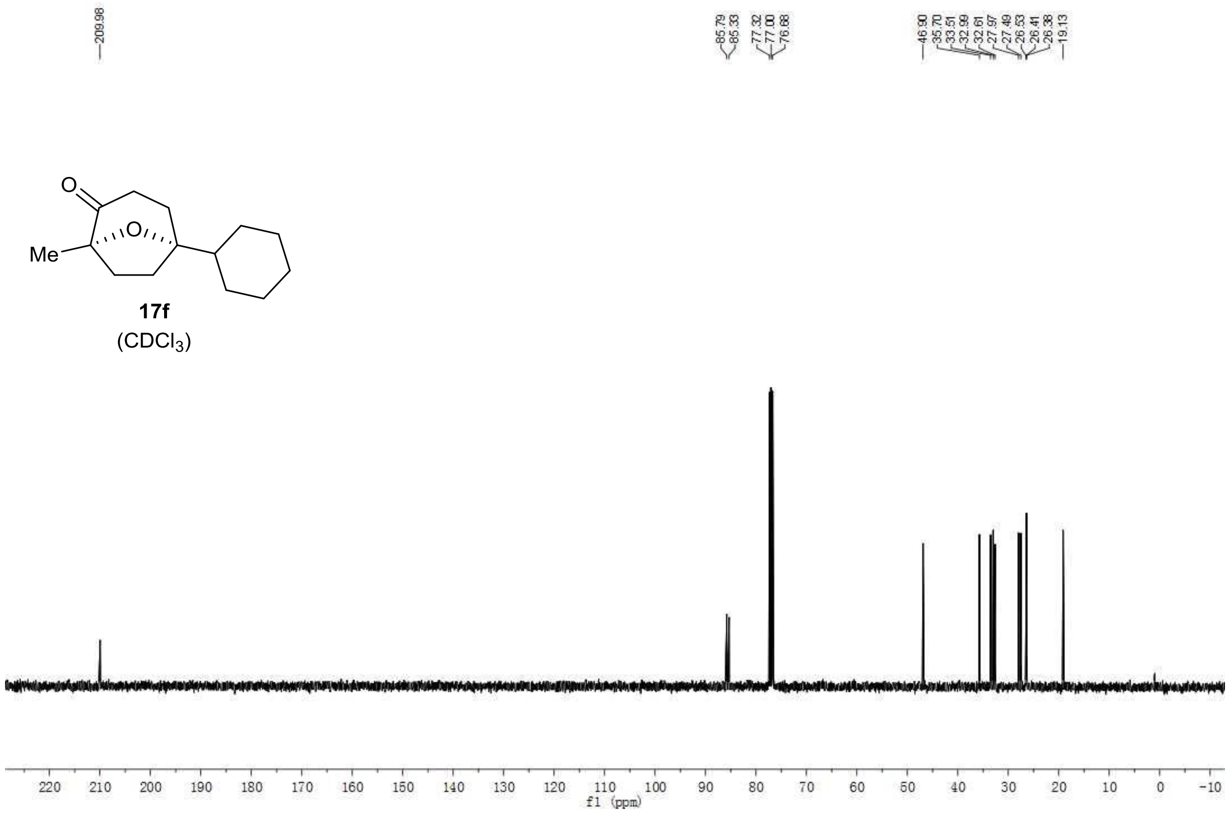

Supplementary Figure 52. ^{1}H and ^{13}C NMR spectra for 17f.

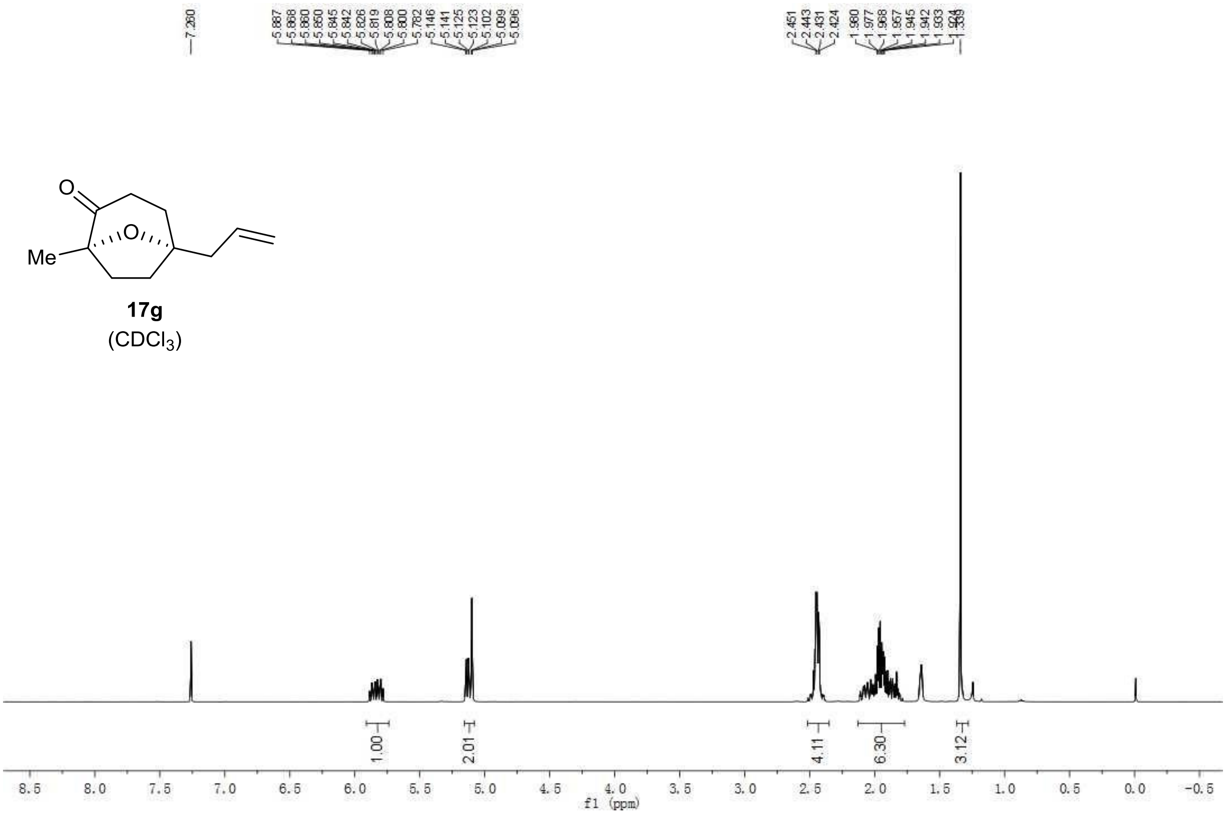

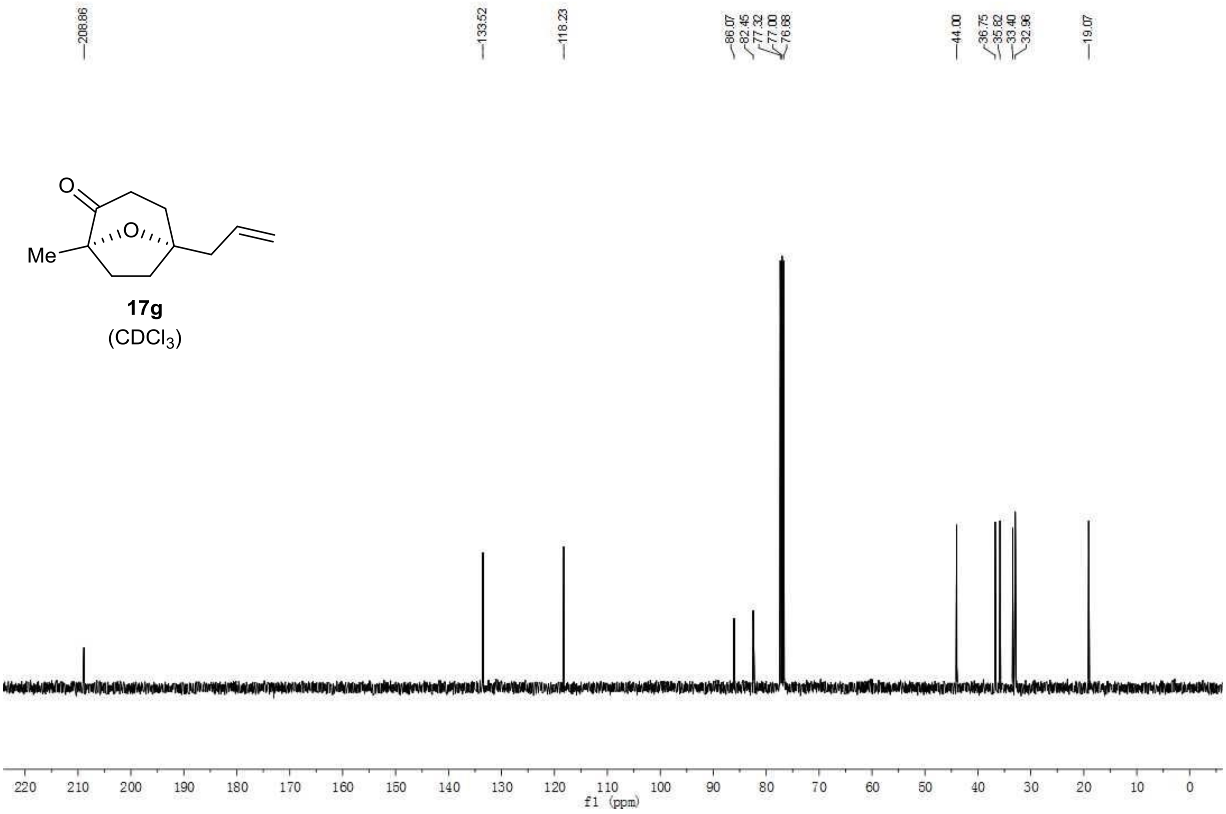

Supplementary Figure 53. ^{1}H and ^{13}C NMR spectra for 17g.

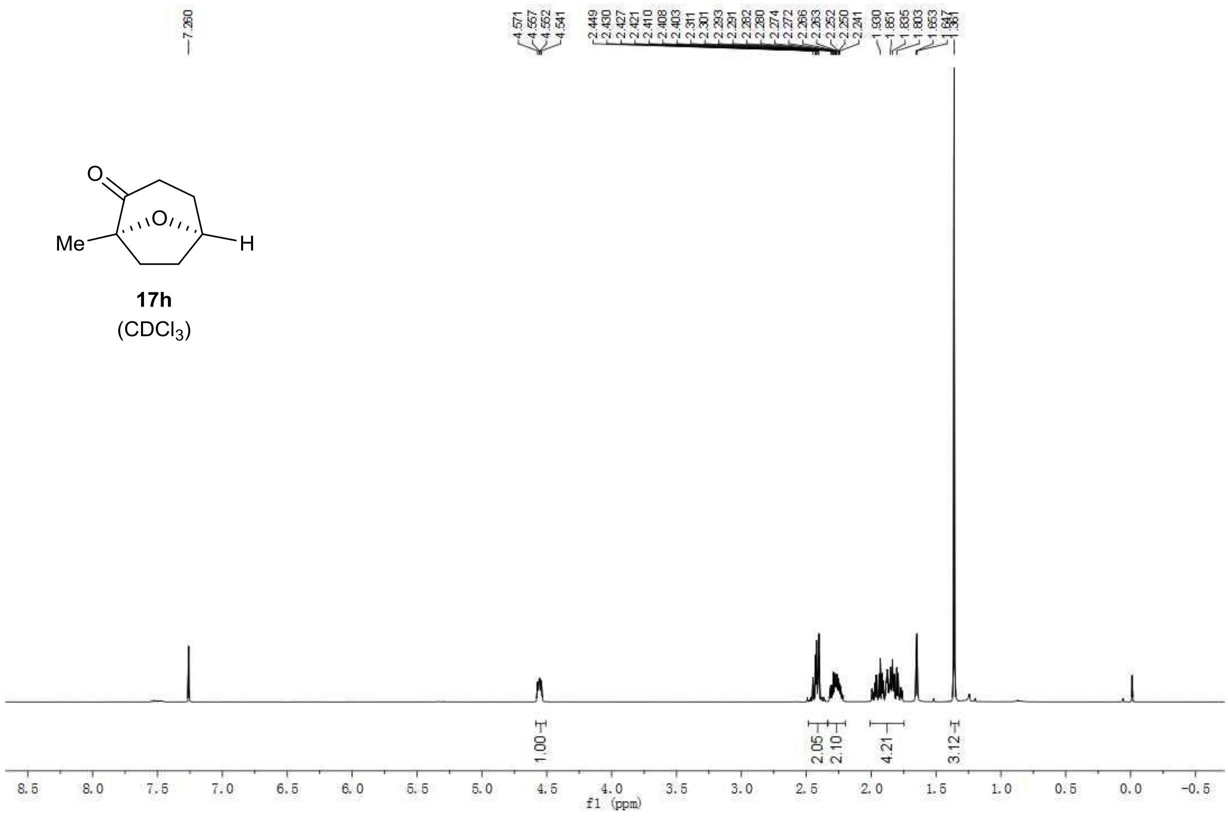

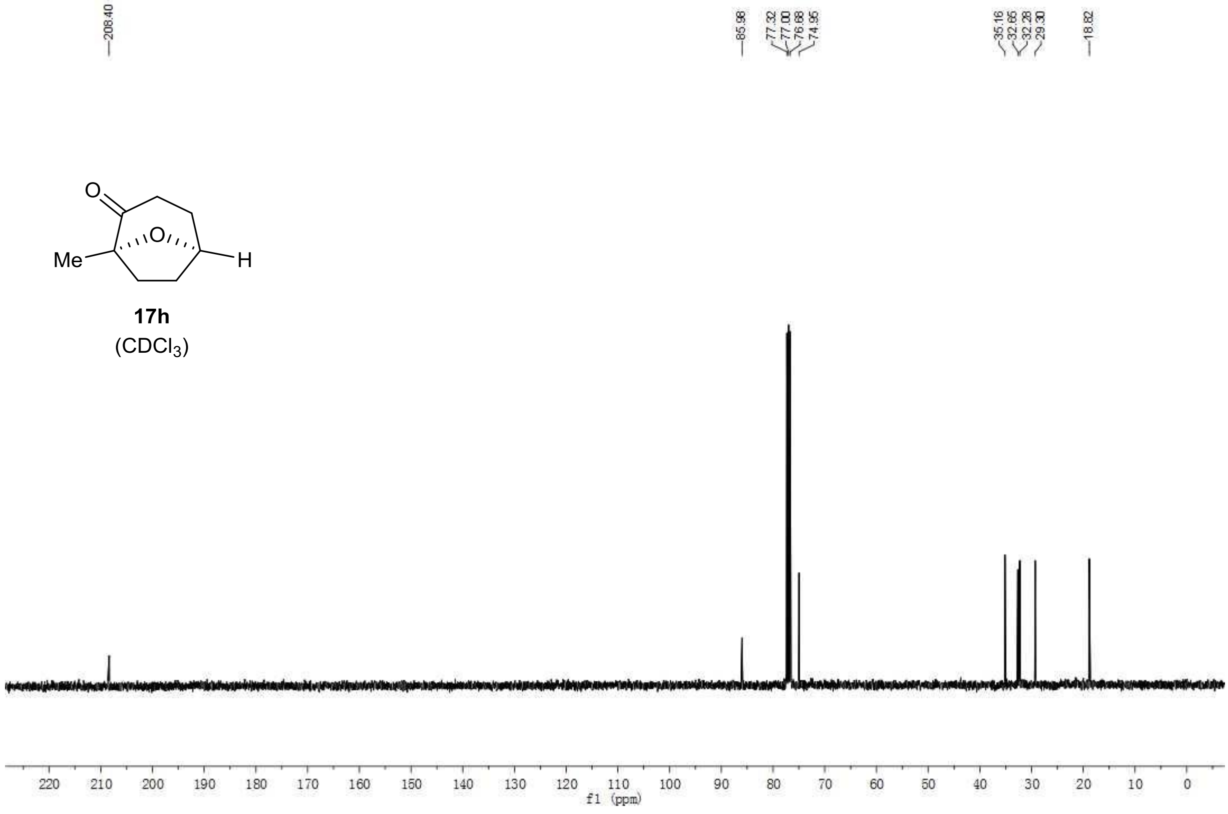

Supplementary Figure 54. ^{1}H and ^{13}C NMR spectra for 17h.

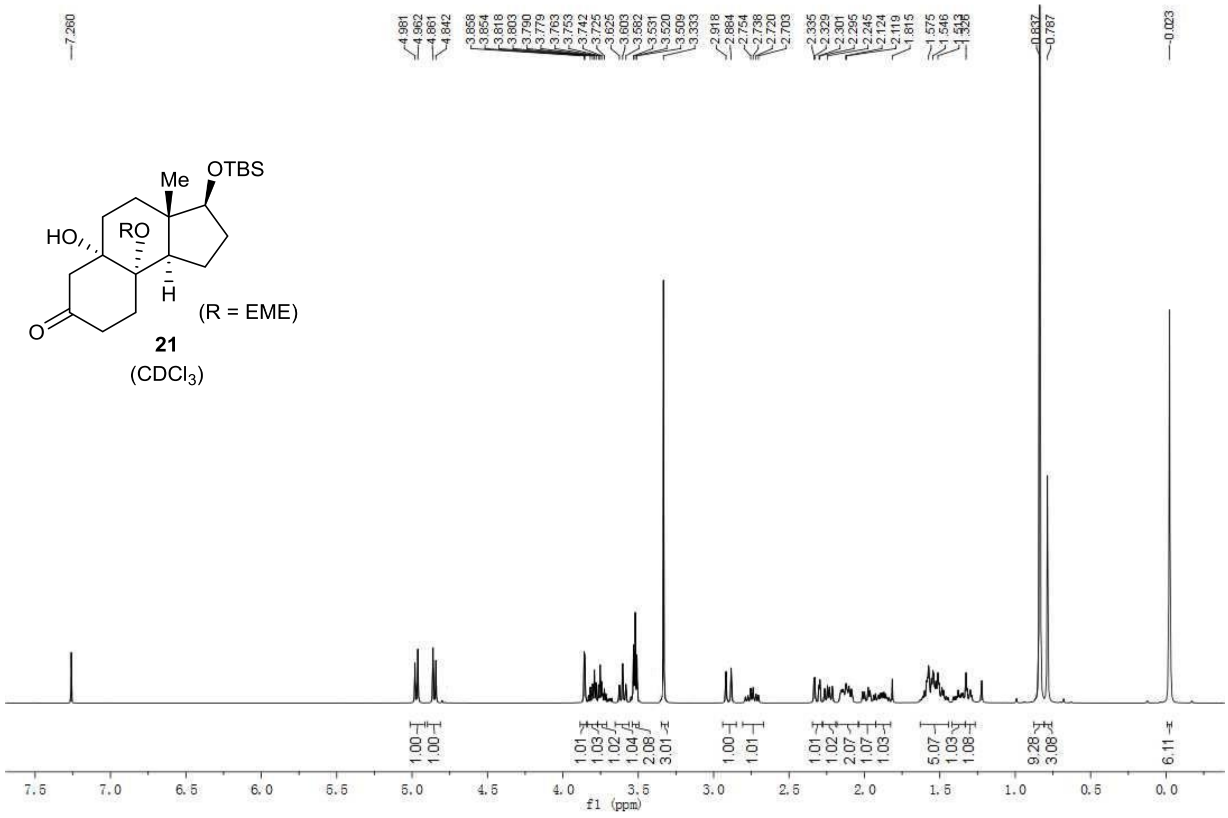

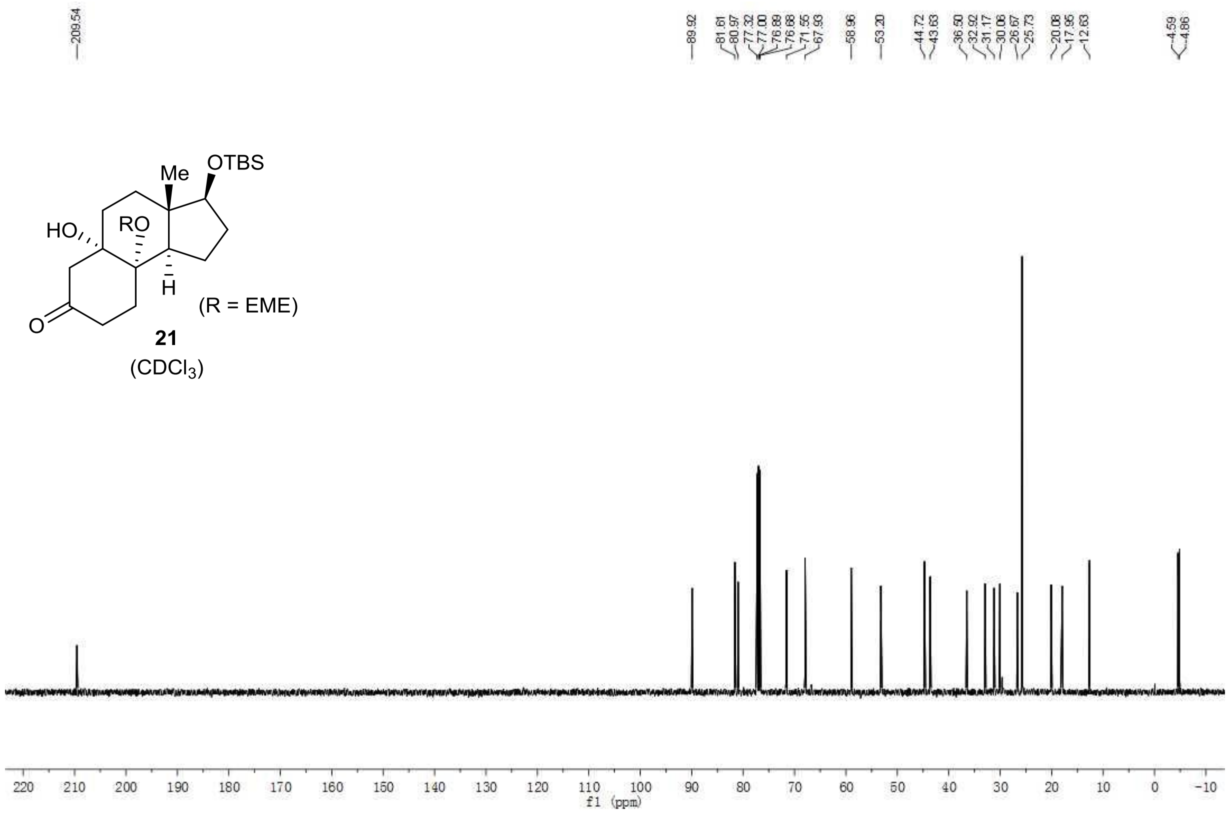

Supplementary Figure 55. ^{1}H and ^{13}C NMR spectra for 21.

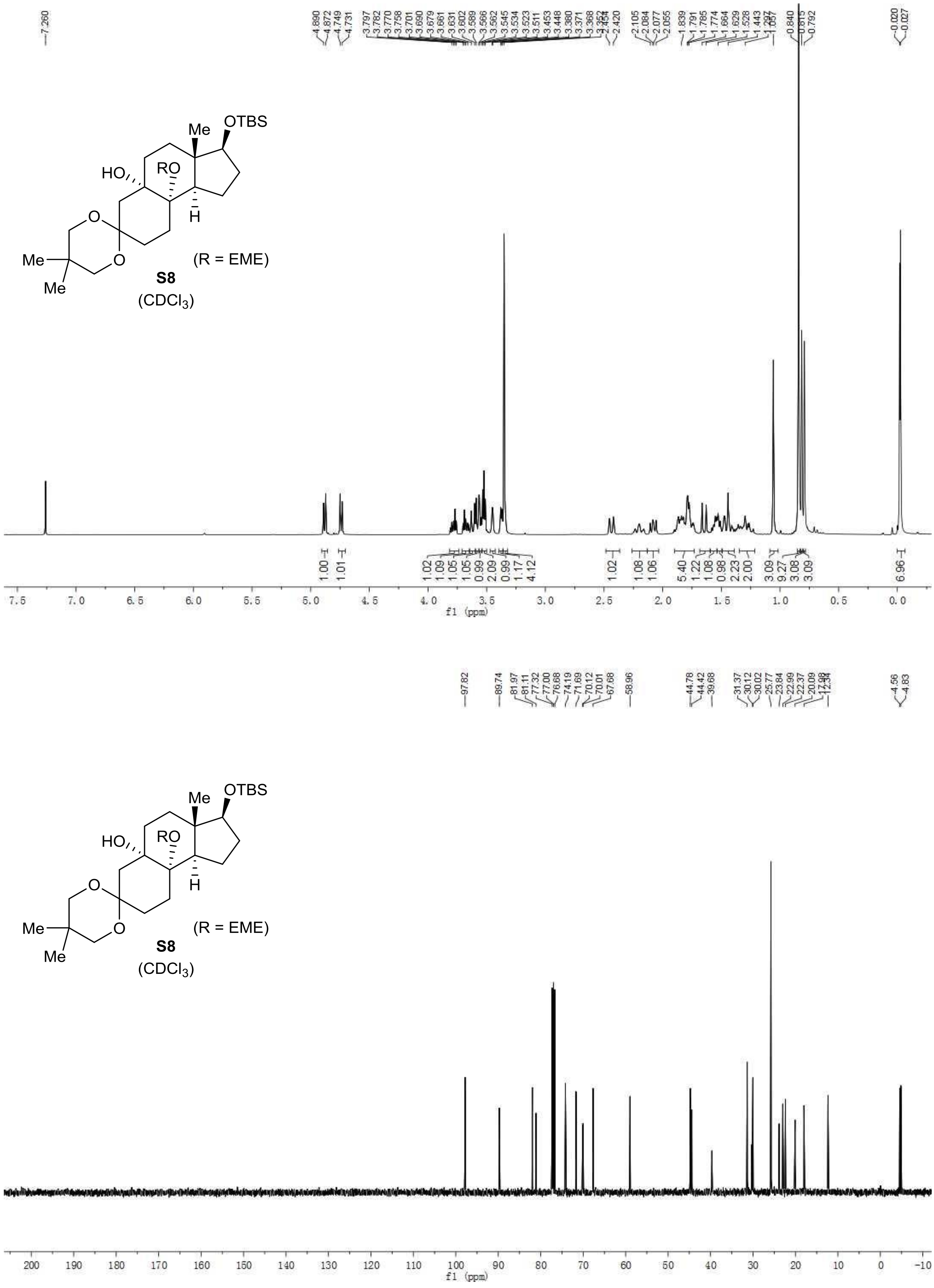

Supplementary Figure 56. ^{1}H and ^{13}C NMR spectra for S8.

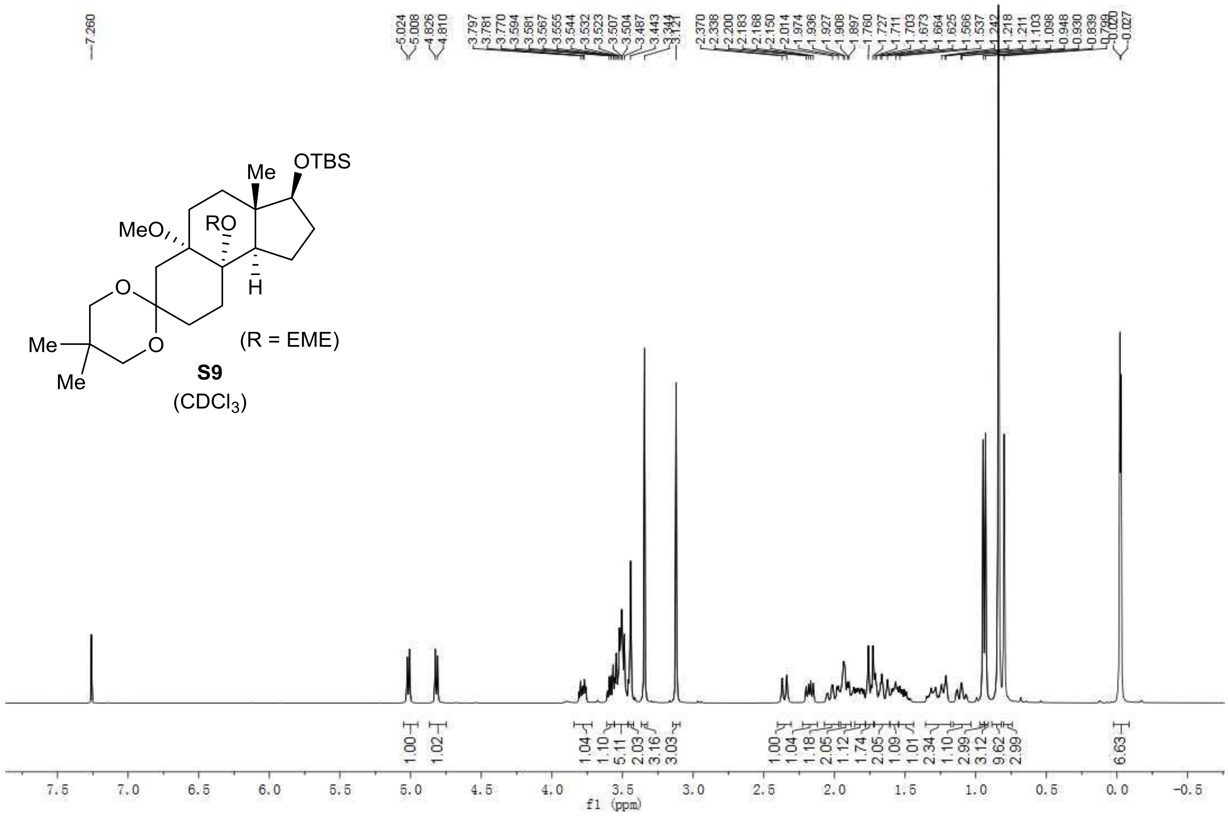

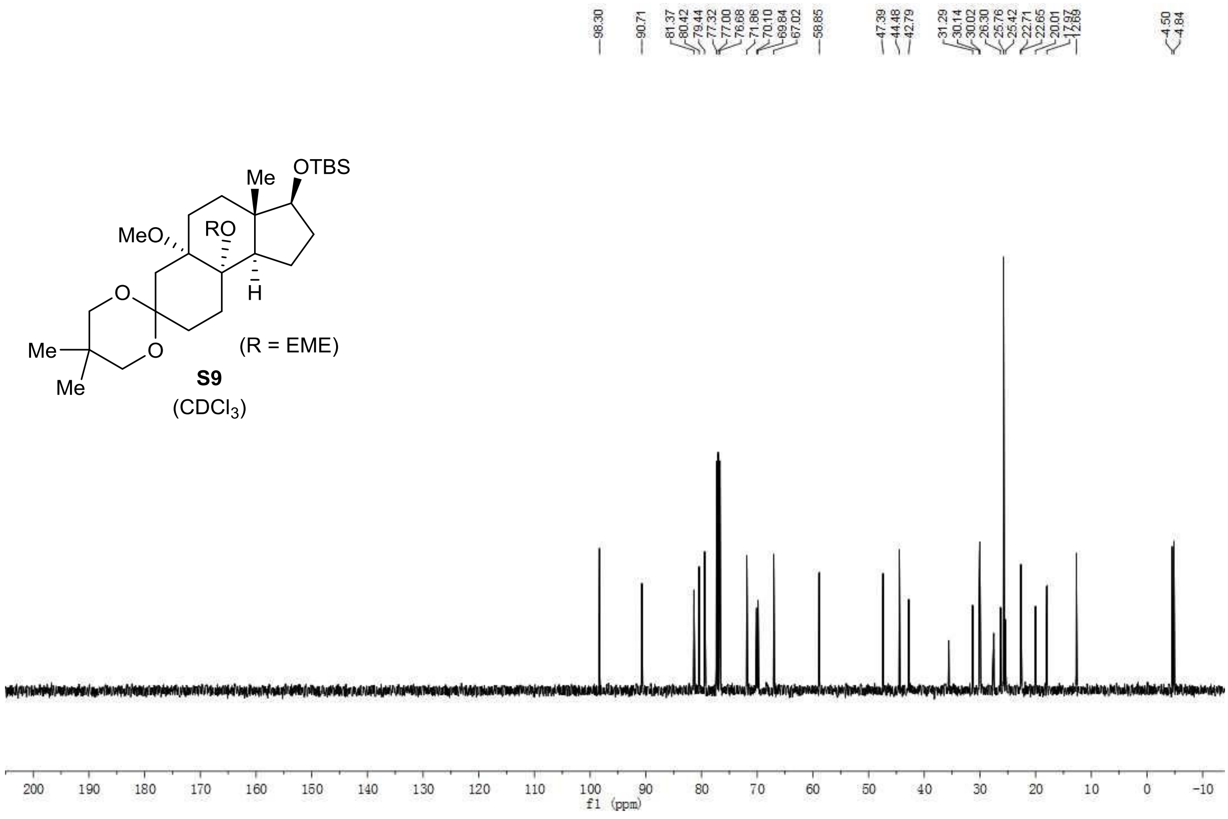

Supplementary Figure 57. ^{1}H and ^{13}C NMR spectra for S9.

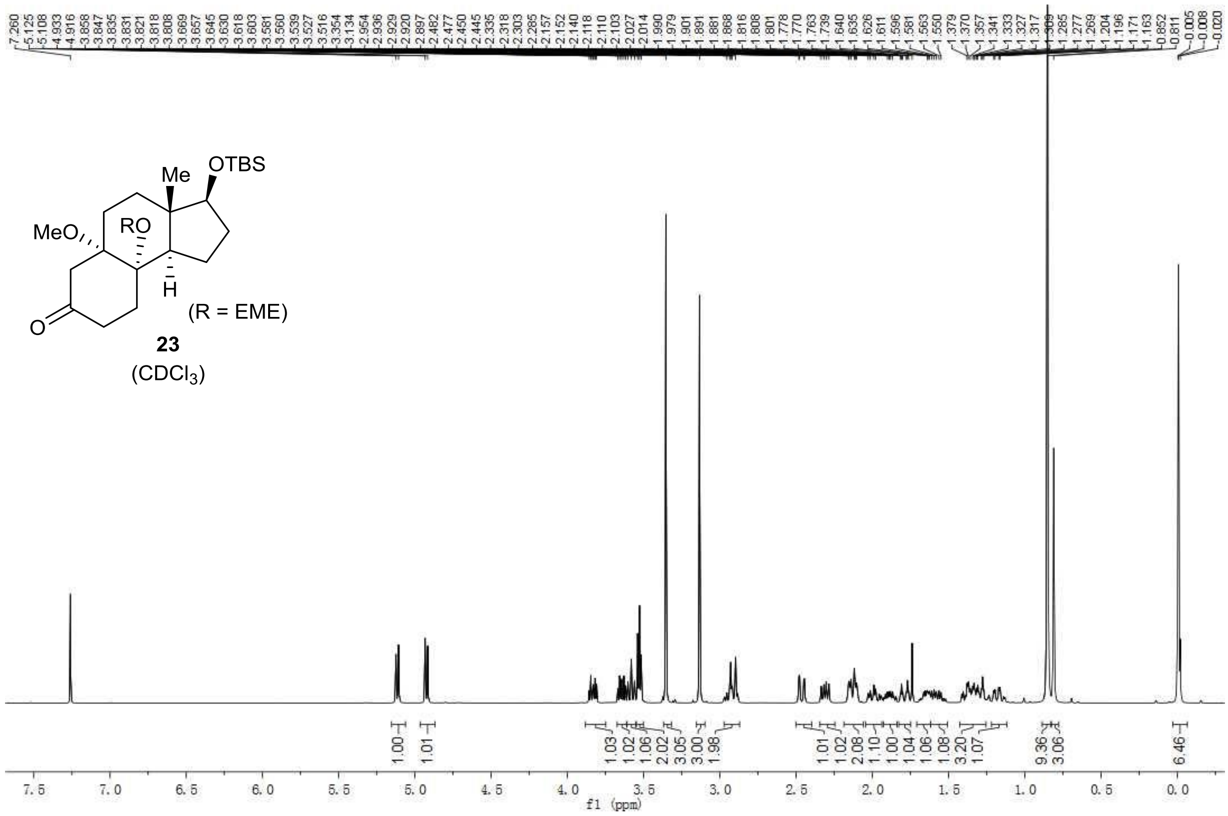

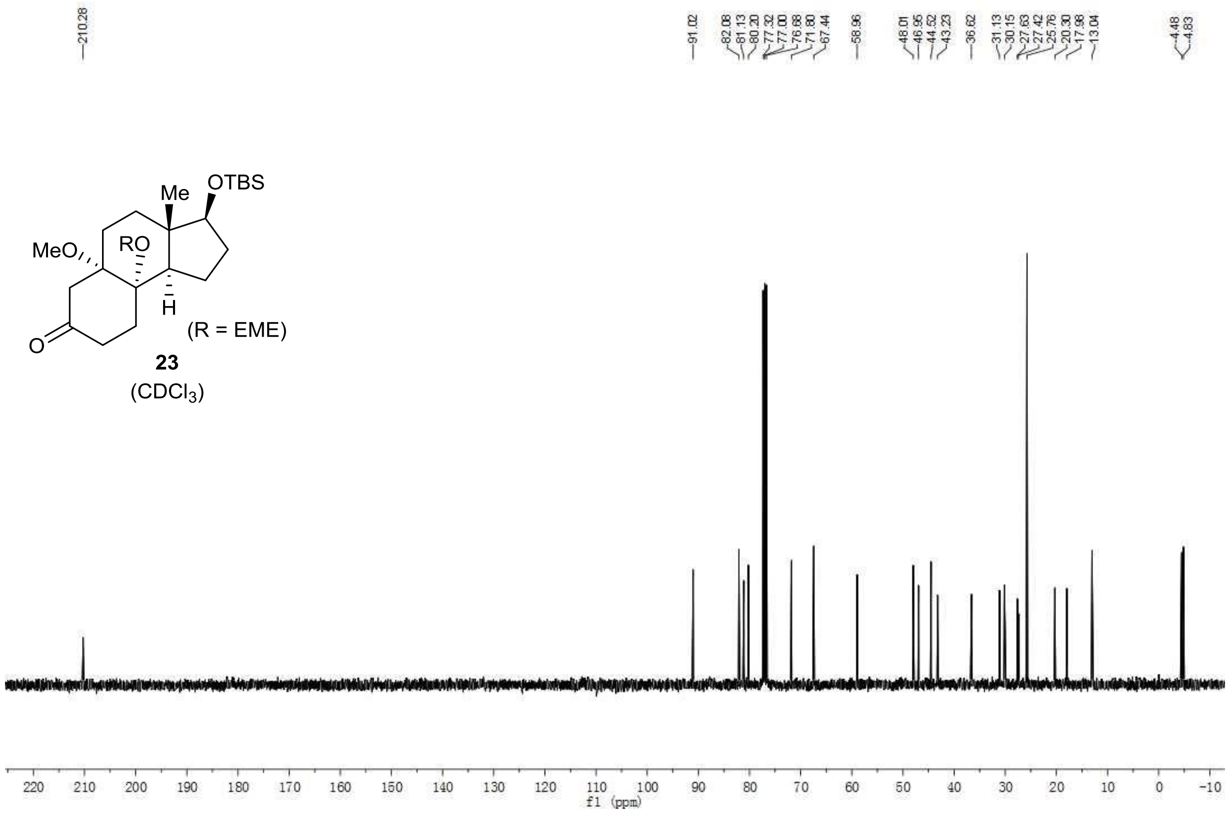

Supplementary Figure 58. ^{1}H and ^{13}C NMR spectra for 23.

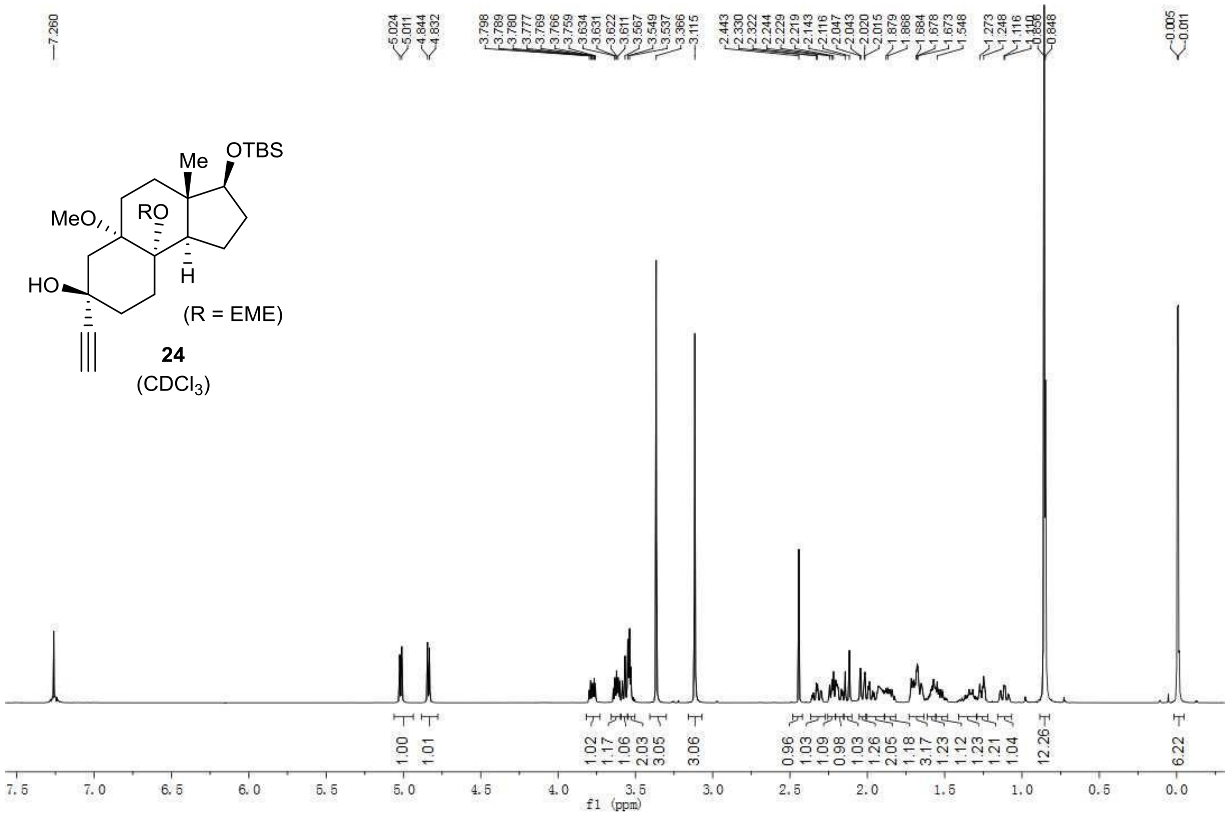

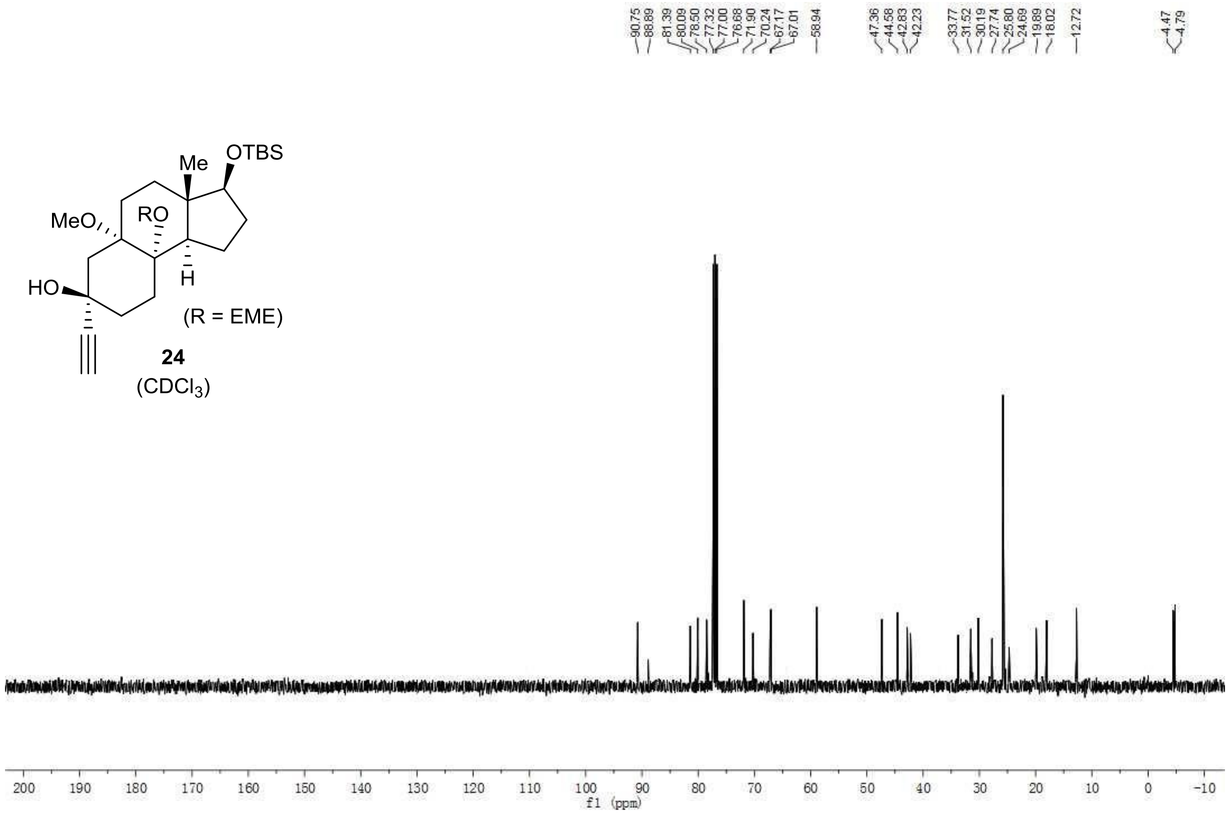

Supplementary Figure 59. ^{1}**H and** ^{13}**C NMR spectra for 24.**

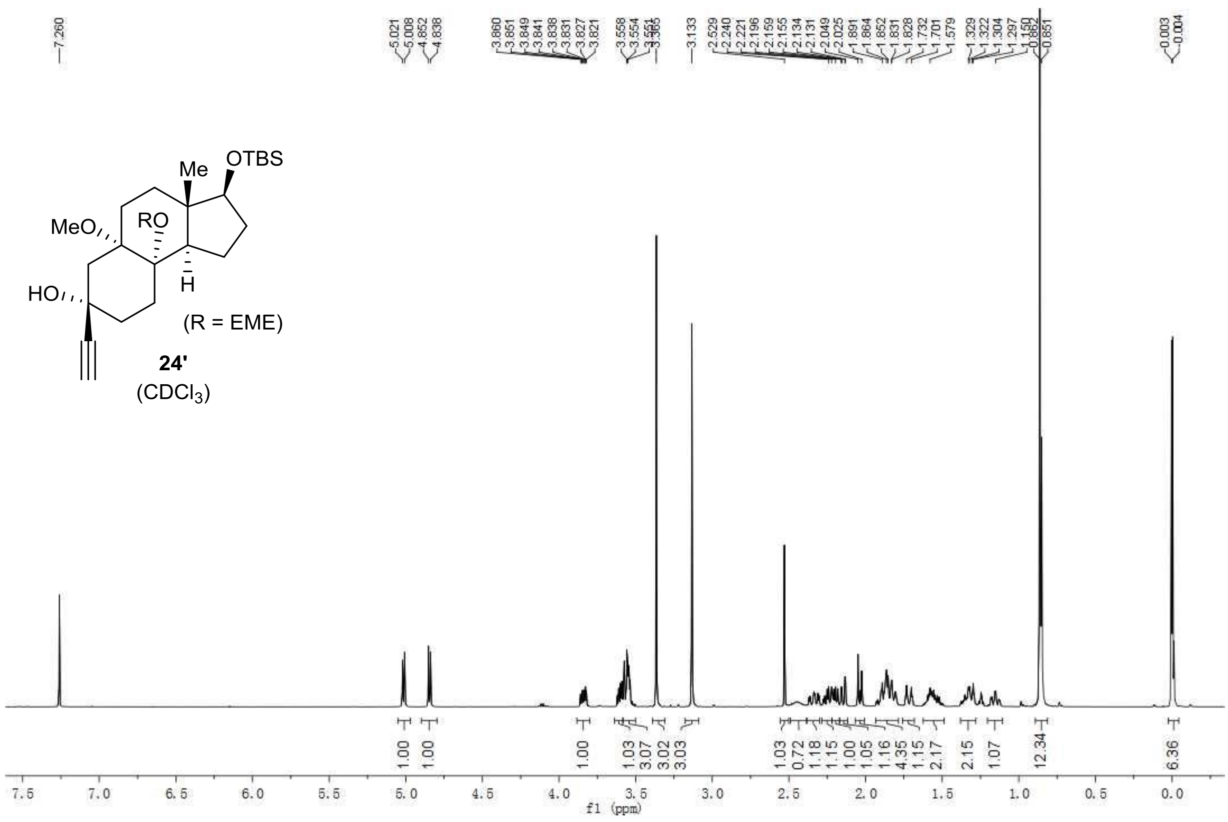

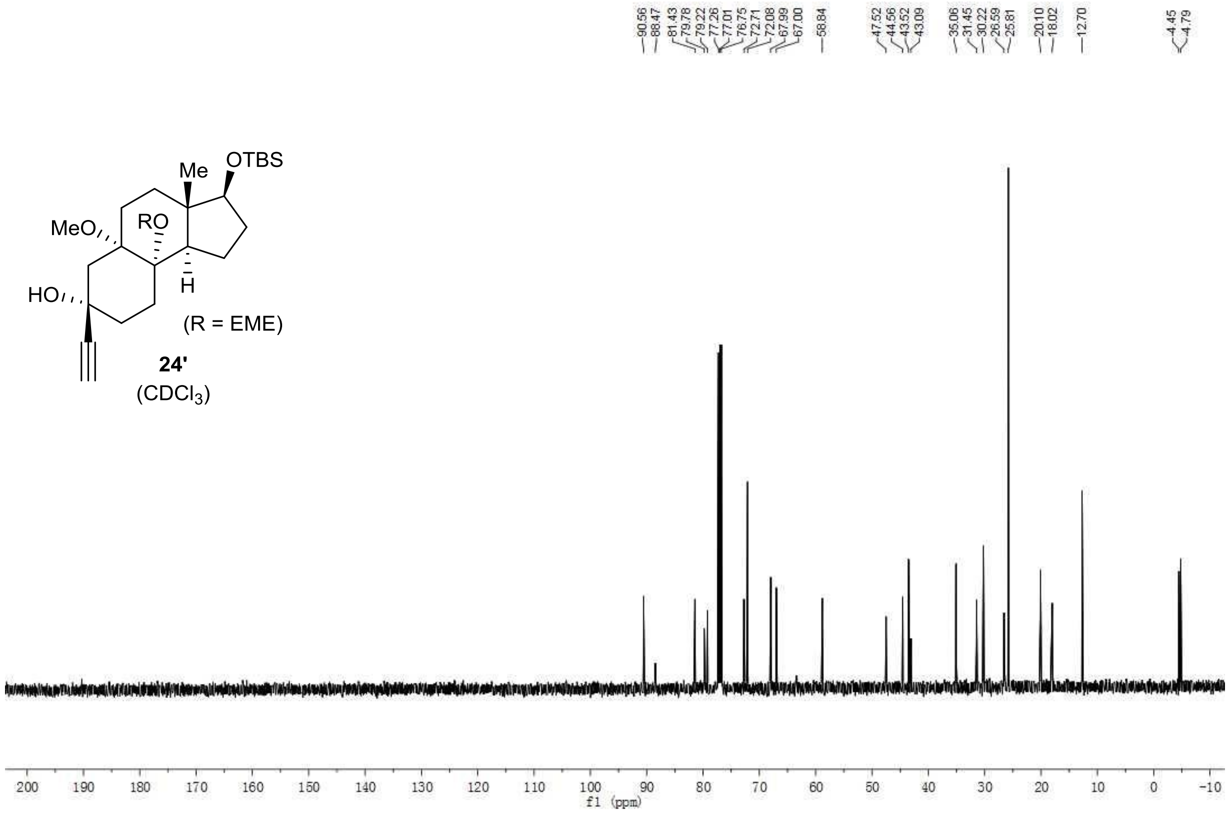

Supplementary Figure 60. ^{1}H and ^{13}C NMR spectra for 24'.

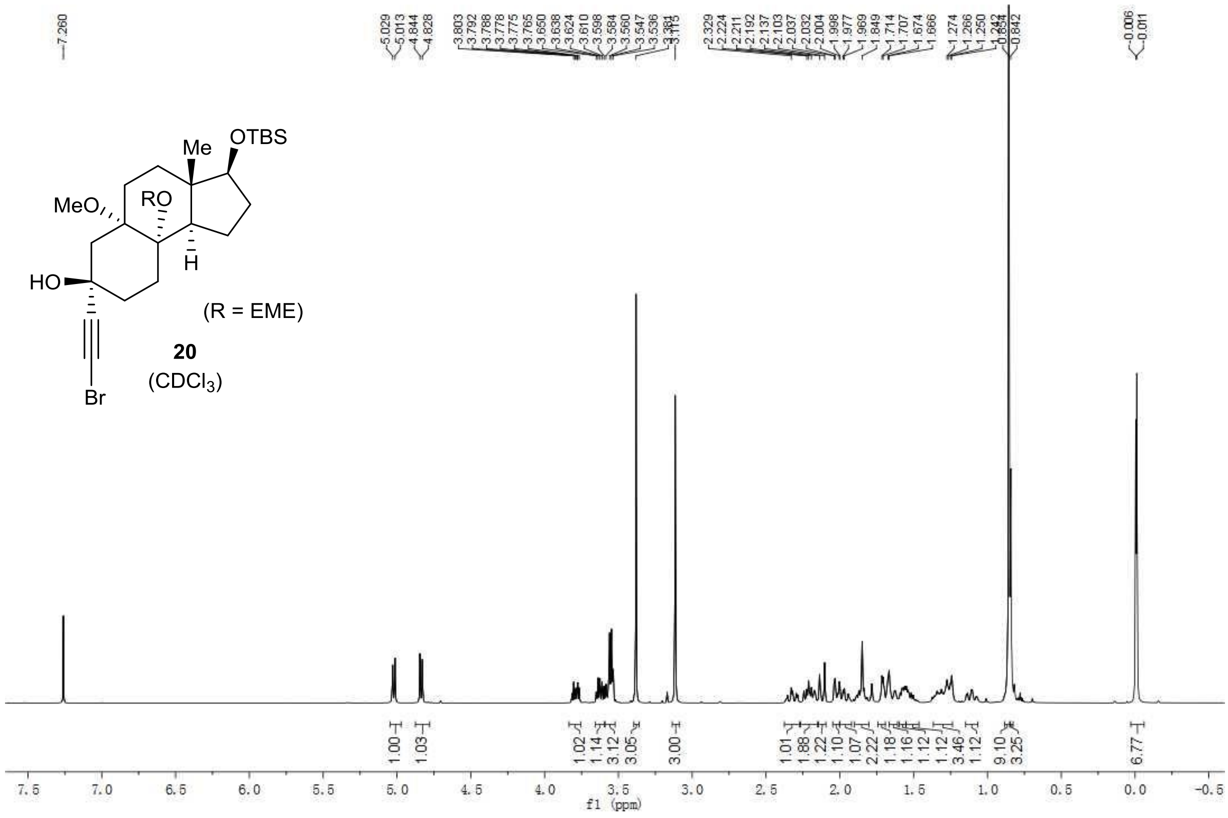

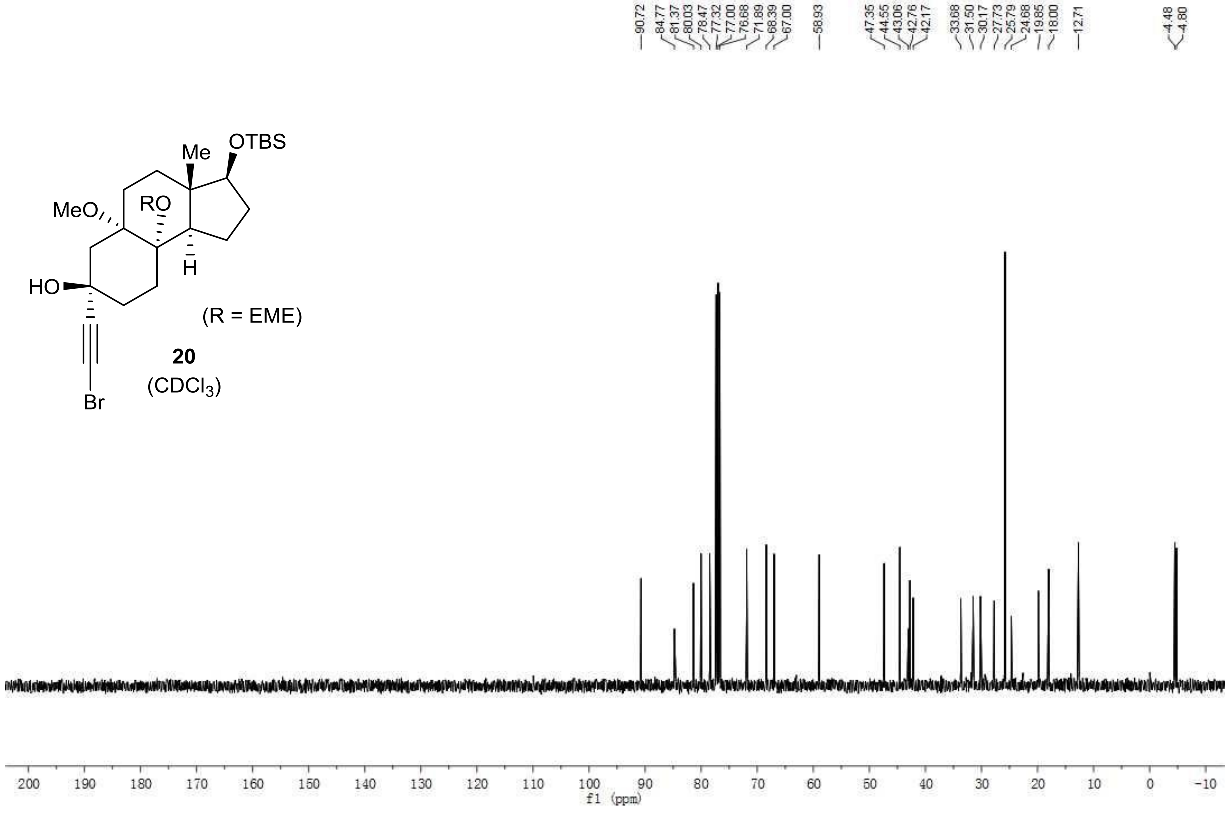

Supplementary Figure 61. ^{1}H and ^{13}C NMR spectra for 20.

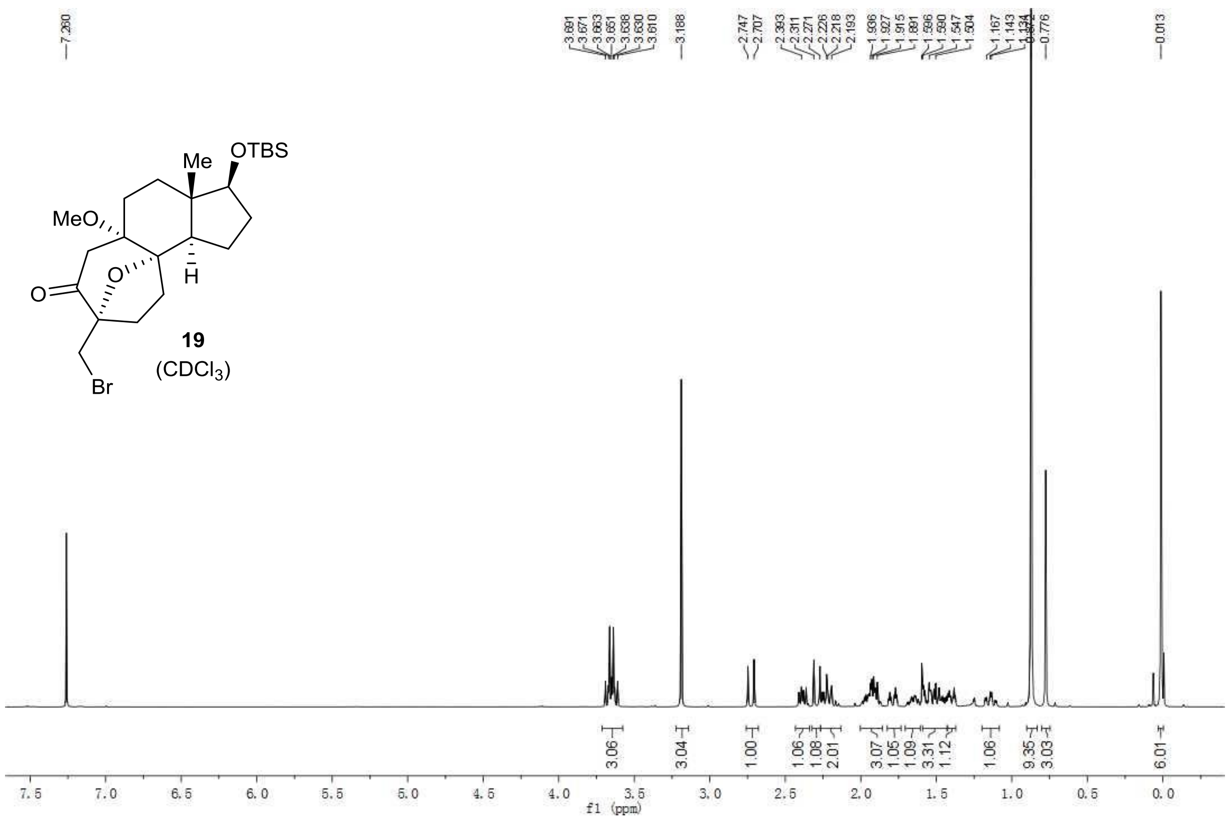

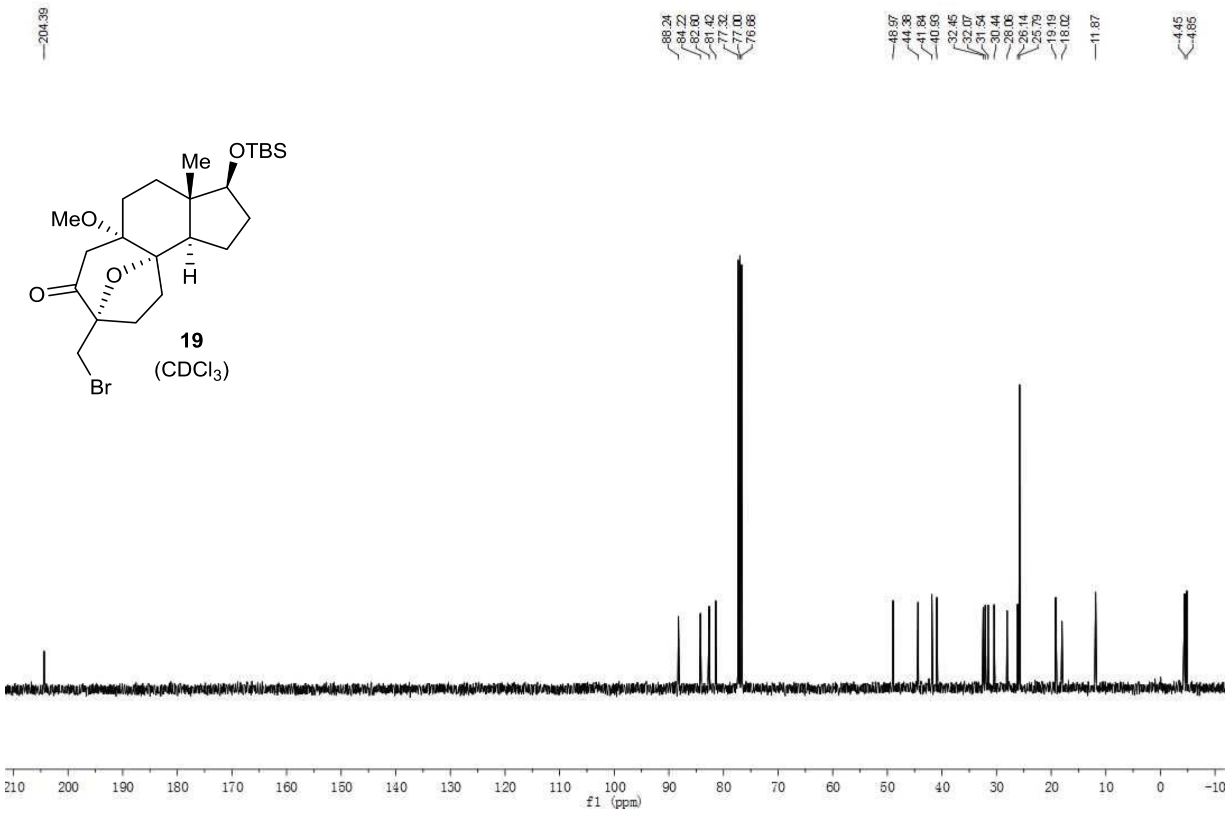

Supplementary Figure 62. ^{1}H and ^{13}C NMR spectra for 19.

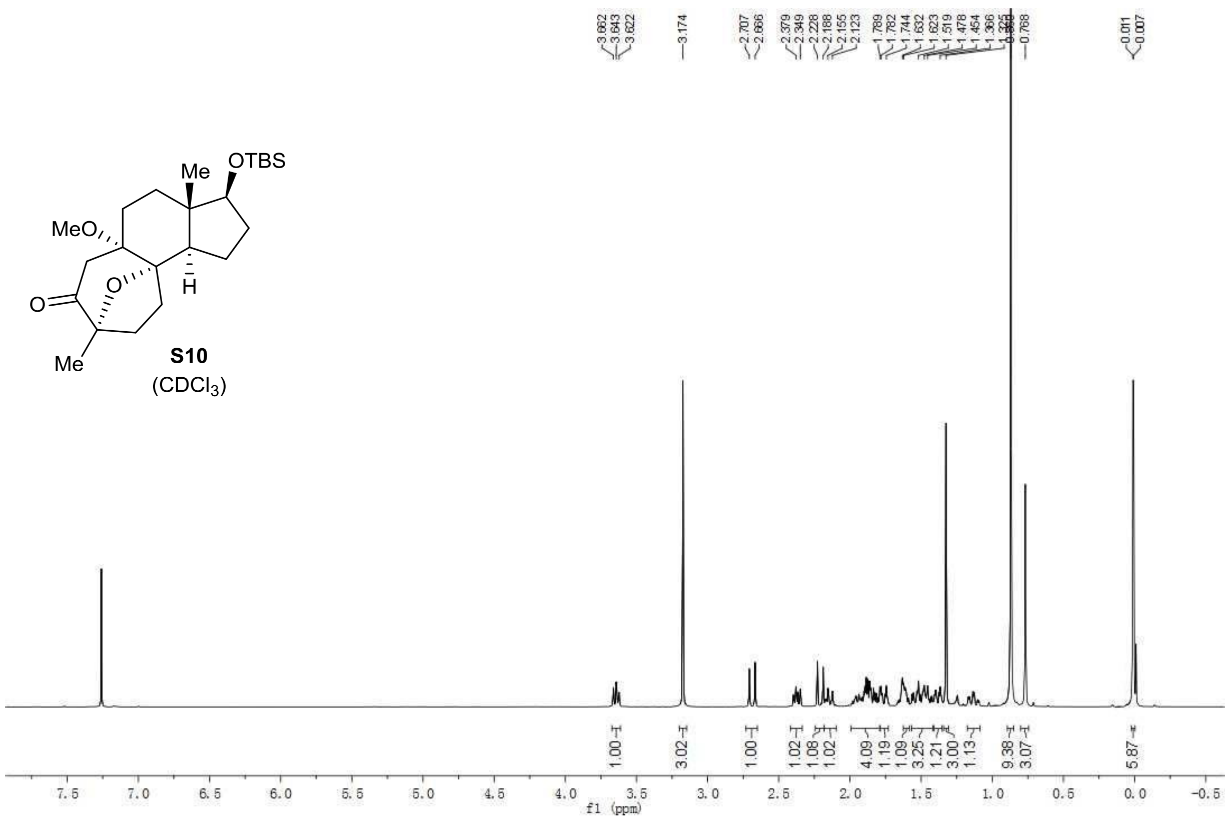

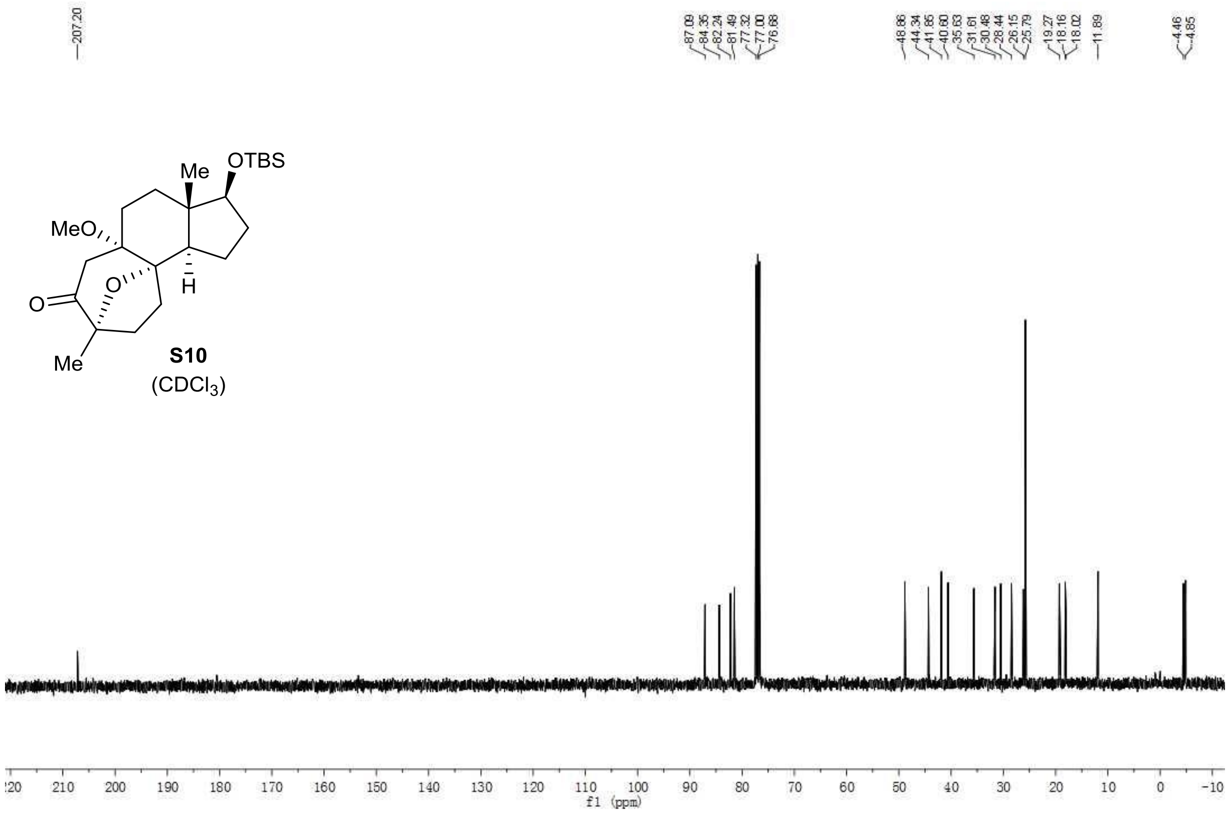

Supplementary Figure 63. ^{1}H and ^{13}C NMR spectra for S10.

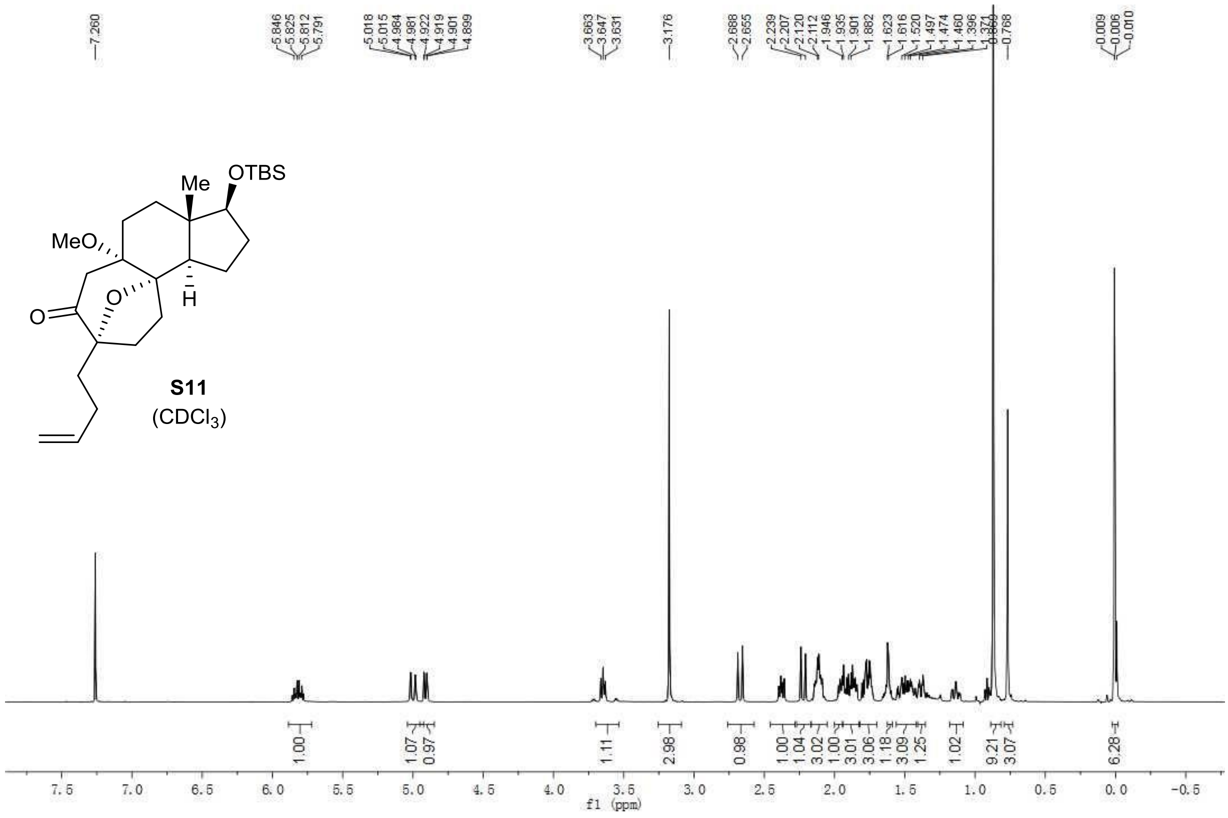

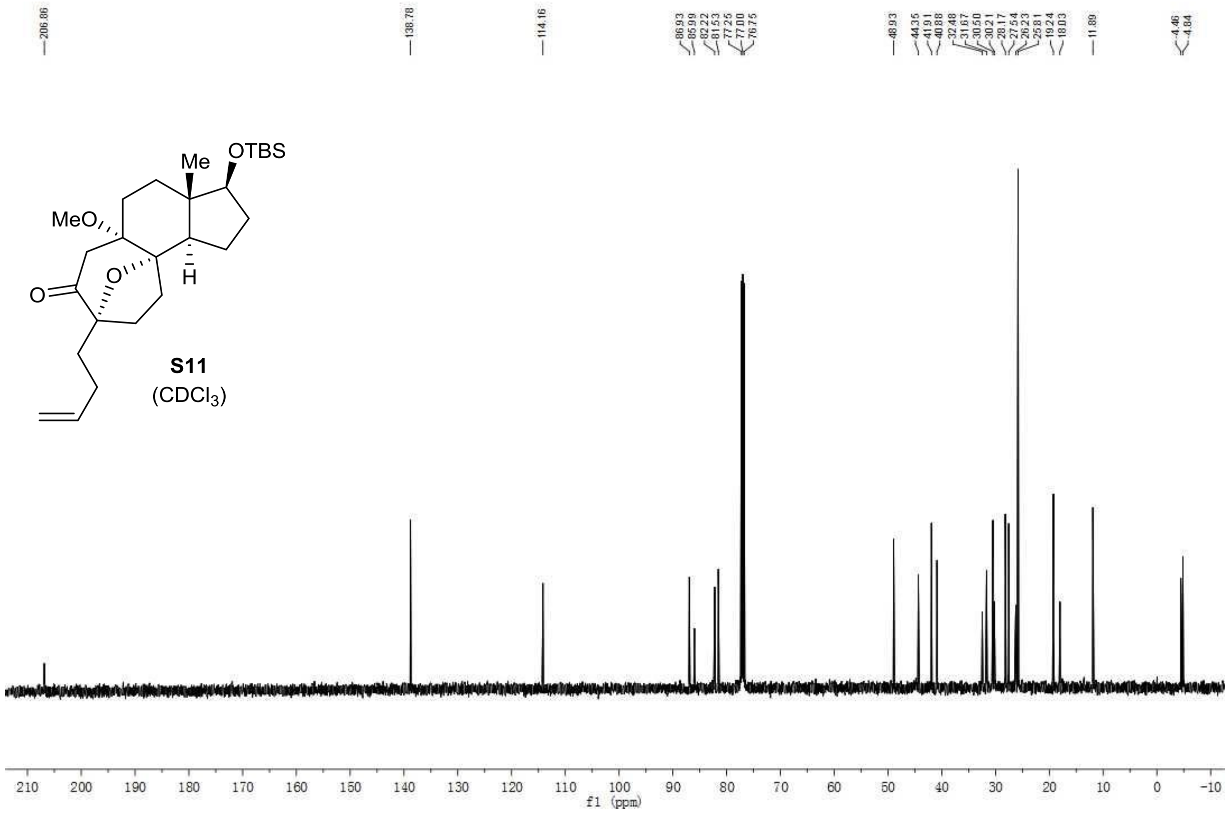

Supplementary Figure 64. ^{1}H and ^{13}C NMR spectra for S11.

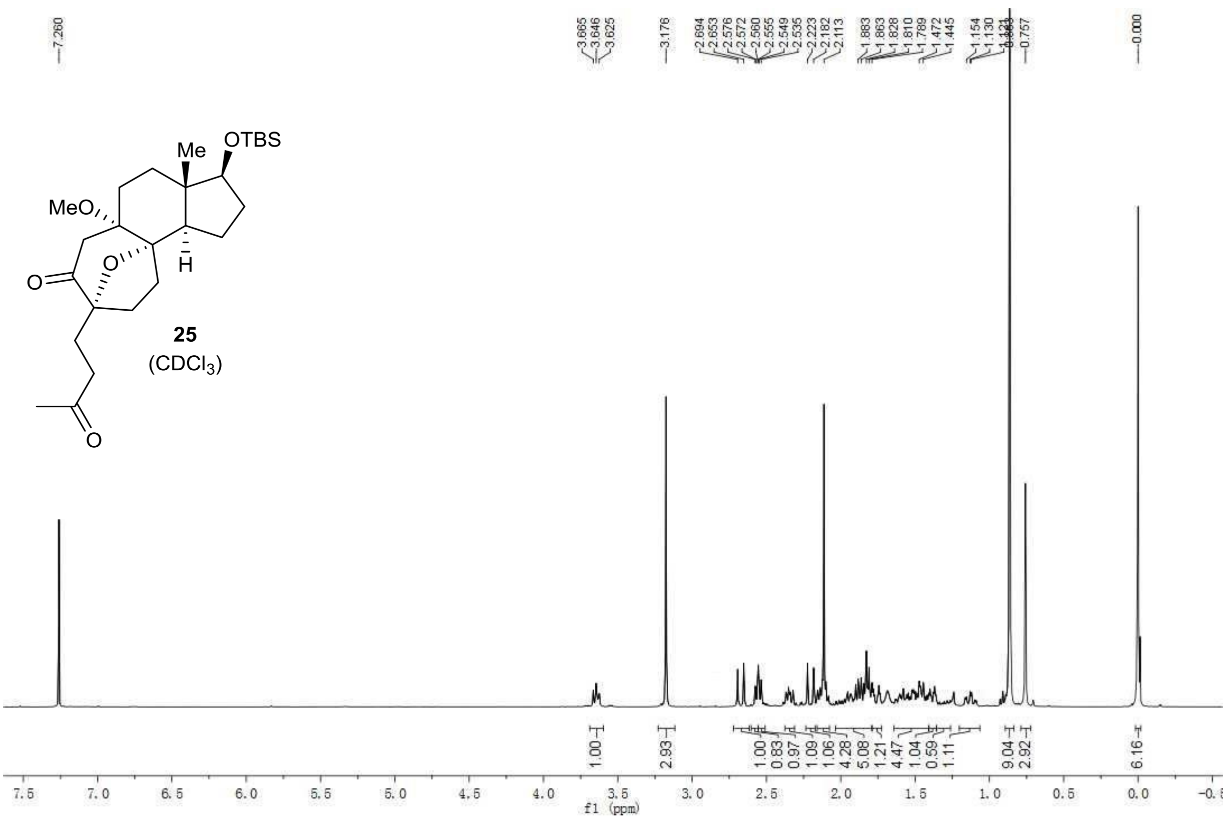

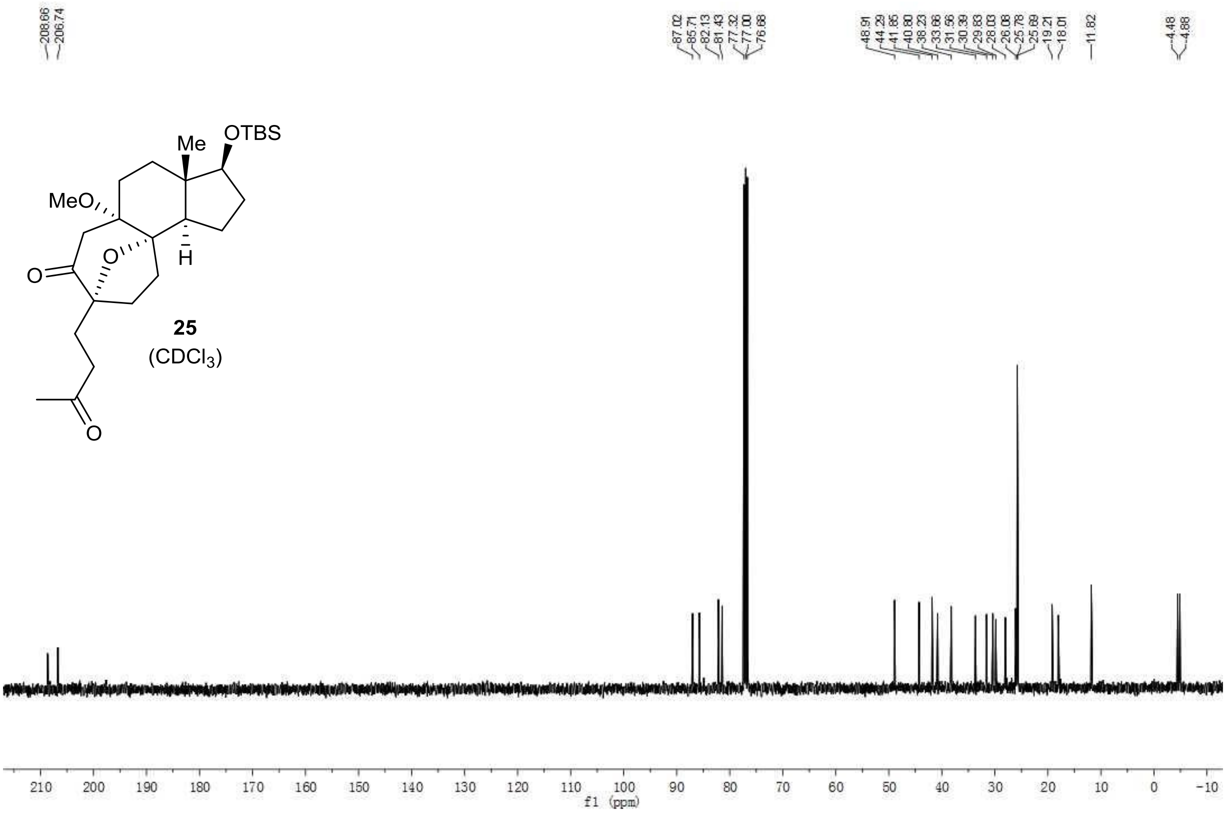

Supplementary Figure 65. ^{1}H and ^{13}C NMR spectra for 25.

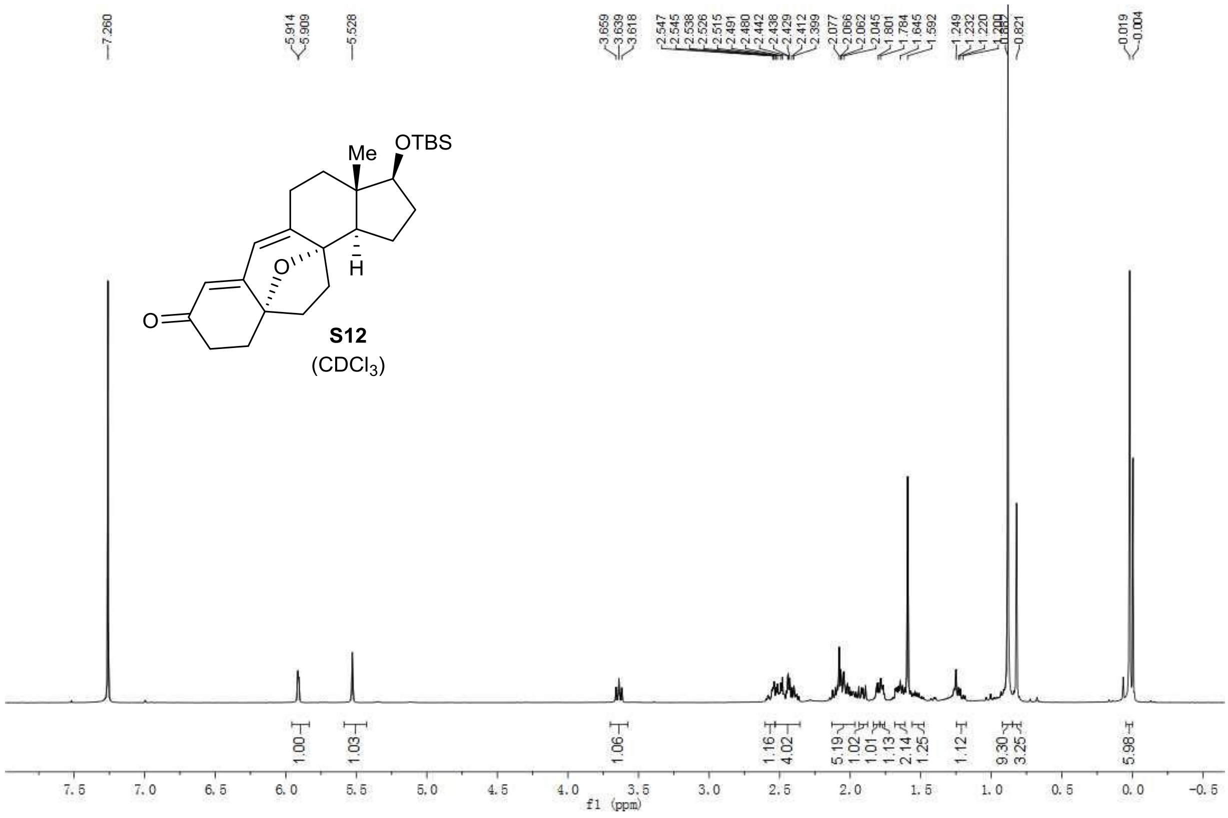

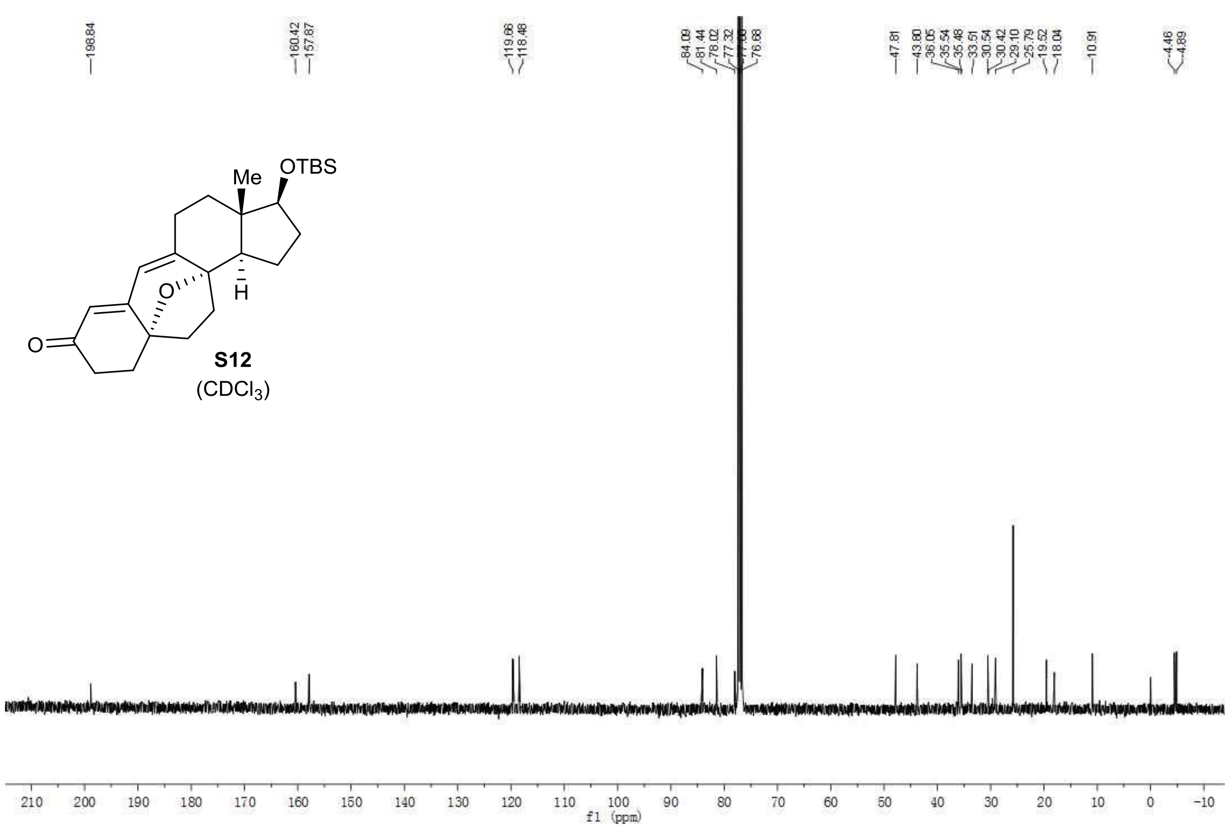

Supplementary Figure 66. ^{1}H and ^{13}C NMR spectra for S12.

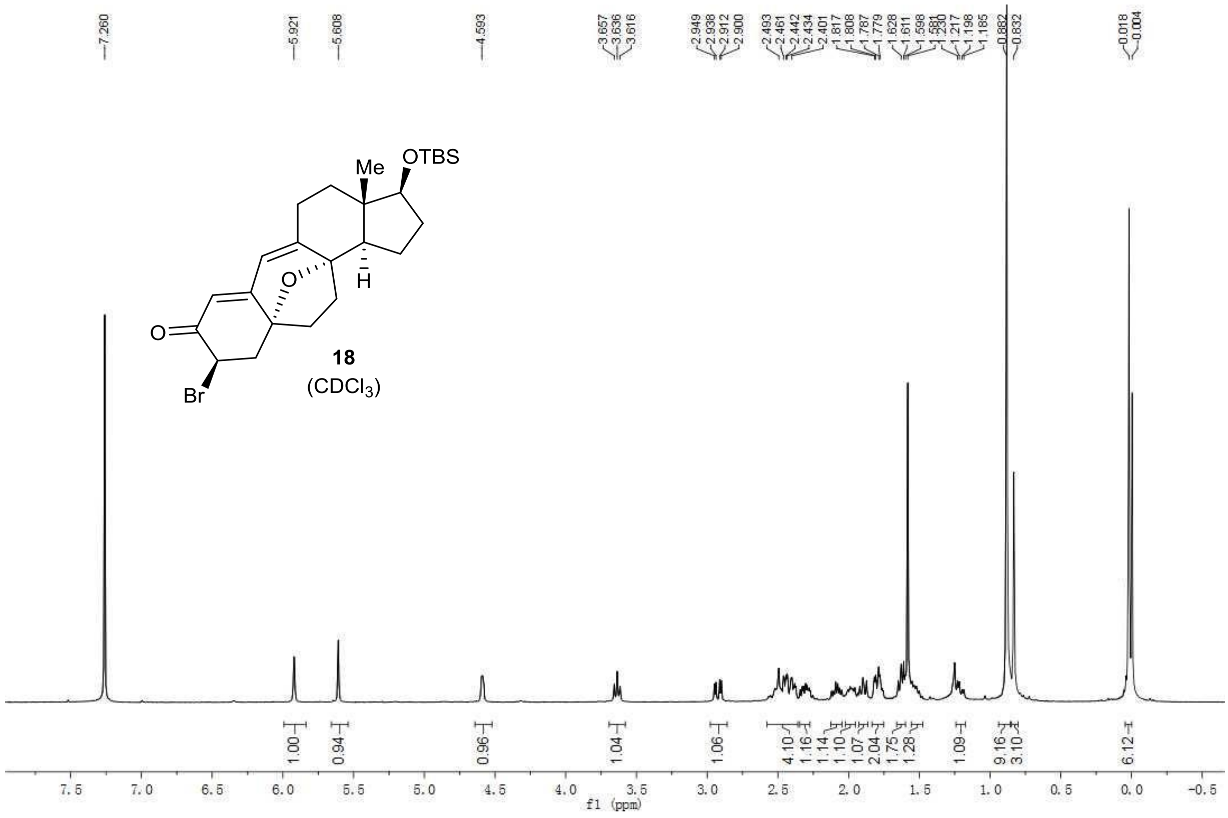

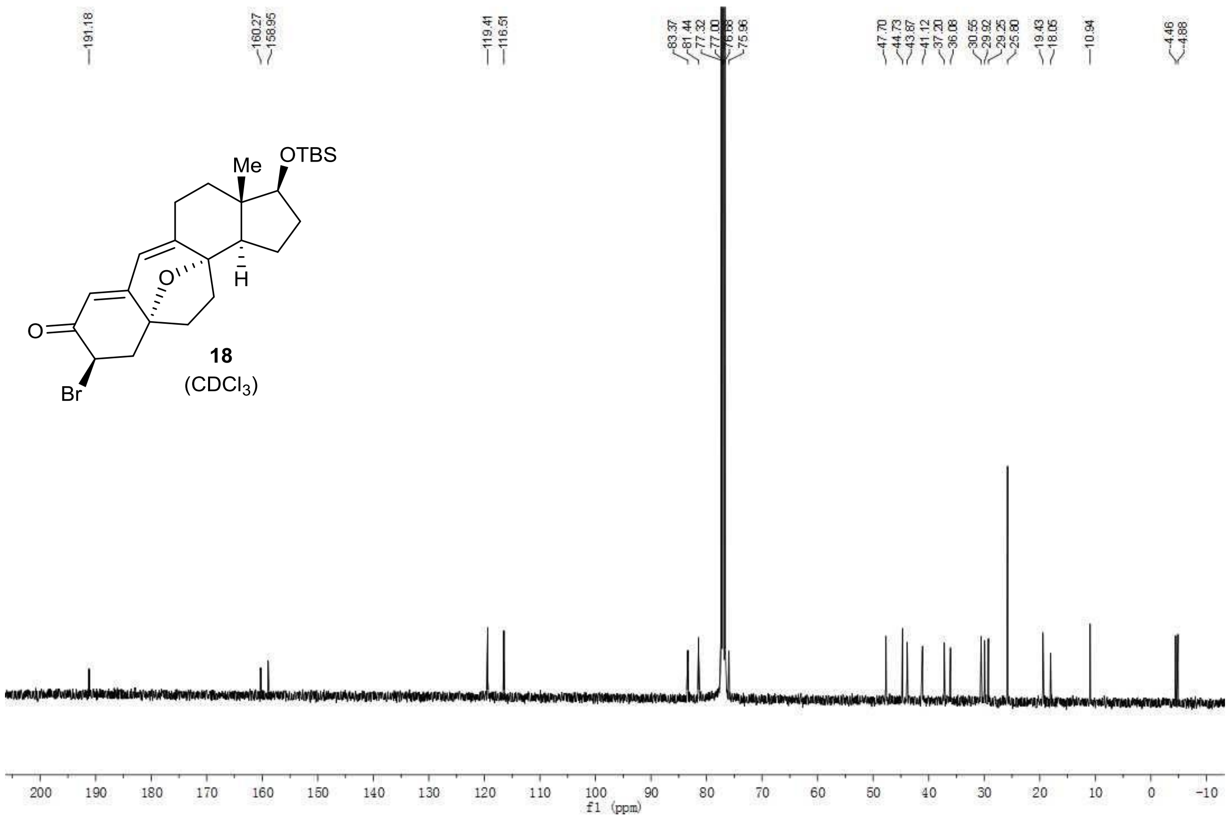

Supplementary Figure 67. ^{1}**H and** ^{13}**C NMR spectra for 18.**

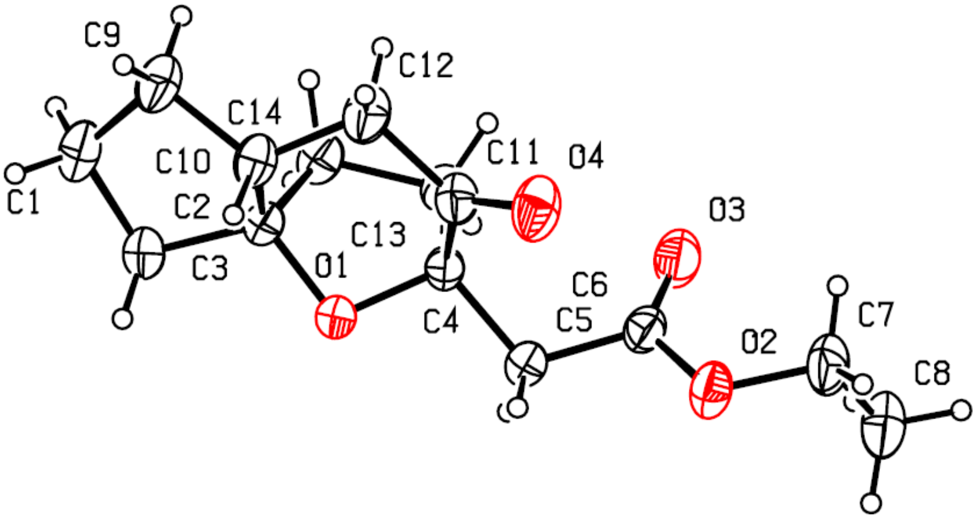

Supplementary Figure 68. ORTEP Diagram of 12k.

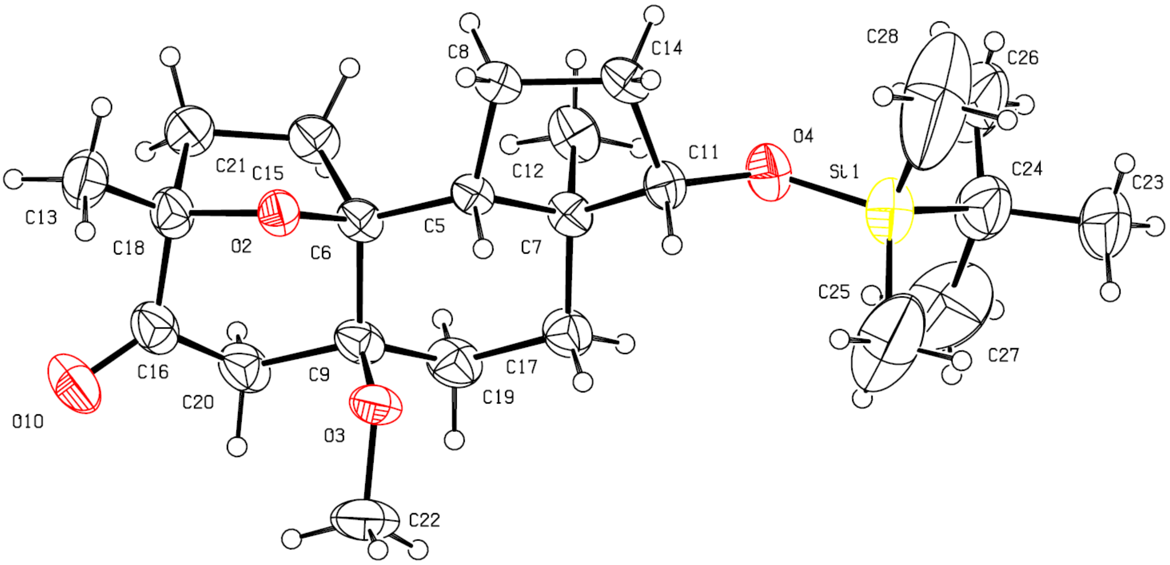

Supplementary Figure 69. ORTEP Diagram of S10.

Supplementary Tables

Supplementary Table 1. B3LYP and N12 absolute calculation energies, enthalpies, and free energies.

Geometry	$E_{(elec\text{-}B3LYP)}$[1]	$H_{(corr\text{-}B3LYP)}$[2]	$G_{(corr\text{-}B3LYP)}$[3]	$E_{(solv\text{-}M11\text{-}L)}$[4]	IF[5]
**CP1**	-1790.455064	0.591244	0.490822	-1791.25188	-
**TS1**	-1790.440304	0.591463	0.488710	-1791.237023	-78.4
**CP2**	-1790.444994	0.592165	0.487391	-1791.241247	-
**TS2**	-1790.443426	0.590264	0.490312	-1791.238878	-30.9
**CP3**	-1790.449215	0.593613	0.492016	-1791.251255	-
**CP4**	-1790.523155	0.594810	0.493683	-1791.316112	-
**CP5**	-1790.523028	0.594777	0.493741	-1791.317507	-
**TS3**	-1790.464026	0.592226	0.492469	-1791.264216	-368.3
**CP6**	-1790.482440	0.593169	0.492657	-1791.279936	-
**9**	-618.500669	0.295141	0.242679	-618.6268057	-
**10**	-618.586898	0.297303	0.247420	-618.702179	-
**TS4**	-1790.458234	0.591369	0.49364	-1791.25849	-339.4
**CP7**	-1790.475386	0.591630	0.496775	-1791.273608	-
**10’**	-618.576915	0.297582	0.247827	-618.693363	-

[1]The electronic energy calculated by B3LYP in gas phase. [2]The thermal correction to enthalpy calculated by B3LYP in gas phase. [3]The thermal correction to Gibbs free energy calculated by B3LYP in gas phase. [4]The electronic energy calculated by N12 in dichloromethane solvent. [5]The B3LYP calculated imaginary frequencies for the transition states.

Complete reference for Gaussian 09

Frisch, M. J.; Trucks, G. W.; Schlegel, H. B.; Scuseria, G. E.; Robb, M. A.; Cheeseman, J. R.; Scalmani, G.; Barone, V.; Mennucci, B.; Petersson, G. A.; Nakatsuji, H.; Caricato, M.; Li, X.; Hratchian, H. P.; Izmaylov, A. F.; Bloino, J.; Zheng, G.; Sonnenberg, J. L.; Hada, M.; Ehara, M.; Toyota, K.; Fukuda, R.; Hasegawa, J.; Ishida, M.; Nakajima, T.; Honda, Y.; Kitao, O.; Nakai, H.; Vreven, T.; Montgomery, Jr., J. A.; Peralta, J. E.; Ogliaro, F.; Bearpark, M.; Heyd, J. J.; Brothers, E.; Kudin, K. N.; Staroverov, V. N.; Keith, T.; Kobayashi, R.; Normand, J.; Raghavachari, K.; Rendell, A.; Burant, J. C.; Iyengar, S. S.; Tomasi, J.; Cossi, M.; Rega, N.; Millam, J. M.; Klene, M.; Knox, J. E.; Cross, J. B.; Bakken, V.; Adamo, C.; Jaramillo, J.; Gomperts, R.; Stratmann, R. E.; Yazyev, O.; Austin, A. J.; Cammi, R.; Pomelli, C.; Ochterski, J. W.; Martin, R. L.; Morokuma, K.; Zakrzewski, V. G.; Voth, G. A.; Salvador,

P.; Dannenberg, J. J.; Dapprich, S.; Daniels, A. D.; Farkas, O.; Foresman, J. B.; Ortiz, J. V.; Cioslowski, J.; and Fox, D. J. Gaussian 09, revision D.01; Gaussian, Inc.: Wallingford, CT, 2013.

All of the DFT calculations conducted in this study were carried out using the GAUSSIAN 09 series of programs. DFT method B3LYP[1-2] with a standard 6-31+G(d) basis set (SDD basis set for Au) was used for the geometry optimizations. The N12 functional, proposed by Truhlar et al.,[3-4] was used with a 6-311++G(d,p) basis set (SDD basis set for Au) to calculate the single point energies. The solvent effects were taken into consideration using single point calculations based on the gas-phase stationary points with a SMD continuum solvation model.[4] The energies presented in this paper are the N12 calculated Gibbs free energies in a dichloromethane solvent with B3LYP calculated thermodynamic corrections.

Supplementary Table 2. B3LYP geometries for all the optimized compounds and transition states.

CP1

C	-5.43755100	-0.64386500	0.54240000
C	-4.18142000	-1.51324500	0.78264200
C	-2.84433100	-0.83550700	0.37611500
C	-2.92580500	-0.25336300	-1.05044200
C	-4.15772800	0.64392700	-1.23227700
C	-5.46426400	-0.10228500	-0.91396400
H	-4.12407000	-1.81560900	1.83811900
H	-4.27528000	-2.43157600	0.19330100
H	-6.29333000	-1.33116500	0.63511300
H	-2.00613800	0.31028000	-1.24635200
H	-2.96989800	-1.08260800	-1.76300000
H	-4.06887400	1.53224200	-0.59979100
H	-4.19341500	0.98472600	-2.27322900
O	-2.47417400	0.24433900	1.24348400
H	-2.65602900	-0.00526900	2.16515800
C	-1.78529900	-1.88310000	0.44969000
C	-1.00181600	-2.83002900	0.54857200
H	-0.58815500	-3.81243400	0.67583700
C	-6.71067700	0.77187200	-1.14749700
H	-6.65939900	1.20064800	-2.15623300
H	-7.59229500	0.11034900	-1.13257200
O	-5.47439200	-1.21540900	-1.83444400
H	-6.37272000	-1.58052100	-1.88199300
Au	0.48698500	-1.17296700	0.23625500
P	2.34731400	0.22029200	-0.04030600
C	2.48395900	0.80551400	-1.76840800
C	2.28220300	-0.11103100	-2.81549800
C	2.79526200	2.14134100	-2.06625400
C	2.40513500	0.30226900	-4.14234700
H	2.02903600	-1.14603500	-2.59740200
C	2.91127500	2.55050700	-3.39814600
H	2.94183700	2.86347600	-1.26900900
C	2.71947400	1.63409100	-4.43500800
H	2.24977700	-0.41211400	-4.94588700
H	3.15007000	3.58645900	-3.62169800
H	2.80864800	1.95609100	-5.46875100
C	2.23877900	1.69017300	1.04131800
C	3.39773300	2.29448800	1.55604000
C	0.97784900	2.23786500	1.33436400
C	3.29366200	3.44152900	2.34677000
H	4.37671300	1.87160900	1.34999000
C	0.88260200	3.38774100	2.12056900
H	0.07091100	1.76753400	0.96124100
C	2.03873300	3.99030700	2.62668300
H	4.19304900	3.90281100	2.74491600
H	-0.09458600	3.80696600	2.34327100
H	1.96142400	4.88123700	3.24365100
C	3.90445800	-0.64753200	0.37371000
C	5.02399600	-0.59393100	-0.47028100
C	3.97524300	-1.37065000	1.57777100
C	6.20276800	-1.25383200	-0.10836300
H	4.98257500	-0.04548300	-1.40628400
C	5.15572200	-2.02156500	1.93566400
H	3.11265300	-1.42268800	2.23829500
C	6.27091500	-1.96505100	1.09178000
H	7.06544000	-1.20945400	-0.76714500
H	5.20415200	-2.57581300	2.86875300
H	7.18799600	-2.47713500	1.36912100
C	-5.64621700	0.46862800	1.59409500
H	-5.69048300	0.00991500	2.59223800
H	-4.78911100	1.15251400	1.59943700
C	-6.92773000	1.27143500	1.32610200
H	-7.03392600	2.06685200	2.07404400
H	-7.80399300	0.61605200	1.44035500
C	-6.91640200	1.86697000	-0.08897500
H	-6.12126300	2.62181200	-0.16608800
H	-7.85752400	2.39291200	-0.29023800

TS1

C	-5.51709800	-0.72483300	-0.42602800
C	-4.20415300	-1.56665000	-0.37923500
C	-2.83965400	-0.87400400	0.00930900
C	-2.91164800	0.59602700	0.52449400
C	-4.32519700	1.10927200	0.81245800
C	-5.26689100	0.79946500	-0.35746800
H	-4.33799500	-2.35403600	0.37194300
H	-4.07246100	-2.08341500	-1.33502500
H	-5.99311400	-0.91308700	-1.39923600
H	-2.29624700	0.63771700	1.42868600
H	-2.45609100	1.25541000	-0.21671700
H	-4.71950800	0.66753800	1.73347100

H	-4.27310200	2.19251700	0.97278800
O	-2.19327000	-1.61438300	1.06288600
H	-2.23372400	-2.56601600	0.86442400
C	-1.96432400	-0.94018300	-1.19365100
C	-1.31344300	-1.05104100	-2.23528700
H	-1.05419200	-1.22577600	-3.26215000
C	-6.59263000	1.57786200	-0.28227900
H	-6.36702000	2.64628200	-0.17698100
H	-7.11135600	1.45962300	-1.24773800
O	-4.53591200	1.21100800	-1.53731700
H	-5.15233300	1.25316600	-2.28630800
Au	0.37542700	-0.46344500	-0.88765800
P	2.40460900	0.10572300	0.12889300
C	2.48120400	1.89475000	0.50466600
C	2.01186800	2.81140400	-0.45260400
C	3.00957000	2.36688300	1.71632600
C	2.08453400	4.18257700	-0.20531100
H	1.58937300	2.45694600	-1.39000400
C	3.07361600	3.74187000	1.96089400
H	3.36565400	1.67119300	2.46988200
C	2.61477300	4.64917700	1.00264200
H	1.72203400	4.88458600	-0.95094000
H	3.48104800	4.10059300	2.90191800
H	2.66467300	5.71688700	1.19705900
C	2.65487300	-0.80547100	1.69411900
C	3.94404300	-1.16483500	2.12110000
C	1.54302300	-1.12109100	2.49398900
C	4.11650600	-1.82359300	3.34095600
H	4.81001900	-0.94047600	1.50521100
C	1.72385700	-1.77516300	3.71419200
H	0.53737600	-0.86830700	2.16630500
C	3.00923800	-2.12632000	4.13886600
H	5.11564800	-2.10165300	3.66445700
H	0.86026200	-2.01612000	4.32766600
H	3.14701800	-2.64078900	5.08580800
C	3.82475300	-0.28830100	-0.95783400
C	4.85104600	0.63847900	-1.19419800
C	3.88568600	-1.56063100	-1.55369400
C	5.92893800	0.29145700	-2.01500000
H	4.81390300	1.62661600	-0.74624700
C	4.96628200	-1.90259000	-2.36654700
H	3.09401700	-2.28592700	-1.37970300
C	5.98884400	-0.97566500	-2.59950200
H	6.71949200	1.01436600	-2.19563700
H	5.00856000	-2.88860200	-2.82065100
H	6.82716700	-1.24116200	-3.23743000
C	-6.50479600	-1.21032200	0.66287500
H	-6.71818400	-2.27425100	0.49421200
H	-6.02260900	-1.14962300	1.64948300
C	-7.81223400	-0.40808400	0.68394600
H	-8.45240600	-0.76248100	1.50102400
H	-8.36771300	-0.58786000	-0.24856200
C	-7.53608700	1.09407800	0.82878400
H	-7.09696300	1.29954400	1.81516800
H	-8.47265300	1.66278600	0.78501700

CP2

C	5.29959600	0.62190200	-0.49887900
C	4.24342000	1.67531100	-0.06347100
C	2.81087100	1.16760900	0.24178600
C	2.83904900	-0.04495400	1.21690900
C	4.20473600	-0.76163800	1.29973900
C	4.89513100	-0.82009200	-0.06855700
H	4.56004700	2.12325400	0.88671300
H	4.19741200	2.49479700	-0.79212900
H	5.36536700	0.60954600	-1.59657900
H	2.56882700	0.35469400	2.19915300
H	2.06406500	-0.76035200	0.93227700
H	4.86122800	-0.24974300	2.01281500
H	4.04865100	-1.77570700	1.68351900
O	2.06616500	2.19764100	0.92083800
H	2.14548800	3.03252200	0.42669000
C	2.09283500	0.89235700	-1.03167000
C	1.52445700	0.82777400	-2.12441500
H	1.35497900	0.79267000	-3.18355600
C	6.07872100	-1.80508100	-0.12946500
H	5.76104900	-2.75812100	0.31101500
H	6.28567600	-2.01024600	-1.19256200
O	3.85810200	-1.30833300	-0.95468100
H	4.26156800	-1.54780100	-1.80503100
Au	-0.26101000	0.38805100	-0.84723700
P	-2.34528200	-0.11190700	0.08858000
C	-2.31360100	-1.73216600	0.93884600
C	-1.59422800	-2.79230000	0.35999200
C	-3.00524900	-1.94012200	2.14284100
C	-1.58128400	-4.04736200	0.97008900
H	-1.04140800	-2.63946700	-0.56398800
C	-2.98292700	-3.19769300	2.75255100

H	-3.55451100	-1.12838500	2.60991900
C	-2.27542800	-4.25120300	2.16747700
H	-1.02442300	-4.86166300	0.51507600
H	-3.51785700	-3.35050800	3.68568100
H	-2.25927000	-5.22675400	2.64534600
C	-2.86187500	1.14919900	1.30741200
C	-4.22028000	1.44784400	1.50551000
C	-1.88549800	1.80285600	2.07852900
C	-4.59520600	2.38322800	2.47307500
H	-4.98349400	0.96132900	0.90510000
C	-2.26811000	2.73348800	3.04652100
H	-0.82938500	1.59636600	1.92229000
C	-3.62159700	3.02382000	3.24522100
H	-5.64706200	2.61200100	2.61973400
H	-1.50840500	3.23594800	3.63860900
H	-3.91641300	3.75267000	3.99504300
C	-3.65112800	-0.18839800	-1.19271700
C	-4.50280200	-1.29652300	-1.31279500
C	-3.79697100	0.89922400	-2.07253000
C	-5.49317000	-1.31201600	-2.30048200
H	-4.39921100	-2.14557600	-0.64441800
C	-4.79043400	0.88010400	-3.05111000
H	-3.14148000	1.76335900	-1.99093000
C	-5.63901200	-0.22709200	-3.16752600
H	-6.14889800	-2.17373400	-2.38807900
H	-4.90023300	1.72581300	-3.72413200
H	-6.40913000	-0.24258700	-3.93362100
C	6.67710300	1.06915800	0.05297900
H	6.94972300	2.02378700	-0.41635100
H	6.57992000	1.27745600	1.12897600
C	7.79027200	0.03348700	-0.13519300
H	8.71996100	0.40811400	0.30993500
H	7.99336900	-0.11724400	-1.20594400
C	7.38045900	-1.29868600	0.50340100
H	7.25622400	-1.16770300	1.58800100
H	8.16538800	-2.05285500	0.37081900

TS2

C	5.30969600	0.57562400	-0.74932900
C	3.99462100	1.28464900	-0.32575200
C	3.06705500	0.46029300	0.59215300
C	3.82878800	0.00795100	1.86301500
C	5.30221100	-0.39908600	1.59711300
C	5.56843600	-0.68480600	0.11497300
H	4.23393900	2.18316100	0.25694800
H	3.43557500	1.61879200	-1.20914000
H	5.20380600	0.22013900	-1.78573600
H	3.78666800	0.86392500	2.54397200
H	3.27674500	-0.80614000	2.33980300
H	5.97790300	0.39561200	1.92892100
H	5.55152200	-1.29007500	2.18206400
O	1.97350200	1.27112400	1.05019800
H	1.60868000	1.77054300	0.29879500
C	2.44698200	-0.69893200	-0.11392800
C	1.63321400	-1.50053900	-0.62970900
H	1.65144000	-2.46032700	-1.12335200
C	6.95496400	-1.29117200	-0.15953800
H	7.12100000	-2.12706900	0.53047800
H	6.94110100	-1.72077600	-1.17410200
O	4.55508900	-1.67964000	-0.22033100
H	4.69720700	-1.97836300	-1.13545400
Au	-0.32557700	-0.67468600	-0.31246300
P	-2.53532600	0.04748500	-0.02716700
C	-3.40126700	-0.89947000	1.27867900
C	-3.23105400	-2.29387600	1.32995900
C	-4.24352200	-0.26979100	2.20842300
C	-3.90506800	-3.04823400	2.29082700
H	-2.57285300	-2.79134900	0.62148100
C	-4.91136600	-1.03014800	3.17303100
H	-4.37726000	0.80751700	2.18798800
C	-4.74524000	-2.41676200	3.21447400
H	-3.76920200	-4.12559700	2.32303600
H	-5.55935900	-0.53575700	3.89136900
H	-5.26417200	-3.00423000	3.96675300
C	-2.58904200	1.81132600	0.46782200
C	-3.49029600	2.71524400	-0.11494500
C	-1.70380200	2.25863000	1.46548900
C	-3.50948800	4.05003900	0.30203700
H	-4.17342400	2.38640900	-0.89203300
C	-1.73300800	3.59030400	1.88180900
H	-0.99366900	1.57066600	1.91873200
C	-2.63517600	4.48811100	1.29961800
H	-4.20927400	4.74436700	-0.15466100
H	-1.05024300	3.92648100	2.65726300
H	-2.65335100	5.52566000	1.62145300
C	-3.52816600	-0.11828200	-1.55594000
C	-4.88474100	-0.47761800	-1.51048600
C	-2.92216400	0.14367900	-2.79661400

C	-5.62452700	-0.56548900	-2.69308500
H	-5.36391300	-0.69565700	-0.56080300
C	-3.66730000	0.06090000	-3.97379100
H	-1.86853700	0.40843000	-2.84437700
C	-5.01933100	-0.29457500	-3.92322700
H	-6.67263900	-0.84819900	-2.65047100
H	-3.19148700	0.26540500	-4.92882800
H	-5.59682500	-0.36636500	-4.84065400
C	6.49276000	1.56925400	-0.72124900
H	6.28695500	2.38432600	-1.42743600
H	6.54816500	2.03505200	0.27372400
C	7.83872900	0.90614900	-1.03937400
H	8.64358000	1.64699000	-0.96375700
H	7.84077200	0.54750800	-2.07942500
C	8.10206100	-0.27074000	-0.09074100
H	8.21944400	0.09955300	0.93728400
H	9.04366400	-0.77038300	-0.34694300

CP3

C	-5.10662900	0.24897200	0.94744500
C	-3.91034500	1.22032000	0.77581400
C	-3.13184400	0.90655500	-0.52773300
C	-4.04276500	1.22549700	-1.73316400
C	-5.42755600	0.54179100	-1.57479700
C	-5.41862900	-0.42752300	-0.39582800
H	-4.25473400	2.25964800	0.71315400
H	-3.23380300	1.15355700	1.63433800
H	-4.82299100	-0.55320200	1.64583300
H	-4.14235900	2.31366000	-1.78635100
H	-3.54545800	0.89957900	-2.65103800
H	-6.21234800	1.28252300	-1.39106700
H	-5.70925200	0.00443800	-2.48573800
O	-1.98514800	1.71574200	-0.66074000
H	-1.27433900	1.38041000	-0.08283200
C	-2.78791600	-0.57187300	-0.53865300
C	-1.71748600	-1.32905300	-0.41548000
H	-1.85658200	-2.41309600	-0.45140000
C	-6.58629900	-1.40266300	-0.32421100
H	-6.78116800	-1.83594800	-1.31182000
H	-6.32813300	-2.23030600	0.35536100
O	-4.15556900	-1.29384100	-0.72405700
H	-4.15183200	-2.11847700	-0.19776000
Au	0.24091000	-0.69660400	-0.18044100
P	2.48990400	-0.00154100	0.07407900
C	3.52215200	-0.39026200	-1.39155300
C	3.34602600	-1.63063600	-2.02844600
C	4.48775500	0.50442700	-1.87858400
C	4.13403100	-1.97553400	-3.12752600
H	2.59343100	-2.32746400	-1.66702900
C	5.26990900	0.15749700	-2.98420300
H	4.62849300	1.47108600	-1.40429900
C	5.09642100	-1.08093800	-3.60758100
H	3.99186600	-2.93734400	-3.61249700
H	6.01291800	0.85726400	-3.35685300
H	5.70481200	-1.34686900	-4.46764000
C	2.61165500	1.81384100	0.32089300
C	3.41423600	2.38003300	1.32256000
C	1.86690200	2.65495100	-0.52545600
C	3.47460300	3.76954600	1.47082500
H	3.98944100	1.74479800	1.98910900
C	1.93612600	4.04087300	-0.37797400
H	1.23506500	2.23005700	-1.30236500
C	2.73941600	4.60035900	0.62202400
H	4.09773300	4.19901300	2.25051200
H	1.35930900	4.68179700	-1.03902600
H	2.78844400	5.67938200	0.73986200
C	3.31988600	-0.78137800	1.51169700
C	4.67884800	-1.13227700	1.48105700
C	2.57333100	-1.01563200	2.67916500
C	5.28157100	-1.70172500	2.60630700
H	5.26661200	-0.97087800	0.58235600
C	3.18112700	-1.57787200	3.80310100
H	1.51616200	-0.76254300	2.70850100
C	4.53629000	-1.92216600	3.76781300
H	6.33286900	-1.97405600	2.57222400
H	2.59608400	-1.75400400	4.70162700
H	5.00741200	-2.36669100	4.64016300
C	-6.37487400	0.92042500	1.51823900
H	-6.13735500	1.32246700	2.51047600
H	-6.64334600	1.78468200	0.89441700
C	-7.56130000	-0.05164100	1.59681800
H	-8.45321500	0.47814400	1.95030000
H	-7.34920300	-0.83426000	2.33984100
C	-7.84429000	-0.70235100	0.23466200
H	-8.19387500	0.05547300	-0.47910000
H	-8.64752400	-1.44258100	0.31741800

CP4

C	5.34975800	-0.53796700	0.26975800
C	4.63399700	-1.86160800	-0.10677700
C	3.11353900	-1.60860500	-0.26963000
C	2.91081300	-0.65551800	-1.46978400
C	3.61197100	0.69230800	-1.17179200
C	4.33144300	0.62054300	0.18567200
H	5.00081900	-2.26330300	-1.05897600
H	4.80767800	-2.62736100	0.66009200
H	5.65102100	-0.59027700	1.32446000
H	3.32301600	-1.15106100	-2.35431100
H	1.83774400	-0.52417800	-1.63929100
H	4.33330600	0.94208800	-1.95404700
H	2.88908200	1.51398700	-1.13487300
O	2.36853800	-2.78457800	-0.50414400
H	2.79861700	-3.54552700	-0.08107300
C	2.64540100	-0.85987300	0.96982700
C	1.64223300	-1.28226900	1.85099800
H	1.62838500	-0.79399300	2.82523200
C	4.94877800	1.93880700	0.64250900
H	4.21450800	2.74819100	0.54977700
H	5.19630100	1.85029300	1.70837100
O	3.27266900	0.28015600	1.18223900
Au	-0.17777800	-0.55715000	0.84482500
P	-2.21518100	0.14983200	-0.08126500
C	-1.99495000	1.62155100	-1.15371200
C	-1.27878500	2.72090100	-0.64683100
C	-2.51505800	1.67742700	-2.45533300
C	-1.10059700	3.86346100	-1.42708100
H	-0.86600600	2.68775800	0.35896400
C	-2.32634200	2.82258500	-3.23649400
H	-3.06520500	0.83514100	-2.86316200
C	-1.62322300	3.91533300	-2.72476300
H	-0.55475800	4.71208200	-1.02378800
H	-2.73249600	2.85723500	-4.24360500
H	-1.48111300	4.80446900	-3.33280200
C	-2.99691500	-1.15274500	-1.10408300
C	-4.39338800	-1.24143400	-1.22824200
C	-2.18161400	-2.06874400	-1.79004900
C	-4.96234700	-2.22771300	-2.03777700
H	-5.03790800	-0.55178400	-0.69111600
C	-2.75556800	-3.04947600	-2.60154100
H	-1.10053900	-2.02361200	-1.68448600
C	-4.14592400	-3.12972500	-2.72653100
H	-6.04329800	-2.29248100	-2.12565000
H	-2.11764800	-3.75467200	-3.12699600
H	-4.59203500	-3.89774800	-3.35227500
C	-3.43599500	0.61719900	1.20215300
C	-4.28793700	1.72196900	1.04530600
C	-3.53535900	-0.17508400	2.35883000
C	-5.23010600	2.02422600	2.03297000
H	-4.21686200	2.34992100	0.16223200
C	-4.48190700	0.12736100	3.33844400
H	-2.87501200	-1.02819800	2.49558900
C	-5.32976400	1.22836100	3.17709200
H	-5.88375500	2.88272900	1.90597200
H	-4.55281900	-0.49113400	4.22879000
H	-6.06178200	1.46728900	3.94352800
H	1.43167900	-2.35056500	1.84419400
C	6.61238600	-0.22834900	-0.55327200
H	7.33146200	-1.04755700	-0.42772200
H	6.36528300	-0.19820700	-1.62440000
C	6.22555700	2.26118700	-0.15979500
H	5.96497700	2.48613200	-1.20313400
H	6.67607200	3.17469600	0.24461400
C	7.24052900	1.10605600	-0.11571600
H	7.62750200	1.00185200	0.90823100
H	8.10257900	1.33958200	-0.75140100

CP5

C	3.79833700	0.70115700	0.68821200
C	3.14804300	-0.46797900	1.46942700
C	3.15271200	-1.74878700	0.59866500
C	4.62289000	-2.19335800	0.39637300
C	5.41052900	-1.04383500	-0.28133600
C	4.46722800	0.13488100	-0.58281200
H	3.69981200	-0.69688200	2.38886400
H	2.11909600	-0.21898100	1.75731100
H	3.00653600	1.37437700	0.33207100
H	5.02207500	-2.44582200	1.38334700
H	4.64286900	-3.10825100	-0.20365900
H	6.22611500	-0.69631900	0.35865800
H	5.86483600	-1.37759000	-1.21982500
O	2.44913000	-2.82408100	1.17504200
H	1.58909600	-2.51498600	1.50689900
C	2.65477100	-1.38881400	-0.79031900
C	1.57430100	-1.99775900	-1.45007000
H	1.34798000	-3.00663100	-1.10483000
C	5.08207900	1.23716900	-1.43943000

H	5.57538100	0.79872400	-2.31498600
H	4.26986200	1.87463300	-1.81312000
O	3.35332700	-0.45200100	-1.39223000
Au	-0.15065600	-0.88922100	-0.67858500
P	-2.11127400	0.20029400	0.04029900
C	-1.87673300	0.99523900	1.67761800
C	-1.52337800	0.18952200	2.77553200
C	-2.00366000	2.38087500	1.85272700
C	-1.31304500	0.76238600	4.02981300
H	-1.42847200	-0.88750900	2.65604700
C	-1.78472100	2.95144200	3.11153100
H	-2.27569900	3.01713900	1.01639200
C	-1.44157800	2.14630300	4.19942900
H	-1.05314600	0.13038300	4.87468300
H	-1.88922000	4.02543400	3.23820700
H	-1.27747700	2.59155000	5.17675500
C	-2.63625100	1.51098900	-1.12584400
C	-3.98886200	1.86597500	-1.25870000
C	-1.66674000	2.18343600	-1.88855400
C	-4.36106900	2.88695100	-2.13617700
H	-4.75223000	1.34511700	-0.68844400
C	-2.04355000	3.20654500	-2.76120000
H	-0.61919600	1.90350300	-1.80714700
C	-3.39076800	3.55904100	-2.88570000
H	-5.40960400	3.15290400	-2.23659800
H	-1.28780200	3.72014400	-3.34890900
H	-3.68457800	4.34968400	-3.57043900
C	-3.52098200	-0.95561100	0.21607200
C	-4.48241900	-0.80315100	1.22839000
C	-3.65711800	-1.99848600	-0.71618900
C	-5.56598700	-1.68292300	1.30153100
H	-4.38798600	-0.00810100	1.96219400
C	-4.74434700	-2.87046500	-0.64213100
H	-2.91502200	-2.13093200	-1.49996200
C	-5.69925800	-2.71436400	0.36783600
H	-6.30409300	-1.56067200	2.08935000
H	-4.84158400	-3.67359900	-1.36715900
H	-6.54182500	-3.39745300	0.42921500
H	1.53963900	-1.84768200	-2.52953300
C	4.78589100	1.54894700	1.50981900
H	4.25528700	1.98245300	2.36697000
H	5.57151600	0.90470400	1.92966800
C	6.07006200	2.09118600	-0.62052000
H	6.94899900	1.49044400	-0.34986000
H	6.44106400	2.90664500	-1.25166200
C	5.41707500	2.65745300	0.65124500
H	4.63986200	3.38272600	0.36888300
H	6.15847300	3.20975300	1.24043000

TS3

C	4.71328100	0.57259100	0.90889600
C	3.98870100	-0.52530600	1.71450200
C	2.98759900	-1.25766600	0.87119700
C	4.29126200	-2.26181100	-0.46835000
C	5.42940600	-1.28437800	-0.72398200
C	4.84017400	0.14904300	-0.57046900
H	4.69102300	-1.25003600	2.15105300
H	3.43849700	-0.10084700	2.56308000
H	4.07094300	1.46445500	0.89924900
H	4.41850500	-2.94531800	0.37245200
H	3.93831500	-2.82128800	-1.33449100
H	6.26222400	-1.44617600	-0.03480300
H	5.81163400	-1.40726900	-1.74181900
O	2.17326600	-2.07394500	1.51313000
H	1.36534100	-2.23615400	0.96991600
C	2.83648000	-1.04760500	-0.53202300
C	1.63738900	-1.54604000	-1.26545200
H	1.57037300	-2.63921200	-1.21158200
C	5.54523700	1.22419600	-1.39286800
H	5.64936300	0.88208900	-2.42923300
H	4.89990800	2.11239000	-1.41338100
O	3.49088800	0.00398800	-1.10544400
Au	-0.21316100	-0.73361600	-0.61083100
P	-2.31367600	0.17829800	0.03172000
C	-3.65187300	-1.07447600	0.10355800
C	-3.67937400	-2.08650500	-0.87164900
C	-4.65618700	-1.03499600	1.08387200
C	-4.70329900	-3.03530700	-0.87319800
H	-2.90175800	-2.13415900	-1.63041100
C	-5.67549400	-1.99170400	1.08271100
H	-4.64451900	-0.26600300	1.85053400
C	-5.70221400	-2.98976600	0.10493500
H	-4.71742400	-3.81193700	-1.63289400
H	-6.44731800	-1.95477700	1.84661500
H	-6.49552500	-3.73216800	0.10690500
C	-2.26587800	0.95968000	1.69433200
C	-2.72392900	2.26569700	1.92073400
C	-1.74158600	0.21814300	2.76818200

C	-2.66403100	2.81846800	3.20463900
H	-3.12701100	2.85402500	1.10236000
C	-1.69159300	0.76965900	4.04874600
H	-1.38222300	-0.79567300	2.60665400
C	-2.15142200	2.07328700	4.26870000
H	-3.02203200	3.83103100	3.36950400
H	-1.29510000	0.18384200	4.87371500
H	-2.10995100	2.50393400	5.26539300
C	-2.88280300	1.47489100	-1.13463000
C	-4.24309000	1.66093600	-1.42882900
C	-1.92665300	2.31001500	-1.73772500
C	-4.63851400	2.67326300	-2.30730500
H	-4.99393500	1.01598100	-0.98231100
C	-2.32668600	3.32398100	-2.61030400
H	-0.86905900	2.16606400	-1.52968000
C	-3.68332000	3.50659200	-2.89639800
H	-5.69301900	2.80735500	-2.53218700
H	-1.57990500	3.96424600	-3.07177400
H	-3.99401700	4.29096000	-3.58104500
H	1.74857700	-1.28188800	-2.32154100
C	6.06657100	0.96988400	1.52378800
H	5.90575000	1.33416300	2.54663100
H	6.70929200	0.08156000	1.61275300
C	6.91175400	1.59514200	-0.78840000
H	7.59763700	0.73829000	-0.85445800
H	7.36293400	2.39465900	-1.38668600
C	6.77598900	2.03828000	0.67705600
H	6.20532600	2.97708300	0.72221800
H	7.76339400	2.25255300	1.10200300

CP6

C	-4.95262900	0.40778100	-0.95852300
C	-4.32785600	-0.78751300	-1.75807700
C	-3.03277000	-1.11919900	-1.11560200
C	-4.17867500	-2.20489500	0.87172700
C	-5.34334900	-1.19809000	1.03179900
C	-4.76500000	0.15766600	0.55512900
H	-4.99768600	-1.65410900	-1.68748600
H	-4.18225400	-0.53984200	-2.81295900
H	-4.34392800	1.29161200	-1.19520400
H	-4.40384000	-3.02539200	0.18111600
H	-3.90801200	-2.65952300	1.82778800
H	-6.23316400	-1.49215700	0.46858600
H	-5.64096400	-1.12289000	2.08171900
O	-1.97327600	-1.01482000	-1.81609100
H	-1.14816400	-1.12811000	-1.21743300
C	-2.98051100	-1.35084400	0.38636300
C	-1.62707500	-1.76949600	0.93609500
H	-1.51153100	-2.84671300	0.75458200
C	-5.25500800	1.37618200	1.33995700
H	-5.13394000	1.19285900	2.41370000
H	-4.61923900	2.23459300	1.08593300
O	-3.32721000	0.01115300	0.76532100
Au	0.19376500	-0.81229800	0.43943800
P	2.32177400	0.18663400	-0.01343700
C	2.44966200	1.90949900	0.60234300
C	1.30895300	2.72962600	0.56419400
C	3.65529100	2.42945200	1.10034100
C	1.37814500	4.05353900	1.00229400
H	0.36505000	2.33309500	0.19786900
C	3.71809500	3.75302100	1.54420400
H	4.54218300	1.80495400	1.15121600
C	2.58282300	4.56682300	1.49313100
H	0.49100000	4.68004500	0.96875200
H	4.65424000	4.14557300	1.93161900
H	2.63459100	5.59481900	1.84108400
C	3.70047400	-0.73980600	0.76505600
C	4.92210100	-0.95616500	0.10852900
C	3.52352700	-1.22959700	2.07082100
C	5.95215600	-1.64727200	0.75360400
H	5.07277300	-0.59411700	-0.90411000
C	4.55709400	-1.91233800	2.71375900
H	2.57953200	-1.07647900	2.58908500
C	5.77293200	-2.12347700	2.05493300
H	6.89350700	-1.81215800	0.23662600
H	4.41099400	-2.28417700	3.72412300
H	6.57507200	-2.66096100	2.55306100
C	2.69366300	0.24138500	-1.81074400
C	3.15023800	1.40562100	-2.44604900
C	2.50661100	-0.92914200	-2.56785300
C	3.42140800	1.39579800	-3.81849400
H	3.29485700	2.31930900	-1.87775800
C	2.78618700	-0.93658800	-3.93480900
H	2.15141600	-1.83844400	-2.08785000
C	3.24291200	0.22772700	-4.56306900
H	3.77479300	2.30263800	-4.30158500
H	2.64699100	-1.84849300	-4.50910400
H	3.45726800	0.22263500	-5.62820800

H	-1.68979900	-1.64242900	2.02432600
C	-6.40541700	0.71495600	-1.34203400
H	-6.46860600	0.93814600	-2.41456300
H	-7.03911700	-0.16620700	-1.16889200
C	-6.72346800	1.69202600	0.99395500
H	-7.03820000	2.58547900	1.54508300
H	-7.37032900	0.87328300	1.33979700
C	-6.92462300	1.90722500	-0.51558100
H	-7.98568900	2.07190200	-0.73613600
H	-6.39532000	2.81915800	-0.82703000

9

C	0.39923900	-0.31872000	0.83355400
C	-0.60972400	-1.19949600	0.06833700
C	-1.86138300	-0.43126700	-0.42077000
C	-1.41031600	0.78316800	-1.26540600
C	-0.41460500	1.67996900	-0.51986700
C	0.82226000	0.93674000	0.01522200
H	-0.14073600	-1.64088500	-0.82251200
H	-0.93380100	-2.03347400	0.70164800
H	-0.11139900	0.06036900	1.72840700
H	-0.95525500	0.38882700	-2.18626600
H	-2.29201200	1.35796400	-1.56854500
H	-0.08286300	2.49739600	-1.17243200
H	-0.91371400	2.14605600	0.33808300
O	-2.70574200	-1.30069500	-1.19866700
H	-2.20030900	-1.58330700	-1.97943200
C	-2.68600800	0.00553100	0.71748500
C	-3.34429000	0.38205900	1.65889500
H	-3.93472300	0.71071700	2.48504800
O	1.44121700	1.90168500	0.89401400
H	2.27638100	1.54224200	1.23117400
C	1.80734300	0.56815400	-1.11654800
H	2.13406700	1.49737000	-1.60032700
H	1.28374200	-0.01943400	-1.88266500
C	1.62801200	-1.13567500	1.29677600
H	2.17827700	-0.56010800	2.05667900
H	1.28646100	-2.04566100	1.80803600
C	3.02156500	-0.24013400	-0.62556900
H	3.65160800	-0.51585700	-1.48090500
H	3.65800000	0.38701400	0.01878700
C	2.58833100	-1.49411000	0.14994300
H	2.10274500	-2.19849400	-0.54048600
H	3.46718800	-2.01596900	0.55061300

10

C	0.74475700	0.96195000	-0.27428100
C	-0.48087200	1.72910800	0.26239400
C	-1.80655900	1.05349700	-0.07854800
C	-1.37731600	-0.97968500	1.35899300
C	0.17155500	-0.99335100	1.32644000
C	0.53203000	-0.56478000	-0.12581200
H	-0.43026200	1.78530800	1.36185200
H	-0.51263900	2.76168200	-0.10141800
H	0.79341900	1.13769600	-1.35935700
H	-1.78666300	-0.34009000	2.14942600
H	-1.77956000	-1.98642700	1.51349900
H	0.61084700	-0.33036300	2.07803300
H	0.55533500	-1.99986600	1.52324600
O	-2.81544100	1.68957800	-0.32484900
C	-1.77729600	-0.49112100	-0.06029300
C	-3.04768300	-1.10550900	-0.61854500
H	-2.94583300	-2.19544800	-0.64353400
C	1.71359200	-1.32197900	-0.73334300
H	1.55889900	-2.40091500	-0.60714200
H	1.72773500	-1.12675000	-1.81443500
O	-0.65710400	-0.89516100	-0.87662200
H	-3.24296900	-0.74356500	-1.63212700
C	2.07643600	1.42935000	0.33588800
H	2.20813500	2.50435300	0.15179500
H	2.04942600	1.30673200	1.42928800
C	3.04925000	-0.87912300	-0.10986800
H	3.87532600	-1.41971800	-0.58906200
H	3.07236000	-1.16006800	0.95364100
C	3.26199600	0.63858000	-0.24251400
H	4.19337600	0.93581100	0.25662300
H	3.38115300	0.89390100	-1.30600100
H	-3.90706000	-0.83923800	0.00438400

TS4

C	5.42450000	-0.32572800	0.83758100
C	4.41093800	-1.44410500	0.98583000
C	2.93590600	-1.35750600	-0.57797500
C	3.82799800	-1.20091000	-1.76587900
C	4.74086600	0.04467100	-1.66258200
C	4.84197400	0.62733000	-0.23749300
H	4.64901700	-2.36101000	0.44226300
H	4.09205600	-1.69855100	1.99599100

H	5.40022200	0.25523600	1.76861800
H	4.39247100	-2.13123900	-1.90637900
H	3.16522600	-1.13027800	-2.63836000
H	5.73172500	-0.19357900	-2.05571700
H	4.33500900	0.83981000	-2.29657900
O	2.13411600	-2.40053500	-0.58194400
H	1.38354300	-2.24823200	0.04306300
C	2.89664400	-0.40967400	0.48670600
C	1.79828000	-0.41735800	1.49761500
H	1.96814900	0.41169100	2.19122100
C	5.60660100	1.94787300	-0.21694000
H	5.21503300	2.63485400	-0.97646600
H	5.44742500	2.42150800	0.76001300
O	3.48168200	0.81396400	0.25099100
Au	-0.16361100	-0.17850000	0.71800800
P	-2.39828100	0.10858600	-0.04892500
C	-3.57703300	0.31616100	1.34122900
C	-3.36990900	-0.42389100	2.51816100
C	-4.67904100	1.18211000	1.25703200
C	-4.26013600	-0.31055300	3.58757700
H	-2.51169700	-1.08659200	2.60091400
C	-5.56365500	1.29777500	2.33293500
H	-4.84678100	1.77108100	0.36025100
C	-5.35770300	0.55146100	3.49659000
H	-4.09230300	-0.88796500	4.49253800
H	-6.41173600	1.97306800	2.26029600
H	-6.04608400	0.64532700	4.33194000
C	-2.59874000	1.58571100	-1.11855700
C	-3.43055000	1.58169600	-2.24922500
C	-1.90949900	2.76122000	-0.77349000
C	-3.57311400	2.74001800	-3.01921600
H	-3.96367600	0.67966500	-2.53444600
C	-2.06130700	3.91758000	-1.54022000
H	-1.25551400	2.77529500	0.09530900
C	-2.89224300	3.90798600	-2.66561300
H	-4.21807100	2.72725200	-3.89360400
H	-1.52805800	4.82254700	-1.26222500
H	-3.00594900	4.80679800	-3.26545000
C	-3.00141900	-1.31914900	-1.03386900
C	-4.23587300	-1.93525200	-0.78067900
C	-2.18699900	-1.80992600	-2.06987700
C	-4.65029400	-3.02283800	-1.55692400
H	-4.87476800	-1.57403500	0.01919600
C	-2.60781900	-2.88915000	-2.84750500

H	-1.22575400	-1.34344000	-2.27369000
C	-3.84082900	-3.49932800	-2.59036900
H	-5.60752700	-3.49407300	-1.35149400
H	-1.97407300	-3.25660000	-3.65013700
H	-4.16604200	-4.34353900	-3.19202600
H	1.81283800	-1.34141100	2.08701200
C	6.89423200	-0.67247800	0.55595800
H	7.28688900	-1.33273900	1.33824800
H	6.99113400	-1.21892200	-0.39268900
C	7.11533400	1.69183300	-0.44356700
H	7.28524600	1.37723100	-1.48202800
H	7.65448400	2.63860500	-0.32607500
C	7.70485500	0.64107800	0.51839100
H	7.73501200	1.05904400	1.53463100
H	8.74384700	0.42853800	0.24095900

CP7

C	-4.94853100	1.09869100	-0.11496200
C	-3.45905600	0.96799400	-0.47842900
C	-2.90481100	-1.49333100	-0.37241000
C	-4.03835000	-1.90442500	-1.23927500
C	-5.42057300	-1.42161000	-0.72628700
C	-5.33111000	-0.31577500	0.35859000
H	-3.30467000	0.76181300	-1.54406500
H	-2.88360500	1.86498600	-0.23760600
H	-4.98766600	1.71264900	0.79567800
H	-3.81223100	-1.45074400	-2.21885400
H	-4.00297200	-2.98688400	-1.39685900
H	-6.04016500	-1.11406200	-1.57179300
H	-5.91906500	-2.28259800	-0.26822300
O	-1.88908600	-2.26578200	-0.30863400
H	-1.13695800	-1.78521300	0.17505400
C	-2.99597600	-0.22315500	0.42290300
C	-1.84271000	0.02317500	1.37018800
H	-1.90632400	-0.69769200	2.19484900
C	-6.62819300	-0.23591000	1.16487100
H	-6.92879100	-1.22337700	1.53417100
H	-6.45470400	0.40033300	2.04130800
O	-4.18738300	-0.66245400	1.18351300
Au	0.14305200	0.02757400	0.63146900
P	2.43499700	0.08839300	-0.05375300
C	2.88893700	1.69813800	-0.80979200
C	1.97514400	2.31548100	-1.68165000
C	4.11736800	2.32317900	-0.54508500

C	2.29310100	3.53003500	-2.29172300
H	1.01506600	1.84661700	-1.88515600
C	4.42838800	3.54365300	-1.15173900
H	4.82886800	1.86620200	0.13574000
C	3.52046700	4.14649600	-2.02624200
H	1.58196700	3.99799400	-2.96699700
H	5.38043900	4.02182500	-0.93864500
H	3.76525300	5.09516800	-2.49587300
C	3.58292000	-0.16905500	1.35295600
C	4.78697800	-0.87713800	1.21054700
C	3.24822900	0.37878000	2.60335500
C	5.64400800	-1.02878200	2.30426300
H	5.05569300	-1.31498800	0.25388200
C	4.11070800	0.23129500	3.69102500
H	2.31404700	0.92113000	2.72972200
C	5.30910500	-0.47441500	3.54275700
H	6.57211100	-1.58135000	2.18662700
H	3.84377000	0.65960500	4.65311100
H	5.97690300	-0.59605800	4.39113500
C	2.85308000	-1.20285300	-1.29115400
C	3.54032600	-0.90731400	-2.47755000
C	2.46557200	-2.52994800	-1.03213100
C	3.83990900	-1.92634000	-3.38808100
H	3.84418000	0.11184100	-2.69533100
C	2.77184600	-3.54429800	-1.93982900
H	1.93511400	-2.77524800	-0.11457800
C	3.45870700	-3.24321000	-3.12161500
H	4.37412900	-1.68690100	-4.30343300
H	2.47510000	-4.56743900	-1.72561700
H	3.69495700	-4.03271200	-3.82963900
H	-1.97168600	1.01841500	1.81091900
C	-5.97212100	1.68792700	-1.08582400
H	-5.68388600	2.70273900	-1.38618200
H	-6.02686900	1.09407500	-2.00936800
C	-7.73859000	0.37333700	0.27264900
H	-8.01136800	-0.34404800	-0.51339900
H	-8.64167700	0.51119100	0.87814500
C	-7.34317400	1.72083600	-0.37012000
H	-7.30376300	2.49126900	0.41292500
H	-8.12512700	2.03433900	-1.07165000

10’

C	0.77574200	-0.90830600	-0.46897500
C	-0.64605000	-1.22856500	-0.96498900
C	-2.17051400	0.66595800	-0.29694900
C	-1.14671200	1.70341100	-0.74482500
C	0.20199500	1.62497200	0.00669800
C	0.55192300	0.21990500	0.55934200
H	-0.87452200	-0.76618200	-1.93236500
H	-0.80725900	-2.30548500	-1.07906600
H	1.08392900	-1.77062000	0.13996800
H	-0.99421900	1.53549700	-1.82085400
H	-1.60011700	2.69557100	-0.65454600
H	1.00254300	2.02047100	-0.62720500
H	0.14529300	2.28177100	0.88371500
O	-3.37062100	0.86394200	-0.34892700
C	-1.54783900	-0.65169600	0.18421400
C	-2.56078900	-1.62355700	0.75712100
H	-3.10577500	-1.16937600	1.58972600
C	1.77606200	0.28631400	1.47418900
H	1.66578600	1.07426200	2.22987300
H	1.85879000	-0.66697800	2.01256600
O	-0.62543800	-0.25497600	1.24339900
H	-2.04773200	-2.52211200	1.11626800
C	1.93852100	-0.61384600	-1.41549700
H	2.07756400	-1.42975100	-2.13709200
H	1.74800800	0.29512000	-2.00384400
C	3.04782600	0.53243800	0.62375000
H	3.03100200	1.55986000	0.23599600
H	3.93068600	0.47182600	1.27278300
C	3.21413900	-0.45449000	-0.55438000
H	3.47889900	-1.44393300	-0.15318500
H	4.05955900	-0.13798900	-1.17881900
H	-3.28977600	-1.90890900	-0.00788500

Supplementary Methods

Procedure A:

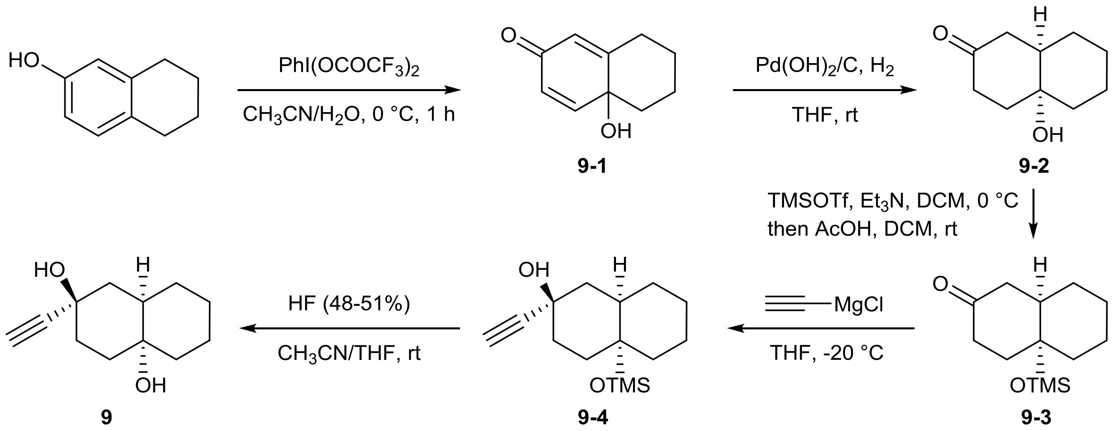

Compound 9-1. To a stirred solution of 5,6,7,8-tetrahydro-2-naphthol (8.00 g, 54.0 mmol, 1.0 equiv) in CH_3CN/H_2O (150 mL/60 mL) at 0 °C was added $PhI(OCOCF_3)_2$ (25.5 g, 59.4 mmol, 1.1 equiv). The reaction mixture was stirred at 0 °C for 1 h, and then diluted with addition of water (60 mL). The mixture was extracted with diethyl ether (150 mL × 4), and the combined organic layers were washed with brine, dried over Na_2SO_4, filtered, and concentrated *in vacuo*. Purification by flash column chromatography afforded the desired known ketone **9-1**[5] (4.60 g, 28.1 mmol) in 52% yield; R_f = 0.60 (silica gel, ethyl acetate/hexanes = 1/1).

Compound 9-2. Ketone **9-1** (2.80 g, 17.1 mmol, 1.0 equiv) was dissolved in anhydrous THF (25 mL), and $Pd(OH)_2/C$ (240 mg, 1.71 mmol, 0.10 equiv, palladium 10% on carbon) was added. The reaction mixture was degassed by hydrogen and stirred at ambient temperature under hydrogen balloon overnight. The mixture was then filtered through celite, washed through with ethyl acetate (20 mL × 3), and the filtrate was concentrated *in vacuo*. The residue was purified by flash column chromatography to afford **9-2** (1.75 g, 10.4 mmol) in 61% yield; R_f = 0.55 (silica gel, ethyl acetate/hexanes = 1/1). The relative stereochemistry of **9-2** was confirmed according to literature[6]. ^1H NMR (400 MHz, $CDCl_3$) δ = 2.99 (dd, *J* = 13.8, 5.9 Hz, 1H), 2.82 – 2.69 (m, 1H), 2.33 – 2.16 (m, 2H), 2.07 – 1.99 (m, 1H), 1.99 – 1.91 (m, 1H), 1.86 – 1.77 (m, 1H), 1.73 – 1.67 (m, 3H), 1.54 – 1.45 (m, 1H), 1.45 – 1.35 (m, 2H), 1.35 – 1.11 (m, 3H); ^{13}C NMR (100 MHz, $CDCl_3$) δ = 211.9, 71.0, 45.9, 43.4, 39.7, 37.0, 32.7, 30.4, 25.2, 23.6 ppm; IR ν_{max} (film): 3406, 2928, 2859, 1701, 1612, 1498, 1452, 1373, 1244, 1155, 1045, 957, 922, 860, 737 cm^{-1}; HRMS (ESI) m/z calcd for $C_{10}H_{16}O_2Na$ $[M+Na]^+$: 191.1043; found: 191.1041.

Compound 9-3. To a solution of **9-2** (2.02 g, 12.0 mmol, 1.0 equiv) in anhydrous DCM (100 mL) at 0 °C was added dried triethylamine (10.1 mL, 72.0 mmol, 6.0 equiv) followed by the addition of TMSOTf (6.6 mL, 36.0 mmol, 3.0 equiv). The reaction mixture was warmed up to ambient temperature slowly during 3 h, and then quenched with water (40 mL), extracted with DCM (50 mL × 2), and the combined organic layers were dried over Na_2SO_4, filtered, and concentrated *in vacuo*. The residue was redissolved in DCM (50 mL), and acetic acid (3.0 mL) was added. The mixture was stirred at ambient temperature for 4 h, and then quenched by careful addition of saturated aqueous sodium bicarbonate (30 mL) at 0 °C. The mixture was extracted with DCM (50 mL × 2), and the

combined organic layers were dried over Na_2SO_4, filtered, and concentrated *in vacuo*. The residue was purified by flash column chromatography to afford **9-3** (2.25 g, 9.36 mmol) in 78% yield; R_f = 0.65 (silica gel, ethyl acetate/hexanes = 1/8). ^{1}H NMR (400 MHz, $CDCl_3$) δ = 2.96 (dd, *J* = 13.8, 6.0 Hz, 1H), 2.69 (dt, *J* = 13.6, 6.8 Hz, 1H), 2.28 – 2.18 (m, 1H), 2.17 – 2.08 (m, 1H), 1.99 – 1.88 (m, 2H), 1.87 – 1.81 (m, 1H), 1.71 – 1.53 (m, 5H), 1.40 – 1.28 (m, 1H), 1.28 – 1.04 (m, 2H), 0.16 (s, 9H); ^{13}C NMR (100 MHz, $CDCl_3$) δ = 212.5, 74.5, 46.7, 43.6, 39.1, 37.1, 32.6, 30.5, 25.5, 23.7, 2.6 ppm; IR ν_{max} (film): 2934, 2859, 1707, 1449, 1260, 1250, 1136, 1067, 991, 887, 841, 752 cm^{-1}; HRMS (ESI) m/z calcd for $C_{13}H_{24}O_2NaSi$ $[M+Na]^+$: 263.1438; found: 263.1441.

Compound 9-4. Ketone **9-3** (800 mg, 3.34 mmol, 1.0 equiv) was dissolved in anhydrous THF (24 mL), and cooled to -20 °C. Ethynylmagnesium chloride (16.8 mL, 10.0 mmol, 3.0 equiv, 0.6 M in THF) was added, and the reaction mixture was warmed up to ambient temperature slowly during 4 h. The reaction was quenched with saturated aqueous ammonium chloride (25 mL), extracted with ethyl acetate (30 mL × 3), and the combined organic layers were dried over Na_2SO_4, filtered, and concentrated *in vacuo*. Purification by flash column chromatography afforded the desired product **9-4** (755 mg, 2.84 mmol) as a single isomer in 85% yield; R_f = 0.30 (silica gel, ethyl acetate/hexanes = 1/8). ^{1}H NMR (500 MHz, $CDCl_3$) δ = 2.52 (s, 1H), 2.33 (br, 1H), 2.03 – 1.80 (m, 5H), 1.81 – 1.72 (m, 2H), 1.70 – 1.60 (m, 3H), 1.46 – 1.13 (m, 5H), 0.12 (s, 9H); ^{13}C NMR (100 MHz, $CDCl_3$) δ = 87.3, 74.4, 73.0, 69.5, 41.9, 40.0, 38.2, 37.3, 31.4, 26.8, 21.7, 20.3, 2.7 ppm; IR ν_{max} (film): 3310, 2936, 2826, 1736, 1449, 1250, 1148, 1074, 953, 837, 754 cm^{-1}; NOTE: ^{13}C NMR substituent-induced shielding effects for some ethynyl-substituted bicycle[4.4.0] substrates were observed.[7,8] This kind of shielding effects were also observed for compounds **9-6**, **9-7**, **9**, **11a-g** and **11l**. HRMS (ESI) m/z calcd for $C_{15}H_{26}O_2NaSi$ $[M+Na]^+$: 289.1594; found: 289.1593.

Compound 9. To a stirred solution of **9-4** (450 mg, 1.69 mmol, 1.0 equiv) in CH_3CN/THF (6.0 mL/6.0 mL) in plastic tube at ambient temperature was added HF (1.2 mL, 48-51% solution in water) dropwise. The reaction was stirred for 2 h, and then quenched by careful addition of saturated aqueous sodium bicarbonate (15 mL). The mixture was extracted with ethyl acetate (20 mL × 3), and the combined organic layers were dried over Na_2SO_4, filtered, and concentrated *in vacuo*. The residue was purified by flash column chromatography to afford alkyndiol **9** (263 mg, 1.36 mmol) in 81% yield; R_f = 0.34 (silica gel, ethyl acetate/hexanes = 1/2). ^{1}H NMR (400 MHz, CD_3OD) δ = 2.86 (s, 1H), 2.05 – 1.78 (m, 6H), 1.77 – 1.67 (m, 2H), 1.67 – 1.55 (m, 2H), 1.55 – 1.45 (m, 1H), 1.45 – 1.35 (m, 2H), 1.35 – 1.20 (m, 2H); ^{13}C NMR (100 MHz, CD_3OD) δ = 88.3, 73.8, 71.6, 69.7, 42.7, 40.4, 39.4, 38.0, 31.7, 28.1, 22.7, 21.4 ppm; IR ν_{max} (film): 3283, 2932, 2862, 1703, 1560, 1449, 1336, 1276, 1144, 1057, 1034, 962, 932, 637 cm^{-1}; HRMS (ESI) m/z calcd for $C_{12}H_{18}O_2Na$ $[M+H]^+$: 217.1199; found: 217.1200.

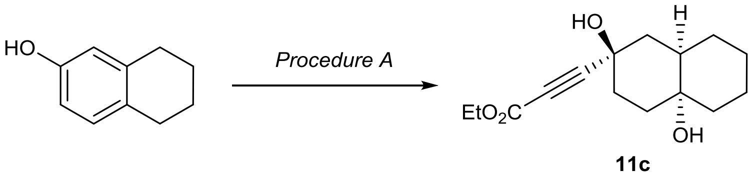

11c

Synthesis of 11c. Substrate **11c** was synthesized following the general procedure A, except that nucleophile generated in situ by reaction of ethyl propiolate (3.5 equiv) and LDA (3.0 equiv, 2.0 M in THF) at -60 °C for 30 min was used to replace the ethynylmagnesium chloride (3.0 equiv); R_f = 0.47

(silica gel, ethyl acetate/hexanes = 1/2). ^{1}H NMR (500 MHz, $CDCl_3$) δ = 4.23 (q, *J* = 7.1 Hz, 2H), 2.60 (br, 1H), 2.03 (br, 1H), 1.95 – 1.78 (m, 5H), 1.77 – 1.58 (m, 4H), 1.56 – 1.34(m, 4H), 1.31 (t, *J* = 7.1 Hz, 3H), 1.32 – 1.28 (m, 1H); ^{13}C NMR (100 MHz, $CDCl_3$) δ = 153.6, 89.8, 77.2, 70.7, 69.1, 62.2, 41.9, 40.9, 39.9, 36.3, 29.7, 26.97, 21.8, 20.9, 14.0 ppm; IR ν_{max} (film): 3385, 2932, 2862, 2234, 1713, 1238, 1034, 934, 754 cm^{-1}; HRMS (ESI) m/z calcd for $C_{15}H_{22}O_4Na$ $[M+Na]^+$: 298.1410; found: 298.1409.

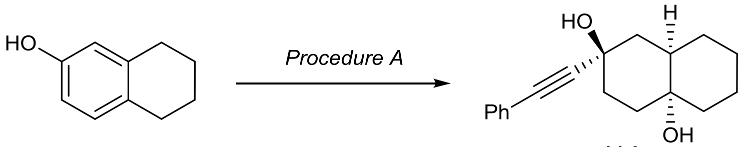

11d

Synthesis of 11d. Substrate **11d** was synthesized following the general procedure A, except that nucleophile generated in situ by reaction of phenylacetylene (3.5 equiv) and LDA (3.0 equiv, 2.0 M in THF) at -60 °C for 30 min was used to replace the ethynylmagnesium chloride (3.0 equiv); R_f = 0.32 (silica gel, ethyl acetate/hexanes = 1/4). ^{1}H NMR (400 MHz, $CDCl_3$) δ = 7.44 – 7.37 (m, 2H), 7.31 – 7.23 (m, 3H), 2.72 (br, 1H), 2.08 – 1.98 (m, 1H), 1.98 – 1.78 (m, 6H), 1.79 – 1.59 (m, 3H), 1.58 – 1.46 (m, 2H), 1.46 – 1.30 (m, 3H), 1.30 – 1.22 (m, 1H); ^{13}C NMR (100 MHz, $CDCl_3$) δ = 131.7, 128.3, 128.2, 122.5, 92.1, 84.9, 71.0, 69.7, 41. 9, 39.9, 38.6, 37.3, 31.1, 27.0, 21.7, 20.5 ppm; IR ν_{max} (film): 3341, 2932, 2862, 2365, 1487, 1443, 1333, 1275, 1148, 1088, 1034, 934, 756, 692 cm^{-1}; HRMS (ESI) m/z calcd for $C_{18}H_{22}O_2Na$ $[M+Na]^+$: 293.1512; found: 293.1510.

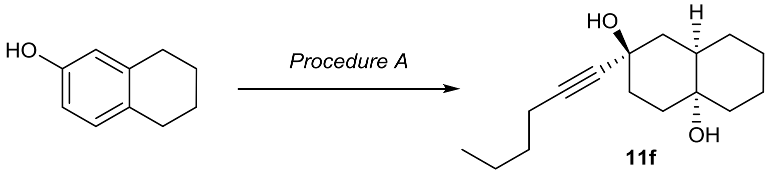

11f

Synthesis of 11f. Substrate **11f** was synthesized following the general procedure A, except that nucleophile generated in situ by reaction of 1-hexyne (3.5 equiv) and LDA (3.0 equiv, 2.0 M in THF) at -60 °C for 30 min was used to replace the ethynylmagnesium chloride (3.0 equiv); R_f = 0.28 (silica gel, ethyl acetate/hexanes = 1/4). ^{1}H NMR (400 MHz, $CDCl_3$) δ = 2.21 (t, *J* = 7.0 Hz, 2H), 2.02 (br, 1H), 1.94 – 1.80 (m, 5H), 1.75 – 1.60 (m, 4H), 1.58 – 1.44 (m, 4H), 1.42 – 1.32 (m, 4H), 1.32 – 1.28 (m, 1H), 1.27 – 1.19 (m, 1H), 0.91 (t, *J* = 7.2 Hz, 3H); ^{13}C NMR (100 MHz, CD_3OD) δ = 85.7, 84.6, 71.7, 70.1, 43.2, 40.5, 39.7, 38.5, 32.1, 31.7, 28.1, 23.0, 22.7, 21.4, 19.1, 14.0 ppm; IR ν_{max} (film): 3385, 2941, 2866, 2845, 1738, 1365, 1363, 1273, 1053, 1032, 1013, 764, 748 cm^{-1}; HRMS (ESI) m/z calcd for $C_{16}H_{26}O_2Na$ $[M+Na]^+$: 273.1825; found: 273.1824.

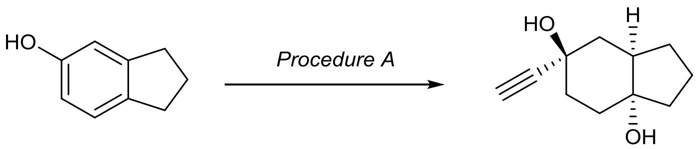

11h

Synthesis of 11h. Substrate **11h** was synthesized following the general procedure A, except that 5-indanol was used to replace 5,6,7,8-tetrahydro-2-naphthol; R_f = 0.25 (silica gel, ethyl acetate/hexanes = 1/2). ^{1}H NMR (500 MHz, CD_3OD) δ = 2.87 (s, 1H), 2.15 – 1.99 (m, 2H), 1.99 – 1.92 (m, 2H), 1.92 – 1.85 (m, 2H), 1.85 – 1.79 (m, 2H), 1.77 – 1.70 (m, 1H), 1.63 – 1.54 (m, 1H), 1.49 – 1.42 (m, 1H), 1.39 – 1.32 (m, 1H), 1.29 (t, *J* = 12.6 Hz, 1H); ^{13}C NMR (75 MHz, CD_3OD) δ = 87.6,

81.4, 74.1, 69.0, 46.0, 44.4, 37.9, 34.8, 33.6, 30.7, 21.5 ppm; IR ν_{max} (film): 3213, 2936, 1740, 1447, 1439, 1364, 1275, 1261, 1215, 1099, 1059, 1030, 750 cm^{-1}; HRMS (ESI) m/z calcd for $C_{11}H_{16}O_2Na$ $[M+Na]^+$: 203.1043; found: 203.1043.

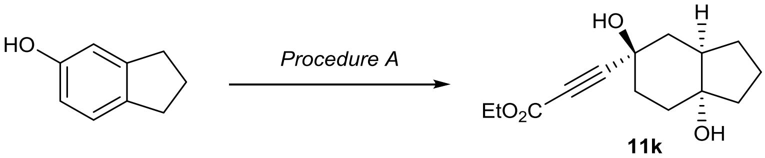

11k

Synthesis of 11k. Substrate **11k** was synthesized following the general procedure A, except that 5-indanol was used to replace 5,6,7,8-tetrahydro-2-naphthol and the nucleophile generated in situ by reaction of ethyl propiolate (3.5 equiv) and LDA (3.0 equiv, 2.0 M in THF) at -60 °C for 30 min was used to replace the ethynylmagnesium chloride (3.0 equiv); R_f = 0.26 (silica gel, ethyl acetate/hexanes = 1/2). ^{1}H NMR (400 MHz, $CDCl_3$) δ = 4.21 (q, J = 7.1 Hz, 2H), 3.49(br, 1H), 2.46 (br, 1H), 2.19 – 2.05 (m, 2H), 2.04 – 1.94 (m, 3H), 1.92 – 1.81 (m, 3H), 1.77 – 1.59 (m, 2H), 1.49 (t, J = 11.0 Hz, 1H), 1.36 (t, J = 12.1 Hz, 2H), 1.29 (t, J = 7.1 Hz, 3H); ^{13}C NMR (100 MHz, $CDCl_3$) δ = 153.8, 89.7, 80.6, 68.4, 62.3, 45.2, 42.5, 36.4, 33.9, 32.7, 29.5, 20.5, 13.9 ppm; IR ν_{max} (film): 3390, 3366, 2928, 2853, 2228, 1711, 1694, 1647, 1468, 1368, 1279, 1260, 1096, 1020, 962, 752 cm^{-1}; HRMS (ESI) m/z calcd for $C_{14}H_{20}O_4Na$ $[M+Na]^+$: 275.1254; found: 275.1258.

Procedure B:

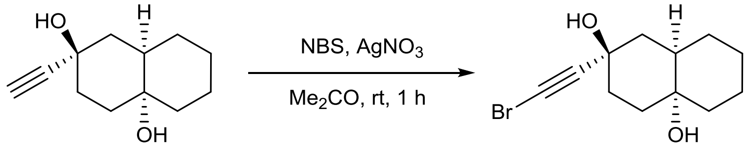

9 11a

Compound 11a. To a solution of alkyndiol **9** (180 mg, 0.93 mmol, 1.0 equiv) in acetone (9.0 mL) at ambient temperature was added sequentially with NBS (215 mg, 1.21 mmol, 1.3 equiv) and catalytic amount of $AgNO_3$ (8.0 mg, 0.047 mmol, 0.05 equiv). The mixture was stirred at ambient temperature for 1 h, and then poured into ice-water (10 mL). The reaction mixture was extracted with ethyl acetate (15 mL × 3), and the combined organic layers were washed with water (5.0 mL) followed by brine (5.0 mL), dried over Na_2SO_4, filtered, and concentrated *in vacuo*. Purification by flash column chromatography afforded the desired bromide **11a** (225 mg, 0.84 mmol) in 90% yield; R_f = 0.60 (silica gel, ethyl acetate/hexanes = 1/2). ^{1}H NMR (500 MHz, CD_3OD) δ = 2.35 – 2.10 (br, 1H), 2.05 – 1.93 (m, 1H), 1.92 – 1.77 (m, 4H), 1.77 – 1.45 (m, 6H), 1.44 – 1.34 (m, 2H), 1.33 – 1.20 (m, 2H); ^{13}C NMR (100 MHz, CD_3OD) δ = 84.8, 71.5, 71.0, 45.1, 42.7, 40.7, 38.9, 38.0, 32.2, 28.1, 22.8, 21.7 ppm; IR ν_{max} (film): 3385, 2936, 2862, 2502, 2247, 2224, 2079, 1452, 1119, 978, 820 cm^{-1}; HRMS (ESI) m/z calcd for $C_{12}H_{18}BrO_2$ $[M+H]^+$: 273.0485; found: 273.0486.

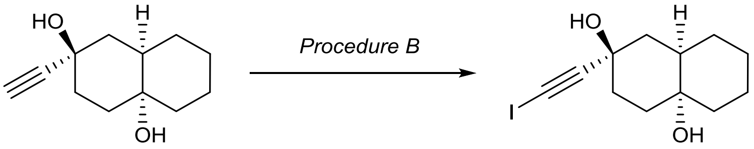

9 11b

Synthesis of 11b. Substrate **11b** was synthesized following the general procedure B, except that NIS was used to replace NBS; R_f = 0.42 (silica gel, ethyl acetate/hexanes = 1/2). ^{1}H NMR (500 MHz, CD_3OD) δ = 2.06 – 1.76 (m, 6H), 1.76 – 1.64 (m, 3H), 1.65 – 1.52 (m, 2H), 1.52 – 1.44 (m, 1H), 1.44

– 1.33 (m, 2H), 1.34 – 1.15 (m, 2H); ^{13}C NMR (100 MHz, CD_3OD) δ = 98.4, 71.6, 71.5, 42.9, 40.5, 39.5, 38.2, 31.7, 28.1, 22.7, 21.6, 5.1 ppm; IR ν_{max} (film): 3362, 2936, 2866, 2502, 2247, 2216, 2070, 1940, 1449, 1092, 1123, 978, 820 cm^{-1}; HRMS (ESI) m/z calcd for $C_{12}H_{17}IO_2Na$ [M+Na]$^+$: 343.0165; found: 343.0164.

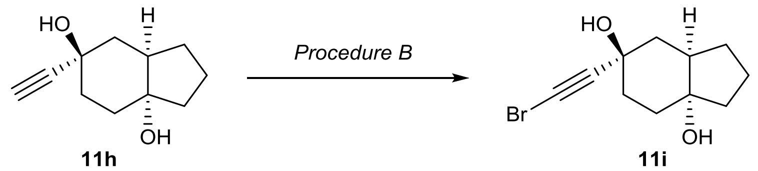

Synthesis of 11i. Substrate **11i** was synthesized following the general procedure B, except that **11h** was used to replace **9**; R_f = 0.38 (silica gel, ethyl acetate/hexanes = 1/2). ^{1}H NMR (500 MHz, CD_3OD) δ = 2.15 – 2.06 (m, 1H), 2.04 – 1.97 (m, 1H), 1.97 – 1.86 (m, 4H), 1.86 – 1.79 (m, 2H), 1.79 – 1.68 (m, 1H), 1.63 – 1.54 (m, 1H), 1.50 – 1.42 (m, 1H), 1.39 – 1.32 (m, 1H), 1.29 (t, J = 12.8 Hz, 1H); ^{13}C NMR (75 MHz, CD_3OD) δ = 84.0, 81.4, 70.2, 46.1, 45.5, 44.3, 37.9, 34.7, 33.6, 30.6, 21.5 ppm; IR ν_{max} (film): 3358, 2941, 2492, 2237, 2220, 2075, 1390, 1219, 1121, 976, 823 cm^{-1}; HRMS (ESI) m/z calcd for $C_{11}H_{15}O_2BrNa$ [M+Na]$^+$: 281.0148; found: 281.0148.

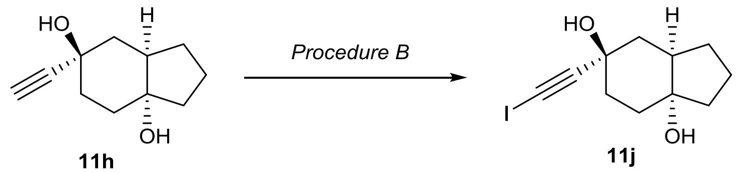

Synthesis of 11j. Substrate **11j** was synthesized following the general procedure B, except that **11h** was used to replace **9** and NIS was used to replace NBS; R_f = 0.40 (silica gel, ethyl acetate/hexanes = 1/2). ^{1}H NMR (400 MHz, CD_3OD) δ = 2.16 – 2.05 (m, 1H), 2.05 – 1.97 (m, 1H), 1.97 – 1.92 (m, 1H), 1.91 – 1.87 (m, 2H), 1.87 – 1.83 (m, 1H), 1.83 – 1.77 (m, 2H), 1.77 – 1.69 (m, 1H), 1.62 – 1.51 (m, 1H), 1.49 – 1.40 (m, 1H), 1.39 – 1.31 (m, 1H), 1.31 – 1.22 (m, 1H); ^{13}C NMR (100 MHz, CD_3OD) δ = 97.7, 81.4, 70.8, 46.0, 44.5, 38.1, 34.7, 33.7, 30.6, 21.5, 5.4 ppm; IR ν_{max} (film): 3451, 3007, 2967, 2945, 1736, 1713, 1423, 1364, 1277, 1260, 1229, 1215, 754 cm^{-1}; HRMS (ESI) m/z calcd for $C_{11}H_{15}O_2NaI$ [M+Na]$^+$: 329.0009; found: 329.0013.

Procedure C:

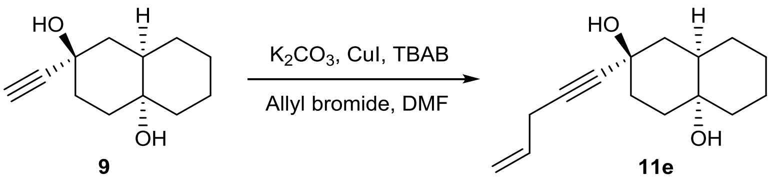

Compound 11e. To a stirred solution of alkyndiol **9** (175 mg, 0.90 mmol, 1.0 equiv) in dry DMF (9.0 mL) under argon atmosphere at ambient temperature was added sequentially with K_2CO_3 (186 mg, 1.35 mmol, 1.5 equiv), TBAB (43 mg, 0.14 mmol, 0.15 equiv) and catalytic amount of CuI (17 mg, 0.09 mmol, 0.1 equiv). After 15 min, allyl bromide (0.11 mL, 1.35 mmol, 1.5 equiv) was added. The mixture was stirred for 24 h at ambient temperature. Then it was poured into water (9.0 mL) and extracted with diethyl ether (20 mL × 4). The combined organic layers were dried over Na_2SO_4, filtered, and concentrated *in vacuo*. The residue was purified by flash column chromatography to afford **11e** (143 mg, 0.61 mmol) in 68% yield, R_f = 0.20 (silica gel, ethyl acetate/hexanes = 1/4),

together with 20% recovering starting material **9**. ^{1}H NMR (400 MHz, $CDCl_3$) δ = 5.81 (ddt, *J* = 17.0, 10.2, 5.2 Hz, 1H), 5.31 (dq, *J* = 17.0, 1.6 Hz, 1H), 5.12 (dq, *J* = 10.2, 1.6 Hz, 1H), 3.00 (dt, *J* = 5.2, 1.8 Hz, 2H), 2.15 (br, 1H), 1.94 – 1.81 (m, 5H), 1.80 – 1.69 (m, 2H), 1.67 – 1.48 (m, 4H), 1.44 – 1.21 (m, 5H); ^{13}C NMR (100 MHz, $CDCl_3$) δ = 132.4, 116.1, 85.7, 81.8, 71.0, 69.6, 42.0, 40.0, 38.5, 37.4, 31.1, 27.1, 23.0, 21.7, 20.5 ppm; IR ν_{max} (film): 3370, 2926, 2855, 1736, 1460, 1165, 1055, 1032, 993, 937, 741 cm^{-1}; HRMS (ESI) m/z calcd for $C_{15}H_{22}O_2Na$ $[M+Na]^+$: 257.1512; found: 257.1514.

Procedure D:

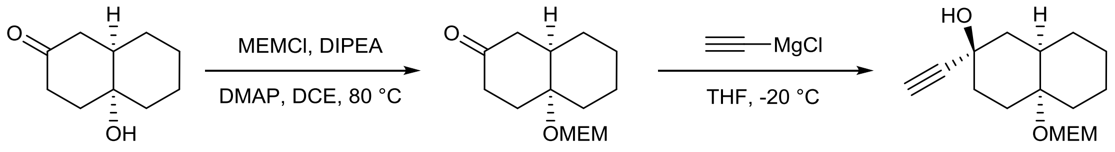

9-2 9-5 11g

Compound 9-5. Ketone **9-2** (262 mg, 1.56 mmol, 1.0 equiv) was dissolved in anhydrous DCE (10 mL), and DIPEA (0.54 mL, 3.12 mmol, 2.0 equiv), DMAP (20 mg, 0.16 mmol, 0.1 equiv) and MEMCl (0.27 mL, 2.34 mmol, 1.5 equiv) were added sequentially at ambient temperature. The mixture was degassed by argon, and heated to 80 °C. After stirred for 4 h, additional DIPEA (0.54 mL, 3.12 mmol, 2.0 equiv) and MEMCl (0.27 mL, 2.34 mmol, 1.5 equiv) were added into the reaction mixture. The reaction was stirred overnight at 80 °C, and after cooled to ambient temperature, it was quenched with saturated aqueous ammonium chloride (12 mL). The mixture was extracted with DCM (20 mL × 2), and the combined organic layers were dried over Na_2SO_4, filtered, and concentrated *in vacuo*. Purification by flash column chromatography afforded the desired product **9-5** (367 mg, 1.44 mmol) in 92% yield; R_f = 0.48 (silica gel, ethyl acetate/hexanes = 1/2). ^{1}H NMR (500 MHz, $CDCl_3$) δ = 4.97 (d, *J* = 7.6 Hz, 1H), 4.91 (d, *J* = 7.6 Hz, 1H), 3.83 – 3.74 (m, 2H), 3.55 (t, *J* = 4.7 Hz, 2H), 3.38 (s, 3H), 2.99 (dd, *J* = 14.1, 6.1 Hz, 1H), 2.70 (dt, *J* = 13.5, 7.5 Hz, 1H), 2.28 – 2.07 (m, 3H), 1.99 – 1.85 (m, 3H), 1.72 – 1.57 (m, 3H), 1.37 – 1.13 (m, 4H); ^{13}C NMR (125 MHz, $CDCl_3$) δ = 211.9, 89.2, 71.9, 67.5, 59.0, 43.9, 43.6, 37.0, 34.9, 30.4, 29.3, 25.3, 23.4 ppm; IR ν_{max} (film): 2928, 2859, 2359, 2342, 1713, 1275, 1260, 1036, 766, 748 cm^{-1}; HRMS (ESI) m/z calcd for $C_{14}H_{24}O_4Na$ $[M+Na]^+$: 279.1567; found: 279.1566.

Compound 11g. Compound **9-5** (367 mg, 1.44 mmol, 1.0 equiv) was dissolved in anhydrous THF (10 mL), and cooled to -20 °C. Ethynylmagnesium chloride (7.2 mL, 4.32 mmol, 3.0 equiv, 0.6 M in THF) was added, and the reaction mixture was warmed up to ambient temperature slowly during 4 h. The reaction was quenched with saturated aqueous ammonium chloride (15 mL), and extracted with ethyl acetate (20 mL × 3). The combined organic layers were dried over Na_2SO_4, filtered, and concentrated *in vacuo*. Purification by flash column chromatography afforded the desired product **11g** (356 mg, 1.26 mmol) as a single isomer in 88% yield; R_f = 0.45 (silica gel, ethyl acetate/hexanes = 1/2). ^{1}H NMR (500 MHz, $CDCl_3$) δ = 4.82 (q, *J* = 7.4 Hz, 2H), 3.80 – 3.69 (m, 2H), 3.55 (t, *J* = 4.7 Hz, 2H), 3.38 (s, 3H), 2.50 (s, 1H), 2.25 (br, 1H), 2.10 – 1.97 (m, 1H), 1.97 – 1.82 (m, 4H), 1.82 – 1.71 (m, 3H), 1.69 – 1.54 (m, 2H), 1.53 – 1.42 (m, 2H), 1.42 – 1.20 (m, 3H); ^{13}C NMR (125 MHz, $CDCl_3$) δ = 89.2, 86.3, 76.6, 72.7, 71.9, 69.0, 67.2, 58.9, 41.5, 38.0, 37.3, 36.7, 29.6, 27.2, 21.7, 20.7 ppm; IR ν_{max} (film): 3416, 3306, 2932, 2866, 1464, 1449, 1364, 1275, 1134, 1090, 1088, 1032, 750 cm^{-1}; HRMS (ESI) m/z calcd for $C_{16}H_{26}O_4Na$ $[M+Na]^+$: 305.1723; found: 305.1724.

Procedure E:

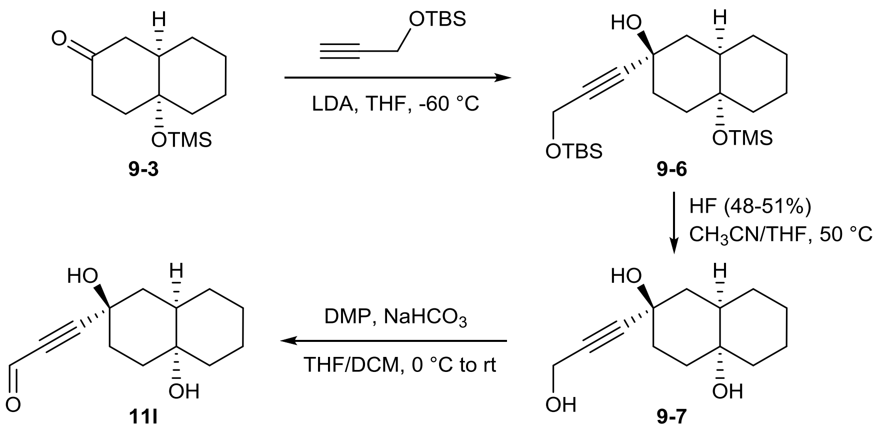

Compound 9-6. To a solution of alkyne (1.05 g, 6.20 mmol, 4.0 equiv) in anhydrous THF (9.0 mL) at -60 °C was added LDA (2.3 mL, 4.65 mmol, 3.0 equiv, 2.0 M in THF) dropwise. The mixture was stirred at -60 °C for 30 min, and then a solution of ketone **9-3** (372 mg, 1.55 mmol, 1.0 equiv) in THF (4.0 mL) was added dropwise. After 20 min, the reaction was warmed up to ambient temperature slowly during 4 h, and quenched with saturated aqueous ammonium chloride (15 mL). The mixture was extracted with ethyl acetate (20 mL × 3), and the combined organic layers were dried over Na_2SO_4, filtered, and concentrated *in vacuo*. The residue was purified by flash column chromatography to afford **9-6** (463 mg, 1.13 mmol) in 73% yield; R_f = 0.60 (silica gel, ethyl acetate/hexanes = 1/8). ^1H NMR (500 MHz, $CDCl_3$) δ = 4.36 (s, 2H), 2.11 (br, 1H), 2.02 – 1.81 (m, 4H), 1.82 – 1.72 (m, 2H), 1.72 – 1.57 (m, 4H), 1.48 – 1.11 (m, 5H), 0.91 (s, 9H), 0.12 (s, 6H), 0.11 (s, 9H); ^{13}C NMR (125 MHz, $CDCl_3$) δ = 88.0, 83.4, 74.6, 69.5, 51.7, 41.9, 40.3, 38.4, 37.3, 31.4, 27.2, 25.8, 22.0, 20.7, 18.3, 2.7, -5.1 ppm; IR v_{max} (film): 3642, 2934, 2859, 1464, 1369, 1250, 1169, 1101, 1055, 955, 868, 837, 777 cm^{-1}; HRMS (ESI) m/z calcd for $C_{22}H_{42}O_3NaSi_2$ $[M+Na]^+$: 433.2565; found: 433.2564.

Compound 9-7. To a stirred solution of **9-6** (463 mg, 1.13 mmol, 1.0 equiv) in CH_3CN/THF (6.0 mL/6.0 mL) in plastic tube at ambient temperature was added HF (1.5 mL, 48-51% solution in water) dropwise. The reaction was stirred for 3 h at 50 °C, and then after cooled to ambient temperature, it was quenched by careful addition of saturated aqueous sodium bicarbonate (20 mL). The mixture was extracted with ethyl acetate (25 mL × 4), and the combined organic layers were dried over Na_2SO_4, filtered, and concentrated *in vacuo*. Purification by flash column chromatography afforded triol **9-7** (197 mg, 0.88 mmol) in 78% yield; R_f = 0.22 (silica gel, ethyl acetate/hexanes = 3/1). ^1H NMR (400 MHz, CD_3OD) δ = 4.23 (s, 2H), 2.03 – 1.78 (m, 5H), 1.78 – 1.67 (m, 2H), 1.67 – 1.58 (m, 2H), 1.58 – 1.45 (m, 2H), 1.45 – 1.34 (m, 2H), 1.34 – 1.20 (m, 2H); ^{13}C NMR (100 MHz, CD_3OD) δ = 89.2, 83.3, 71.7, 69.9, 50.8, 42.9, 42.7, 40.5, 38.1, 31.8, 28.1, 22.7, 21.7 ppm; IR v_{max} (film): 3684, 2965, 2928, 2367, 1564, 1443, 1368, 1273, 1225, 1219, 766, 752cm^{-1}; HRMS (ESI) m/z calcd for $C_{13}H_{20}O_3Na$ $[M+Na]^+$: 247.1305; found: 247.1305.

Compound 11l. Triol **9-7** (197 mg, 0.88 mmol, 1.0 equiv) was dissolved in THF/DCM (8.0 mL/8.0 mL), and cooled to 0 °C. $NaHCO_3$ (222 mg, 2.64 mmol, 3.0 equiv) and DMP (746 mg, 1.76 mmol, 2.0 equiv) were added sequentially, and the reaction was warmed up to ambient temperature. After 2 h,

the reaction was quenched with saturated aqueous sodium thiosulfate (10 mL), and extracted with DCM (15 mL × 3), and the combined organic layers were dried over Na_2SO_4, filtered, and concentrated *in vacuo*. The residue was purified by flash column chromatography to afford aldehyde **11l** (169 mg, 0.76 mmol) in 87% yield; R_f = 0.50 (silica gel, ethyl acetate/hexanes = 1/1). ^{1}H NMR (400 MHz, $CDCl_3$) δ = 9.23 (s, 1H), 2.98 (br, 1H), 2.10 – 2.02 (m, 1H), 1.96 – 1.83 (m, 5H), 1.81 –1.71 (m, 3H), 1.69 – 1.59 (m, 2H), 1.56 – 1.47 (m, 1H), 1.45 – 1.33 (m,3H), 1.33 – 1.25 (m, 1H); ^{13}C NMR (125 MHz, $CDCl_3$) δ = 176.7, 98. 9, 83.8, 70.7, 69.2, 40.9, 40.0, 36.5, 36.2, 31.8, 27.2, 21.8, 21.0 ppm; IR ν_{max} (film): 3393, 2924, 2862, 1649, 1450, 1341, 1273, 1159, 1065, 999, 764, 748 cm^{-1}; HRMS (ESI) m/z calcd for $C_{13}H_{18}O_3Na$ [M+Na]$^+$: 245.1148; found: 245.1150.

General procedure F for the gold-catalyzed sequential reactions:

A mixture of $Ph_3PAuNTf_2$ (2.5 mg, 0.005 mmol, 0.025 equiv) and $AgNTf_2$ (1.9 mg, 0.005 mmol, 0.025 equiv) in DCM (1.0 mL) was stirred for 0.5 h to generate the active gold catalyst in situ. Then the mixture was added into a stirred solution of the substrate (0.20 mmol, 1.0 equiv) in DCM (4.0 mL), and the reaction was stirred at ambient temperature for 2 h. Direct purification by flash column chromatography afforded the desired product.

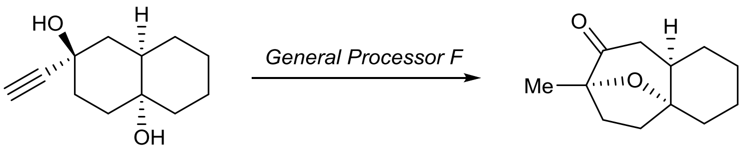

9 10

Synthesis of 10. Product **10** was obtained in 83% yield from substrate **9** following the general procedure F; R_f = 0.70 (silica gel, ethyl acetate/hexanes = 1/4). ^{1}H NMR (400 MHz, $CDCl_3$) δ = 2.39 – 2.27 (m, 1H), 2.27 – 2.15 (m, 1H), 2.07 – 1.95 (m, 2H), 1.90 – 1.76 (m, 4H), 1.73 – 1.55 (m, 3H), 1.60 (d, J = 1.6 Hz, 2H), 1.35 (s, 3H), 1.28 – 1.20 (m, 2H); ^{13}C NMR (100 MHz, $CDCl_3$) δ = 207.8, 85.7, 83.8, 46.4, 40.3, 37.0, 35.7, 30.8, 29.9, 25.1, 24.2, 18.8 ppm; IR ν_{max} (film): 2924, 2853, 1722, 1449, 1377, 1277, 1261, 1113, 764, 748 cm^{-1}; HRMS (ESI) m/z calcd for $C_{12}H_{18}O_2Na$ [M+Na]$^+$: 217.1199; found: 217.1200.

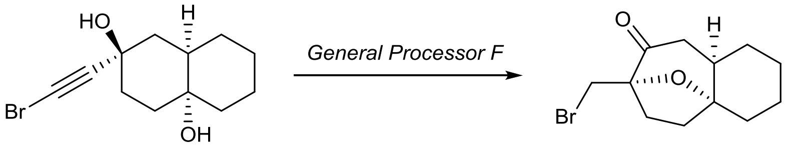

11a 12a

Synthesis of 12a. Product **12a** was obtained in 75% yield from substrate **11a** following the general procedure F; R_f = 0.63 (silica gel, ethyl acetate/hexanes = 1/4). ^{1}H NMR (500 MHz, $CDCl_3$) δ = 3.68 (d, J = 11.0 Hz, 1H), 3.64 (d, J = 11.0 Hz, 1H), 2.43 – 2.33(m, 1H), 2.31 – 2.17 (m, 2H), 2.14 – 1.98(m, 2H), 1.91 – 1.75(m, 3H), 1.77 – 1.60 (m, 4H), 1.31 – 1.10 (m, 3H); ^{13}C NMR (125 MHz, $CDCl_3$) δ = 205.2, 85.8, 84.9, 46.3, 40.4, 36.7, 32.8, 32.6, 30.7, 29.7, 25.0, 24.1 ppm; IR ν_{max} (film): 2936, 2853, 2357, 2338, 1722, 1649, 1456, 1100, 1096, 1030, 1003, 964, 945, 887 cm^{-1}; HRMS (ESI) m/z calcd for $C_{12}H_{17}O_2NaBr$ [M+Na]$^+$: 295.0304; found: 295.0309.

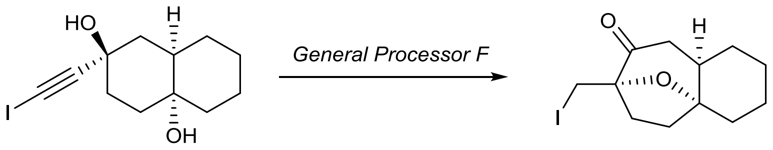

11b **12b**

Synthesis of 12b. Product **12b** was obtained in 80% yield from substrate **11b** following the general procedure F; R_f = 0.50 (silica gel, ethyl acetate/hexanes = 1/8). ^{1}H NMR (400 MHz, $CDCl_3$) δ = 3.52 (dd, *J* = 11.0, 2.0 Hz, 1H), 3.43 (dd, *J* = 11.0, 1.9 Hz, 1H), 2.41 – 2.33 (m, 1H), 2.33 – 2.23 (m, 1H), 2.17 – 1.98 (m, 3H), 1.93 – 1.77 (m, 3H), 1.73 – 1.61 (m, 4H), 1.30 – 1.10 (m, 3H); ^{13}C NMR (100 MHz, $CDCl_3$) δ = 204.5, 85.2, 85.0, 46.6, 40.4, 36.7, 34.6, 30.7, 30.1, 25.0, 24.1, 6.8 ppm; IR ν_{max} (film): 2934, 2859, 1726, 1452, 1375, 1246, 1099, 1065, 1001, 966, 945, 887, 868, 766, 748 cm^{-1}; HRMS (ESI) m/z calcd for $C_{12}H_{17}O_2INa$ [M+Na]$^+$: 343.0165; found: 343.0165.

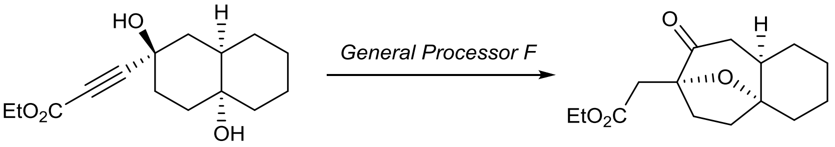

11c **12c**

Synthesis of 12c. Product **12c** was obtained in 76% yield from substrate **11c** following the general procedure F, except that the reaction was carried out in tube sealing at 70 °C for 2 h; R_f = 0.42 (silica gel, ethyl acetate/hexanes = 1/8). ^{1}H NMR (400 MHz, $CDCl_3$) δ = 4.12 (qd, *J* = 7.2, 1.2 Hz, 2H), 2.82 (d, *J* = 16.0 Hz, 1H), 2.65 (d, *J* = 16.0 Hz, 1H), 2.40 – 2.28 (m, 1H), 2.27 – 2.16 (m, 1H), 2.16 – 2.04 (m, 3H), 2.04 – 1.96 (m, 1H), 1.85 – 1.74 (m, 2H), 1.71 – 1.57 (m, 4H), 1.30 – 1.11 (m, 3H), 1.24 (t, *J* = 7.2 Hz, 3H); ^{13}C NMR (100 MHz, $CDCl_3$) δ = 205.9, 170.2, 85.5, 83.9, 60.4, 46.4, 40.0, 38.0, 36.7, 33.9, 30.8, 29.4, 25.0, 24.2, 14.1 ppm; IR ν_{max} (film): 2934, 2859, 1726, 1452, 1373, 1335, 1300, 1275, 1260, 1186, 1130, 1040, 766, 752 cm^{-1}; HRMS (ESI) m/z calcd for $C_{15}H_{23}O_4Na$ [M+Na]$^+$: 289.1410; found: 289.1406.

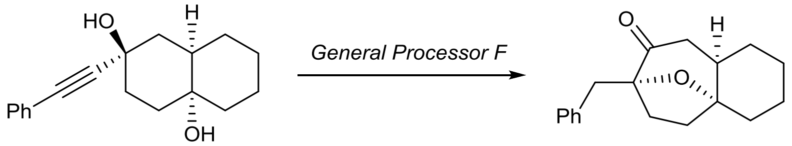

11d **12d**

Synthesis of 12d. Product **12d** was obtained in 68% yield from substrate **11d** following the general procedure F; R_f = 0.58 (silica gel, ethyl acetate/hexanes = 1/8). ^{1}H NMR (400 MHz, $CDCl_3$) δ = 7.35 – 7.12 (m, 5H), 3.11 (d, *J* = 14.6 Hz, 1H), 3.05 (d, *J* = 14.6 Hz, 1H), 2.43 – 2.29 (m, 1H), 2.20 – 2.08 (m, 1H), 2.09 – 1.95(m, 2H), 1.88 – 1.71 (m, 3H), 1.70 – 1.60 (m, 4H), 1.53 – 1.38 (m, 1H), 1.27 – 1.09 (m, 3H); ^{13}C NMR (100 MHz, $CDCl_3$) δ = 207.6, 137.1, 130.9, 127.7, 126.1, 87.6, 83.5, 46.4, 40.5, 36.9, 36.8, 31.8, 30.7, 29.5, 25.1, 24.1 ppm; IR ν_{max} (film): 2928, 1721, 1493, 1452, 1277, 1260, 1126, 1092, 1030, 943, 889, 750, 698 cm^{-1}; HRMS (ESI) m/z calcd for $C_{18}H_{22}O_2Na$ [M+Na]$^+$: 293.1512; found: 293.1509.

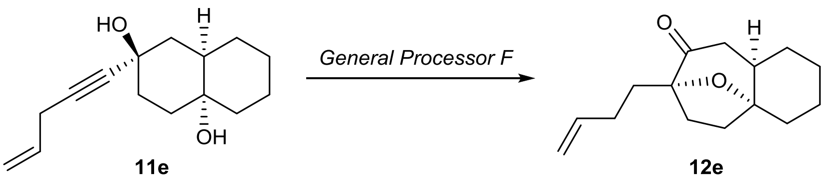

11e **12e**

Synthesis of 12e. Product **12e** was obtained in 52% yield from substrate **11e** following the general procedure F; R_f = 0.75 (silica gel, ethyl acetate/hexanes = 1/4). ^{1}H NMR (400 MHz, $CDCl_3$) δ = 5.84 (ddt, *J* = 16.8, 10.2, 6.5 Hz, 1H), 5.03 (dd, *J* = 17.1, 1.8 Hz, 1H), 4.93 (dd, *J* = 10.2, 1.9 Hz, 1H), 2.38 – 2.26 (m, 1H), 2.25 – 2.08 (m, 3H), 2.05 – 1.96 (m, 2H), 1.96 – 1.84 (m, 2H), 1.84 – 1.71 (m, 4H), 1.70 – 1.61 (m, 3H), 1.29 – 1.18 (m, 4H); ^{13}C NMR (100 MHz, $CDCl_3$) δ = 207.8, 138.8, 114.2, 87.6, 83.6, 46.2, 40.6, 37.0, 33.7, 31.8, 30.8, 29.5, 28.2, 25.1, 24.2 ppm; IR v_{max} (film): 2926, 2854, 2361, 2343, 1719, 1560, 1450, 1260, 1096, 1070, 1020, 903, 868, 795 cm^{-1}; HRMS (ESI) m/z calcd for $C_{15}H_{22}O_2Na$ $[M+Na]^+$: 257.1512; found: 257.1512.

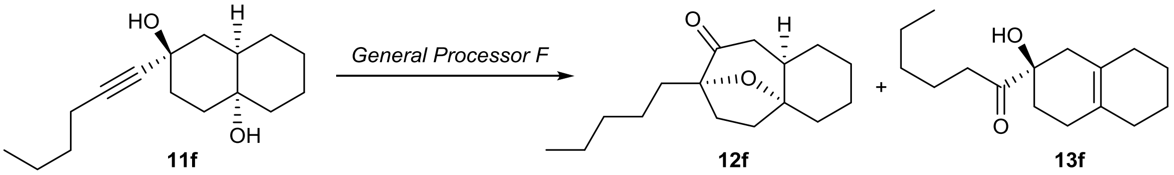

11f **12f** **13f**

Synthesis of 12f and 13f. Product **12f** and **13f** were obtained in 58% and 21% yield respectively from substrate **11f** following the general procedure F, except that the reaction was carried out in tube sealing at 70 °C for 2 h.

12f: R_f = 0.68 (silica gel, ethyl acetate/hexanes = 1/8); ^{1}H NMR (400 MHz, $CDCl_3$) δ = 2.39 – 2.25 (m, 1H), 2.25 – 2.12 (m, 1H), 2.08 – 1.86 (m, 3H), 1.84 – 1.72 (m, 3H), 1.72 – 1.55 (m, 6H), 1.47 – 1.07 (m, 9H), 0.87 (t, *J* = 6.6 Hz, 3H); ^{13}C NMR (100 MHz, $CDCl_3$) δ = 208.1, 88.0, 83.5, 46.3, 40.6, 37.0, 33.5, 32.6, 32.5, 30.8, 29.5, 25.1, 24.2, 23.6, 22.6, 14.1 ppm; IR v_{max} (film): 2932, 2862, 2365, 1722, 1452, 1277, 1261, 1126, 1099, 1070, 754 cm^{-1}; HRMS (ESI) m/z calcd for $C_{16}H_{26}O_2Na$ $[M+Na]^+$: 273.1825; found: 273.1827.

13f: R_f = 0.40 (silica gel, ethyl acetate/hexanes = 1/8). ^{1}H NMR (400 MHz, $CDCl_3$) δ = 3.89 (s, 1H), 2.60 (s, 2H), 2.43 (t, *J* = 7.4 Hz, 2H), 2.16 – 1.98 (m, 2H), 1.97 – 1.68 (m, 7H), 1.60 – 1.50 (m, 6H), 1.37 – 1.23 (m, 3H), 0.90 (t, *J* = 7.3 Hz, 3H); ^{13}C NMR (100 MHz, $CDCl_3$) δ = 213.6, 126.9, 125.2, 70.3, 50.5, 44.4, 43.1, 33.8, 30.2, 29.7, 28.0, 25.6, 23.0, 22.9, 22.2, 13.8 ppm; IR v_{max} (film): 3451, 2932, 2873, 2835, 2361, 2337, 1703, 1404, 1132, 1105, 1037, 966 cm^{-1}; HRMS (ESI) m/z calcd for $C_{16}H_{26}O_2Na$ $[M+Na]^+$: 273.1825; found: 273.1825.

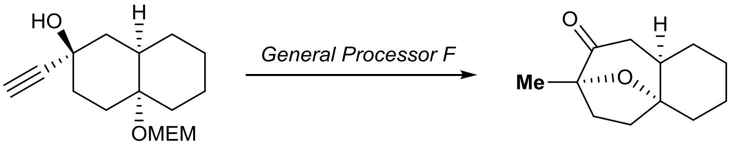

11g **12g**
(that is also **10**)

Synthesis of 12g. Product **12g** (that is also **10**) was obtained in 65% yield from substrate **11g** following the general procedure F. ^{1}H NMR (400 MHz, $CDCl_3$) δ = 2.39 – 2.27 (m, 1H), 2.27 – 2.15 (m, 1H), 2.07 – 1.95 (m, 2H), 1.90 – 1.76 (m, 4H), 1.73 – 1.55 (m, 3H), 1.60 (d, *J* = 1.6 Hz, 2H), 1.35 (s, 3H), 1.28 – 1.20 (m, 2H); ^{13}C NMR (100 MHz, $CDCl_3$) δ = 207.8, 85.7, 83.8, 46.4, 40.3, 37.0, 35.7, 30.8, 29.9, 25.1, 24.2, 18.8 ppm; IR v_{max} (film): 2924, 2853, 1722, 1449, 1377, 1277, 1261, 1113, 764, 748 cm^{-1}; HRMS (ESI) m/z calcd for $C_{12}H_{18}O_2Na$ $[M+Na]^+$: 217.1199; found: 217.1200.

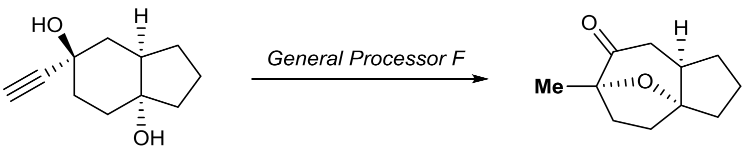

11h **12h**

Synthesis of 12h. Product **12h** was obtained in 85% yield from substrate **11h** following the general procedure F; R_f = 0.83 (silica gel, ethyl acetate/hexanes = 1/2). ^{1}H NMR (400 MHz, $CDCl_3$) δ = 2.49 (dd, *J* = 14.4, 4.0 Hz, 1H), 2.35 – 2.10 (m, 3H), 1.99 – 1.90 (m, 1H), 1.89 – 1.69 (m, 7H), 1.36 (s, 3H), 1.32 – 1.18 (m, 1H); ^{13}C NMR (100 MHz, $CDCl_3$) δ = 208.0, 88.8, 85.7, 48.9, 39.7, 35.4, 32.7, 30.9, 26.1, 20.8, 18.9 ppm; IR v_{max} (film): 2949, 2874, 1719, 1375, 1315, 1275, 1260, 1103, 1059, 950, 802, 750 cm^{-1}; HRMS (ESI) m/z calcd for $C_{11}H_{16}O_2Na$ [M+Na]$^+$: 203.1043; found: 203.1041.

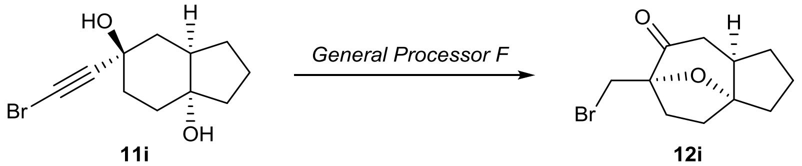

11i **12i**

Synthesis of 12i. Product **12i** was obtained in 78% yield from substrate **11i** following the general procedure F; R_f = 0.40 (silica gel, ethyl acetate/hexanes = 1/8). ^{1}H NMR (400 MHz, $CDCl_3$) δ = 3.72 (d, *J* = 11.3 Hz, 1H), 3.64 (d, *J* = 11.3 Hz, 1H), 2.55 (dd, *J* = 14.8, 4.4 Hz, 1H), 2.39 – 2.19 (m, 4H), 2.09 – 1.95 (m, 1H), 1.91 – 1.72 (m, 6H), 1.35 – 1.28 (m, 1H); ^{13}C NMR (100 MHz, $CDCl_3$) δ = 205.5, 89.6, 85.9, 48.7, 39.9, 32.7, 32.6, 32.5, 30.7, 26.0, 20.8 ppm; IR v_{max} (film): 2959, 2880, 1721, 1466, 1425, 1416, 1321, 1253, 1121, 1094, 1055, 1018 cm^{-1}; HRMS (ESI) m/z calcd for $C_{11}H_{15}O_2NaBr$ [M+Na]$^+$: 281.0148; found: 281.0145.

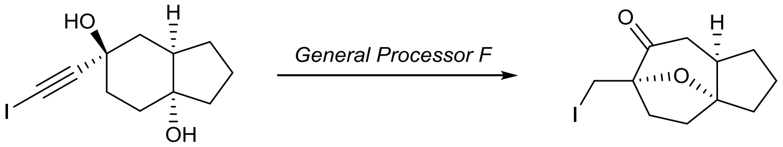

11j **12j**

Synthesis of 12j. Product **12j** was obtained in 81% yield from substrate **11j** following the general procedure F; R_f = 0.42 (silica gel, ethyl acetate/hexanes = 1/8). ^{1}H NMR (400 MHz, $CDCl_3$) δ = 3.55 (d, *J* = 11.0 Hz, 1H), 3.43 (d, *J* = 11.0 Hz, 1H), 2.54 (dd, *J* = 14.4, 4.4 Hz, 1H), 2.40 – 2.27 (m, 2H), 2.27 – 2.19 (m, 2H), 2.16 – 2.08 (m, 1H), 2.04 – 1.95 (m, 1H), 1.92 – 1.82 (m, 4H), 1.76 – 1.71 (m, 2H), 1.34 – 1.24 (m, 1H); ^{13}C NMR (100 MHz, $CDCl_3$) δ = 204.8, 89.6, 85.3, 48.9, 39.8, 34.3, 32.5, 31.2, 26.0, 20.8, 6.4 ppm; IR v_{max} (film): 2955, 2874, 2361, 1717, 1464, 1319, 1273, 1260, 1103, 1053, 748 cm^{-1}; HRMS (ESI) m/z calcd for $C_{11}H_{15}O_2NaI$ [M+Na]$^+$: 329.0009; found: 329.0009.

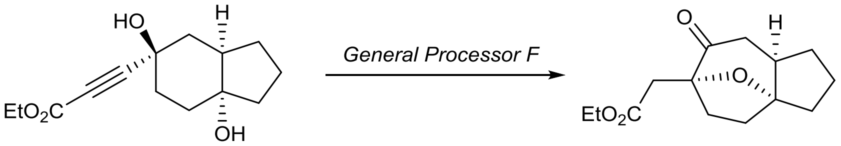

11k **12k**

Synthesis of 12k. Product **12k** was obtained in 81% yield from substrate **11k** following the general procedure F, except that the reaction was carried out in tube sealing at 70 °C for 2 h; R_f = 0.67 (silica gel, ethyl acetate/hexanes = 1/2). ^{1}H NMR (400 MHz, $CDCl_3$) δ = 4.20 – 4.02 (m, 2H), 2.83 (d, *J* = 16.1 Hz, 1H), 2.69 (d, *J* = 16.1 Hz, 1H), 2.52 (dd, *J* = 13.6, 4.0 Hz, 1H), 2.37 – 2.25 (m, 2H), 2.24 – 2.03 (m, 3H), 2.01 – 1.90 (m, 1H), 1.87 – 1.65 (m, 5H), 1.36 – 1.27 (m, 1H), 1.24 (t, *J* = 7.1 Hz, 3H); ^{13}C NMR (100 MHz, $CDCl_3$) δ = 206.3, 170.2, 88.8, 85.7, 60.4, 48.8, 39.5, 38.1, 33.7, 32.5, 30.3, 26.1, 20.8, 14.1 ppm; IR v_{max} (film): 2959, 2879, 1736, 1722, 1373, 1321, 1273, 1260, 1194, 1096, 1032, 945, 764, 748 cm^{-1}; HRMS (ESI) m/z calcd for $C_{14}H_{20}O_4Na$ [M+Na]$^+$: 275.1254; found: 275.1253.

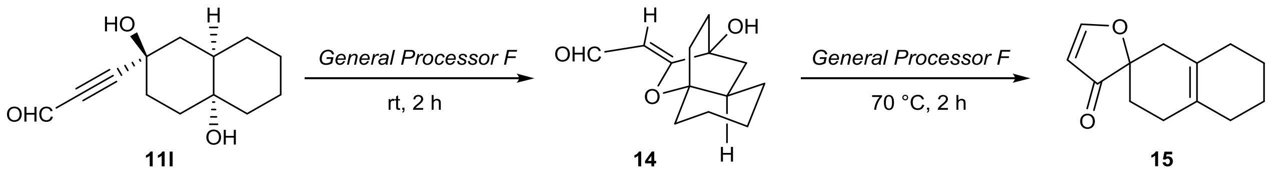

Compound 14. Product **14** was obtained in 85% yield from substrate **11l** following the general procedure F; R_f = 0.45 (silica gel, ethyl acetate/hexanes = 1/1). ^{1}H NMR (400 MHz, $CDCl_3$) δ = 9.90 (d, *J* = 8.8 Hz, 1H), 5.49 (d, *J* = 8.8 Hz, 1H), 3.41 (s, 1H), 2.37 (ddd, *J* = 14.0, 11.6, 4.4 Hz, 1H), 2.04 – 1.94 (m, 2H), 1.90 – 1.82 (m, 4H), 1.78 – 1.64 (m, 4H), 1.49 – 1.39 (m, 1H), 1.31 – 1.16 (m, 3H); ^{13}C NMR (100 MHz, $CDCl_3$) δ = 189.9, 182.9, 99.2, 80.5, 69.3, 40.1, 39.0, 35.2, 32.0, 31.7, 25.3, 25.2, 22.9 ppm; IR v_{max} (film): 3426, 2930, 2866, 1688, 1557, 1450, 1366, 1206, 1144, 999, 750 cm^{-1}; HRMS (ESI) m/z calcd for $C_{13}H_{18}O_3Na$ [M+Na]$^+$: 245.1148; found: 245.1150.

Compound 15. Product **15** was obtained in 91% yield from substrate **14** or in 82% yield directly from substrate **11l** following the general procedure F except that the reaction was carried out in tube sealing at 70 °C for 2 h; R_f = 0.58 (silica gel, ethyl acetate/hexanes = 1/4). ^{1}H NMR (400 MHz, $CDCl_3$) δ = 8.19 (d, *J* = 2.4 Hz, 1H), 5.66 (d, *J* = 2.4 Hz, 1H), 2.40 (d, *J* = 17.6 Hz, 1H), 2.27 – 2.12 (m, 1H), 2.04 – 1.77 (m, 7H), 1.76 – 1.68 (m, 2H), 1.65 – 1.60 (m, 1H), 1.60 – 1.48 (m, 2H); ^{13}C NMR (100 MHz, $CDCl_3$) δ = 207.2, 176.4, 127.3, 124.0, 105.5, 88.8, 36.5, 29.9, 29.7, 27.9, 26.6, 22.9, 22.8 ppm; IR v_{max} (film): 2928, 2887, 2835, 1699, 1562, 1439, 1275, 1258, 1180, 1157, 1049, 1022, 804, 791, 750 cm^{-1}; HRMS (ESI) m/z calcd for $C_{13}H_{16}O_2Na$ [M+Na]$^+$: 227.1043; found: 227.1041.

General procedure G:

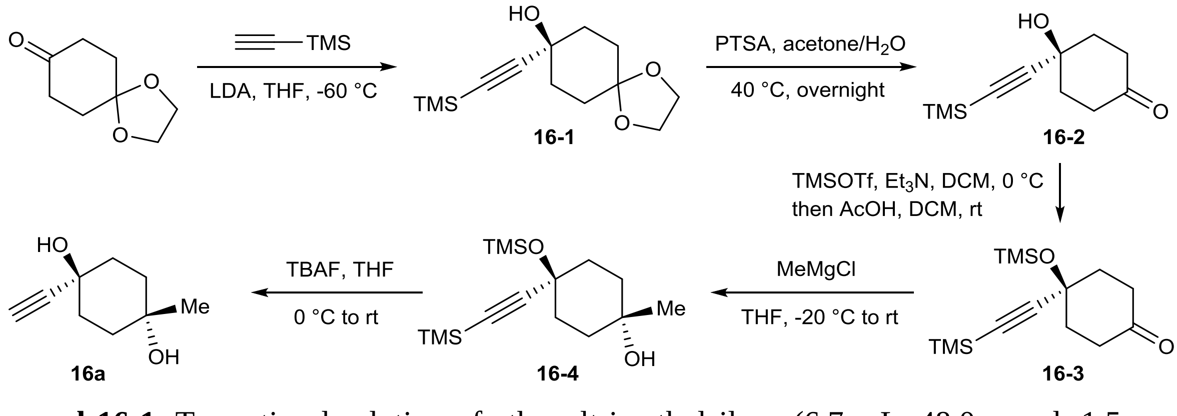

Compound 16-1. To a stirred solution of ethynyltrimethylsilane (6.7 mL, 48.0 mmol, 1.5 equiv) in anhydrous THF (300 mL) at -60 °C was added LDA (22.4 mL, 44.8 mmol, 1.4 equiv, 2.0 M in THF). The mixture was stirred at -60 °C for 30 min, and then a solution of 1,4-dioxaspiro[4.5]decan-8-one (5.00 g, 32.0 mmol, 1.0 equiv) in THF (20 mL) was added dropwise. After 20 min, the reaction was warmed up to ambient temperature slowly during 4 h, and quenched with saturated aqueous ammonium chloride (120 mL). The mixture was extracted with ethyl acetate (150 mL × 3), and the combined organic layers were dried over Na_2SO_4, filtered, and concentrated *in vacuo*. The residue was purified by flash column chromatography to afford **16-1** (7.72 g, 30.4 mmol) in 95% yield; R_f = 0.45 (silica gel, ethyl acetate/hexanes = 1/4). ^{1}H NMR (500 MHz, $CDCl_3$) δ = 3.91 (s, 4H), 2.39 (br, 1H), 1.95 – 1.88 (m, 2H), 1.88 – 1.81 (m, 2H), 1.78 – 1.69 (m, 4H), 0.12 (s, 9H); ^{13}C NMR (125 MHz, $CDCl_3$) δ = 108.8, 108.0, 88.2, 67.4, 64.2, 64.1, 37.0, 31.4, -0.1 ppm; IR v_{max} (film): 3443, 2959, 2887,

1364, 1250, 1107, 1034, 980, 935, 843, 758 cm^{-1}; HRMS (ESI) m/z calcd for $C_{13}H_{22}O_3NaSi$ $[M+Na]^+$: 277.1230; found: 277.1232.

Compound 16-2. Compound **16-1** (7.00 g, 27.6 mmol, 1.0 equiv) was dissolved in acetone/H_2O (180 mL/60 mL), and PTSA (2.37 g, 13.8 mmol, 0.5 equiv) was added into the solution at ambient temperature. The mixture was warmed up to 40 °C, and stirred overnight. After cooled to ambient temperature, the reaction was quenched by careful addition of saturated aqueous sodium bicarbonate (40 mL). The mixture was extracted with ethyl acetate (120 mL × 4), and the combined organic layers were dried over Na_2SO_4, filtered, and concentrated *in vacuo*. Purification by flash column chromatography afforded ketone **16-2** (5.45 g, 25.9 mmol) in 94% yield; R_f = 0.40 (silica gel, ethyl acetate/hexanes = 1/4). ^{1}H NMR (500 MHz, $CDCl_3$) δ = 3.09 (br, 1H), 2.58 – 2.32 (m, 4H), 2.22 – 1.91 (m, 4H), 0.13 (s, 9H); ^{13}C NMR (125 MHz, $CDCl_3$) δ = 210.5, 107.3, 89.3, 66.4, 38.7, 37.4, -0.2 ppm; IR v_{max} (film): 3286, 2953, 2928, 2158, 1738, 1722, 1694, 1420, 1364, 1256, 1229, 1126, 1092, 962, 841, 762 cm^{-1}; HRMS (ESI) m/z calcd for $C_{11}H_{18}O_2NaSi$ $[M+Na]^+$: 233.0968; found: 233.0969.

Compound 16-3. To a solution of ketone **16-2** (5.45 g, 25.9 mmol, 1.0 equiv) in anhydrous DCM (200 mL) at 0 °C was added dried triethylamine (21.8 mL, 155.4 mmol, 6.0 equiv) followed by the addition of TMSOTf (14.1 mL, 77.7 mmol, 3.0 equiv). The reaction mixture was warmed up to ambient temperature slowly during 3 h, and then quenched with water (100 mL). The organic layer was separated, and the aqueous phase was extracted with DCM (150 mL × 2). The combined organic extracts were dried over Na_2SO_4 and filtered, and the solvents were removed *in vacuo*. The residue was redissolved in DCM (100 mL), and acetic acid (8.0 mL) was added. The mixture was stirred at ambient temperature overnight, and then quenched by careful addition of saturated aqueous sodium bicarbonate (100 mL) at 0 °C until no gas generated. The mixture was extracted with DCM (150 mL × 2), and the combined organic layers were dried over Na_2SO_4, filtered, and concentrated *in vacuo*. The residue was purified by flash column chromatography to afford **16-3** (5.84 g, 20.7 mmol) in 80% yield; R_f = 0.53 (silica gel, ethyl acetate/hexanes = 1/16). ^{1}H NMR (500 MHz, $CDCl_3$) δ = 2.54 – 2.41 (m, 2H), 2.43 – 2.33 (m, 2H), 2.08 (t, *J* = 6.8 Hz, 4H), 0.21 (s, 9H), 0.17 (s, 9H); ^{13}C NMR (125 MHz, $CDCl_3$) δ = 210.4, 107.7, 90.5, 67.5, 40.1, 37.3, 1.7, -0.3 ppm; IR v_{max} (film): 2963, 1724, 1350, 1252, 1103, 1043, 999, 951, 879, 843, 760 cm^{-1}; HRMS (ESI) m/z calcd for $C_{14}H_{26}O_2NaSi_2$ $[M+Na]^+$: 305.1364; found: 305.1363.

Compound 16-4 and 16-4'. To a flame dried round-bottom flask containing ketone **16-3** (2.00 g, 7.09 mmol, 1.0 equiv) was added anhydrous THF (70 mL), and the mixture was cooled to -20 °C. Methylmagnesium chloride (4.7 mL, 14.2 mmol, 2.0 equiv, 3.0 M in diethyl ether) was added into the solution, and the reaction mixture was warmed up to ambient temperature slowly during 4 h. The reaction was quenched with saturated aqueous ammonium chloride (40 mL), and the organic layer was separated. The aqueous phase was extracted with ethyl acetate (60 mL × 2). The combined organic extracts were washed with brine (20 mL), dried over Na_2SO_4 and filtered, and the solvents were removed *in vacuo*. Purification by flash column chromatography afforded the desired alcohol **16-4** (1.12 g, 3.76 mmol) in 53% yield along with its diastereoisomer **16-4'** (700 mg, 2.35 mmol) in 33% yield.

16-4: R_f = 0.40 (silica gel, ethyl acetate/hexanes = 1/8); ^{1}H NMR (500 MHz, $CDCl_3$) δ = 1.96 – 1.85 (m, 2H), 1.72 – 1.64 (m, 4H), 1.57 – 1.45 (m, 2H), 1.22 (s, 3H), 0.17 (s, 9H), 0.16 (s, 9H); ^{13}C NMR

(125 MHz, $CDCl_3$) δ = 110.3, 88.5, 69.0, 68.1, 36.6, 34.8, 29.6, 1.8, -0.2 ppm; IR ν_{max} (film): 3275, 2963, 2945, 1275, 1248, 1090, 1030, 968, 883, 839, 748 cm^{-1}; HRMS (ESI) m/z calcd for $C_{15}H_{30}O_2NaSi_2$ $[M+Na]^+$: 321.1677; found: 321.1674.

16-4': R_f = 0.35 (silica gel, ethyl acetate/hexanes = 1/8); ^1H NMR (500 MHz, $CDCl_3$) δ = 1.91 – 1.80 (m, 2H), 1.77 – 1.68(m, 2H), 1.69 – 1.57 (m, 4H), 1.22 (s, 3H), 0.19 (s, 9H), 0.16 (s, 9H); ^{13}C NMR (125 MHz, $CDCl_3$) δ = 109.0, 89.9, 69.9, 68.6, 37.0, 36.4, 29.9, 2.1, -0.2 ppm; IR ν_{max} (film): 3275, 2963, 2945, 1275, 1248, 1090, 1030, 968, 883, 839, 748 cm^{-1}; HRMS (ESI) m/z calcd for $C_{15}H_{30}O_2NaSi_2$ $[M+Na]^+$: 321.1677; found: 321.1674.

Compound 16a. TBAF (5.0 mL, 5.04 mmol, 3.0 equiv, 1.0 M in THF) was added into a stirred solution of alcohol **16-4** (500 mg, 1.68 mmol, 1.0 equiv) in anhydrous THF (15 mL) at 0 °C. The reaction mixture was warmed up to ambient temperature, and upon TLC showed complete consumption of the starting material (about 2 h), the reaction was quenched with saturated aqueous ammonium chloride (15 mL). The organic layer was separated, and the aqueous phase was extracted with ethyl acetate (25 mL × 3). The combined organic extracts were dried over Na_2SO_4, filtered, and concentrated *in vacuo*. The residue was purified by flash column chromatography to afford alkyndiol **16a** (217 mg, 1.41 mmol) in 84% yield; R_f = 0.30 (silica gel, ethyl acetate/hexanes = 1/2). ^1H NMR (400 MHz, CD_3OD) δ = 2.77 (s, 1H), 2.04 – 1.94 (m, 2H), 1.75 – 1.62 (m, 4H), 1.62 – 1.51 (m, 2H), 1.20 (s, 3H); ^{13}C NMR (100 MHz, CD_3OD) δ = 89.6, 71.9, 69.4, 67.0, 36.2, 35.3, 29.5 ppm; IR ν_{max} (film): 3262, 2972, 2949, 1738, 1364, 1261, 1217, 1132, 1007, 947, 764, 750 cm^{-1}; HRMS (ESI) m/z calcd for $C_9H_{15}O_2$ $[M+H]^+$: 155.1067; found: 155.1064.

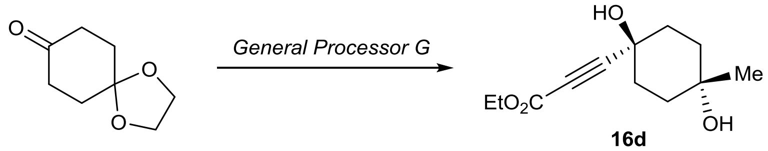

16d

Synthesis of 16d. Substrate **16d** was synthesized following the general procedure G, except that ethyl propiolate was used to replace ethynyltrimethylsilane; R_f = 0.27 (silica gel, ethyl acetate/hexanes = 1/2). ^1H NMR (400 MHz, CD_3OD) δ = 4.20 (q, J = 7.2 Hz, 2H), 2.10 – 1.95 (m, 2H), 1.79 – 1.62 (m, 4H), 1.60 – 1.48(m, 2H), 1.28 (t, J = 7.2 Hz, 3H), 1.20 (s, 3H); ^{13}C NMR (100 MHz, CD_3OD) δ = 155.1, 92.6, 75.3, 69.1, 66.8, 63.0, 35.1, 34.8, 29.7, 14.3 ppm; IR ν_{max} (film): 3372, 2972, 2936, 2492, 2241, 2220, 2075, 1713, 1697, 1369, 1267, 1123, 980 cm^{-1}; HRMS (ESI) m/z calcd for $C_{12}H_{18}O_4Na$ $[M+Na]^+$: 249.1097; found: 249.1097.

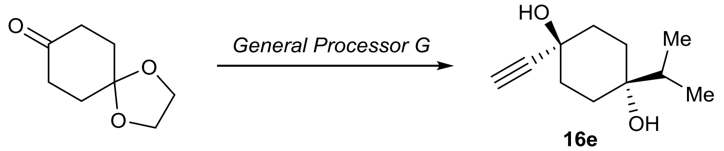

16e

Synthesis of 16e. Substrate **16e** was synthesized following the general procedure G, except that isopropylmagnesium chloride (2.0 M in THF) was used to replace methylmagnesium chloride; R_f = 0.32 (silica gel, ethyl acetate/hexanes = 1/4). ^1H NMR (400 MHz, CD_3OD) δ = 2.72 (s, 1H), 2.09 – 1.95 (m, 2H), 1.81 – 1.66 (m, 4H), 1.66 – 1.51 (m, 1H), 1.48 – 1.38 (m, 2H), 0.92 (s, 3H), 0.90 (s, 3H); ^{13}C NMR (100 MHz, CD_3OD) δ = 90.3, 73.1, 71.1, 66.4, 38.9, 35.2, 29.4, 17.3 ppm; IR ν_{max} (film): 3358, 3306, 2963, 2936, 2884, 2492, 2241, 2075, 1437, 1370, 1260, 1123, 978, 820 cm^{-1}; HRMS (ESI) m/z calcd for $C_{11}H_{18}O_2Na$ $[M+Na]^+$: 205.1199; found: 205.1199.

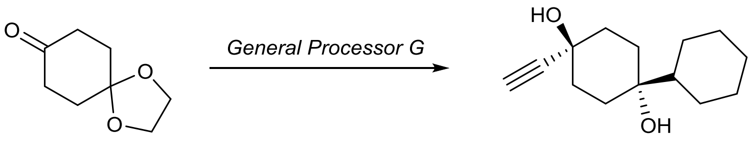

16f

Synthesis of 16f. Substrate **16f** was synthesized following the general procedure G, except that cyclohexylmagnesium bromide (1.0 M in THF) was used to replace methylmagnesium chloride; R_f = 0.33 (silica gel, ethyl acetate/hexanes = 1/4). ^{1}H NMR (400 MHz, $CDCl_3$) δ = 2.44 (s, 1H), 2.15 – 2.02 (m, 2H), 1.85 – 1.78 (m, 5H), 1.75 – 1.60 (m, 3H), 1.48 – 1.37 (m, 2H), 1.31 – 1.10 (m, 4H), 1.09 – 0.94 (m, 3H); ^{13}C NMR (100 MHz, $CDCl_3$) δ = 88.8, 72.1, 70.6, 66.0, 48.1, 34.3, 29.1, 26.7, 26.5, 26.4 ppm; IR ν_{max} (film): 3358, 2941, 2835, 2602, 2553, 2048, 1657, 1452, 1415, 1113, 1032 cm^{-1}; HRMS (ESI) m/z calcd for $C_{14}H_{22}O_2Na$ $[M+Na]^+$: 245.1512; found: 245.1512.

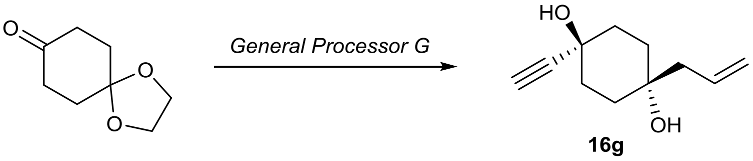

16g

Synthesis of 16g. Substrate **16g** was synthesized following the general procedure G, except that allylmagnesium chloride (1.7 M in THF) was used to replace methylmagnesium chloride; R_f = 0.16 (silica gel, ethyl acetate/hexanes = 1/4). ^{1}H NMR (400 MHz, CD_3OD) δ = 5.90 (ddt, *J* = 17.8, 10.5, 7.3 Hz, 1H), 5.12 – 4.98 (m, 2H), 2.76 (s, 1H), 2.21 (d, *J* = 7.3 Hz, 2H), 2.07 – 1.87 (m, 2H), 1.81 – 1.59 (m, 4H), 1.59 – 1.42 (m, 2H); ^{13}C NMR (100 MHz, CD_3OD) δ = 135.2, 118.1, 89.8, 71.7, 70.9, 66.8, 47.9, 35.6, 32.9 ppm; IR ν_{max} (film): 3304, 2964, 2938, 2367, 2340, 1736, 1431, 1366, 1277, 1227, 1217, 1070, 750 cm^{-1}; HRMS (ESI) m/z calcd for $C_{11}H_{16}O_2Na$ $[M+Na]^+$: 203.1043; found: 203.1052.

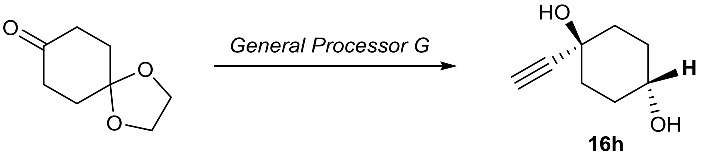

16h

Synthesis of 16h. Substrate **16h** was synthesized following the general procedure G, except that reduction of ketone **16-3** by $NaBH_4$ (1.0 equiv) in dry EtOH (0.15 M) at -78 °C for 3 h was employed to replace the nucleophilic addition of the ketone by methylmagnesium chloride; R_f = 0.10 (silica gel, ethyl acetate/hexanes = 1/2). ^{1}H NMR (400 MHz, CD_3OD) δ = 3.68 – 3.50 (m, 1H), 2.87 (s, 1H), 2.05 – 1.74 (m, 4H), 1.72 – 1.44 (m, 4H); ^{13}C NMR (100 MHz, CD_3OD) δ = 88.2, 73.8, 69.6, 68.5, 38.1, 32.4 ppm; IR ν_{max} (film): 3371, 3310, 2932, 2866, 2492, 1628, 1443, 1368, 1148, 1076, 955, 900, 646 cm^{-1}; HRMS (ESI) m/z calcd for $C_8H_{12}O_2Na$ $[M+Na]^+$: 163.0730; found: 163.0729.

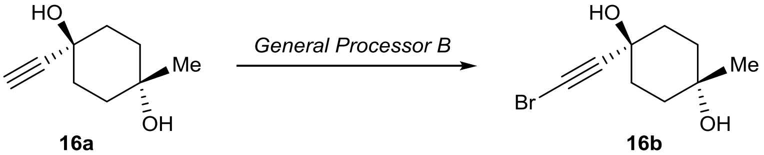

16a **16b**

Synthesis of 16b. Substrate **16b** was synthesized following the general procedure B, except that **16a** was used to replace **9**; R_f = 0.38 (silica gel, ethyl acetate/hexanes = 1/2). ^{1}H NMR (400 MHz, CD_3OD) δ = 2.03 – 1.90 (m, 2H), 1.74 – 1.61 (m, 4H), 1.61 – 1.48 (m, 2H), 1.19 (s, 3H); ^{13}C NMR (100 MHz, CD_3OD) δ = 85.7, 69.3, 68.2, 43.7, 36.0, 35.3, 29.4 ppm; IR ν_{max} (film): 3368, 3302, 2972, 2932, 2361, 1738, 1440, 1364, 1215, 1110, 1065, 750 cm^{-1}; HRMS (ESI) m/z calcd for $C_9H_{14}BrO_2$ $[M+H]^+$: 233.0172; found: 233.0174.

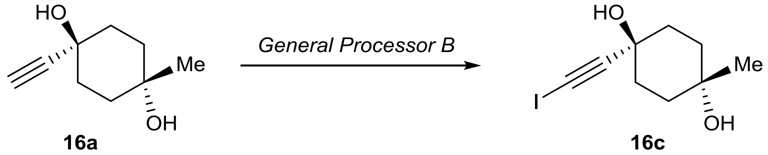

Synthesis of 16c. Substrate **16c** was synthesized following the general procedure B, except that **16a** was used to replace **9** and NIS was used to replace NBS; R_f = 0.37 (silica gel, ethyl acetate/hexanes = 1/2). ^1H NMR (400 MHz, CD_3OD) δ = 2.02 – 1.89 (m, 2H), 1.75 – 1.60 (m, 4H), 1.60 – 1.48 (m, 2H), 1.19 (s, 3H); ^{13}C NMR (100 MHz, CD_3OD) δ = 99.4, 69.3, 68.8, 36.2, 35.3, 29.3, 3.4 ppm; IR ν_{max} (film): 3375, 2967, 2945, 2365, 1740, 1437, 1364, 1261, 1229, 1217, 1009, 750 cm^{-1}; HRMS (ESI) m/z calcd for $C_9H_{13}O_2NaI$ $[M+Na]^+$: 302.9852; found: 302.9860.

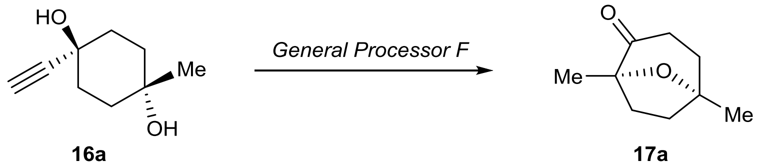

Synthesis of 17a. Product **17a** was obtained in 86% yield from substrate **16a** following the general procedure F; R_f = 0.57 (silica gel, ethyl acetate/hexanes = 1/4). ^1H NMR (400 MHz, $CDCl_3$) δ = 2.55 – 2.35 (m, 2H), 2.15 – 2.01 (m, 2H), 2.00 – 1.82 (m, 4H), 1.43 (s, 3H), 1.35 (s, 3H); ^{13}C NMR (100 MHz, $CDCl_3$) δ = 208.6, 86.0, 80.9, 38.8, 36.2, 35.7, 33.1, 26.1, 19.1 ppm; IR ν_{max} (film): 2970, 2932, 2849, 1757, 1722, 1452, 1375, 1275, 1260, 1240, 1096, 937, 862 cm^{-1}; HRMS (ESI) m/z calcd for $C_9H_{15}O_2$ $[M+H]^+$: 154.0994; found: 155.1068.

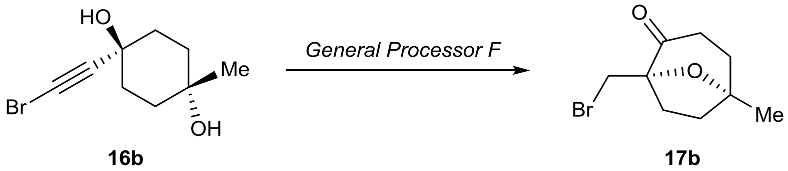

Synthesis of 17b. Product **17b** was obtained in 81% yield from substrate **16b** following the general procedure F; R_f = 0.70 (silica gel, ethyl acetate/hexanes = 1/2). ^1H NMR (400 MHz, $CDCl_3$) δ = 3.70 (d, J = 11.2 Hz, 1H), 3.60 (d, J = 11.2 Hz, 1H), 2.58 – 2.41 (m, 2H), 2.28 – 2.02 (m, 3H), 2.01 – 1.87 (m, 3H), 1.47 (s, 3H); ^{13}C NMR (100 MHz, $CDCl_3$) δ = 206.7, 86.3, 81.8, 38.1, 35.9, 33.5, 33.4, 32.7, 26.0 ppm; IR ν_{max} (film): 2976, 2918, 2848, 1721, 1449, 1420, 1274, 1263, 1099, 905, 764, 748 cm^{-1}; HRMS (ESI) m/z calcd for $C_9H_{13}O_2NaBr$ $[M+Na]^+$: 254.9991; found: 254.9990.

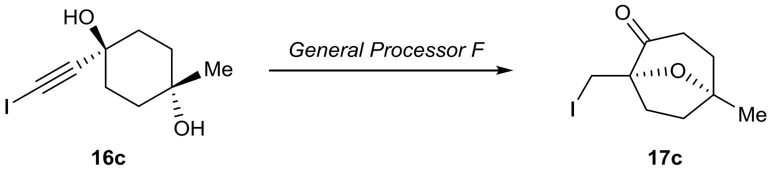

Synthesis of 17c. Product **17c** was obtained in 78% yield from substrate **16c** following the general procedure F; R_f = 0.66 (silica gel, ethyl acetate/hexanes = 1/2). ^1H NMR (400 MHz, $CDCl_3$) δ = 3.53 (d, J = 11.0 Hz, 1H), 3.41 (d, J = 11.0 Hz, 1H), 2.58 – 2.41 (m, 2H), 2.20 – 2.05 (m, 3H), 2.04 – 1.90 (m, 3H), 1.46 (s, 3H); ^{13}C NMR (100 MHz, $CDCl_3$) δ = 206.0, 85.7, 81.8, 38.4, 36.2, 35.0, 33.5, 26.0, 7.0 ppm; IR ν_{max} (film): 2924, 2857, 2361, 2334, 1722, 1415, 1375, 1094, 1012, 897, 764, 748cm^{-1}; HRMS (ESI) m/z calcd for $C_9H_{13}O_2NaI$ $[M+Na]^+$: 302.9852; found: 302.9852.

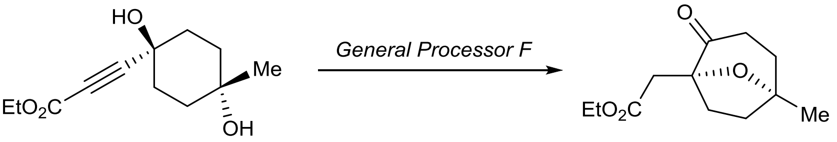

16d 17d

Synthesis of 17d. Product **17d** was obtained in 73% yield from substrate **16d** following the general procedure F, except that the reaction was carried out in tube sealing at 70 °C for 2 h; R_f = 0.65 (silica gel, ethyl acetate/hexanes = 1/2). ^{1}H NMR (400 MHz, $CDCl_3$) δ = 4.13 (q, *J* = 7.1 Hz, 2H), 2.77 (s, 2H), 2.54 – 2.44 (m, 2H), 2.24 – 2.09 (m, 3H), 2.09 – 2.00 (m, 1H), 1.95 – 1.84 (m, 2H), 1.43 (s, 3H), 1.25 (t, *J* = 7.1 Hz, 3H); ^{13}C NMR (100 MHz, $CDCl_3$) δ = 207.8, 170.2, 86.0, 81.0, 60.5, 38.4, 38.3, 35.5, 34.5, 33.0, 26.1, 14.1 ppm; IR v_{max} (film): 2941, 2853, 2369, 2347, 1722, 1370, 1263, 1184, 1092, 1030, 748 cm^{-1}; HRMS (ESI) m/z calcd for $C_{12}H_{18}O_4Na$ $[M+Na]^+$: 249.1097; found: 249.1099.

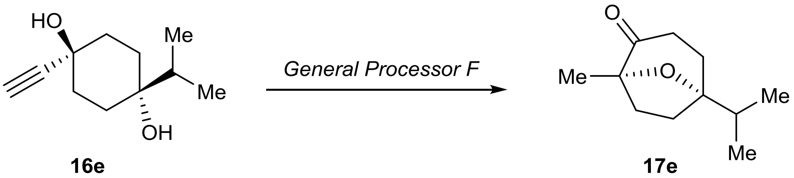

16e 17e

Synthesis of 17e. Product **17e** was obtained in 85% yield from substrate **16e** following the general procedure F; R_f = 0.88 (silica gel, ethyl acetate/hexanes = 1/4). ^{1}H NMR (400 MHz, $CDCl_3$) δ = 2.46 – 2.34 (m, 2H), 2.06 – 1.81 (m, 6H), 1.81 – 1.69 (m, 1H), 1.29 (s, 3H), 0.94 (d, *J* = 6.4 Hz, 3H), 0.92 (d, *J* = 6.4 Hz, 3H); ^{13}C NMR (100 MHz, $CDCl_3$) δ = 209.9, 85.9, 85.7, 36.0, 35.8, 33.0, 32.9, 31.8, 19.1, 17.8, 17.3 ppm; IR v_{max} (film): 2926, 2961, 2851, 1452, 1273, 1261, 762, 750 cm^{-1}; HRMS (ESI) m/z calcd for $C_{11}H_{18}O_2Na$ $[M+Na]^+$: 205.1199; found: 205.1197.

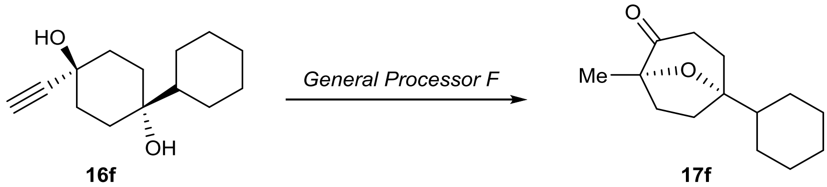

16f 17f

Synthesis of 17f. Product **17f** was obtained in 86% yield from substrate **16f** following the general procedure F; R_f = 0.77 (silica gel, ethyl acetate/hexanes = 1/4). ^{1}H NMR (400 MHz, $CDCl_3$) δ = 2.47 – 2.37 (m, 2H), 2.10 – 1.98 (m, 1H), 1.98 – 1.85 (m, 4H), 1.84 – 1.71 (m, 5H), 1.70 – 1.65 (m, 1H), 1.63 – 1.52 (m, 1H), 1.32 (s, 3H), 1.29 – 1.19 (m, 2H), 1.18 – 1.09 (m, 1H), 1.08 – 0.96 (m, 2H); ^{13}C NMR (100 MHz, $CDCl_3$) δ = 210.0, 85.8, 85.3, 46.9, 35.7, 33.5, 33.0, 32.6, 28.0, 27.5, 26.5, 26.4, 26.3, 19.1 ppm; IR v_{max} (film): 2928, 2849, 1722, 1557, 1443, 1416, 1375, 1258, 1101, 1038, 999, 885, 795, 750 cm^{-1}; HRMS (ESI) m/z calcd for $C_{14}H_{23}O_2$ $[M+H]^+$: 223.1693; found: 223.1701.

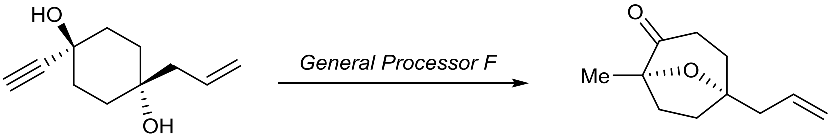

16g 17g

Synthesis of 17g. Product **17g** was obtained in 86% yield from substrate **16g** following the general procedure F; R_f = 0.70 (silica gel, ethyl acetate/hexanes = 1/4). ^{1}H NMR (400 MHz, $CDCl_3$) δ = 5.83 (ddt, *J* = 17.6, 10.4, 7.2 Hz, 1H), 5.16 – 5.07 (m, 2H), 2.52 – 2.32 (m, 4H), 2.13 – 1.77 (m, 6H), 1.34 (s, 3H); ^{13}C NMR (100 MHz, $CDCl_3$) δ = 208.9, 133.5, 118.2, 86.1, 82.5, 44.0, 36.8, 35.8, 33.4, 33.0,

19.1 ppm; IR ν_{max} (film): 2984, 2953, 2853, 2359, 1769, 1759, 1724, 1379, 1275, 1260, 1246, 1051, 997, 916, 766, 752 cm^{-1}; HRMS (ESI) m/z calcd for $C_{11}H_{16}O_2Na$ $[M+Na]^+$: 203.1043; found: 203.1041.

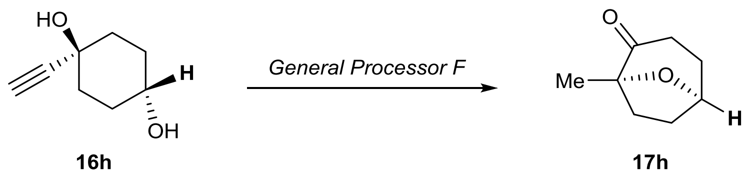

Synthesis of 17h. Product **17h** was obtained in 77% yield from substrate **16h** following the general procedure F, except that the reaction was carried out in tube sealing at 70 °C for 2 h; R_f = 0.30 (silica gel, ethyl acetate/hexanes = 1/8). ^1H NMR (400 MHz, $CDCl_3$) δ = 4.58 – 4.52 (m, 1H), 2.48 – 2.34 (m, 2H), 2.33 – 2.20 (m, 2H), 2.01 – 1.75 (m, 4H), 1.36 (s, 3H); ^{13}C NMR (100 MHz, $CDCl_3$) δ = 208.4, 86.0, 75.0, 35.2, 32.7, 32.3, 29.3, 18.8 ppm; IR ν_{max} (film): 2938, 2876, 2361, 2340, 1722, 1285, 1155, 1109, 1038, 993, 968, 871, 750 cm^{-1}; HRMS (ESI) m/z calcd for $C_8H_{12}O_2Na$ $[M+Na]^+$: 163.0730; found: 163.0734.

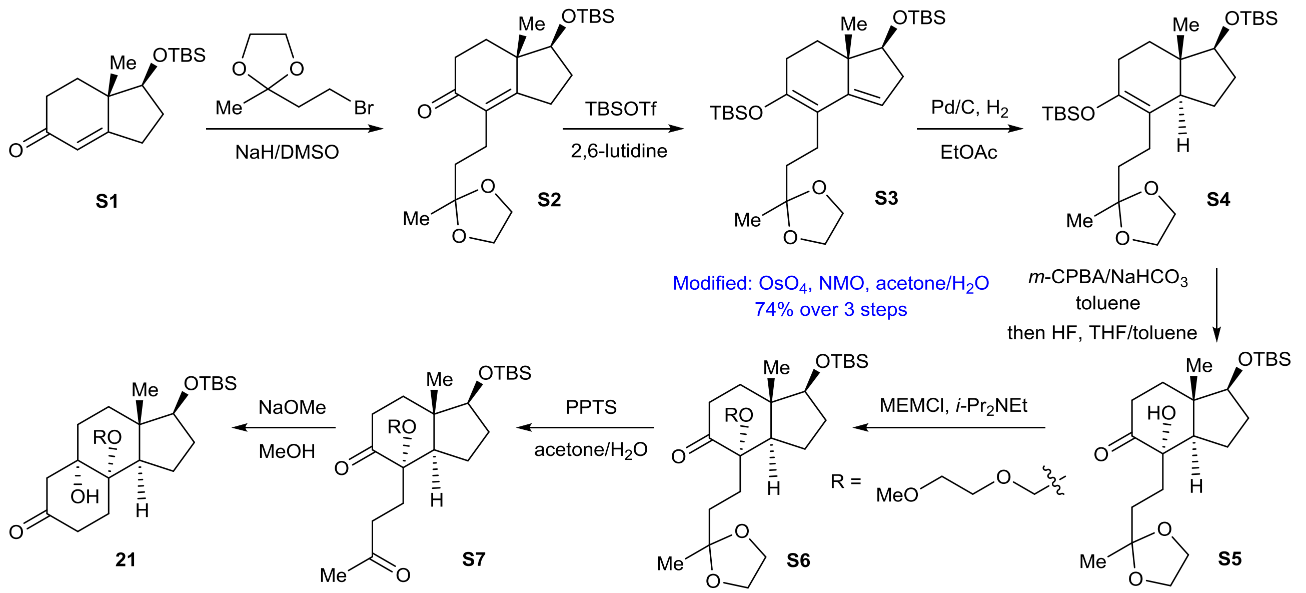

Compound 21. Ketone **21** was synthesized following the procedure developed by Shair group[9], except a modification[10] was made for the transformation of **S4** to **S5**: To a stirred solution of crude **S4** (11.7 g, 23.0 mmol, 1.0 equiv) in acetone/H_2O (240 mL/30 mL) at ambient temperature were added sequentially with NMO (5.38 g, 46.0 mmol, 2.0 equiv) and OsO_4 (12.7 mL, 4.60 mmol, 0.2 equiv, 4 wt.% in H_2O). The reaction mixture was warmed up to 50 °C, and stirred for 18 h. The reaction mixture was quenched with saturated aqueous Na_2SO_3 (50 mL) at 0 °C, and diluted with water (70 mL). The organic layer was separated, and the aqueous phase was extracted with ethyl acetate (200 mL × 3). The combined organic layers were dried over Na_2SO_4, filtered, and concentrated *in vacuo*. The residue was purified by flash column chromatography to afford **S5** (7.86 g, 19.1 mmol) in 83% yield, which is also 74% yield over 3 steps from **S2**. For ketone **21**, R_f = 0.43 (silica gel, ethyl acetate/hexanes = 1/2); $[\alpha]_D^{20}$ = 4.4 (*c* 0.5, $CHCl_3$).

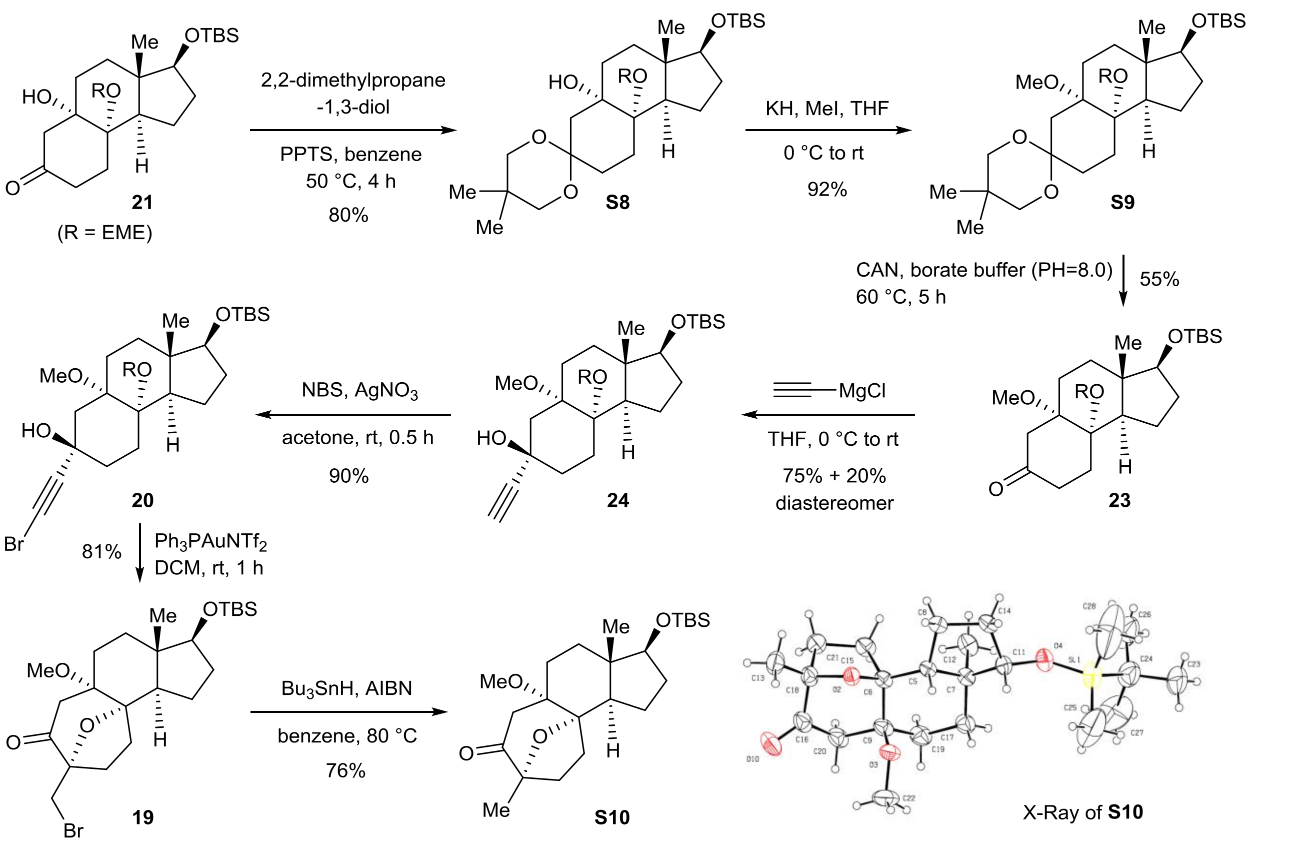

Compound S8. A mixture of ketone **21** (2.30 g, 5.04 mmol, 1.0 equiv), 2,2-dimethylpropane-1,3-diol (5.24 g, 50.4 mmol, 10.0 equiv) and PPTS (127 mg, 0.50 mmol, 0.1 equiv) in dry benzene (50 mL) was heated to 50 °C, and stirred for 5 h. After cooled to ambient temperature, the mixture was concentrated *in vacuo* to give a raffinate (about 8.0 mL), which was directly purified by flash column chromatography to afford **S8** (2.18 g, 4.03 mmol) in 80% yield. R_f = 0.56 (silica gel, ethyl acetate/hexanes = 1/2); $[\alpha]_D^{20}$ = -30.2 (*c* 0.6, $CHCl_3$); ^{1}H NMR (400 MHz, $CDCl_3$) δ = 4.88 (d, *J* = 7.2 Hz, 1H), 4.74 (d, *J* = 7.2 Hz, 1H), 3.78 (ddd, *J* = 10.4, 4.8, 4.8 Hz, 1H), 3.68 (ddd, *J* = 10.4, 4.8, 4.8 Hz, 1H), 3.62 (d, *J* = 11.6 Hz, 1H), 3.58 (d, *J* = 11.2 Hz, 1H), 3.57 (t, *J* = 8.4 Hz, 1H), 3.52 (t, *J* = 4.8 Hz, 2H), 3.45 (d, *J* = 2.0 Hz, 1H), 3.38 (dd, *J* = 5.2, 1.6 Hz, 1H), 3.34 (dd, *J* = 5.2, 1.6 Hz, 1H), 3.35 (s, 3H), 2.44 (d, *J* = 13.6 Hz, 1H), 2.20 (ddd, *J* = 14.0, 14.0, 4.4 Hz, 1H), 2.08 (dd, *J* = 11.6, 8.8 Hz, 1H), 1.91 – 1.72 (m, 5H), 1.65 (d, *J* = 14.0 Hz, 1H), 1.56 (dd, *J* = 11.6, 5.6 Hz, 1H), 1.53 (dd, *J* = 11.6, 5.6 Hz, 1H), 1.49 – 1.39 (m, 2H), 1.35 – 1.21 (m, 2H), 1.06 (s, 3H), 0.84 (s, 9H), 0.82 (s, 3H), 0.79 (s, 3H), -0.02 (s, 3H), -0.03 (s, 3H); ^{13}C NMR (100 MHz, $CDCl_3$) δ = 97.8, 89.7, 82.0, 81.1, 74.2, 71.7, 70.1, 70.0, 67.7, 59.0, 44.8, 44.4, 39.7, 31.4, 30.3, 30.1, 30.0, 25.8, 23.8, 23.0, 22.4, 20.1, 18.0, 12.3, -4.6, -4.8 ppm; IR ν_{max} (film): 3451, 2955, 2928, 1472, 1393, 1273, 1254, 1175, 1111, 1092, 1038, 835, 766, 750 cm^{-1}; HRMS (ESI) m/z calcd for $C_{29}H_{54}O_7NaSi$ $[M+Na]^+$: 565.3531; found: 565.3531.

Compound S9. To a solution of KH (480 mg, 12.0 mmol, 4.0 equiv) in anhydrous THF (30 mL) at 0 °C was added **S8** (1.63 g, 3.00 mmol, 1.0 equiv) in THF (5.0 mL), and the reaction was stirred for 1 h at 0 °C. Then MeI (1.5 mL, 24.0 mmol, 8.0 equiv) was added into the reaction, and the mixture was warmed up to ambient temperature slowly. After 4 h, the reaction was quenched with saturated aqueous ammonium chloride (30 mL). The mixture was extracted with ethyl acetate (40 mL × 3), and the combined organic layers were dried over Na_2SO_4, filtered, and concentrated *in vacuo*. Purification

by flash column chromatography afforded the desired ketal **S9** (1.53 g, 2.76 mmol) in 92% yield. R_f = 0.45 (silica gel, ethyl acetate/hexanes = 1/4); $[\alpha]_D^{20}$ = 6.7 (*c* 0.5, $CHCl_3$); ^1H NMR (400 MHz, $CDCl_3$) δ = 5.02 (d, *J* = 6.4 Hz, 1H), 4.82 (d, *J* = 6.4 Hz, 1H), 3.78 (ddd, *J* = 10.4, 4.4, 4.4 Hz, 1H), 3.59 (ddd, *J* = 10.4, 4.4, 4.4 Hz, 1H), 3.56 – 3.46 (m, 5H), 3.46 – 3.42 (m, 1H), 3.44 (s, 1H), 3.34 (s, 3H), 3.12 (s, 3H), 2.35 (d, *J* = 12.8 Hz, 1H), 2.18 (dd, *J* = 12.8, 6.8 Hz, 1H), 2.01 (ddd, *J* = 14.6, 14.6, 3.6 Hz, 1H), 1.96 – 1.88 (m, 2H), 1.84 – 1.78 (m, 1H), 1.78 – 1.72 (m, 1H), 1.74 (d, *J* = 13.2 Hz, 1H), 1.71 – 1.61 (m, 2H), 1.61 – 1.55 (m, 1H), 1.51 (dddd, *J* = 12.0, 12.0, 12.0, 5.6 Hz, 1H), 1.36 – 1.18 (m, 2H), 1.10 (ddd, *J* = 13.2, 13.2, 2.8 Hz, 1H), 0.95 (s, 3H), 0.93 (s, 3H), 0.84 (s, 9H), 0.80 (s, 3H), -0.02 (s, 3H), -0.03 (s, 3H); ^{13}C NMR (100 MHz, $CDCl_3$) δ = 98.3, 90.7, 81.4, 80.4, 79.4, 71.9, 70.1, 69.8, 67.0, 58.9, 47.4, 44.5, 42.8, 35.6, 31.3, 30.1, 30.0, 27.5, 26.3, 25.8, 25.4, 22.8, 22.7, 20.0, 18.0, 12.7, -4.5, -4.8 ppm; IR ν_{max} (film): 2955, 2928, 2857, 1734, 1472, 1462, 1371, 1260, 1125, 1092, 1040, 835, 871, 766 cm^{-1}; HRMS (ESI) m/z calcd for $C_{30}H_{56}O_7NaSi$ $[M+Na]^+$: 579.3688; found: 579.3690.

Compound 23. Solid cerium ammonium nitrate (46 mg, 0.084 mmol, 0.05 equiv) was added to a stirred solution of ketal **S9** (930 mg, 1.67 mmol, 1.0 equiv) in MeCN (5.5 mL) and borate-HCl buffer (Merck, pH 8, 5.5 mL). The faintly yellow solution was heated at 60 °C for 5 h. After cooling to room temperature, water (12 mL) was added. The organic layer was separated, and the aqueous phase was extracted with DCM (25 mL × 3). The combined organic extracts were dried over Na_2SO_4 and filtered, and the solvents were removed *in vacuo*. The crude product was further purified by column chromatography on silica gel to give the ketone **23** (430 mg, 0.92 mmol) in 55% yield.[11] R_f = 0.62 (silica gel, ethyl acetate/hexanes = 1/2); $[\alpha]_D^{20}$ = 16.2 (*c* 0.5, $CHCl_3$); ^1H NMR (400 MHz, $CDCl_3$) δ = 5.12 (d, *J* = 6.8 Hz, 1H), 4.93 (d, *J* = 6.8 Hz, 1H), 3.83 (ddd, *J* = 10.8, 4.8, 4.8 Hz, 1H), 3.65 (ddd, *J* = 10.8, 4.8, 4.8 Hz, 1H), 3.58 (t, *J* = 8.4 Hz, 1H), 3.53 (t, *J* = 4.8 Hz, 2H), 3.35 (s, 3H), 3.13 (s, 3H), 2.93 (ddd, *J* = 13.6, 13.6, 6.4 Hz, 1H), 2.91 (d, *J* = 12.8, 1H), 2.46 (dd, *J* = 12.8, 2.0 Hz, 1H), 2.31 (dd, *J* = 12.8, 6.8 Hz, 1H), 2.17 – 2.06 (m, 2H), 1.98 (ddd, *J* = 14.4, 14.4, 4.8 Hz, 1H), 1.89 (dddd, *J* = 14.4, 9.6, 9.2, 4.6 Hz, 1H), 1.79 (ddd, *J* = 15.2, 3.2, 3.2 Hz, 1H), 1.69 – 1.62 (m, 1H), 1.57 (dddd, *J* = 12.4, 12.4, 12.4, 5.6 Hz, 1H), 1.42 – 1.26 (m, 3H), 1.17 (ddd, *J* = 13.2, 13.2, 3.6 Hz, 1H), 0.85 (s, 9H), 0.81 (s, 3H), 0.00 (s, 6H); ^{13}C NMR (100 MHz, $CDCl_3$) δ = 210.3, 91.0, 82.1, 81.1, 80.2, 71.8, 67.4, 59.0, 48.0, 47.0, 44.5, 43.2, 36.6, 31.1, 30.2, 27.6, 27.4, 25.8, 20.3, 18.0, 13.0, -4.5, -4.8 ppm; IR ν_{max} (film): 2955, 2928, 1715, 1275, 1260, 1099, 1016, 764, 748 cm^{-1}; HRMS (ESI) m/z calcd for $C_{25}H_{46}O_6NaSi$ $[M+Na]^+$: 493.2956; found: 493.2956.

Compound 24. Ketone **23** (1.00 g, 2.13 mmol, 1.0 equiv) was dissolved in anhydrous THF (30 mL), and cooled to 0 °C. Ethynylmagnesium chloride (10.7 mL, 6.39 mmol, 3.0 equiv, 0.6 M in THF) was added, and the reaction mixture was warmed up to ambient temperature slowly during 4 h. The reaction was quenched with saturated aqueous ammonium chloride (30 mL), and the mixture was extracted with ethyl acetate (40 mL × 3). The combined organic layers were dried over Na_2SO_4, filtered, and concentrated *in vacuo*. Purification by flash column chromatography afforded the alkyndiol **24** (794 mg, 1.60 mmol) in 75% yield together with its diastereoisomer **24’** (213 mg, 0.43 mmol) in 20% yield.

24: R_f = 0.70 (silica gel, ethyl acetate/hexanes = 1/2); $[\alpha]_D^{20}$ = 15.0 (*c* 0.5, $CHCl_3$); ^1H NMR (500 MHz, $CDCl_3$) δ = 5.02 (d, *J* = 6.5 Hz, 1H), 4.84 (d, *J* = 6.5 Hz, 1H), 3.78 (ddd, *J* = 10.5, 5.0, 5.0 Hz, 1H), 3.62 (ddd, *J* = 10.5, 5.0, 5.0 Hz, 1H), 3.57 (t, *J* = 8.5 Hz, 1H), 3.54 (t, *J* = 5.0 Hz, 2H), 3.37 (s, 3H), 3.12 (s, 3H), 2.44 (s, 1H), 2.33 (ddd, *J* = 14.0, 14.0, 4.0 Hz, 1H), 2.23 (dd, *J* = 12.5, 7.5 Hz, 1H),

2.21 – 2.15 (m, 1H), 2.13 (d, J = 13.5 Hz, 1H), 2.03 (dd, J = 13.5, 2.5 Hz, 1H), 2.00 – 1.89 (m, 2H), 1.86 (dddd, J = 14.0, 9.5, 9.0, 4.5 Hz, 1H), 1.73 – 1.63 (m, 3H), 1.61 – 1.55 (m, 1H), 1.53 (dddd, J = 12.5, 12.5, 12.5, 5.5 Hz, 1H), 1.41 – 1.29 (m, 1H), 1.26 (ddd, J = 12.5, 3.0, 3.0 Hz, 1H), 1.11 (ddd, J = 13.0, 13.0, 3.0 Hz, 1H), 0.86 (s, 9H), 0.85 (s, 3H), 0.00 (s, 3H), -0.01 (s, 3H); ^{13}C NMR (100 MHz, $CDCl_3$) δ = 90.8, 88.9, 81.4, 80.1, 78.5, 71.9, 70.2, 67.2, 67.0, 58.9, 47.4, 44.6, 42.8, 42.2, 33.8, 31.5, 30.2, 27.7, 25.8, 24.7, 19.9, 18.0, 12.7, -4.5, -4.8 ppm; IR ν_{max} (film): 3437, 3310, 2932, 2883, 2857, 1736, 1462, 1368, 1275, 1217, 1157, 1086, 1040, 870, 835, 773 cm^{-1}; HRMS (ESI) m/z calcd for $C_{27}H_{48}O_6NaSi$ $[M+Na]^+$: 519.3112; found: 519.3112.
24': R_f = 0.30 (silica gel, ethyl acetate/hexanes = 1/2); $[\alpha]_D^{20}$ = 8.5 (c 0.3, $CHCl_3$); ^{1}H NMR (500 MHz, $CDCl_3$) δ = 5.01 (d, J = 6.5 Hz, 1H), 4.85 (d, J = 6.5 Hz, 1H), 3.84 (ddd, J = 10.5, 4.5, 4.5 Hz, 1H), 3.61 (ddd, J = 10.5, 4.5, 4.5 Hz, 1H), 3.58 – 3.49 (m, 3H), 3.37 (s, 3H), 3.13 (s, 3H), 2.53 (s, 1H), 2.44 (br, 1H), 2.34 (ddd, J = 14.5, 14.5, 4.0 Hz, 1H), 2.25 (ddd, J = 12.5, 12.5, 4.5 Hz, 1H), 2.20 (dd, J = 12.5, 7.0 Hz, 1H), 2.15 (dd, J = 12.5, 2.0 Hz, 1H), 2.04 (d, J = 12.0 Hz, 1H), 1.94 – 1.78 (m, 4H), 1.72 (ddd, J = 15.5, 3.0, 3.0 Hz, 1H), 1.62 – 1.47 (m, 2H), 1.38 – 1.27 (m, 2H), 1.15 (ddd, J = 13.0, 13.0, 3.0 Hz, 1H), 0.86 (s, 9H), 0.85 (s, 3H), 0.00 (s, 3H), -0.01 (s, 3H); ^{13}C NMR (125 MHz, $CDCl_3$) δ = 90.6, 88.5, 81.4, 79.8, 79.2, 72.7, 72.1, 68.0, 67.0, 58.8, 47.5, 44.6, 43.5, 43.1, 35.1, 31.5, 30.2, 26.6, 25.8, 20.1, 18.0, 12.7, -4.5, -4.8 ppm; IR ν_{max} (film): 3412, 3306, 2959, 2936, 2893, 1472, 1250, 1126, 1040, 912, 837, 777, 741, 667 cm^{-1}; HRMS (ESI) m/z calcd for $C_{27}H_{48}O_6NaSi$ $[M+Na]^+$: 519.3112; found: 519.3112.

Compound 20. The bromide **20** was obtained in 90% yield following the general procedure B from alkyndiol **24**, except that the reaction time was shorted to 0.5 h. R_f = 0.50 (silica gel, ethyl acetate/hexanes = 1/4); $[\alpha]_D^{20}$ = 13.6 (c 0.9, $CHCl_3$); ^{1}H NMR (400 MHz, $CDCl_3$) δ = 5.02 (d, J = 6.4 Hz, 1H), 4.84 (d, J = 6.4 Hz, 1H), 3.79 (ddd, J = 10.4, 4.4, 4.4 Hz, 1H), 3.62 (ddd, J = 10.4, 4.8, 4.8 Hz, 1H), 3.59 – 3.52 (m, 3H), 3.38 (s, 3H), 3.11 (s, 3H), 2.33 (ddd, J = 14.0, 14.0, 4.0 Hz, 1H), 2.22 (dd, J = 12.8, 7.6 Hz, 1H), 2.22 – 2.15 (m, 1H), 2.12 (d, J = 13.6 Hz, 1H), 2.02 (dd, J = 13.6, 2.4 Hz, 1H), 2.01 – 1.92 (m, 1H), 1.91 – 1.80 (m, 2H), 1.73 – 1.61 (m, 2H), 1.59 – 1.55 (m, 1H), 1.53 (dddd, J = 12.4, 12.4, 12.4, 5.6 Hz, 1H), 1.37 – 1.22 (m, 3H), 1.11 (ddd, J = 13.2, 13.2, 3.2 Hz, 1H), 0.85 (s, 9H), 0.84 (s, 3H), 0.00 (s, 3H), -0.01 (s, 3H); ^{13}C NMR (100 MHz, $CDCl_3$) δ = 90.7, 84.8, 81.4, 80.0, 78.5, 71.9, 68.4, 67.0, 58.9, 47.4, 44.6, 43.1, 42.8, 42.2, 33.7, 31.5, 30.2, 27.7, 25.8, 24.7, 19.9, 18.0, 12.7, -4.5, -4.8 ppm; IR ν_{max} (film): 3406, 2955, 2932, 2887, 1472, 1387, 1250, 1201, 1128, 1109, 1086, 1040, 1015, 899, 835, 773 cm^{-1}; HRMS (ESI) m/z calcd for $C_{27}H_{47}O_6NaSiBr$ $[M+Na]^+$: 597.2217; found: 597.2215.

Compound 19. The compound **19** was obtained in 81% yield following the general gold-catalyzed procedure F from bromide **20**, except that the reaction time was shorted to 1 h. R_f = 0.60 (silica gel, ethyl acetate/hexanes = 1/4); $[\alpha]_D^{20}$ = -3.3 (c 0.4, $CHCl_3$); ^{1}H NMR (400 MHz, $CDCl_3$) δ = 3.67 (d, J = 11.2 Hz, 1H), 3.65 (t, J = 8.4 Hz, 1H), 3.62 (d, J = 11.2 Hz, 1H), 3.19 (s, 3H), 2.73 (d, J = 16.0 Hz, 1H), 2.39 (dd, J = 12.4, 7.2 Hz, 1H), 2.29 (d, J = 16.0 Hz, 1H), 2.26 – 2.13 (m, 2H), 2.01 – 1.86 (m, 3H), 1.79 (ddd, J = 15.2, 3.2, 3.2 Hz, 1H), 1.71 – 1.61 (m, 1H), 1.58 – 1.43 (m, 3H), 1.40 (ddd, J = 12.8, 3.2, 3.2 Hz, 1H), 1.14 (ddd, J = 13.2, 13.2, 3.6 Hz, 1H), 0.87 (s, 9H), 0.78 (s, 3H), 0.01 (s, 6H); ^{13}C NMR (100 MHz, $CDCl_3$) δ = 204.4, 88.2, 84.2, 82.6, 81.4, 49.0, 44.4, 41.8, 40.9, 32.5, 32.1, 31.5, 30.4, 28.1, 26.1, 25.8, 19.2, 18.0, 11.9, -4.5, -4.9 ppm; IR ν_{max} (film): 2959, 2932, 1769, 1730, 1462,

1277, 1128, 1086, 1011, 835, 748 cm^{-1}; HRMS (ESI) m/z calcd for $C_{23}H_{39}O_4NaSiBr$ $[M+Na]^+$: 509.1693; found: 509.1689.

Compound S10. The mixture of **19** (150 mg, 0.31 mmol, 1.0 equiv), *n*-Bu_3SnH (0.62 mL, 2.48 mmol, 8 equiv) and 2,2-azobis(2-methylpropionitrile) (26 mg, 0.16 mmol, 0.5 equiv) in dry benzene (6 mL) was degassed by argon, and then warmed up to 80 °C. After 10 h, the mixture was cooled to ambient temperature, and concentrated *in vacuo* to give a raffinate (about 1.0 mL), which was directly purified by flash column chromatography to afford alkane **S10** (96 mg, 0.24 mmol) in 76% yield. The structure of **S10** was confirmed by singlecrystal X-ray diffraction analysis, which gave a strong support for the structure of **19**. R_f = 0.63 (silica gel, ethyl acetate/hexanes = 1/4); ^1H NMR (400 MHz, $CDCl_3$) δ = 3.64 (t, *J* = 8.0 Hz, 1H), 3.17 (s, 3H), 2.68 (d, *J* = 16.4 Hz, 1H), 2.37 (dd, *J* = 12.0, 7.2 Hz, 1H), 2.21 (d, *J* = 16.4 Hz, 1H), 2.18 – 2.09 (m, 1H), 1.98 – 1.78 (m, 4H), 1.76 (ddd, *J* = 15.2, 3.2, 3.2 Hz, 1H), 1.67 – 1.58 (m, 1H), 1.56 – 1.41 (m, 3H), 1.38 (ddd, *J* = 12.8, 3.2, 3.2 Hz, 1H), 1.33 (s, 3H), 1.13 (ddd, *J* = 13.2, 13.2, 3.6 Hz, 1H), 0.87 (s, 9H), 0.77 (s, 3H), 0.01 (s, 3H), 0.00 (s, 3H); ^{13}C NMR (100 MHz, $CDCl_3$) δ = 207.2, 87.1, 84.4, 82.2, 81.5, 48.9, 44.3, 41.8, 40.6, 35.6, 31.6, 30.5, 28.4, 26.2, 25.8, 19.3, 18.2, 18.0, 11.9, -4.4, -4.8 ppm; IR v_{max} (film): 2988, 2850, 1773, 1722, 1450, 1377, 1268, 1141, 1075, 786, 748 cm^{-1}; HRMS (ESI) m/z calcd for $C_{23}H_{41}O_4Si$ $[M+H]^+$: 409.2769; found: 409.2771.

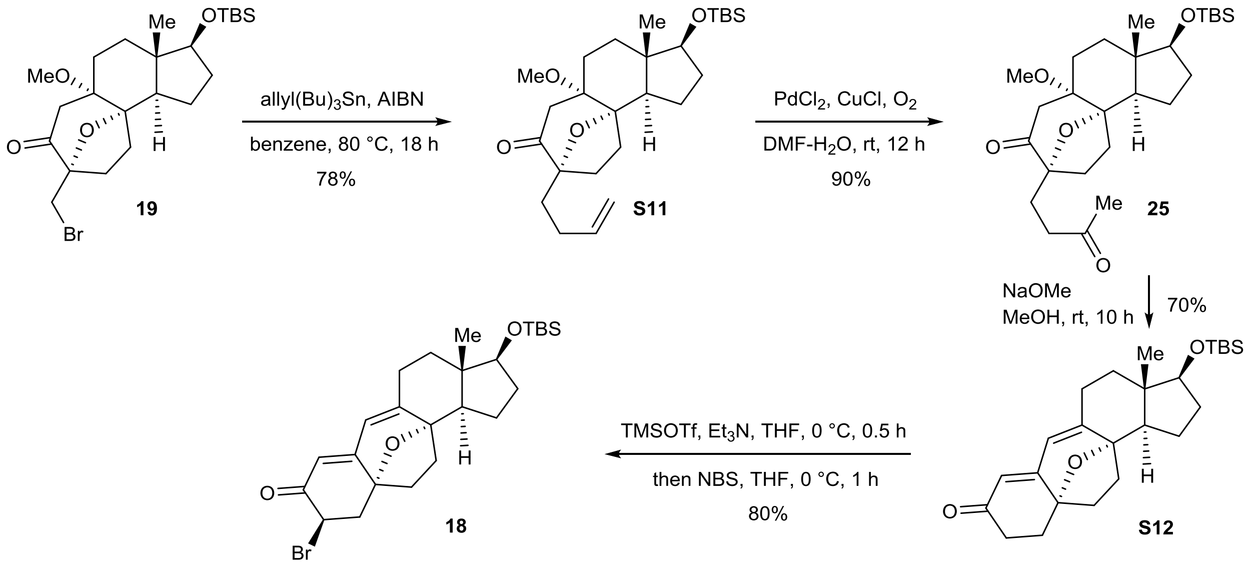

Compound S11. To a stirred solution of **19** (480 mg, 1.00 mmol, 1.0 equiv) in dry benzene (20 mL) were added sequentially with 2,2-azobis(2-methylpropionitrile) (82 mg, 0.50 mmol, 0.5 equiv) and allyltributyltin (3.1 mL, 10.0 mmol, 10 equiv) at ambient temperature. The reaction was degassed by argon, and then warmed up to 80 °C. After 18 h, the mixture was cooled to ambient temperature, and concentrated *in vacuo* to give a raffinate (about 2.0 mL), which was directly purified by flash column chromatography to afford alkene **S11** (350 mg, 0.78 mmol) in 78% yield. R_f = 0.45 (silica gel, ethyl acetate/hexanes = 1/8); $[\alpha]_D^{20}$ = -10.8 (*c* 0.3, $CHCl_3$); ^1H NMR (500 MHz, $CDCl_3$) δ = 5.82 (ddt, *J* = 17.0, 10.0, 6.5 Hz, 1H), 5.00 (dd, *J* = 17.0, 1.5 Hz, 1H), 4.91 (dd, *J* = 10.0, 1.5 Hz, 1H), 3.65 (t, *J* = 8.0 Hz, 1H), 3.18 (s, 3H), 2.67 (d, *J* = 16.5 Hz, 1H), 2.38 (dd, *J* = 12.0, 7.0 Hz, 1H), 2.22 (d, *J* = 16.5 Hz, 1H), 2.17 – 2.05 (m, 3H), 1.94 (dd, *J* = 13.0, 5.5 Hz, 1H), 1.94 – 1.83 (m, 3H), 1.82 – 1.70 (m, 3H), 1.69 – 1.58 (m, 1H), 1.56 – 1.42 (m, 3H), 1.38 (ddd, *J* = 12.5, 3.0, 3.0 Hz, 1H), 1.14 (ddd, *J* = 13.0, 3.5 Hz, 1H), 0.87 (s, 9H), 0.77 (s, 3H), 0.00 (s, 3H), -0.01 (s, 3H); ^{13}C NMR (125 MHz, $CDCl_3$) δ = 206.9, 138.8, 114.2, 86.9, 86.0, 82.2, 81.5, 48.9, 44.4, 41.9, 40.9, 32.5, 31.7, 30.5, 30.2, 28.2, 27.5,

26.2, 25.8, 19.2, 18.0, 11.9, -4.5, -4.8 ppm; IR v_{max} (film): 2990, 1769, 1759, 1377, 1244, 1159, 1125, 1092, 1057, 764, 750cm^{-1}; HRMS (ESI) m/z calcd for $C_{26}H_{44}O_4NaSi$ $[M+Na]^+$: 471.2901; found: 471.2901.

Compound 25. Alkene **S11** (330 mg, 0.74 mmol, 1.0 equiv) was dissolved in DMF/H_2O (7/1, 7.5 mL), and $PdCl_2$ (26 mg, 0.15 mmol, 0.2 equiv) and CuCl (110 mg, 1.11 mmol, 1.5 equiv) were added into the solution. The reaction was degassed by oxygen, and stirred at ambient temperature for 12 h. The mixture was diluted with water (8.0 mL), and extracted with diethyl ether (20 mL × 4). The combined organic layers were dried over Na_2SO_4 and filtered, and the solvents were removed *in vacuo*. The crude product was further purified by column chromatography on silica gel to give the diketone **25** (310 mg, 0.67 mmol) in 90% yield. R_f = 0.65 (silica gel, ethyl acetate/hexanes = 1/2); $[\alpha]_D^{20}$ = -10.1 (*c* 0.4, $CHCl_3$); ^{1}H NMR (400 MHz, $CDCl_3$) δ = 3.65 (t, *J* = 8.0 Hz, 1H), 3.18 (s, 3H), 2.67 (d, *J* = 16.4 Hz, 1H), 2.57 (dd, *J* = 6.8, 2.0 Hz, 1H), 2.54 (dd, *J* = 6.8, 2.4 Hz, 1H), 2.34 (dd, *J* = 12.0, 7.2 Hz, 1H), 2.20 (d, *J* = 16.4 Hz, 1H), 2.16 – 2.07 (m, 1H), 2.11 (s, 3H), 2.03 – 1.78 (m, 5H), 1.76 (ddd, *J* = 15.2, 3.2, 3.2 Hz, 1H), 1.63 – 1.40 (m, 5H), 1.38 (ddd, *J* = 12.8, 3.2, 3.2 Hz, 1H), 1.13 (ddd, *J* = 13.2, 13.2, 3.6 Hz, 1H), 0.86 (s, 9H), 0.76 (s, 3H), 0.00 (s, 6H); ^{13}C NMR (100 MHz, $CDCl_3$) δ = 208.7, 206.7, 87.0, 85.7, 82.1, 81.4, 48.9, 44.3, 41.9, 40.8, 38.2, 33.7, 31.6, 30.4, 29.8, 28.0, 26.1, 25.8, 25.7, 19.2, 18.0, 11.8, -4.5, -4.9 ppm; IR v_{max} (film): 2955, 2928, 1721, 1275, 1260, 1092, 872, 764, 750 cm^{-1}; HRMS (ESI) m/z calcd for $C_{26}H_{44}O_5NaSi$ $[M+Na]^+$: 487.2850; found: 487.2847.

Compound S12. To a solution of diketone **25** (250 mg, 0.54 mmol, 1.0 equiv) in anhydrous MeOH (18 mL) was added NaOMe (2.2 mL, 1.08 mmol, 2.0 equiv, 0.5 M in MeOH) at 0 °C. The reaction mixture was warmed up to ambient temperature, and after stirred for 10 h, the reaction was quenched with saturated aqueous ammonium chloride (15 mL). The organic layer was separated, and the aqueous phase was extracted with ethyl acetate (20 mL × 3). The combined organic layers were dried over Na_2SO_4, filtered, and concentrated *in vacuo*. Purification by flash column chromatography afforded the dienone **S12** (156 mg, 0.38 mmol) in 70% yield. R_f = 0.38 (silica gel, ethyl acetate/hexanes = 1/4); $[\alpha]_D^{20}$ = -35.0 (*c* 0.2, $CHCl_3$); ^{1}H NMR (400 MHz, $CDCl_3$) δ = 5.91 (d, *J* = 2.0 Hz, 1H), 5.53 (s, 1H), 3.64 (t, *J* = 8.4 Hz, 1H), 2.56 (ddd, *J* = 15.2, 15.2, 6.4 Hz, 1H), 2.53 – 2.35 (m, 4H), 2.13 – 1.97 (m, 5H), 1.92 (dd, *J* = 11.6, 8.0 Hz, 1H), 1.81 (dd, *J* = 5.6, 2.0 Hz, 1H), 1.79 – 1.75 (m, 1H), 1.68 – 1.61 (m, 2H), 1.56 – 1.48 (m, 1H), 1.23 (ddd, *J* = 13.2, 13.2, 5.2 Hz, 1H), 0.88 (s, 9H), 0.82 (s, 3H), 0.02 (s, 6H); ^{13}C NMR (100 MHz, $CDCl_3$) δ = 198.8, 160.4, 157.9, 119.7, 118.5, 84.1, 81.4, 78.0, 47.8, 43.8, 36.1, 35.6, 35.5, 33.5, 30.5, 30.4, 29.1, 25.8, 19.5, 18.0, 10.9, -4.5, -4.9 ppm; IR v_{max} (film): 2997, 1771, 1759, 1375, 1246, 1055, 764, 750 cm^{-1}; HRMS (ESI) m/z calcd for $C_{25}H_{39}O_3Si$ $[M+H]^+$: 437.2482; found: 437.2494.

Compound 18. To a solution of dienone **S12** (128 mg, 0.31 mmol, 1.0 equiv) in anhydrous THF (20 mL) at 0 °C was added triethylamine (0.43 mL, 3.10 mmol, 10 equiv), followed by the addition of trimethylsilyl trifluoromethanesulfonate (0.33 mL, 1.86 mmol, 6.0 equiv) dropwise. The reaction was stirred at 0 °C for 30 min, and then a solution of *N*-bromosuccinimide (167 mg, 0.93 mmol, 3.0 equiv) in THF (5.0 mL) was added into the solution. After 1 h at 0 °C, the reaction was quenched with saturated aqueous ammonium chloride (15 mL). The organic layer was separated, and the aqueous phase was extracted with ethyl acetate (20 mL × 3). The combined organic layers were dried over Na_2SO_4, filtered, and concentrated *in vacuo*. Purification by flash column chromatography afforded

the bromo ketone **18** (122 mg, 0.25 mmol) in 80% yield.[12-13] R_f = 0.55 (silica gel, ethyl acetate/hexanes = 1/4); $[\alpha]_D^{20}$ = 4.5 (*c* 0.2, $CHCl_3$); ^{1}H NMR (400 MHz, $CDCl_3$) δ = 5.92 (s, 1H), 5.61 (s, 1H), 4.59 (br, 1H), 3.64 (t, *J* = 8.3 Hz, 1H), 2.92 (dd, *J* = 15.0, 4.6 Hz, 1H), 2.58 – 2.36 (m, 4H), 2.35 – 2.27 (m, 1H), 2.08 (ddd, *J* = 11.3, 11.3, 5.7 Hz, 1H), 2.03 – 1.94 (m, 1H), 1.90 (t, *J* = 9.6 Hz, 1H), 1.84 – 1.76 (m, 2H), 1.67 – 1.52 (m, 3H), 1.23 (ddd, *J* = 12.8, 12.8, 5.2 Hz, 1H), 0.88 (s, 9H), 0.83 (s, 3H), 0.02 (s, 6H); ^{13}C NMR (100 MHz, $CDCl_3$) δ = 191.2, 160.3, 159.0, 119.4, 116.5, 83.4, 81.4, 76.0, 47.7, 44.7, 43.9, 41.1, 37.2, 36.1, 30.6, 29.9, 29.3, 25.8, 19.4, 18.1, 10.9, -4.5, -4.9 ppm; IR ν_{max} (film): 2953, 2928, 2860, 2843, 1645, 1633, 1411, 1396, 1250, 1053, 1032, 1016, 781 cm^{-1}; HRMS (ESI) m/z calcd for $C_{25}H_{37}O_3NaSiBr$ $[M+Na]^+$: 515.1588; found: 515.1587.

Supplementary References:

1. Becke, A. D. Density-functional thermochemistry. III. The role of exact exchange. *J. Chem. Phys.* ***98***, 5648-5642 (1993).
2. Lee, C.; Yang, W. & Parr, R. G. Development of the Colle-Salvetti correlation-energy formula into a functional of the electron density. *Phys. Rev. B* ***37***, 785-789 (1988).
3. Peverati, R. & Truhlar, D. G. Exchange–correlation functional with good accuracy for both structural and energetic properties while depending only on the density and its gradient. *J. Chem. Theory Comput.* ***8***, 2310-2319 (2012).
4. Marenich, A. V.; Cramer, C. J. & Truhlar, D. G. Universal solvation model based on solute electron density and on a continuum model of the solvent defined by the bulk dielectric constant and atomic surface tensions. *J. Phys. Chem. B* ***113***, 6378-6396 (2009).
5. Solaja, B. A.; Milic, D. R. & Gasic, M. J. A novel *m*-CPBA oxidation: *p*-quinols and epoxyquinols from phenols. *Tetrahedron Lett.* ***37***, 3765-3768 (1996).
6. Desmaële, D. & d'Angelo, J. Enantioselective synthesis of oxa-spiro compounds. *Tetrahedron Lett.* ***30***, 345-348 (1989).
7. Schneider, H.-J.; Gschwendtner, W. & Weigand, E. F. Conformational relaxation as limitation of chemical models. Empirical force field calculations and ^{13}C NMR shielding effects for some

cyclohexanes, bicyclo[2.2.1]heptanes, bicyclo[3.3.l]nonane, and 11 β-substituted estrenes. *J. Am. Chem. Soc.* ***101***, 7195-7198 (1979).

8. Schneider, H.-J. & Hoppen, V. Carbon-13 nuclear magnetic resonance substituent-induced shieldings and conformational equilibria in cyclohexanes. *J. Org. Chem.* ***43***, 3866-3873 (1978).
9. Lee, H. M.; Nieto-Oberhuber, C. & Shair, M. D. Enantioselective synthesis of (+)-cortistatin A, a potent and selective inhibitor of endothelial cell proliferation. *J. Am. Chem. Soc.* ***130***, 16864-16866 (2008).
10. Kotoku, N.; Sumii, Y.; Hayashi, T. & Kobayashi, M. Synthetic study of carbocyclic core of cortistatin A, an anti-angiogenic steroidal alkaloid from marine sponge. *Heterocycles* ***83***, 1535-1552 (2011).
11. Markó, I. E.; Ates, A.; Gautier, A.; Leroy, B.; Plancher, J.-M.; Quesnel, Y. & Vanherck, J.-C. Cerium(IV)-catalyzed deprotection of acetals and ketals under mildly basic conditions. *Angew. Chem. Int. Ed.* ***38***, 3207-3209 (1999).
12. Flyer, A. N.; Si, C. & Myers, A. G. Synthesis of cortistatins A, J, K and L. *Nature Chem.* ***2***, 886-892 (2010).
13. Fang, L.; Chen, Y.; Huang, J.; Liu, L.; Quan, J.; Li, C.-C. & Yang, Z. Formal synthesis of cortistatins. *J. Org. Chem.* ***76***, 2479-2487 (2011).
